# Type II Toxin-Antitoxin Distribution and Adaptive Aspects on *Xanthomonas* Genomes: Focus on *Xanthomonas citri*

**DOI:** 10.3389/fmicb.2016.00652

**Published:** 2016-05-10

**Authors:** Paula M. M. Martins, Marcos A. Machado, Nicholas V. Silva, Marco A. Takita, Alessandra A. de Souza

**Affiliations:** Centro de Citricultura Sylvio Moreira, Instituto AgronômicoCordeirópolis, Brazil

**Keywords:** anti-addiction model, citrus canker, death factor, EDF, gene loss

## Abstract

Prokaryotic toxin-antitoxin (TA) systems were first described as being designed to prevent plasmid loss in bacteria. However, with the increase in prokaryotic genome sequencing, recently many TAs have been found in bacterial chromosomes, having other biological functions, such as environmental stress response. To date, only few studies have focused on TA systems in phytopathogens, and their possible impact on the bacterial fitness. This may be especially important for pathogens like *Xanthomonas* spp., which live epiphytically before entering the host. In this study, we looked for TA systems in the genomes of 10 *Xanthomonas* strains. We verified that citrus-infecting pathovars have, on average, 50% more TAs than other *Xanthomonas* spp. and no genome harbors classical toxins such as MqsR, RelB, and HicA. Only one TA system (PIN_VapC-FitB-like/SpoVT_AbrB) was conserved among the *Xanthomonas* genomes, suggesting adaptive aspects concerning its broad occurrence. We also detected a trend of toxin gene loss in this genus, while the antitoxin gene was preferably maintained. This study discovers the quantitative and qualitative differences among the type II TA systems present in *Xanthomonas* spp., especially concerning the citrus-infecting strains. In addition, the antitoxin retention in the genomes is possibly related with the resistance mechanism of further TA infections as an anti-addiction system or might also be involved in regulation of certain specific genes.

## Introduction

*Xanthomonas* genus is known to infect a wide range of plants like crucifers, rice, cassava, tomato, sugarcane, and citrus, causing disease resulting in a considerable economic loss (Vurro et al., 2010). Many *Xanthomonas* strains carry extra-chromosomal DNA in the form of plasmids. They can confer a myriad of possible benefits for the host bacteria, like antibiotic resistance and virulence traits. Plasmids are stably transmitted through generations via certain maintenance systems such as partition systems, multimer resolution, and toxin-antitoxin systems (TAs; Sengupta and Austin, 2011). TAs were initially discovered during 1980's as “post-segregational killing systems” composed of single operon-encoded proteins for both toxin and its cognate antitoxin, leading to cell death when the plasmid is lost (Ogura and Hiraga, 1983; Gerdes et al., 1986). The basic mechanism, behind this successful strategy to avoid plasmid loss in populations, is based on the biochemical properties of the protein pair, where one counterpart, antitoxin, is labile, and the other, toxin, is stable. During the cell division, both the daughter cells receive pre-made toxin and antitoxin. However, only the cells that have also received the plasmid carrying the TA genes will be able to keep synthesizing the antitoxin, and therefore, will survive (Gerdes et al., 2005). Thus, TAs may be important in maintaining adaptive traits present on plasmids, that otherwise would be lost by bacterial populations under a non-selective pressure.

Despite its undeniable role in plasmid maintenance within prokaryotic cells, many other functions are currently assigned to the TA system activity profile. With the increasing number of bacterial genomes being sequenced, several novel TA pairs have been found in chromosomes, where they perform different functions such as the stabilization of genomic regions, defense against phage, and plasmid infection, biofilm formation, programmed cell death, and induction of persister cells (Van Melderen, 2010).

Most prokaryotic genomes of free-living bacteria have at least one TA system (Pandey and Gerdes, 2005); and several different toxin families have been described with varying cellular targets. Although new information on its molecular functioning is continuously being added, most toxins are endoribonucleases that act on free or ribosome-associated mRNAs (RelE, MazF, HigB, HicA, VapC; Christensen and Gerdes, 2003; Christensen et al., 2003; Daines et al., 2007; Hurley and Woychik, 2009; Jørgensen et al., 2009); while few others target DNA gyrases (CcdB, ParE), tRNA synthetases (HipA), EF-Tu (Doc), and even peptidoglycan precursors (ζ) (Bahassi et al., 1999; Jiang et al., 2002; Mutschler et al., 2011; Castro-Roa et al., 2013; Germain et al., 2013; Kaspy et al., 2013).

There are five different types of TA systems, where type II is the most studied one, which is the main system involved in cellular processes such as persister cells formation (Gerdes and Maisonneuve, 2012; Goeders and Van Melderen, 2014). The majority of the studies regarding type II TA systems are related to human-associated bacteria, and only a few have addressed TAs from phytopathogens. Some studies have found TAs in *Xylella fastidiosa* (Lee et al., 2012; Muranaka et al., 2012) and *Erwinia pyrifoliae* (Unterholzner et al., 2013) to be involved in formation of persister cells (Ayrapetyan et al., 2015). Considering the economic impact of *Xanthomonas* spp. on several crops, such as rice, tomato, and citrus, we carried out an *in silico* analysis of ten completely sequenced and annotated genomes of *Xanthomonas*, and found that citrus-infecting pathovars have more TA operons than other strains. In addition, we show that although the distribution of toxin superfamilies is very similar on the chromosomes of different strains, there are many toxin deletions with the maintenance of only cognate antitoxin. This antitoxin retention is possibly rendering *Xanthomonas* strains resistant to further TA infections as an anti-addiction system (Saavedra De Bast et al., 2008) or, in addition, is involved in *trans* regulation of genes related to important processes such as biofilm and mobility (Li and Wang, 2011; Wang and Wood, 2011).

## Materials and methods

### Searching for toxin and antitoxin systems

A web-based program, RASTA (Sevin and Barloy-Hubler, 2007), was used to search the genomes for TA systems. This program was able to find TA systems not only on the 14 genomes tested by Sevin and Barloy-Hubler (2007), but also on other prokaryotic genomes (Muranaka et al., 2012). All genomes were scanned and proteins with a minimum score of 60 were selected for a further bioinformatics analysis. We also referred to the TA Database, and analyzed the available *Xanthomonas* genomes (Shao et al., 2011). Due to the differences observed between these programs, BLASTp search (*E* < 10^−4^) was also carried out and all TA proteins were used as the query sequence against the proteins from the studied *Xanthomonas* genomes. The gene pairs were manually curated and those not clearly related to this topic, such as transposases and the proteins whose domains are not related to toxins or antitoxins, were excluded. Unknown genes carrying possibly unknown domains, such as when along with a toxin, were considered as special cases.

The toxin domain analysis was based on the Conserved Domains Database (CDD; Marchler-Bauer et al., 2014); and all putative proteins were scanned to determine their domains. The further analysis of domain architecture was done using the Domain Architecture Analysis with CDART (Geer et al., 2002).

Only the TA pairs that were considered “complete” were added to further analysis. The presence of toxin domain in one gene of the pair was obligatory, though exceptions were considered for the putative Zeta toxin and HipA found in *Xanthomonas campestris* for the reasons explained in the text. All these solitary toxins or antitoxins are detailed in Table S2.

### The analysis of distribution and prevalence of TAs in *Xanthomonas* spp.

The complete TA pairs were counted for each strain and a radar graph depicting these numbers was plotted using the freely available “Online Chart Tool” (www.onlinecharttool.com). The prevalence of TA domains identified in the CDD analysis was tabulated and visualized by the online software “CIRCOS” (www.circos.ca).

### The analysis of the PIN_VapC-FitB-like/SpoVT/AbrB TA pair

An analysis for the amino acid sequence similarity of PIN_VapC-FitB-like/SpoVT/AbrB TA pair among the different *Xanthomonas* strains was done by taking XAC306 sequence as a reference in BLASTp. The bit score results obtained from the alignments with fixed *E* < 10^−10^ were visualized by the tool “Circoletto” (http://tools.bat.infspire.org/circoletto/; Darzentas, 2010).

To asses if these TA pairs were under positive or negative selection, a dN/dS ratio analysis was carried out, which is widely used to quantitatively determine the prevalence of non-synonymous (dN) over synonymous (dS) mutations. A dN/dS ratio below one (ω < 1) indicates that this gene is under purifying selection, which means that protein changes are being suppressed. If the ratio is above one (ω > 1) the opposite is true, i.e., natural selection is favoring the appearance of new protein variants. This analysis was carried out by employing MEGA 6.0 and using the gene sequences of the toxins and antitoxins previously selected.

### The analysis of missing toxins in genus *Xanthomonas*

To check if our results are biased by the strain selection, we run Delta-BLAST against the entire genus *Xanthomonas* (total of 369 strains) using the following missing proteins from *Escherichia coli* as a query: WP_000833473.1 (HicA/YcfA); WP_038988657.1 (MqsR); WP_050939938.1 (RelE). It is important to highlight that the RelE domain, we attempted to find (COG2026), was virtually absent, but a similar domain (plasmid_stabil–pfam05016) was found broadly among *Xanthomonas* genomes.

### Synteny analysis on chromosomes and plasmids

To assess whether the TA genes were aligned at the same genomic regions throughout the strains, we did a synteny analysis using the Synttax bioinformatics tool with ABSYNTE algorithm (http://archaea.u-psud.fr/synttax/; Despalins et al., 2011; Oberto, 2013) using the ten *Xanthomonas* genomes as reference and fourteen plasmids. When necessary (as specified in the text), we added the genomes of other members of Xanthomonadaceae, e.g., *Xylella* sp., for the comparison.

### RNA extraction and RT-qPCR

An overnight culture of *Xanthomonas citri* 306 was diluted to OD ~0.2 in 5 mL of NBY (0.5% peptone, 0.3% meat extract, 0.2% yeast extract, 0.2% K_2_HPO_4_, 0.05% KH_2_PO_4_), and maintained at 28°C and 180 rpm. After 2 h, an aliquot of 1.5 mL of this culture was transferred into a 2-mL Eppendorf tube and was subjected to a non-lethal heat stress (37°C for 2 h; Sumares et al., 2016) or copper oxychloride (18 μg/mL for 3 h; Picchi et al., 2016). The cells were subsequently harvested and RNA was extracted with the RNeasy Extraction Kit (Qiagen) followed by elimination of total DNA using an on-column RNase-Free DNase set (Qiagen). A total amount of 1 μg was used for cDNA using GoScript (Promega). qPCR was carried out in a 7500 Fast Real Time PCR machine (Applied Biosystems) and the reactions mixtures were made using the GoTaq qPCR Master Mix (Promega). The *X. citri* ribosomal 16S expression was used as an endogenous control to normalize gene expression. After each run, the melting curve was analyzed to ensure that the threshold values obtained are originating from a unique PCR product. The relative expression analysis was calculated from the Ct values obtained using the formula 2^(−ddCT)^. The ddCt was obtained as follows: dC_T_ = C_T_(target gene)—C_T_ (endogenous control); ddC_T_ = dCT (treatment)—dCT (reference). Student's *t*-test was used to assess the significance of the data (*P* < 0.05). For the analysis of the gene expressions following genes were used: XACa0027, XACa0028, XAC1499, XAC1501, and XAC4314. The primers sequences, shown in Table S3, were designed using the Primer Express software version 2.0 (Applied Biosystems). To evaluate the efficiency of the primers, standard curves were constructed and the slope of these curves was used to calculate the efficiency according to the formula *E* = 10^(−1/slope)^ − 1. The encoding gene of the toxin XAC4315 could not be used as its primers did not meet the above mentioned efficiency criteria. Each RT-qPCR reaction was carried out in duplicate and each condition had three biological replicates.

## Results

### Citrus pathovars showed higher number of TA systems

**Figure 1:**
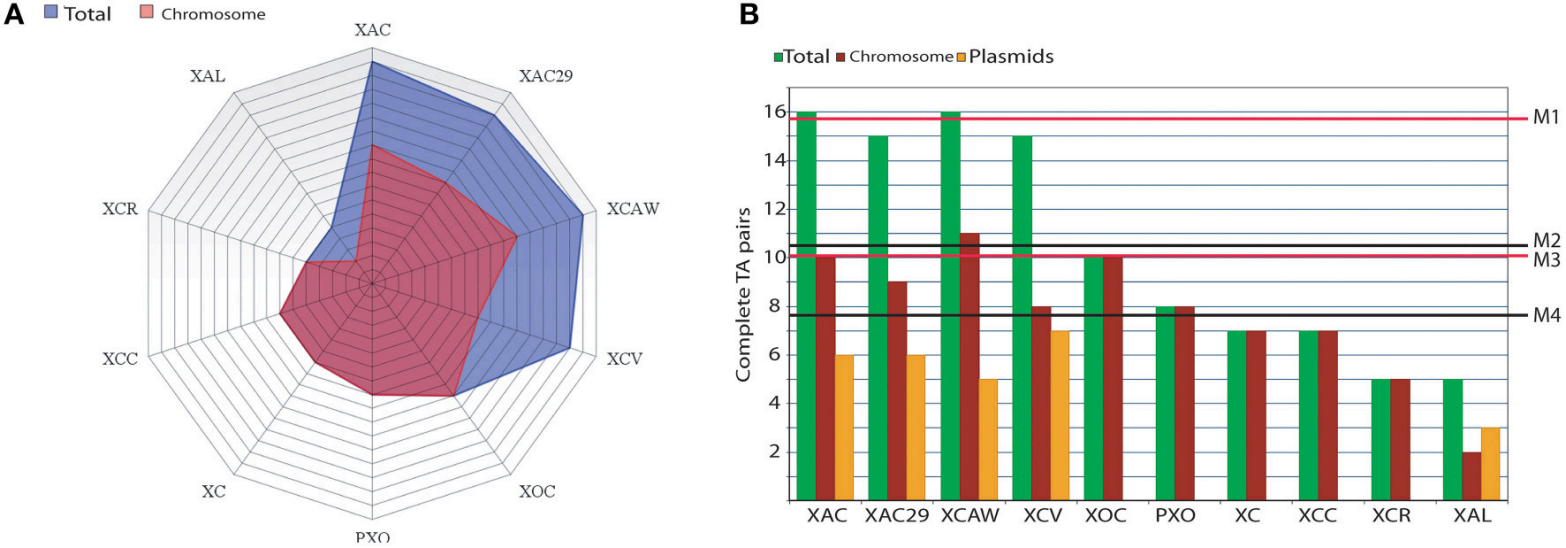
**Abundance of TAs in *Xanthomonas* spp. (A)** A radar graph showing the abundance of complete TA systems found in the genomes of the 10 *Xanthomonas* strains. Each line on the grid represents one TA pair. Blue area represents the totality of TAs in both plasmids and chromosomes while the pink area shows the abundance of TAs on the chromosomes. **(B)** A bar graph showing the complete TA pairs found in the whole genome (green bars; for each genome); chromosome only (red bars) and plasmids (orange bars). The mean values of TA per genome are M1 = 15.6 (for the Citrus-infecting strains XAC, XAC29, and XCAW) and M2 = 10.4 (accounting the ten strains). The mean values for TAs per chromosome (i.e., excluding plasmids TAs) are M3 = 10 (only Citrus-infecting strains XAC, XAC29, and XCAW) and M4 = 7.7 (for the 10 chromosomes). XAC (*Xanthomonas citri* subsp. *citri* 306); XAC29 (*Xanthomonas axonopodis* Xac29-1); XCAW (*Xanthomonas citri* subsp. *citri* Aw12869); XCV (*Xanthomonas campestris* pv. *vesicatoria* 85-10); XOC (*Xanthomonas oryzae* pv. *oryzicola* BLS256); PXO (*Xanthomonas oryzae* pv. *oryzae* PXO99A); XC (*Xanthomonas campestris* pv. *campestris* 8004); XCC (*Xanthomonas campestris* pv. *campestris* ATTC33913); XCR (*Xanthomonas campestris* pv. *raphani* 756C); XAL (*Xanthomonas albilineans* GPE PC73).

The overall quantitative distribution of complete TA operons among *Xanthomonas* is shown in Figure 1A (blue area). Each line on the grid represents a complete TA pair on the genomes. It is noteworthy that the three *Citrus* infecting pathovars showed the highest number of complete TA pairs with a mean value of 15.6 TAs per genome (Figure 1B—M1), which is higher than 10.4 observed when all *Xanthomonas* genomes are considered together (Figure 1B—M2). This value is still higher in this pathovar even when the plasmids are excluded from the analysis (Figure 1A—pink area), reaching the mean value of 10 TAs per genome (Figure 1B—M3), while the overall TA per chromosome ratio is 7.7 for the *Xanthomonas* strains (Figure 1B—M4). *X. campestris* pv. *vesicatoria* has a high global number of TA operons (15), and without the plasmids this value is 8, which is very close to the 7.7, the average number found in other *Xanthomonas* spp., and still under the mean value of 10 found in the chromosomes of Citrus-infecting pathovars.

The presence of plasmids can be pointed as one of the main sources of TA operons in *Xanthomonas*, since almost all plasmid-harboring strains (XAC, XAC29, XCAW, XCV) have more TAs in comparison to the plasmid-free strains (XCC, XC, PXO, XOC, and XCR), except *Xanthomonas albilineans* that has a lower TA number (Figure 1A).

### *Xanthomonas* strains show different sets of toxin families

Different toxin families were found to be prevalent when chromosomes and plasmids were analyzed. Plasmids were indeed responsible for the great extent of the TA pool found in *Xanthomonas* genomes (Figure 1A), but its presence was not a rule. Only half of the ten genomes analyzed had these extra-chromosomal DNA (*X. citri* strains, *X. campestris* pv. *vesicatoria* and *X. albilineans;* Table 1—Column 1). Almost all the 14 plasmids analyzed showed at least one TA system, except for pXCV2 (in *X. campestris pv. vesicatoria*) that did not show the presence of any TA.

**Table 1:** ***Xanthomonas* spp. used in this work**.

***Xanthomonas* Strain/Abbreviation/[Native plasmids]**	**Host/Tissue colonized**	**Genome references**
*Xanthomonas citri* subsp. *citri* str. 306 (taxid:190486)/*X. citri* 306/[pXAC33/pXAC64]	Citrus/Mesophyll	da Silva et al., 2002
*Xanthomonas axonopodis* Xac29-1 (taxid:1304892)/*X. citri* 29-1/[pXAC33/pXAC47/pXAC64]	Citrus/Mesophyll	Unpublished
*Xanthomonas citri* subsp. *citri* Aw12879 (taxid:1137651)/*X. citri* Aw/[pXCaw19/pXCaw58]	Mexican lime/Mesophyll	Jalan et al., 2013
*Xanthomonas campestris* pv. *vesicatoria* str. 85-10 (taxid:316273)/ *X. campestris* pv. *vesicatoria*/[pXCV2/pXCV19/pXCV38/pXCV183]	Tomato/Mesophyll	Thieme et al., 2005
*Xanthomonas campestris* pv. *raphani* 756C (taxid:990315)/*X. campestris*. pv. *raphani*	Crucifers/Mesophyll	Bogdanove et al., 2011
*Xanthomonas campestris* pv. *campestris* str. ATCC 33913 (taxid:190485)/*X. campestris* ATCC 33913	Crucifers/Vascular	da Silva et al., 2002
*Xanthomonas campestris* pv. *campestris* str. 8004 (taxid:314565)/*X. campestris* 8004	Crucifers/Vascular	Qian et al., 2005
*Xanthomonas albilineans* GPE PC73 (taxid:380358)/*X. albilineans*/[plasmI/plasmII/plasmIII]	Sugarcane/Vascular	Pieretti et al., 2009
*Xanthomonas oryzae* pv. *oryzicola* BLS256 (taxid:383407)/*X. oryzae* pv. *oryzicola*	Rice/Mesophyll	Bogdanove et al., 2011
*Xanthomonas oryzae* pv. *oryzae* PXO99A (taxid:360094)/*X. oryzae* pv. *Oryzae*	Rice/Vascular	Salzberg et al., 2008

*Columns: (1) the strains name and taxid number is followed by the abbreviation utilized throughout this work. Native plasmids (if present) are shown in brackets. (2) host and tissue preferably colonized by these Xanthomonas spp. (3) genome reference*.

**Figure 2:**
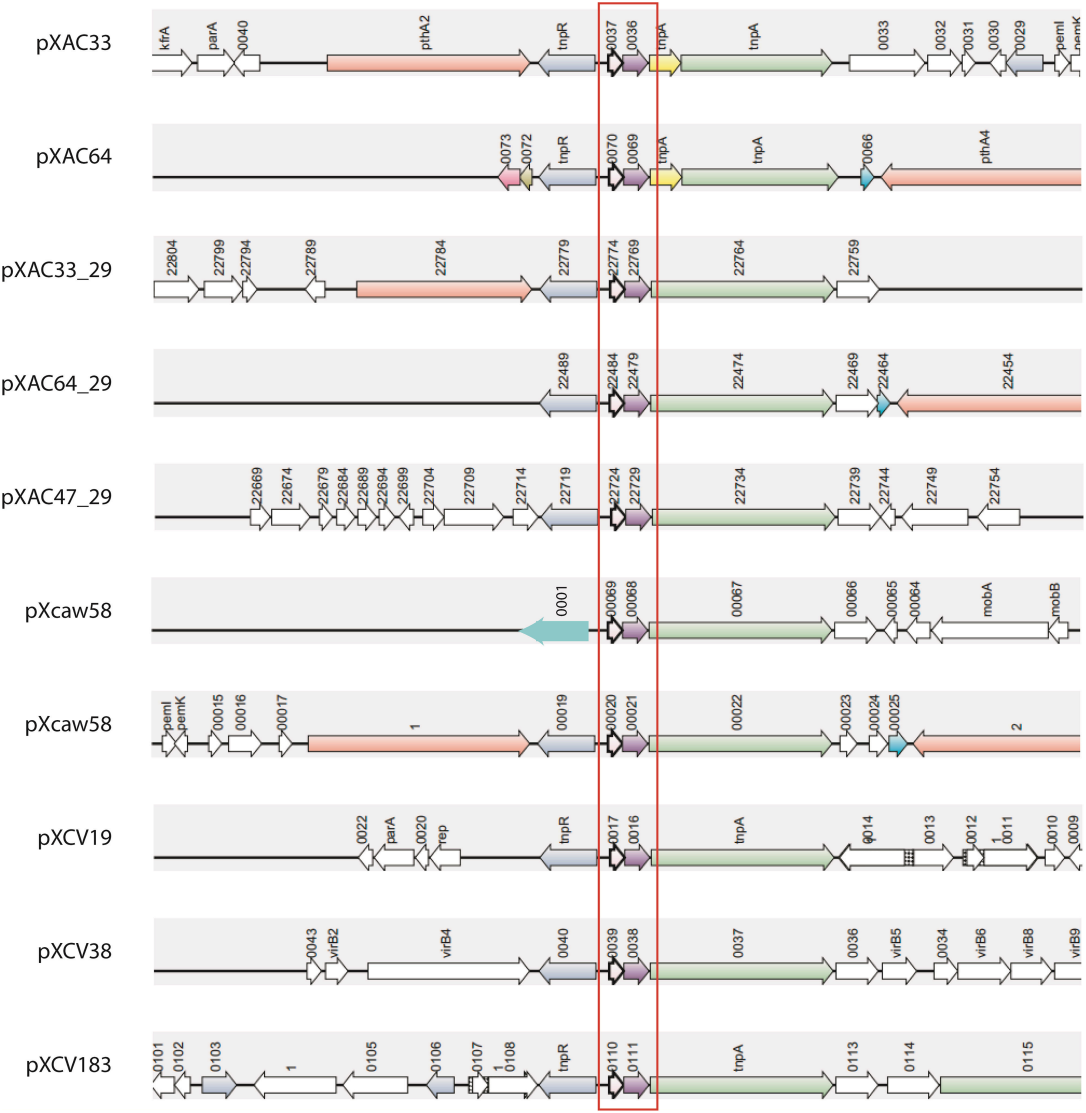
**Synteny of the most common TA operons found on plasmids**. Ten copies of this TA system were found in nine of the 14 plasmids analyzed. This TA operon (red box) is always flanked by transposases (green and blue arrows). Four *Xanthomonas* strains are represented above: XAC (pXAC33, pXAC64); XAC29 (pXAC33_29, pXAC64_29, pXAC47_29); XCAW (pXcaw58); and XCV (pXCV184, pXCV38, pXCV19). XAL is the unique plasmid-harboring strain that does not have any copy of this TA system.

Nine of these 14 plasmids share a plasmid-exclusive TA system (Figure 2, Table S1). The toxin found in this system has a COG1569 domain, which is a member of the PIN superfamily (cl14812). No specific function has been assigned to this COG, but since the PIN superfamily encompasses nucleases, it is possible that this toxin may function as an RNA interferase. At least two copies of this TA were found in each plasmid-harboring *Xanthomonas*, except for *X. albilineans*, wherein this TA was absent. Although in most cases, the copy number for this TA was one per plasmid, in pXCaw58 (*X. citri* Aw) two operons were found. This TA was conserved, but the genomic contexts in which it appeared showed differences. However, in all cases this TA system was flanked by two transposases, suggesting that they moved by transposon in different *Xanthomonas* plasmids (Figure 2). The structure of the antitoxin has been unveiled, which shows that this protein has a domain belonging to the RHH superfamily of DNA binding domains (Gallo et al., 2010). Besides this, no more information is available for this TA system, remaining it an open question, why it appears in so many *Xanthomonas* plasmids.

We also found an unannotated pair of TA in *X. citri* 306 plasmid pXAC33 named as *Rorf 81/Rorf 85* (Table S1), also present in pXAC33 in *X. citri* 29-1, plasmIII of *X. albilineans* and in the chromosomes of the two *X. campestris* pv. *campestris* strains. Interestingly, in these chromosomes, this TA was found inserted in genomic islands (putative genomic island in XCC: 1,861,662—1,914,670 and in XC: 3,122,997—3,180,603).

One TA system was exclusively present on the plasmids of Citrus-infecting pathovars (Table S1). In contrast to other *Xanthomonas* strains, the presence of the toxin ChpB (PRK09812 domain/PemK superfamily) was restricted only to sweet-orange infecting strains (*X. citri* 306 and *X. citri* 29-1), being absent in *X. citri* Aw, which is limited to Mexican Lime and Alemow (Jalan et al., 2013). It still remains unknown whether the absence or presence of this toxin confers any fitness to the host (sweet orange or Mexican lime).

Another toxin domain exclusively found on plasmid was ζ (pfam06414). In this specific case, it is not possible to say if this TA system is functional, since it deviates from the criteria described for this TA system. Usually ζ toxins appear in an operon composed of three genes: ω, ϵ, and ζ, where ω is the regulator, ϵ is the antitoxin, and ζ is the toxin (Mutschler and Meinhart, 2011). However, the operon we found on the *Xanthomonas* plasmid lacks the ω gene; and the ϵ gene upstream of ζ does not have any domain assigned to it. An alternate operon consisting of only two genes has already been described (Khoo et al., 2007) but, in this case, the gene upstream the toxin performs both regulator and antitoxin functions. Due to such unusual operon configurations in *Xanthomonas*, this TA system was not considered as a normal TA (Table S2) and, therefore, was not included in the analysis. Whether this TA system is inactive or having a novel operon configuration (with a to-be-described antitoxin domain) remains to be discovered.

**Figure 3:**
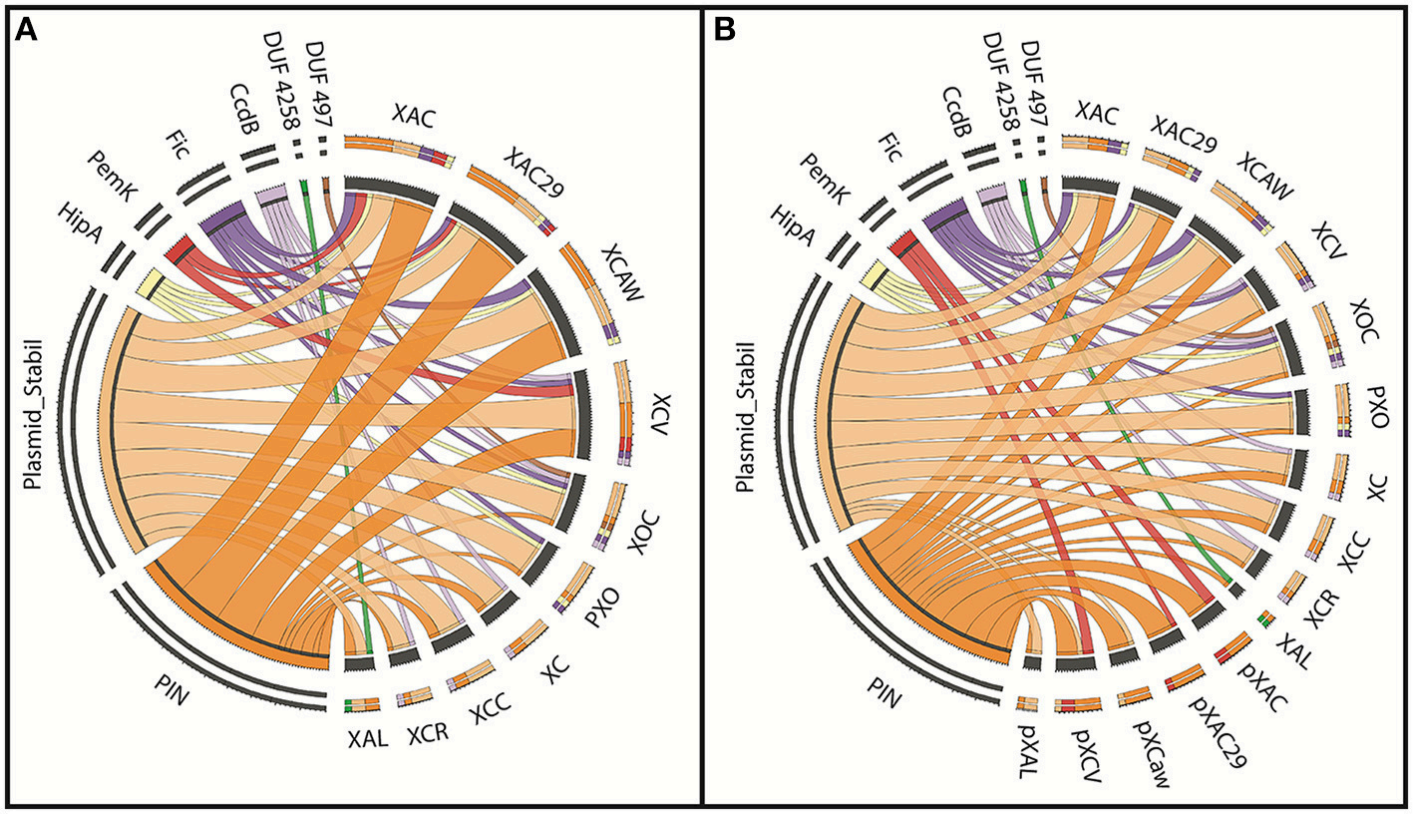
**The prevalence of TAs in each *Xanthomonas* strain. (A)** The visual representation of the toxin superfamilies found (on the left) among the ten genomes analyzed (on the right). Each color represents one superfamily and the wider the ribbons, the larger the number of toxins contained in this domain. **(B)** This is same to the panel A, but in this panel the genome toxin contents of the chromosome and plasmids are visualized separately. XAC (*X. citri* 306); XAC29 (*X. citri* Xac29-1); XCAW (*X. citri* Aw); XCV (*X. campestris* pv. *vesicatoria*); XOC (*X. oryzae* pv. *oryzicola*); PXO (*X. oryzae* pv. *oryzae*); XC (*X. campestris* 8004); XCC (*X. campestris* ATTC33913); XCR (*X. campestris* pv. *raphani*); XAL (*X. albilineans*); pXAC (plasmids of *X. citri* 306); pXAC29 (plasmids of *X. citri* Xac29-1); pXCaw (plasmids of *X. citri* Aw); pXCV (plasmids of *X. campestris* pv. *vesicatoria*); pXAL (plasmids of *X. albilineans*).

Eight superfamilies were identified, six of them had known toxin domains and the other two with undetermined functions [DUF497 (Maj et al., 2013) and DUF4258] (Table S1). A visual representation of the toxin domains found is shown in Figure 3A, where each color on the left panel represents one domain superfamily and on the right their respective contribution to the toxin pool for each strain is also shown. It is possible to note that Citrus-infecting strains are clearly distinct from other *Xanthomonas* spp., since the main superfamily found within these genomes is the ribonuclease *PIN* (cl14812) (Arcus et al., 2004; Figure 3A—orange), while other *Xanthomonas* strains had the *Plasmid_stabil* (cl21503) (Figure 3A—light orange) as the prevalent toxin superfamily. *X. albilineans* shows equal numbers of both toxin superfamilies thus there is no prevalence.

When plasmids and chromosomes have their toxin superfamily content separately visualized (Figure 3B), it is possible to see more similarities among *Xanthomonas* spp. chromosomes. The occurrence of the *Plasmid_stabil* superfamily (cl21503) (Figure 3B—light orange) is increased in *X. citri*, while the *PIN* superfamily (cl14812) (Figure 3B—orange) prevalence is limited only to the plasmids. In Figure 3B is also noticed that the toxin content in the plasmids is similar, with a maximum of two toxin superfamilies. The PemK superfamily (Figure 3B—red), is present only in the extra-chromosomal DNA, but the opposite is true about the other toxins superfamilies such as Fic (Figure 2B—purple), CcdB (Figure 3B—lilac) and the two domains of unknown function, DUF497 (Figure 3B—brown) and DUF4258 (Figure 3—green), which are exclusively found on the chromosomes.

The superfamily, which is more prevalent in *Xanthomonas* chromosomes, *Plasmid_stabil* (Figure 3B—light orange), includes classical toxin domains like ParE (COG3668) and HigB (COG3549), which are seen in some strains of *Xanthomonas* (Table S1). However, the well-known starvation-related toxin RelE (COG2026) (Christensen et al., 2001) was not found in any *Xanthomonas* genome. Although, a similar domain (pfam05016) (Anantharaman and Aravind, 2003) was found as best hit for five types of TA systems found in *Xanthomonas*; however, the mode of action and function of these proteins remain to be determined.

Other toxin domains, such as HicA (COG1724) and MqsR (cd12869), were not found among the ten *Xanthomonas* genomes evaluated. To confirm that this outcome is not biased due to strain selection, we run a Delta-BLAST and BLASTp search against the entire genus *Xanthomonas* (a total of 369 draft and annotated genomes) using the copies of these missing proteins from *E. coli* as query sequence. We found only one significant hit for HicA (COG1724—YcfA/HicA-like) in *Xanthomonas translucens* (WP_039008097.1) and RelE (COG2026) in *Xanthomonas axonopodis* pv. *vasculorum* (KGE50220.1) and two hits for MqsR *Xanthomonas fuscans* pv. *fuscans* (NB99_09060) and *X. axonopodis* pv. *phaseoli* (NY94_11655). These results show that these domains are indeed underrepresented in *Xanthomonas*, and also that the strains selected in this work seem to be representative of TA profiles in this genus. The complete result of the BLASTp analysis showing common TA systems among *Xanthomonas* spp. can be observed in Table S1 and the alignments showing the similarity are shown in Data Sheet 1.

**Figure 4:**
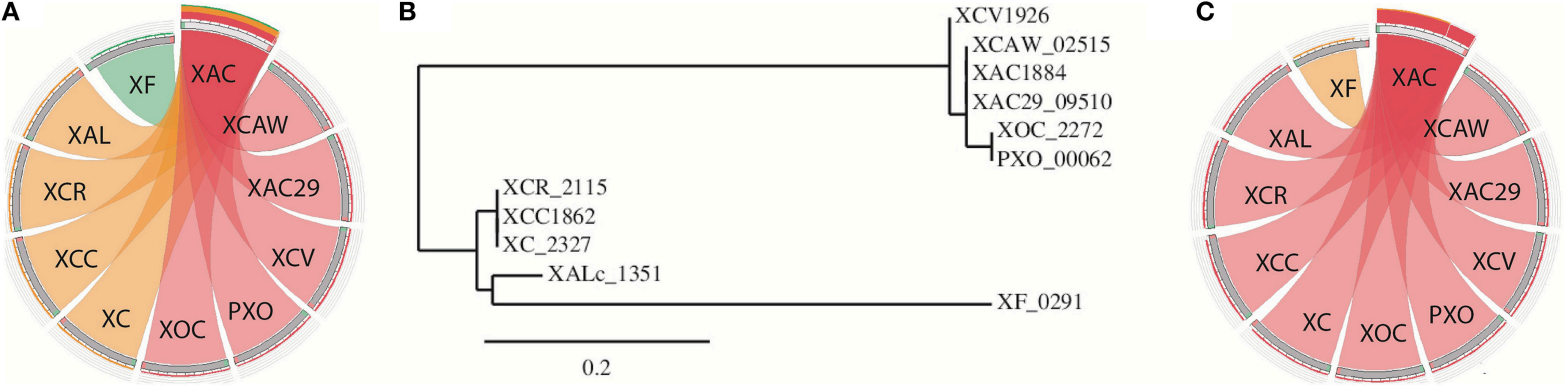
**The analysis of the single TA pair that is present in all *Xanthomonas* genomes. (A)** BLASTp visualization of the protein sequence of the toxin. XAC was used as a query against the other genomes. The pink color represents sequences that are more similar (bitscores >75%) than those that are in orange (bitscores 50–75%) or green (bitscores 25–50%). **(B)** Phylogenetic tree designed using the toxin protein sequence. Names represent the toxin tag name on NCBI; **(C)** the same as in item A but now using the antitoxin protein sequence. XAC (*X. citri* 306); XAC29 (*X. citri* Xac29-1); XCAW (*X. citri* Aw); XCV (*X. campestris* pv. *vesicatoria*); XOC (*X. oryzae* pv. *oryzicola*); PXO (*X. oryzae* pv. *oryzae*); XC (*X. campestris* 8004); XCC (*X. campestris* ATTC33913); XCR (*X. campestris* pv. *raphani*); XAL (*X. albilineans*).

Only one of the TA systems found was present in every *Xanthomonas* strains (Table S1). This TA system is composed of a toxin domain *PIN_VapC-FitB-like* (cd09875) and an antitoxin domain *SpoVT/AbrB* (smart00966; hereinafter called as PIN/SpoVT). The putative toxin of this TA system is very similar in the different genomes, being in the same genomic context (Figure S1), but its sequence is not identical. Interestingly, the differences shown by this protein increases with the phylogenetic distance between the strains (Figures 4A,B). The dissimilarity based on different color-codes is clearly noticeable, where the pink color represents the most similar sequences and the green the most different ones. The phylogenetic tree, drawn using the amino-acid sequence of this toxin (Figure 4B), reestablishes the evolutionary proximity configuration observed among *Xanthomonas* spp. (Jalan et al., 2013). The protein sequences of the antitoxins showed no significant difference in the *Xanthomonas* strains (Figure 4C). It can be gleaned that some selective pressure favored an unaltered maintenance of the antitoxin, while mutations in the toxin gene were allowed. We therefore conducted a dN/dS ratio analysis, and verified that this toxin is under a positive selection, with a prevalence of non-synonymous (dN = 0.260) over synonymous mutations (dS = 0.174), resulting in ω = 1.49. However, the antitoxin is under negative selection with much fewer non-synonymous (dN = 0.086) than synonymous mutations (dS = 0.550), resulting in ω = 0.15.

### The anti-addiction model: *Xanthomonas* strains have TAs in a process of being lost

**Figure 5:**
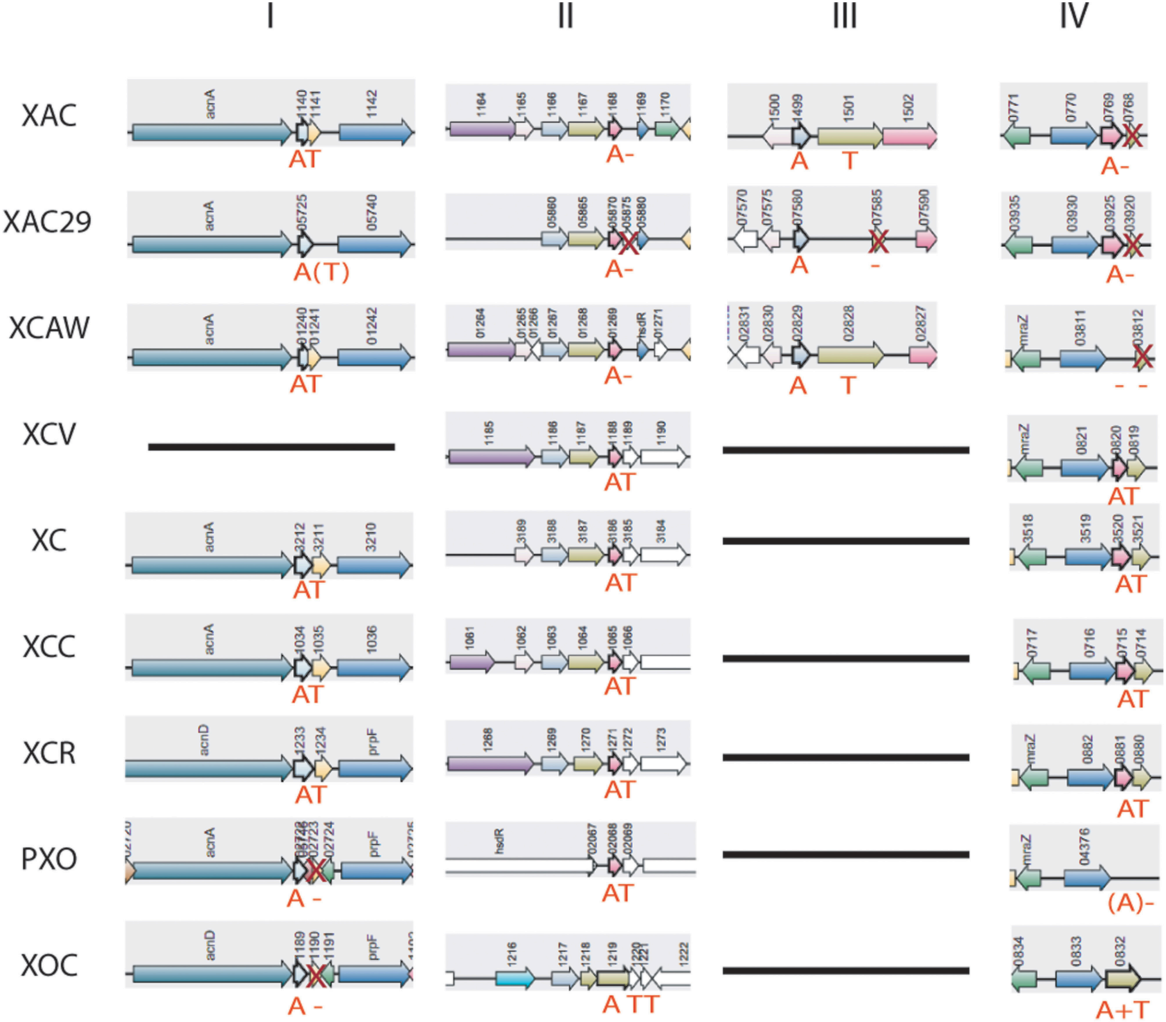
**Toxin genes loss on *Xanthomonas* genomes**. “A,” antitoxin; “T,” toxin; X, toxin gene is present, but without protein domain; A or T in brackets represents ORFs that are not annotated on the genomes, but were predicted by RASTA bioinformatics tool. XAC (*X. citri* 306); XAC29 (*X. citri* Xac29-1); XCAW (*X. citri* Aw); XCV (*X. campestris* pv. *vesicatoria*); XOC (*X. oryzae* pv. *oryzicola*); PXO (*X. oryzae* pv. *oryzae*); XC (*X. campestris* 8004); XCC (*X. campestris* ATTC33913); XCR (*X. campestris* pv. *raphani*); XAL (*X. albilineans*).

Many TA operons were found lacking a complete toxin gene, and therefore we investigated the possible antitoxin maintenance in *Xanthomonas* spp. genomes (Table S2). In some cases, the proteins lacking the toxin domain were present up or downstream of the antitoxins. The examples of these processes are shown in Figure 5, where, in column I, the toxin is absent in both *X. oryzae* strains, columns II, III, and IV show toxin deletions occurring in *X. citri* strains. It is interesting to highlight that the TA system shown in column III is in a genomic region located at the border of a genomic island, which is exclusive to *X. citri*. Even in this case, we can observe the retention of the antitoxin, with the toxin loss having already occurred in *X. axonopodis* 29-1. In addition, the column IV shows an unusual TA structure in *X. oryzae* pv. *oryzicola*—a gene fusion, leading to the production of a putative protein with both toxin and antitoxin domains. A Cdart analysis using this fusion protein showed that this is indeed a very rare phenomenon, and a similar configuration appears only in the Gammaproteobacteria *Spiribacter* sp. (data not shown). A functional fusion of toxin and antitoxin domains has been described very recently (Rocker and Meinhart, 2015), but whether the fusion found in *X. oryzae* is functional remains to be discovered.

**Figure 6:**
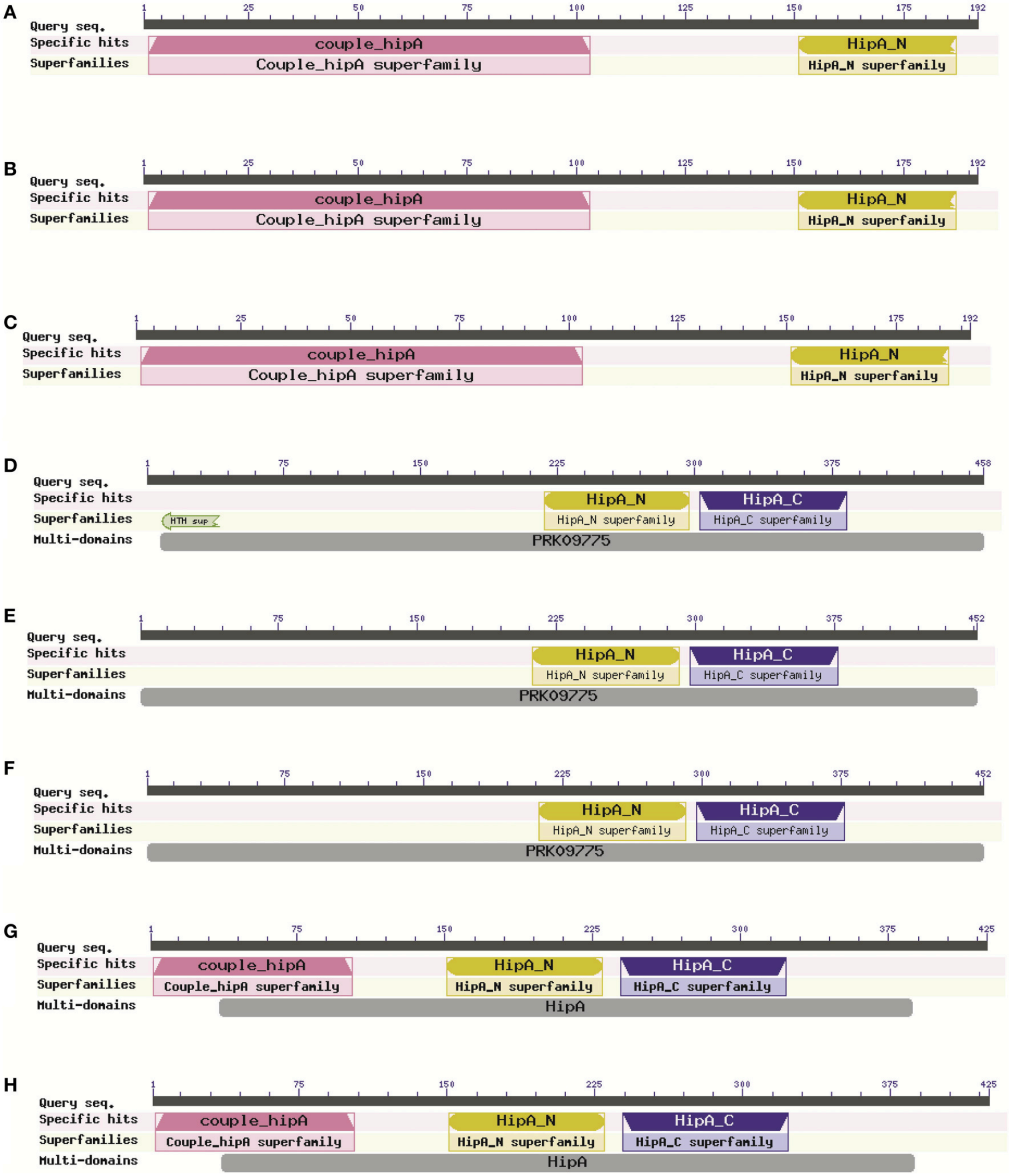
**Different HipA toxin domain configuration found in *Xanthomonas* genomes**. **(A–C)** The C-terminal domain deletion occurrence in Citrus-infecting pathovars (*Xanthomonas citri* pv. *citri* 306); *Xanthomonas axonopodis* Xac29-1; *Xanthomonas citri* subsp. *citri* Aw12869); **(D–F)** Couple_HipA domain deletion in *X. campestris* strains (*Xanthomonas campestris* pv. *campestris* 8004; *Xanthomonas campestris* pv. *campestris* ATTC33913; *Xanthomonas campestris* pv. *raphani* 756C); **(G,H)** Complete domain composition of HipA is found only in *X. oryzae* strains (*Xanthomonas oryzae* pv. *oryzicola* BLS256; *Xanthomonas oryzae* pv. *oryzae* PXO99A).

Similar to the toxin gene loss, some HipA proteins also appear to be lacking of the domains. The most common architecture found among HipA proteins contains three domains: *couple_HipA, HipA_N*, and *HipA_C*; it is a multidomain architecture, known as COG3550. The structure shown by *X. citri* strains is much less common in NCBI (Cdart—data not shown) and seems to be incomplete, lacking the C-terminal domain (Figures 6A–C). Otherwise, *X. campestris* strains had undergone *Couple_HipA* domain loss, which is located at the N-terminal portion of the protein (Figures 6D–F). Interestingly, *X. campestris* HipA is neither predicted by RASTA bioinformatic tool as a possible toxin nor seems to be located in a TA operon, since it is not flanked by any antitoxin gene. These *X. campestris* proteins, therefore, can be considered solitary toxins—a very rare configuration found in *Xanthomonas* spp. (Table S2). But it is important to highlight that its functionality still remains uncertain due to such domain configuration. It is known that both C- and N-terminal domains of HipA are involved in HipB antitoxin ligation and consequent neutralization of its toxic activity (Schumacher et al., 2009). *X. oryzae* strains are unique to show the complete and widespread domain configuration found for HipA toxin (Figures 6G,H).

The loss of the toxin gene (or its toxin domain) was observed in at least seven of the 26 TA systems considered (Table S1—in red). In contrast, a putative antitoxin deletion was found only in one case (XAC29_22294 of *X. citri* 29-1).

### TA systems present only in *X. citri* are differentially modulated by stresses

**Figure 7:**
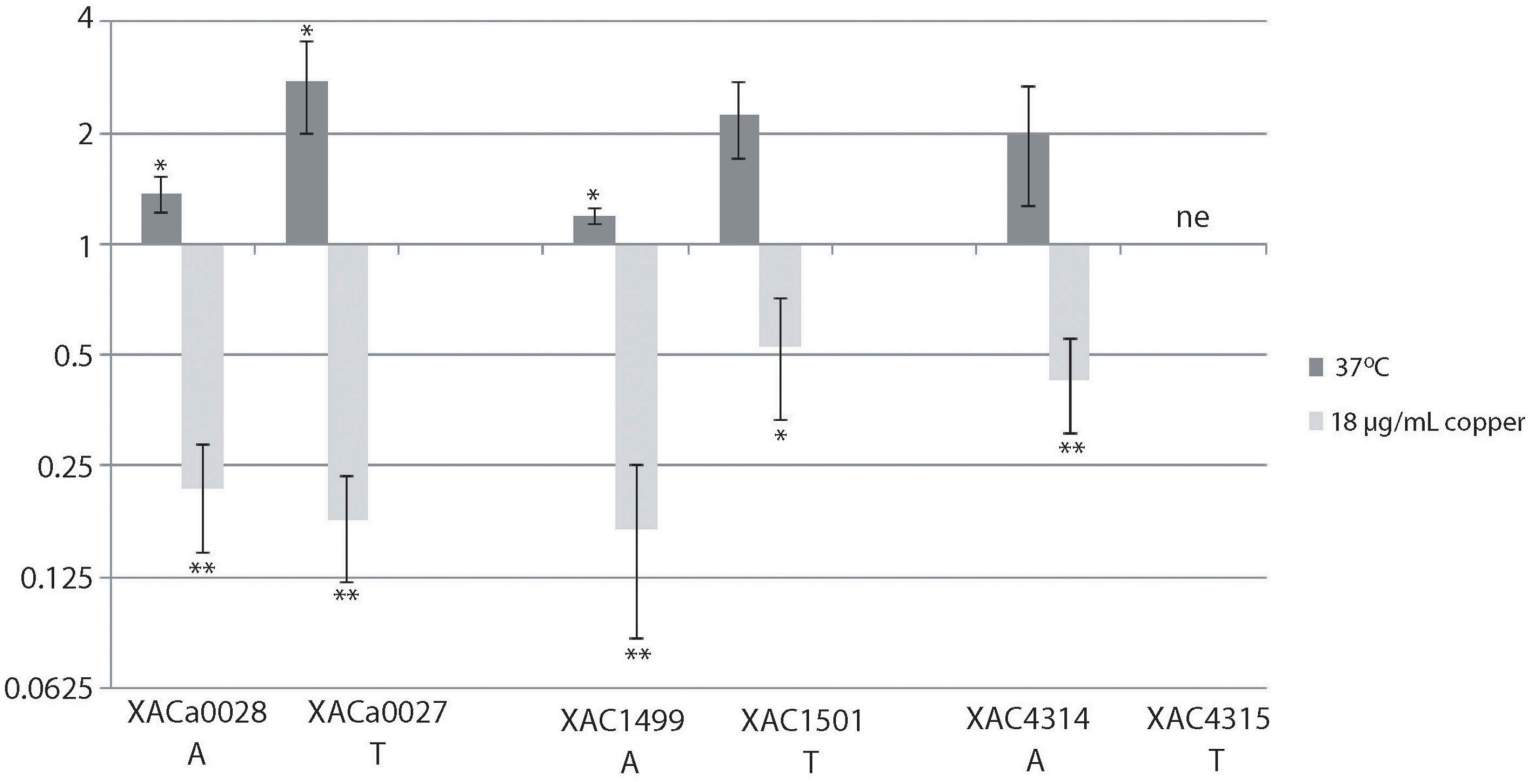
**The relative expression of toxin-antitoxin systems in *X. citri* under stress**. The relative expression of the genes XACa0028, XACa0027, XAC1499, XAC1501, and XAC4314 after temperature or copper stress in comparison to cells maintained at 28°C and without copper. The fold change data are presented in Log_2_. 16S gene expression was used as endogenous control. RT-qPCR reactions were repeated twice and each condition had three biological replicates. XAC4315 was not evaluated (ne). ^*^indicates significant difference *P* ≥ 0.05 and ^**^indicates significant difference *P* ≥ 0.01.

To validate the gene expression of some TA systems, we chose the XACa0027/XACa0028, XAC4314/XAC4315, and XAC1499/XAC1501 TA systems since they are present only in *X. citri*. Using RT-PCR analysis we verified that the expression behavior of these three operons was similar, being induced at high temperature (37°C) and repressed at high copper concentration (18 μg/mL; Figure 7). Even though the trend was similar, XACa0027/XACa0028 showed relatively a higher expression modulation (Figure 7).

## Discussion

Antimicrobial compounds and environmental fluctuations lead to hostile conditions for bacteria. TA systems play an important role in bacterial survival under these conditions as they induce physiological changes such as persister cell formation (Wang and Wood, 2011). In the genus *Xanthomonas*, no such study has ever been done to assess the occurrence of these systems, although they belong to a group of very important phytopathogens that affect many crops worldwide (Vurro et al., 2010). Therefore, we analyzed TA profiles in ten *Xanthomonas* genomes, discriminately selected to cover a representative phylogenic range (Jalan et al., 2013) and spanning different hosts and tissues preferably colonized (Table 1).

The TAs found in *X. citri* strains outnumber those in other *Xanthomonas* genomes. Since the chromosome size in all *Xanthomonas* spp. is very similar (around 5 Mb), except for *X. albilineans* (3.8 MB), the TA prevalence observed in the citrus pathovars cannot be attributed to the divergent chromosomal content. It is known that there is no correlation between the number of TA systems and genome size, cell shape, or phylum; nevertheless the more host-associated the microbe is, the less TA pairs can be found in its genome (Pandey and Gerdes, 2005). Therefore, we hypothesize that the bacterium, *X. albilineans*, shows less TA numbers due to its deep proximity to its host, since this strain is primarily a xylem inhabitant with a reduced/absent life cycle outside the plant (Pieretti et al., 2009). In contrast, other *Xanthomonas* spp. are widely exposed to environmental stressors during their epiphytic survival on the host's living tissues but not in free soil (Graham et al., 2004; Köhl et al., 2011). Regarding the other *Xanthomonas* hosts, citrus pathovars are unique for infecting a perennial crop, as sugarcane, tomato, cruciferous, and rice are all removed from the field after harvest. This fact indicates that citrus-infecting *Xanthomonas* spp. live in the host plant for a long time, and consequently are more exposed to the environmental stresses. However, if this is the reason for TA abundance in *X. citri* needs further investigation. The quantitative effect of many TAs in one cell is *per se* an important feature that needs to be considered. In *E. coli*, for instance, the progressive deletion of 10 RNA-degrading toxins reduced significantly the level of persister induction as they become more TA depleted (Maisonneuve et al., 2011). Thus, it is reasonable to consider that the Citrus-infecting pathovars can somehow benefit from harboring more TAs than other *Xanthomonas* spp.

Distinguishing features were found concerning the TA domains present on the genomes of some strains. For instance, the operon *chpBS* was exclusively found in *X. citri* strains. In *E. coli*, this TA has earlier been described as being responsive to “extracellular death factors” (EDF; Belitsky et al., 2011). These molecules are quorum-sensing peptides supposed to enhance the ribonucleolytic activity of the toxin, inducing programmed cell death. This is the unique toxin present in the studied *Xanthomonas* genomes that apparently has this characteristic. This TA occurrence in *X. citri* opens the possibility that, in the future, EDFs could be used as a control measure for citrus canker, as also proposed for human pathogens such as *E. coli* (Kumar and Engelberg-Kulka, 2014).

In this study, we observed that the gene expression of the three TA systems analyzed by RT-qPCR showed the same trend, where the operons were significantly up and down-regulated by high temperature and copper stress, respectively (Figure 7). The XACa0028/XACa0027 system of *X. citri* is the most responsive one among them. In *E. coli*, temperature stress also induces the expression of a similar toxin, *mazF* (Singh and Jiang, 2015). Interestingly, it has recently been shown that, in *X. citri*, cells are elongated when cultured at 37° (Sumares et al., 2016), and that this feature is related with persister cell formation in *E. coli* (Maisonneuve et al., 2013).

It is intriguing that only one TA system (PIN/SpoVT) was conserved throughout all the genomes analyzed. This system, localized in the proximity of an aconitase gene, might be acting as a stabilizer, since TAs may prevent gene losses in bacterial genomic regions (Szekeres et al., 2007). It was previously shown that, at least in *X. campestris* pv. *vesicatoria*, this TA is co-transcribed in the same operon as the aconitase gene, which is required for virulence by this pathogen (Kirchberg et al., 2012). It is quite interesting that despite the similarity of the antitoxin amino acid sequences among the ten genomes, the toxin sequences show a much higher diversity. Besides, the more distantly the strains are related, the more divergent the sequences are, being the toxin under positive selection, as shown by dN/dS analysis.

The toxin variability observed in the system PIN/SpoVT (Figure 4A) is also present in the HipA toxin (Figure 6). This plasticity can eventually lead to the toxin loss, as indeed detected in many genomes (Table S1—in red). In summary, there is a trend of toxin gene loss in *Xanthomonas* genomes. The toxin gene deletion (or the toxin domain deletion) along with the diverse domain configurations found for HipA could be taken as a snapshot of a possible in-process toxin gene loss occurring during the bacterial genome evolution. This might be happening for those TAs in which the toxic counterpart failed to co-opt for any important cellular function. This deletion has occurred in at least seven TA systems found in *Xanthomonas*, leading us to think that the toxin deletion and antitoxin retention could be a common phenomenon in *Xanthomonas* genomes.

The antitoxin retention after the toxin gene loss may be related to the so called “anti-addiction model” (Saavedra De Bast et al., 2008), in which the chromosomal retention of TAs may have some role in the protection of the prokaryotic cell against an exogenous DNA invasion. According to this model, the cells that harbor TAs are supposed to be protected by not allowing the infection of exogenous DNA (like plasmids) that relies on this system to be stably maintained in the population. However, bacteria also have other tools to fight against these infections, such as CRISPR and restriction-modification systems, but the retention of antitoxins could help the cells to deal with the invasive DNA in some different ways. Another possibility is that the antitoxins retained were co-opted to other functions, e.g., acting in *trans* as a regulator of gene expression. It is already known that TA systems found in bacterial chromosomes have an undeniable involvement in many cellular processes within the cell (Van Melderen and Saavedra De Bast, 2009). It is accepted that many of them are involved in cellular functions like persistence and growth modulation in a wide range of bacterial species (Wang and Wood, 2011; Muranaka et al., 2012; Butt et al., 2014). These findings, however, usually rely on toxin activity as the main contributor, since it can decrease the overall translation process in response to environmental conditions (Christensen et al., 2001; Kolodkin-Gal and Engelberg-Kulka, 2006). One of the first known examples of a regulatory function of an antitoxin is the MqsA of *E. coli*, which is known to regulate cellular functions by directly repressing *rpoS*, the stationary sigma factor (Wang and Wood, 2011). Recently, another antitoxin (DinJ) has also been shown to regulate other cellular functions (Hu et al., 2012). These observations strengthen the importance of these antitoxins in prokaryotic biology. It has also been shown that the antitoxin XAC1499 in *X. citri* 306 is involved in phenotypic characters such as EPS production and cell motility regulation (Li and Wang, 2011). Therefore, it is possible that antitoxins play a much broader role in the bacterial physiology than that conceived earlier.

In summary, this study outlines the intra-genus differences regarding the TA systems in *Xanthomonas*, which seem to be arising due to horizontal gene transfer and toxin gene loss over time. Our results open up new perspectives for the study of this important system that confers advantages to the bacterial genus *Xanthomonas*. It also offers some novel insights for controlling phytopathogen similar to the programmed cell death strategy proposed for human bacterial pathogens (Allocati et al., 2015).

## Author contributions

AS planned and supervised the study. PM performed the analysis and drafted the manuscript. PM, NS, and MT performed the bioinformatics analysis. AS, MT, PM, and MM contributed to the interpretation of the data and provided intellectual input. AS, MT, and PM revised the manuscript. All authors read and approved the final manuscript.

## Funding

This work was supported by research grants from Fundação de Amparo a Pesquisa do Estado de São Paulo and Conselho Nacional de Desenvolvimento Científico e Tecnológico (INCT—Citros 08/57909-2, 573848/08-4 and CNPq—Universal 470475/2013-7). PM is a post-doc fellow from CNPq (164342/2015-0). MM, MT, and AS are recipients of research fellowships from CNPq.

### Conflict of interest statement

The authors declare that the research was conducted in the absence of any commercial or financial relationships that could be construed as a potential conflict of interest.
