# Supplementary material for: Type II Toxin-Antitoxin Distribution and Adaptive Aspects on Xanthomonas Genomes: Focus on Xanthomonas citri
Source: Front Microbiol. 2016 May 10;7:652. doi: 10.3389/fmicb.2016.00652 (PMC4861877; doi:10.3389/fmicb.2016.00652)
Supplement: Supplementary file 4 [file DataSheet1.DOCX]

**Multiple sequence alignment**

**Results Produced with T-COFFEE Version_11.00.8cbe486**

**1 - DOMAIN COG3668 - ParE**

**COG3668 - ParE (1)**

NUCLEOTIDE ALIGNMENTS:

XAC2428|21240774_c2828610-2828320 ATGCCACAAGTAATCTTTGCGCCGGCGGCGATCGGGGACATGCAACGGCT

XAC29_12305|470469929_c2844207-2843917 ATGCCACAAGTAATCTTTGCGCCGGCGGCGATCGGGGACATGCAACGGCT

XCAW_02095|471265562_c2308975-2308685 ATGCCACAAGTAATCTTTGCGCCGGCGGCGATCGGGGACATGCAACGGCT

**************************************************

XAC2428|21240774_c2828610-2828320 GCGCGAATTCCTGAAGCCGAAGAGTCCCGATGCCGCGAGGCGGGCCGGTG

XAC29_12305|470469929_c2844207-2843917 GCGCGAATTCCTGAAGCCGAAGAGTCCCGATGCCGCGAGGCGGGCCGGTG

XCAW_02095|471265562_c2308975-2308685 GCGCGAATTCCTGAAGCCGAAGAGTCCCGATGCCGCGAGGCGGGCCGGTG

**************************************************

XAC2428|21240774_c2828610-2828320 AAGCGATTAGGCGCGGCGTCCAGGCTCTCGGTGCCCACCCTCGAATGGGG

XAC29_12305|470469929_c2844207-2843917 AAGCGATTAGGCGCGGCGTCCAGGCTCTCGGTGCCCACCCTCGAATGGGG

XCAW_02095|471265562_c2308975-2308685 AAGCGATTAGGCGCGGCGTCCAGGCTCTCGGTGCCCACCCTCGAATGGGG

**************************************************

XAC2428|21240774_c2828610-2828320 CGGCTCATAGAAGATCTGCCTGAACAGTATCGGGAGTGGCTTATCGATTT

XAC29_12305|470469929_c2844207-2843917 CGGCTCATAGAAGATCTGCCTGAACAGTATCGGGAGTGGCTTATCGATTT

XCAW_02095|471265562_c2308975-2308685 CGGCTCATAGAAGATCTGCCTGAACAGTATCGGGAGTGGCTTATCGATTT

**************************************************

XAC2428|21240774_c2828610-2828320 TGGGGATAGTGGCTATGTGGCTCGGTACCGCCACGCTGGCGATACCGTCA

XAC29_12305|470469929_c2844207-2843917 TGGGGATAGTGGCTATGTGGCTCGGTACCGCCACGCTGGCGATACCGTCA

XCAW_02095|471265562_c2308975-2308685 TGGGGATAGTGGCTATGTGGCTCGGTACCGCCACGCTGGCGATACCGTCA

**************************************************

XAC2428|21240774_c2828610-2828320 CGATTTTGGCCGTGCGACACCAGAAGGAAGCGGGGTACTGA

XAC29_12305|470469929_c2844207-2843917 CGATTTTGGCCGTGCGACACCAGAAGGAAGCGGGGTACTGA

XCAW_02095|471265562_c2308975-2308685 CGATTTTGGCCGTGCGACACCAGAAGGAAGCGGGGTACTGA

*****************************************

PROTEIN ALIGNMENTS:

XAC2428|AAM37280.1|conserved MPQVIFAPAAIGDMQRLREFLKPKSPDAARRAGEAIRRGVQALGAHPRMG

XAC29_12305|AGH77914.1|hypothetical MPQVIFAPAAIGDMQRLREFLKPKSPDAARRAGEAIRRGVQALGAHPRMG

XCAW_02095|AGI07884.1|Hypothetical MPQVIFAPAAIGDMQRLREFLKPKSPDAARRAGEAIRRGVQALGAHPRMG

**************************************************

XAC2428|AAM37280.1|conserved RLIEDLPEQYREWLIDFGDSGYVARYRHAGDTVTILAVRHQKEAGY

XAC29_12305|AGH77914.1|hypothetical RLIEDLPEQYREWLIDFGDSGYVARYRHAGDTVTILAVRHQKEAGY

XCAW_02095|AGI07884.1|Hypothetical RLIEDLPEQYREWLIDFGDSGYVARYRHAGDTVTILAVRHQKEAGY

**********************************************

**COG3668 - ParE (2)**

NUCLEOTIDE ALIGNMENTS:

XCC1035|21229478_1184213-1184530 ATGAAGCCGGCGCGCTGGCGCCCCTTGGCGCTGCGCGATGTCGACGAGGC

XAC1141|21240774_1292060-1292236 GTGAAGCCTGCGCACTGGCGTCCGTTGGCGCTGCGCGATGTCGAGGCCGC

XC_3211|66766352_c3856336-3856019 ATGAAGCCGGCGCGCTGGCGCCCCTTGGCGCTGCGCGATGTCGACGAGGC

XCR_1234|384425691_1248630-1248923 TTGGCG------------------------CTGCGCGATGTCGACGAGGC

XCAW_01241|471265562_1379261-1379437 GTGAAGCCTGCGCACTGGCGTCCGTTGGCGCTGCGCGATGTCGAGGCCGC

** * ************** * **

XCC1035|21229478_1184213-1184530 AGCCGCCTGGTACGGCGCCGAGGGCGGGCTTGCGCTGGAATTGGCCTTTA

XAC1141|21240774_1292060-1292236 AGCCGCGTGGTACGGCGAACAGGCGGGGCTGGAGGTCGAACTGGCGTTTG

XC_3211|66766352_c3856336-3856019 AGCCGCCTGGTACGGCGCCGAGGGCGGGCTTGCGCTGGAATTGGCCTTTA

XCR_1234|384425691_1248630-1248923 CGCCGCCTGGTACGGCGCCGAGGGCGGGCTTGCGCTGGAATTGGCCTTTA

XCAW_01241|471265562_1379261-1379437 AGCCGCGTGGTACGGCGAACAGGCGGGGCTGGAGGTCGAACTGGCGTTTG

***** ********** *** ***** * * * *** **** ***

XCC1035|21229478_1184213-1184530 CCAAAGCACTGGAGTCGGCGGTGACCACGCTCATGCGCAATCCGGCGGCC

XAC1141|21240774_1292060-1292236 TCGACGCACTGGTATCGGCGGTCGACATGCTCGTGCAGC-----------

XC_3211|66766352_c3856336-3856019 CCAAAGCACTGGAGTCGGCGGTGACCACGCTCATGCGCAATCCGGCGGCC

XCR_1234|384425691_1248630-1248923 CCAAAGCACTGGAGTCGGCGGTGACCACGCTCATGCGCAATCCGGCGGCC

XCAW_01241|471265562_1379261-1379437 TCGACGCACTGGTATCGGCGGTCGACATGCTCGTGCAGC-----------

* * ******* ******** ** **** ***

XCC1035|21229478_1184213-1184530 GGTTCTAGCCGCCACGCGGTGGTGCTGAAGCTGCCGCAGATTCGTGTCTG

XAC1141|21240774_1292060-1292236 -ATCCTAGC--------GCT------------------------------

XC_3211|66766352_c3856336-3856019 GGTTCTAGCCGCCACGCGGTGGTGCTGAAGCTGCCGCAGATTCGTGTCTG

XCR_1234|384425691_1248630-1248923 GGTTCTAGCCGCCACGCGGTGGTGCTGAAGCTGCCGCAGATTCATGTCTG

XCAW_01241|471265562_1379261-1379437 -ATCCTAGC--------GCT------------------------------

* ***** * *

XCC1035|21229478_1184213-1184530 GCCGCTCAAGCGGTTTCCCTATCTGCTGTTCTACAACGAGCGCGCAACCG

XAC1141|21240774_1292060-1292236 --------------------------------------------------

XC_3211|66766352_c3856336-3856019 GCCGCTCAAGCGGTTTCCCTATCTGCTGTTCTACAACGAGCGCGCAACCG

XCR_1234|384425691_1248630-1248923 GCCGCTCAAGCGGTTTCCCCATCTGCTGTTTTACAACGAGCGCGCAACCG

XCAW_01241|471265562_1379261-1379437 --------------------------------------------------

XCC1035|21229478_1184213-1184530 ACATCGTCATCTGGCGTGTCCTGCACATGCAACGCGATATTCCTGCGTGG

XAC1141|21240774_1292060-1292236 -------------------------GGTGCAAGTCGCTATG----CGGTG

XC_3211|66766352_c3856336-3856019 ACATCGTCATCTGGCGTGTCCTGCACATGCAACGCGATATTCCTGCGTGG

XCR_1234|384425691_1248630-1248923 ACATCGTCATCTGGCGTGTCCTGCACATGCAACGCGATATTCCTGCGTGG

XCAW_01241|471265562_1379261-1379437 -------------------------GGTGCAAGTCGCTATG----CGGTG

***** ** *** ** *

XCC1035|21229478_1184213-1184530 ATGAGCGCACATCCGTAA

XAC1141|21240774_1292060-1292236 AT------------GTGA

XC_3211|66766352_c3856336-3856019 ATGAGCGCACATCCGTAA

XCR_1234|384425691_1248630-1248923 ATGAGCGCACATCCGTAA

XCAW_01241|471265562_1379261-1379437 AT------------GTGA

** ** *

PROTEIN ALIGNMENTS:

XCC1035|AAM40334.1|conserved MKPARWRPLALRDVDEAAAWYGAEGGLALELAFTKALESAVTTLMRNPAA

XAC1141|AAM36013.1|conserved MKPAHWRPLALRDVEAAAAWYGEQAGLEVELAFVDALVSAVDMLVQHPSA

XC_3211||AAY50255.1|conserved MKPARWRPLALRDVDEAAAWYGAEGGLALELAFTKALESAVTTLMRNPAA

XCR_1234|AEL06135.1|plasmid --------MALRDVDEAAAWYGAEGGLALELAFTKALESAVTTLMRNPAA

XCAW_01241|AGI07043.1|Hypothetical MKPAHWRPLALRDVEAAAAWYGEQAGLEVELAFVDALVSAVDMLVQHPSA

:*****: ****** :.** :****..** *** *:::*:*

XCC1035|AAM40334.1|conserved GSSRHAVVLKLPQIRVWPLKRFPYLLFYNERATDIVIWRVLHMQRDIPAW

XAC1141|AAM36013.1|conserved GASRYAVM------------------------------------------

XC_3211|AAY50255.1|conserved GSSRHAVVLKLPQIRVWPLKRFPYLLFYNERATDIVIWRVLHMQRDIPAW

XCR_1234|AEL06135.1|plasmid GSSRHAVVLKLPQIHVWPLKRFPHLLFYNERATDIVIWRVLHMQRDIPAW

XCAW_01241|AGI07043.1|Hypothetical GASRYAVM------------------------------------------

*:**:**:

XCC1035|AAM40334.1|conserved MSAHP

XAC1141|AAM36013.1|conserved -----

XC_3211||AAY50255.1|conserved MSAHP

XCR_1234|AEL06135.1|plasmid MSAHP

XCAW_01241|AGI07043.1|Hypothetical -----

**2 - DOMAIN COG3905 - Transcriptional regulator**

NUCLEOTIDE ALIGNMENTS:

XAC2429|21240774_c2828876-2828598 ATGGTTACGGCAACTTCCATCAAGCTTGATGATGAATTGAAAGGGCGGGT

XCAW_02096|471265562_c2309241-2308963 ATGGTTACGGCAACTTCCATCAAGCTTGATGATGAATTGAAAGGGCGGGT

XAC29_12310|470469929_c2844473-2844195 ATGGTTACGGCAACTTCCATCAAGCTTGATGATGAATTGAAAGGGCGGGT

**************************************************

XAC2429|21240774_c2828876-2828598 TCAGCACTTGGCCGAGGCGCGCCGGCGTACCCCGCATTGGATCATGCGCG

XCAW_02096|471265562_c2309241-2308963 TCAGCACTTGGCCGAGGCGCGCCGGCGTACCCCGCATTGGATCATGCGCG

XAC29_12310|470469929_c2844473-2844195 TCAGCACTTGGCCGAGGCGCGCCGGCGTACCCCGCATTGGATCATGCGCG

**************************************************

XAC2429|21240774_c2828876-2828598 AAGCCATTGAGCAGTACGTCGAGCGCGAGGAAAAGCGTGAGGCGTTGAAC

XCAW_02096|471265562_c2309241-2308963 AAGCCATTGAGCAGTACGTCGAGCGCGAGGAAAAGCGTGAGGCGTTGAAC

XAC29_12310|470469929_c2844473-2844195 AAGCCATTGAGCAGTACGTCGAGCGCGAGGAAAAGCGTGAGGCGTTGAAC

**************************************************

XAC2429|21240774_c2828876-2828598 CGGGACACGCTCAAGGCATGGGATGAATTTCAGGCGACGGGCCTGCACGT

XCAW_02096|471265562_c2309241-2308963 CGCGACACGCTCAAGGCATGGGATGAATTTCAGGCGACGGGCCTGCACGT

XAC29_12310|470469929_c2844473-2844195 CGGGACACGCTCAAGGCATGGGATGAATTTCAGGCGACGGGCCTGCACGT

** ***********************************************

XAC2429|21240774_c2828876-2828598 GACCGCTGAGGAAGTGGACAAGTGGCTCGCGAGCTGGGGAACAGACGACG

XCAW_02096|471265562_c2309241-2308963 GACCGCTGAGGAAGTGGACAAGTGGCTCGCGAGCTGGGGAACAGACGACG

XAC29_12310|470469929_c2844473-2844195 GACCGCTGAGGAAGTGGACAAGTGGCTCGCGAGCTGGGGAACAGACGACG

**************************************************

XAC2429|21240774_c2828876-2828598 AACTGCCTCCGCCAGAATGCCACAAGTAA

XCAW_02096|471265562_c2309241-2308963 AACTGCCTCCGCCAGAATGCCACAAGTAA

XAC29_12310|470469929_c2844473-2844195 AACTGCCTCCGCCAGAATGCCACAAGTAA

*****************************

PROTEIN ALIGNMENTS:

XAC2429|AAM37280.1|conserved MVTATSIKLDDELKGRVQHLAEARRRTPHWIMREAIEQYVEREEKREALN

XCAW_02096|AGI07885.1|transcriptional MVTATSIKLDDELKGRVQHLAEARRRTPHWIMREAIEQYVEREEKREALN

XAC29_12310|AGH77915.1|hypothetical MVTATSIKLDDELKGRVQHLAEARRRTPHWIMREAIEQYVEREEKREALN

**************************************************

XAC2429|AAM37280.1|conserved RDTLKAWDEFQATGLHVTAEEVDKWLASWGTDDELPPPECHK

XCAW_02096|AGI07885.1|transcriptional RDTLKAWDEFQATGLHVTAEEVDKWLASWGTDDELPPPECHK

XAC29_12310|AGH77915.1|hypothetical RDTLKAWDEFQATGLHVTAEEVDKWLASWGTDDELPPPECHK

******************************************

**3 - DOMAIN pfam05016 - plasm. stab. prot. RelE/ParE**

**pfam05016 - plasm. stab. prot. RelE/ParE (1)**

NUCLEOTIDE ALIGNMENTS:

XAC0081|21240774_99706-100005 ATGGCTGAAATAATCTGGTCGGTGCCGGCATTGGCTG-ATCTGGACGCAA

XAC29_00410|470469929_99739-100038 ATGGCTGAAATAATCTGGTCGGTGCCGGCATTGGCTG-ATCTGGACGCAA

XCV0058|78045556_70082-70363 GTGC---------------------CGGCATTGGCTG-ATCTGGACGCAA

PXO_03335|188574270_c149225-148995 ATGCG--CACCGAACG------TGTCACCAC--GCTCAAGCGTCAGGCGA

XOC_0152|384417079_156365-156736 TTGG----------------------------------------AAGCGA

** * ** *

XAC0081|21240774_99706-100005 TTGCCGACTACATCGCAATCGATAACGCACCCGCTGCAGCG---GCGCTG

XAC29_00410|470469929_99739-100038 TTGCCGACTACATCGCAATCGATAACGCACCCGCTGCAGCG---GCGCTG

XCV0058|78045556_70082-70363 TTGCGGACTACATCGCAATCGATAACGCACCCGCTGCAGCGGCGGCGCTG

PXO_03335|188574270_c149225-148995 CCG--AACTTC-TTGCA---------------GCTACAGA------GCGC

XOC_0152|384417079_156365-156736 CTGCCGACGACATCACAATTGACAACGTGCCCGTTGCAGCG---GCGCTG

* ** * * ** * * *** **

XAC0081|21240774_99706-100005 GTGAAACGGGTGTTC--GGGCACGTTGAGCAATTGATCGAACACCCGGAC

XAC29_00410|470469929_99739-100038 GTGAAACGGGTGTTC--GGGCACGTTGAGCAATTGATCGAACACCCGGAC

XCV0058|78045556_70082-70363 GTCAAATGGGTGTTG--CCGCAGGTTGAGCAATTAATTGAACACCCCGAC

PXO_03335|188574270_c149225-148995 GACAAGGAGCCAATCCTGATCACACAGCACGGTTTACCCAGCGCCTATCT

XOC_0152|384417079_156365-156736 GTGAAACGGGTGTTC--GCGCACGTTGAGCAGTTGATCGAACCCCCGGAC

* ** * * ** * * ** * * * **

XAC0081|21240774_99706-100005 AGCGGAAGCCGACCACAGGAACTCAAGCGCTCGCGCTATCGCCAGATTGT

XAC29_00410|470469929_99739-100038 AGCGGAAGCCGACCACAGGAACTCAAGCGCTCGCGCTATCGCCAGATTGT

XCV0058|78045556_70082-70363 AGCGGAAGCCGGCCACAGGAACTCAAGCGCTCGCGCTATCGCCAGATTGT

PXO_03335|188574270_c149225-148995 GAGGGATGTTG-CC--AGCTA--TGAGCGCATGCAGCAGCGC---A-TGG

XOC_0152|384417079_156365-156736 AGTGGAAGCCGGCTACAGGAACTCAAGCGCTCGCGCTATCGCCAGATTGT

*** * * * ** * ***** ** * *** * **

XAC0081|21240774_99706-100005 CGAACCACCTTGCCGCGTGTTCTACCGCGTGGATGGGCAGCGCATCGTGG

XAC29_00410|470469929_99739-100038 CGAACCACCTTGCCGCGTGTTCTACCGCGTGGATGGGCAGCGCATCGTGG

XCV0058|78045556_70082-70363 CGAACCACCTTGCCGCGTGTTCTACCGCGTGGATGGGCAGCACATTGTGC

PXO_03335|188574270_c149225-148995 CGATCCTC---GAAG--GAATCG--CCCGTGGAG-------AAATGGCAG

XOC_0152|384417079_156365-156736 CGAACCACCTTGCCGCGTGATCTACCGCGTGGATGGGCAGCGCATCGTGG

*** ** * * * ** * ****** ** *

XAC0081|21240774_99706-100005 TGGTGCACGTCATGCGGTCGGAGCGCGCGCT-TCGCG-------------

XAC29_00410|470469929_99739-100038 TGGTGCACGTCATGCGGTCGGAGCGCGCGCT-TCGCG-------------

XCV0058|78045556_70082-70363 TGGTGCACGTCATGCGGTGGGAGCGCGCGCT-TCGCA-------------

PXO_03335|188574270_c149225-148995 TAGCGCAAG-------GC-----CGCACGCTGACCCA-------------

XOC_0152|384417079_156365-156736 TGGTGCACGTGTTGCGCTCGGAGCGCGCGCT-TCGCAAGTGCCGCATTTC

* * *** * *** **** * *

XAC0081|21240774_99706-100005 -----GGAA------------------------CCG-------------T

XAC29_00410|470469929_99739-100038 -----GGAA------------------------CCG-------------T

XCV0058|78045556_70082-70363 -----GGAA------------------------CCG-------------T

PXO_03335|188574270_c149225-148995 -----TGAA------------------------CAG-------------G

XOC_0152|384417079_156365-156736 ACGCTGGAAGCGGCTAACAAAACGACGGCGCAGCCGCCCGGTGGGCGCGG

*** * *

XAC0081|21240774_99706-100005 CT---------------------------T--------------------

XAC29_00410|470469929_99739-100038 CT---------------------------T--------------------

XCV0058|78045556_70082-70363 CT---------------------------T--------------------

PXO_03335|188574270_c149225-148995 C-------------------------------------------------

XOC_0152|384417079_156365-156736 CTTGTGCCCCATGCTGCATGGACCAGGCCTCCACTCCGCTTGTGAATGCG

*

XAC0081|21240774_99706-100005 ----TCAC-----GCTAA

XAC29_00410|470469929_99739-100038 ----TCAC-----GCTAA

XCV0058|78045556_70082-70363 ----TCAC-----GCTAA

PXO_03335|188574270_c149225-148995 --------------ATAG

XOC_0152|384417079_156365-156736 CTGTCCACACCCAGCTGA

*

PROTEIN ALIGNMENTS:

XAC0081|AAM34973.1|conserved MAEIIWSVPALADLDAIADYIAIDNAPAA-AALVKRVFGHVEQLIEHPDS

XAC29_00410|EMBOSS_001_1|plasmid MAEIIWSVPALADLDAIADYIAIDNAPAA-AALVKRVFGHVEQLIEHPDS

XCV0058|CAJ21689.1|conserved M-------PALADLDAIADYIAIDNAPAAAAALVKWVLPQVEQLIEHPDS

PXO_03335|ACD56821.1|antitoxin MRT----------------------------ERVTTLKRQATELLAATER

XOC_0152|AEQ94406.1|plasmid -------------MEATADDITIDNVPVA-AALVKRVFAHVEQLIEPPDS

*. : :. :*: .:

XAC0081|AAM34973.1|conserved GSRPQELKRSRYRQIVEPPCRVFYRVDGQRIVVVHVMRSERALRGNRLS-

XAC29_00410|EMBOSS_001_1|plasmid GSRPQELKRSRYRQIVEPPCRVFYRVDGQRIVVVHVMRSERALRGNRLS-

XCV0058|CAJ21689.1|conserved GSRPQELKRSRYRQIVEPPCRVFYRVDGQHIVLVHVMRWERALRRNRLS-

PXO_03335|ACD56821.1|antitoxin DKEPILITQHGLPSAYLRDVASYERMQQRMAILEGIARGEMAVAQGRTLT

XOC_0152|AEQ94406.1|plasmid GSRLQELKRSRYRQIVEPPCRVIYRVDGQRIVVVHVLRSERALRKCRIS-

... :.: . *:: : :: : * * *: *

XAC0081|AAM34973.1|conserved R-------------------------------------

XAC29_00410|EMBOSS_001_1|plasmid R-------------------------------------

XCV0058|CAJ21689.1|conserved R-------------------------------------

PXO_03335|ACD56821.1|antitoxin H-E---------------------QA------------

XOC_0152|AEQ94406.1|plasmid RWKRLTKRRRSRPVGAACAPCCMDQASTPLVNALSTPS

:

**pfam05016 - plasm. stab. prot. RelE/ParE (2)**

NUCLEOTIDE ALIGNMENTS:

XCC4179|21229478_4985218-4986339 GTGTTGATGTTGCGGCGTGACTGGACCGTCCCGGCCGCCACGCAGCTCGC

XAC4313|21240774_5098629-5099735 ATGT------TACGGCGTGACTGGACCGTCCCGGCCGCCACGCAGCTTGC

XC_4266|66766352_5059416-5060537 GTGTTGATGTTGCGGCGTGACTGGACCGTCCCGGCCGCCACGCAGCTCGC

XCV4415|78045556_5090439-5091518 ATGT------TACGGCGTGACTGGACCGTCCCGGCCGCCACGCAGCTTGC

PXO_03524|188574270_5205512-5206558 GTG-----------------------------------------------

XCR_4519|384425691_4850609-4851724 ATGT------TGCGGCGTGACTGGACCGTCCCGGCCGCCACGCAGCTTGC

XOC_0202|384417079_c192798-191686 ATGT------TACGGCGTGACTGGACCGTCCCGGCCGCCACGCAGCTTGC

XAC29_21730|470469929_5078310-5079416 ATGT------TACGGCGTGACTGGACCGTCCCGGCCGCCACGCAGCTTGC

XCAW_04682|471265562_5244573-5245679 ATGT------TACGGCGTGACTGGACCGTCCCGGCCGCCACGCAGCTTGC

**

XCC4179|21229478_4985218-4986339 CCACGCCCAGGATCACTACCACGCGCTCAACCCCACCGCGGCCGCGGCGA

XAC4313|21240774_5098629-5099735 ACACGCCCAGGATCACTACCACGCGCTCAACCCGACCGCCGCTGCCGCGA

XC_4266|66766352_5059416-5060537 CCACGCCCAGGATCACTACCACGCGCTCAACCCCACCGCGGCCGCGGCGA

XCV4415|78045556_5090439-5091518 ACACGCCCAGGATCACTACCACGCGCTCAACCCGACCGCCGCTGCCGCGA

PXO_03524|188574270_5205512-5206558 -------------------------CTCAACCCGACCGCCGCTGCCGCGA

XCR_4519|384425691_4850609-4851724 CCACGCCCAGGATCACTACCACGCGCTCAACCCCACCGCGGCCGCGGCGA

XOC_0202|384417079_c192798-191686 ACACGCCCAGGATCATTACCACGCGCTCAACCCGACCGCCGCTGCCGCGA

XAC29_21730|470469929_5078310-5079416 ACACGCCCAGGATCACTACCACGCGCTCAACCCGACCGCCGCTGCCGCGA

XCAW_04682|471265562_5244573-5245679 ACACGCCCAGGATCACTACCACGCGCTCAACCCGACCGCCGCTGCCGCGA

******** ***** ** ** ****

XCC4179|21229478_4985218-4986339 TGGCCCGGCAGGTGCGCGAAGCCACCCGCACCCTGGCCGAGCAACCCGGC

XAC4313|21240774_5098629-5099735 TGGCGCGGCAGGTGCTGGAAGCCACCCGCACTCTGGCCGAGCAACCAGGC

XC_4266|66766352_5059416-5060537 TGGCCCGGCAGGTGCGCGAAGCCACCCGCACCCTGGCCGAGCAACCCGGC

XCV4415|78045556_5090439-5091518 TGGCGCGGCAGGTGCTGGAAGCCACCCGCACCCTGGCCGAGCAACCCGGC

PXO_03524|188574270_5205512-5206558 TGGCGCGGCAGGTGCTGGAAGCCACCCGCGCCCTGGCCGAGCAACCCGGC

XCR_4519|384425691_4850609-4851724 TGGCCCGGCAGGTGCGCGAAGCCACCCGCACCCTGGCCGAGCAACCCGGC

XOC_0202|384417079_c192798-191686 TGGCGCGGCAGGTGCTGGAAGCCACCCGCACCCTGGCCGAGCAACCCGGC

XAC29_21730|470469929_5078310-5079416 TGGCGCGGCAGGTGCTGGAAGCCACCCGCACTCTGGCCGAGCAACCAGGC

XCAW_04682|471265562_5244573-5245679 TGGCGCGGCAGGTGCTGGAAGCCACCCGCACTCTGGCCGAGCAACCAGGC

**** ********** ************ * ************** ***

XCC4179|21229478_4985218-4986339 CGTGGCCGTGCCGGCCGGGTTGCAGGTACCCGCGAATGGACGGTGAAGCA

XAC4313|21240774_5098629-5099735 CGCGGCCGCGTTGGCCGCGTGCCCGGCACCCGCGAATGGGTGGTCAAGCA

XC_4266|66766352_5059416-5060537 CGTGGCCGTGCCGGCCGGGTTGCAGGTACCCGCGAATGGACGGTGAAGCA

XCV4415|78045556_5090439-5091518 CGTGGCCGCGTTGGCCGCGCGCCCGGCACCCGCGAATGGGTGGTCAAGCA

PXO_03524|188574270_5205512-5206558 CGTGGCCGCGCTGGCCGCGTGCCCGGCACCCGCGAATGGGTGGTCAAGCA

XCR_4519|384425691_4850609-4851724 CGTGGCCGTGCCGGCCGGGTTGCAGGTACCCGCGAATGGACGGTGAAGCA

XOC_0202|384417079_c192798-191686 CGTGGCCGCGCTGGCCGCGTGCCCGGCACCCGCGAATGGGTGGTCAAGCA

XAC29_21730|470469929_5078310-5079416 CGCGGCCGCGTTGGCCGCGTGCCCGGCACCCGCGAATGGGTGGTCAAGCA

XCAW_04682|471265562_5244573-5245679 CGCGGCCGCGTTGGCCGCGTGCCCGGCACCCGCGAATGGGTGGTCAAGCA

** ***** * ***** * * ** ************ *** *****

XCC4179|21229478_4985218-4986339 CACCCCGTACGTGCTGGTGTACCGCGTGCGCGACGACGCCCTGCAGCTGC

XAC4313|21240774_5098629-5099735 GACCCCGTATGTGTTGGTCTACCGCGTGCGTGACGGCGCCCTGCAGGTGT

XC_4266|66766352_5059416-5060537 CACCCCGTACGTGCTGGTGTACCGCGTGCGCGACGACGCCCTGCAGCTGC

XCV4415|78045556_5090439-5091518 GACCCCGTACGTGCTGGTCTACCGCGTGCGCGAGGGCGCCCTGCAGGTGC

PXO_03524|188574270_5205512-5206558 AACCCCGTATGTGCTGGTCTACCGCGTGCGCGACGGCGCCCTGCAGGTGT

XCR_4519|384425691_4850609-4851724 CACCCCGTACGTGCTGGTGTACCGCGTGCGCGACGACGCCCTGCAGCTGC

XOC_0202|384417079_c192798-191686 AACCCCGTATGTGCTGGTCTACCGCGTGCGCGACGGCGCCCTGCAGGTGT

XAC29_21730|470469929_5078310-5079416 GACCCCGTATGTGTTGGTCTACCGCGTGCGTGACGGCGCCCTGCAGGTGT

XCAW_04682|471265562_5244573-5245679 GACCCCGTATGTGTTGGTCTACCGCGTGCGTGACGGCGCCCTGCAGGTGT

******** *** **** *********** ** * ********** **

XCC4179|21229478_4985218-4986339 TGCATGTGCGCCTGGAGACCCAGGACTGGCTGCCGCGCACCGAGCCGGTG

XAC4313|21240774_5098629-5099735 TGCACGTCCATCTGGATGCCAGCGACTGGCTGCCACGCACCGAACCGCTG

XC_4266|66766352_5059416-5060537 TGCATGTGCGCCTGGAGACCCAGGACTGGCTGCCGCGCACCGAGCCGGTG

XCV4415|78045556_5090439-5091518 TGCACGTCCATCTGGATGCCAGCGACTGGCTGCCCCGCACCGAGCCGCTG

PXO_03524|188574270_5205512-5206558 TGCACGTCCATCTGGATGCCAGCGACTGGCTGCCACGGACCGAGCCGCTG

XCR_4519|384425691_4850609-4851724 TGCATGTGCGGCTGGAGACCCAGGACTGGCTGCCGCGCACCGAGCCGGTG

XOC_0202|384417079_c192798-191686 TGCACGTCCATCTGGATGCCAGCGACTGGCTGCCACGGACCGAGCCGCTG

XAC29_21730|470469929_5078310-5079416 TGCACGTCCATCTGGATGCCAGCGACTGGCTGCCACGCACCGAACCGCTG

XCAW_04682|471265562_5244573-5245679 TGCACGTCCATCTGGATGCCAGCGACTGGCTGCCACGCACCGAACCGCTG

**** ** * ***** ** *********** ** ***** *** **

XCC4179|21229478_4985218-4986339 ATCGAGCGCATCGAACCGTGGATTGCCGCGCTGATCAGCGCGCTGCTGCA

XAC4313|21240774_5098629-5099735 ATCGAACGCATCGAACCGTGGATTGCCGCGCTGATCAGCGCGCTGCTGCA

XC_4266|66766352_5059416-5060537 ATCGAGCGCATCGAACCGTGGATTGCCGCGCTGATCAGCGCGCTGCTGCA

XCV4415|78045556_5090439-5091518 ATCGAACGCATCGAACCGTGGATTGCCGCGCTGATCAGCGCGCTGCTGCA

PXO_03524|188574270_5205512-5206558 ATCGAACGCATCGAACCGTGGATTGCCGCGCTGATCAGCGCGTTGTTGCA

XCR_4519|384425691_4850609-4851724 ATCGAGCGCATCGAACCGTGGATTGCCGCGCTGATCAGCGCGCTGCTGCA

XOC_0202|384417079_c192798-191686 ATCGAACGCATCGAACCGTGGATTGCCGCGCTGATCAGCGCGTTGTTGCA

XAC29_21730|470469929_5078310-5079416 ATCGAACGCATCGAACCGTGGATTGCCGCGCTGATCAGCGCGCTGCTGCA

XCAW_04682|471265562_5244573-5245679 ATCGAACGCATCGAACCGTGGATTGCCGCGCTGATCAGCGCGCTGCTGCA

***** ************************************ ** ****

XCC4179|21229478_4985218-4986339 TGTGCTGATGCTGCTGATCCTGTTGTCGGCCTCCACGCCCAGCATGACCC

XAC4313|21240774_5098629-5099735 CGTGCTGATGTTGCTGATCCTGCTGTCGGCCTCCACCCCCACCATGACCC

XC_4266|66766352_5059416-5060537 TGTGCTGATGCTGCTGATCCTGTTGTCGGCCTCCACGCCCAGCATGACCC

XCV4415|78045556_5090439-5091518 CGTGCTGATGTTGCTGATCCTGCTGTCGGCCTCCACCCCCACCATGACCC

PXO_03524|188574270_5205512-5206558 CGTGCTGATGTTGCTGATCCTGCTGTCGGCCTCCACGCCCACCATGACCC

XCR_4519|384425691_4850609-4851724 TGTGCTGATGCTGCTGATCCTGTTGTCGGCCTCCACGCCCAGCATGACCC

XOC_0202|384417079_c192798-191686 CGTGCTGATGTTGCTGATCCTGCTGTCGGCCTCCACGCCCACCATGACCC

XAC29_21730|470469929_5078310-5079416 CGTGCTGATGTTGCTGATCCTGCTGTCGGCCTCCACCCCCACCATGACCC

XCAW_04682|471265562_5244573-5245679 CGTGCTGATGTTGCTGATCCTGCTGTCGGCCTCCACCCCCACCATGACCC

********* *********** ************* **** ********

XCC4179|21229478_4985218-4986339 CGCCGCAGGGCTCGGCCAGCGGTGGCCGCACCAAGGTGGACTTCGTCGGC

XAC4313|21240774_5098629-5099735 CGCCGCAGGGCTCGGCCAGCGGTGGACGCATCAAGGTGGATTTCGTCGGC

XC_4266|66766352_5059416-5060537 CGCCGCAGGGCTCGGCCAGCGGTGGCCGCACCAAGGTGGACTTCGTCGGC

XCV4415|78045556_5090439-5091518 CGCCGCAGGGCTCGGCCAGCGGTGGACGCATCAAGGTGGATTTTGTCGGC

PXO_03524|188574270_5205512-5206558 CGCCGCAAGGCTCGGCCAGCGGTGGACGCATCAAGGTGGATTTCGTCGGC

XCR_4519|384425691_4850609-4851724 CGCCGCAGGGCTCGGCCAGCGGTGGCCGCACCAAGGTGGACTTCGTCGGC

XOC_0202|384417079_c192798-191686 CGCCGCAAGGCTCGGCCAGCGGTGGACGCATCAAGGTGGATTTCGTCGGC

XAC29_21730|470469929_5078310-5079416 CGCCGCAGGGCTCGGCCAGCGGTGGACGCATCAAGGTGGATTTCGTCGGC

XCAW_04682|471265562_5244573-5245679 CGCCGCAGGGCTCGGCCAGCGGTGGACGCATCAAGGTGGATTTCGTCGGC

******* ***************** **** ********* ** ******

XCC4179|21229478_4985218-4986339 GACACCTCCCAGCCCGATCAACCGGTGCCCTCGCCCACCCCGGTACCGCC

XAC4313|21240774_5098629-5099735 GACACCTCGCAGCCCGATGAGCCGGTGCCCTCGCCCACCCCGGTGCCACC

XC_4266|66766352_5059416-5060537 GACACCTCCCAGCCCGATCAACCGGTGCCCTCGCCCACCCCGGTACCGCC

XCV4415|78045556_5090439-5091518 GACACCTCCCAGCCCGATCAGCCGGTGCCCTCGCCCACCCCGGTGCCACC

PXO_03524|188574270_5205512-5206558 GACACCTCGCAGCCCGATCAGCCGGTGCCCTCGCCCACCCCAGTGCCGCC

XCR_4519|384425691_4850609-4851724 GACACCTCCCAGCCCGATCAACCGGTGCCCTCGCCCACCCCGGTACCGCC

XOC_0202|384417079_c192798-191686 GACACCTCGCAGCCCGATCAGCCGGTGCCCTCGCCCACCCCAGTGCCGCC

XAC29_21730|470469929_5078310-5079416 GACACCTCGCAGCCCGATGAGCCGGTGCCCTCGCCCACCCCGGTGCCACC

XCAW_04682|471265562_5244573-5245679 GACACCTCGCAGCCCGATGAGCCGGTGCCCTCGCCCACCCCGGTGCCACC

******** ********* * ******************** ** ** **

XCC4179|21229478_4985218-4986339 ATCGCCCACACCCACGCCCGTGCAACCGCCGCCGGCTGCCTCGCCGGTGC

XAC4313|21240774_5098629-5099735 ------GACACCCACGCCCGTGCAGCCGCCACCGGCTGCCTCGCCGGTAC

XC_4266|66766352_5059416-5060537 ATCGCCCACACCCACGCCCGTGCAACCGCCGCCGGCTGCCTCGCCGGTGC

XCV4415|78045556_5090439-5091518 ------GACATCCACG---------------------------CCGGTGC

PXO_03524|188574270_5205512-5206558 AACGCCAACACCCACGCCCGTGCAGCCGCCCCCGGCCGCCTCGCCGGTGC

XCR_4519|384425691_4850609-4851724 ATCGCCCACACCCACGCCCGTGCAACCACCGCCGGCCGCCTCGCCGGTGC

XOC_0202|384417079_c192798-191686 AACGCCAATACCCACGCCCGTGCAGCCGCCCCCGGCCGCCTTGCCGGTGC

XAC29_21730|470469929_5078310-5079416 ------GACACCCACGCCCGTGCAGCCGCCACCGGCTGCCTCGCCGGTAC

XCAW_04682|471265562_5244573-5245679 ------GACACCCACGCCCGTGCAGCCGCCACCGGCTGCCTCGCCGGTAC

* * ***** ***** *

XCC4179|21229478_4985218-4986339 AGTCCACGCTGGTGCAGCAGGCCAAGAACCCGGTACCGCCGCAGGGCGAC

XAC4313|21240774_5098629-5099735 AGTCCACGCTGGTGCAGCAAGCCAAACATCCGGTGCCGCCGCAGGGCGAT

XC_4266|66766352_5059416-5060537 AGTCCACGCTGGTGCAGCAGGCCAAGAACCCGGTACCGCCGCAGGGCGAC

XCV4415|78045556_5090439-5091518 AGTCCACGCTGGTGCAGCAGGCCAAAAATCCGGTGCCGCGGCAGGGCGAT

PXO_03524|188574270_5205512-5206558 AGTCCACGCTGGTGCAGCAAGCCAAGAACCCGGTGCCGCCGCAGGGCAAC

XCR_4519|384425691_4850609-4851724 AGTCCACGCTGGTGCAGCAGGCCAAGAACCCGGTGCCGCCGCAGGGCGAC

XOC_0202|384417079_c192798-191686 AATCCACGCTGGTGCAGCAAGCCAAGAACCCGGTGCCGCCGCAGGGCAAC

XAC29_21730|470469929_5078310-5079416 AGTCCACGCTGGTGCAGCAAGCCAAACATCCGGTGCCGCCGCAGGGCGAT

XCAW_04682|471265562_5244573-5245679 AGTCCACGCTGGTGCAGCAAGCCAAACATCCGGTGCCGCCGCAGGGCGAT

* ***************** ***** * ***** **** ******* *

XCC4179|21229478_4985218-4986339 ACCGCGCCCGGCAGCCTGGCCGAACGCCGCCGACAAACTCGGCGCCAGAC

XAC4313|21240774_5098629-5099735 ACCGCGCAAGGCAGCCTGGCCGAACGCCGC---CAGCCGCGTCGCCAGCA

XC_4266|66766352_5059416-5060537 ACCGCGCCCGGCAGCCTGGCCGAACGCCGCCGACAAACTCGGCGCCAGAC

XCV4415|78045556_5090439-5091518 ACCGCGCAAGGCAGCCTGGCCGAACGCCGC---CAGCCGCGTCGCCAGCA

PXO_03524|188574270_5205512-5206558 ACCGCGCCGGGCAGCCTGGCCGAACGCCGC---CAGCCGCGTCGCCAGCA

XCR_4519|384425691_4850609-4851724 ACCGCGCCCGGCAGCCTGGCCGAACGTCGCCGCCAGACCCGGCGCCAGAC

XOC_0202|384417079_c192798-191686 ACCGCGCCGGGCAGCCTGGCCGAACGCCGC---CAGCCGCGTCGCCAGCA

XAC29_21730|470469929_5078310-5079416 ACCGCGCAAGGCAGCCTGGCCGAACGCCGC---CAGCCGCGTCGCCAGCA

XCAW_04682|471265562_5244573-5245679 ACCGCGCAAGGCAGCCTGGCCGAACGCCGC---CAGCCGCGTCGCCAGCA

******* ***************** *** ** * ** ******

XCC4179|21229478_4985218-4986339 CCGTCCCACTCCGCCGCAACCGCCGGCGCCGCCGGCCGCCTCCACCCAGC

XAC4313|21240774_5098629-5099735 ACGGCCCACGCCGCCGCAACCGCCCGCACCGCCGGCGGCATCCGCCCAGC

XC_4266|66766352_5059416-5060537 CCGTCCCACTCCGCCGCAACCGCCGGCGCCGCCGGCCGCCTCCACCCAGC

XCV4415|78045556_5090439-5091518 ACGCCCCACCCCGCCGCAACCGCCCGCTCCGCCGGCGGCATCCGCACAGC

PXO_03524|188574270_5205512-5206558 ACGGCCCACCCCGCCGCAACCGCCCGGCCCGCCGGCGGCATCCGCCCAGC

XCR_4519|384425691_4850609-4851724 CCGTCCCACCCCGCCGCTACCGCCGGCGCCGCCGGTCGCCTCCACCCAGC

XOC_0202|384417079_c192798-191686 ACGGCCCACCCCGCCGCAACCGCCCGGCCCGCCGGCGGCATCCGCCCAGC

XAC29_21730|470469929_5078310-5079416 ACGGCCCACGCCGCCGCAACCGCCCGCACCGCCGGCGGCATCCGCCCAGC

XCAW_04682|471265562_5244573-5245679 ACGGCCCACGCCGCCGCAACCGCCCGCACCGCCGGCGGCATCCGCCCAGC

** ***** ******* ****** * ******* ** *** * ****

XCC4179|21229478_4985218-4986339 GCCGCCCGGAAACCTGGACCGGCCGCCCGCCCGGCATGCTGGAAGAAGAG

XAC4313|21240774_5098629-5099735 GCCGCCCGGATACCTGGACCGGCCGCCCGCCGGGCATGCTCGACGAACCC

XC_4266|66766352_5059416-5060537 GCCGCCCGGAAACCTGGACCGGCCGCCCGCCCGGCATGCTGGAAGAAGAG

XCV4415|78045556_5090439-5091518 GCCGCCCGGACACCTGGACCGGCAGGCCGCCCGGCATGCTGGACGAACCG

PXO_03524|188574270_5205512-5206558 GCCGCCCGGATACCTGGACCGGCCGCCCGCCGGGCATGCTCGACGAACCC

XCR_4519|384425691_4850609-4851724 GCCGTCCCGAGACCTGGACCGGCCGCCCGCCCGGCATGCTGGAGGAAGAG

XOC_0202|384417079_c192798-191686 GCCGCCCGGATACCTGGACCGGCCGCCCGCCGGGCATGCTCGACGAACCC

XAC29_21730|470469929_5078310-5079416 GCCGCCCGGATACCTGGACCGGCCGCCCGCCGGGCATGCTCGACGAACCC

XCAW_04682|471265562_5244573-5245679 GCCGCCCGGATACCTGGACCGGCCGCCCGCCGGGCATGCTCGACGAACCC

**** ** ** ************ * ***** ******** ** ***

XCC4179|21229478_4985218-4986339 GCCGACGGCGCCGAAGACGGCACCGCCAACACCTCCACCATCAGCCAGGG

XAC4313|21240774_5098629-5099735 GCCGACGGCGCCGAAGACGGCATGGCCAACACCCCCACCATCAGCCAGGG

XC_4266|66766352_5059416-5060537 GCCGACGGCGCCGAAGACGGCACCGCCAACACCTCCACCATCAGCCAGGG

XCV4415|78045556_5090439-5091518 GCCGACGGCGCCGAAGACGGCATGGCCAACACCCCCACCATCAGCCAGGG

PXO_03524|188574270_5205512-5206558 GCCGACGGTGCCGAAGACGGCATGGCCAACACCCCCACCATCAGCCAGGG

XCR_4519|384425691_4850609-4851724 GCAGACGGCGCCGAAGACGGCACCGCCAACACCTCCACCATCAGCCAGGG

XOC_0202|384417079_c192798-191686 GCCGACGGTGCCGAAGACGGCATGGCCAACACCCCCACCATCAGCCAGGG

XAC29_21730|470469929_5078310-5079416 GCCGACGGCGCCGAAGACGGCATGGCCAACACCCCCACCATCAGCCAGGG

XCAW_04682|471265562_5244573-5245679 GCCGACGGCGCCGAAGACGGCATGGCCAACACCCCCACCATCAGCCAGGG

** ***** ************* ********* ****************

XCC4179|21229478_4985218-4986339 CCGCCGCAACGACCGCAACAACGCCCAGCCCAGCATGGATGTGGGCGGCT

XAC4313|21240774_5098629-5099735 CCGCCGCAACGACCGCAACAACGCCCAGCCCAGCCTGGATATCGGCGGCT

XC_4266|66766352_5059416-5060537 CCGCCGCAACGACCGCAACAACGCCCAGCCCAGCATGGATGTGGGCGGCT

XCV4415|78045556_5090439-5091518 CCGCCGCAACGACCGCAACAACGCCCAGCCCAGCCTGGATATCGGCGGCT

PXO_03524|188574270_5205512-5206558 CCGCCGCAACGACCGCAACAACGCTCAGCCCAGCCTGGATATCGGCGGCT

XCR_4519|384425691_4850609-4851724 CCGCCGCAACGACCGCAACAACGCCCAGCCCAGCATGGATGTGGGCGGCT

XOC_0202|384417079_c192798-191686 CCGCCGCAACGACCGCAACAACGCTCAGCCCAGCCTGGATATCGGCGGCT

XAC29_21730|470469929_5078310-5079416 CCGCCGCAACGACCGCAACAACGCCCAGCCCAGCCTGGATATCGGCGGCT

XCAW_04682|471265562_5244573-5245679 CCGCCGCAACGACCGCAACAACGCCCAGCCCAGCCTGGATATCGGCGGCT

************************ ********* ***** * *******

XCC4179|21229478_4985218-4986339 TCCAGGTGTATTACGAAGTGCGCAGCGAAACCCAGCTGCGCGCCTGGAAG

XAC4313|21240774_5098629-5099735 TCCAGGTCTATTACGACGTGCGCAGCGAAACCCAGCTGCGCGCCTGGAAA

XC_4266|66766352_5059416-5060537 TCCAGGTGTATTACGAAGTGCGCAGCGAAACCCAGCTGCGCGCCTGGAAG

XCV4415|78045556_5090439-5091518 TCCAGGTCTATTACGACGTGCGCAGCGAAACCCAGCTGCGCGCCTGGAAA

PXO_03524|188574270_5205512-5206558 TCCAGGTCTACTACGACGTGCGCAGCGAAACCCAGCTGCGCGCCTGGAAA

XCR_4519|384425691_4850609-4851724 TCCAGGTGTATTACGAAGTGCGCAGCGAAACCCAGCTGCGCGCCTGGAAG

XOC_0202|384417079_c192798-191686 TCCAGGTCTACTACGACGTGCGCAGCGAAACCCAGCTGCGCGCCTGGAAA

XAC29_21730|470469929_5078310-5079416 TCCAGGTCTATTACGACGTGCGCAGCGAAACCCAGCTGCGCGCCTGGAAA

XCAW_04682|471265562_5244573-5245679 TCCAGGTCTATTACGACGTGCGCAGCGAAACCCAGCTGCGCGCCTGGAAA

******* ** ***** ********************************

XCC4179|21229478_4985218-4986339 GAGCAAGGCATGCAGGAGATCGCCATCATCCTGCCCGGCACCCAGCAACG

XAC4313|21240774_5098629-5099735 GAACAAGGCATGCAGGAAATCGCCATCATCCTGCCCGGCACCCAGCAACG

XC_4266|66766352_5059416-5060537 GAGCAAGGCATGCAGGAGATCGCCATCATCCTGCCCGGCACCCAGCAACG

XCV4415|78045556_5090439-5091518 GAACAAGGCATGCAGGAAATCGCCATCATCCTGCCCGGCACCCAGCAACG

PXO_03524|188574270_5205512-5206558 GAACAAGGCATGCAGGAAATCGCCATCATCCTGCCCGGCACCCAGCAACG

XCR_4519|384425691_4850609-4851724 GAGCAAGGCATGCAGGAGATCGCCATCATCCTGCCCGGCACCCAGCAACG

XOC_0202|384417079_c192798-191686 GAACAAGGCATGCAGGAAATCGCCATCATCCTGCCCGGCACCCAGCAACG

XAC29_21730|470469929_5078310-5079416 GAACAAGGCATGCAGGAAATCGCCATCATCCTGCCCGGCACCCAGCAACG

XCAW_04682|471265562_5244573-5245679 GAACAAGGCATGCAGGAAATCGCCATCATCCTGCCCGGCACCCAGCAACG

** ************** ********************************

XCC4179|21229478_4985218-4986339 CATGGTCTGCCCGCTGGACGTCGCGCTCAAACGCGGCTCCAGCAAATGCC

XAC4313|21240774_5098629-5099735 CATGATCTGCCCGCTGGATGTCGCGCTCAAACGCGGCTCCAGCAAATGCC

XC_4266|66766352_5059416-5060537 CATGGTCTGCCCGCTGGACGTCGCGCTCAAACGCGGCTCCAGCAAATGCC

XCV4415|78045556_5090439-5091518 CATGATCTGCCCGCTGGATGTCGCGCTCAAGCGCGGCTCCAGCAAATGCC

PXO_03524|188574270_5205512-5206558 CATGATCTGCCCGCTGGATGTCGCGCTCAAACGCGGCTCCAGCAAATGCC

XCR_4519|384425691_4850609-4851724 CATGGTCTGCCCGCTGGACGTCGCGCTCAAACGCGGCTCCAGCAAATGCC

XOC_0202|384417079_c192798-191686 CATGATCTGCCCGCTGGATGTCGCGCTCAAACGCGGCTCCAGCAAATGCC

XAC29_21730|470469929_5078310-5079416 CATGATCTGCCCGCTGGATGTCGCGCTCAAACGCGGCTCCAGCAAATGCC

XCAW_04682|471265562_5244573-5245679 CATGATCTGCCCGCTGGATGTCGCGCTCAAACGCGGCTCCAGCAAATGCC

**** ************* *********** *******************

XCC4179|21229478_4985218-4986339 GCCTGCTGCCGCCGGATTCGCCCGAGCTCAAGAGCATTGGCGACGCCCGC

XAC4313|21240774_5098629-5099735 GCCTGCTGCCACCGGATTCGCCGGAGCTGAAGACCATCGGCGACGCGCGC

XC_4266|66766352_5059416-5060537 GCCTGCTGCCGCCGGATTCGCCCGAGCTCAAGAGCATTGGCGACGCCCGC

XCV4415|78045556_5090439-5091518 GCCTGCTGCCGCCGGATTCGCCCGAACTGAAGTCCATCGGCGACGCCCGC

PXO_03524|188574270_5205512-5206558 GCCTGCTCCCGCCGGATTCGCCCGAGCTGAAGTCCATCGGCGACGCCCGC

XCR_4519|384425691_4850609-4851724 GCCTGCTGCCGCCGGATTCGCCCGAGCTCAAGAGCATTGGCGACGCCCGC

XOC_0202|384417079_c192798-191686 GCCTGCTCCCGCCGGATTCGCCCGAGCTGAAGTCCATCGGCGACGCCCGC

XAC29_21730|470469929_5078310-5079416 GCCTGCTGCCACCGGATTCGCCGGAGCTGAAGACCATCGGCGACGCGCGC

XCAW_04682|471265562_5244573-5245679 GCCTGCTGCCACCGGATTCGCCGGAGCTGAAGACCATCGGCGACGCGCGC

******* ** *********** ** ** *** *** ******** ***

XCC4179|21229478_4985218-4986339 GAAGTCATCAACATGATGGAGGTCTACCGCCAGGGCGAACCGGTGTGGCG

XAC4313|21240774_5098629-5099735 GAGGTCATCAACATGATGGAGGTCTACCGCCAGGGCGAACCGGTCTGGCG

XC_4266|66766352_5059416-5060537 GAAGTCATCAACATGATGGAGGTCTACCGCCAGGGCGAACCGGTGTGGCG

XCV4415|78045556_5090439-5091518 GAAGTCATCAACATGATGGAGGTTTACCGCCAGGGCGAACCGGTCTGGCG

PXO_03524|188574270_5205512-5206558 GAAGTCATCAACATGATGGAGGTCTACCGCCAGGGCGAACCGGTTTGGCG

XCR_4519|384425691_4850609-4851724 GAAGTCATCAACATGATGGAGGTCTACCGCCAGGGCGAACCGGTGTGGCG

XOC_0202|384417079_c192798-191686 GAAGTCATCAACATGATGGAGGTCTACCGCCAGGGCGAACCGGTTTGGCG

XAC29_21730|470469929_5078310-5079416 GAGGTCATCAACATGATGGAGGTCTACCGCCAGGGCGAACCGGTCTGGCG

XCAW_04682|471265562_5244573-5245679 GAGGTCATCAACATGATGGAGGTCTACCGCCAGGGCGAACCGGTCTGGCG

** ******************** ******************** *****

XCC4179|21229478_4985218-4986339 CGGGCCTGGACCGTATCGGTAA

XAC4313|21240774_5098629-5099735 TGGGCCGGGGCCGTATCGGTGA

XC_4266|66766352_5059416-5060537 CGGGCCTGGACCGTATCGGTAA

XCV4415|78045556_5090439-5091518 TGGGCCGGGGCCGTATCGCTGA

PXO_03524|188574270_5205512-5206558 TGGGCCGGGGCCGTATCGGTGA

XCR_4519|384425691_4850609-4851724 CGGGCCTGGGCCGTATCGGTGA

XOC_0202|384417079_c192798-191686 TGGGCCGGGGCCGTATCGGTGA

XAC29_21730|470469929_5078310-5079416 TGGGCCGGGGCCGTATCGGTGA

XCAW_04682|471265562_5244573-5245679 TGGGCCGGGGCCGTATCGGTGA

***** ** ******** * *

PROTEIN ALIGNMENTS:

XCC4179|AAM43395.1|conserved MLMLRRDWTVPAATQLAHAQDHYHALNPTAAAAMARQVREATRTLAEQPG

XAC4313|AAM39143.1|conserved --MLRRDWTVPAATQLAHAQDHYHALNPTAAAAMARQVLEATRTLAEQPG

XC_4266|AAY51304.1|conserved MLMLRRDWTVPAATQLAHAQDHYHALNPTAAAAMARQVREATRTLAEQPG

XCV4415|CAJ26146.1|putative --MLRRDWTVPAATQLAHAQDHYHALNPTAAAAMARQVLEATRTLAEQPG

PXO_03524|ACD61624.1|plasmid ------------------------MLNPTAAAAMARQVLEATRALAEQPG

XCR_4519|AEL09369.1| --MLRRDWTVPAATQLAHAQDHYHALNPTAAAAMARQVREATRTLAEQPG

XOC_0202|AEQ94449.1|plasmid --MLRRDWTVPAATQLAHAQDHYHALNPTAAAAMARQVLEATRTLAEQPG

XAC29_21730|AGH79727.1|hypothetical --MLRRDWTVPAATQLAHAQDHYHALNPTAAAAMARQVLEATRTLAEQPG

XCAW_04682|AGI10440.1|Hypothetical --MLRRDWTVPAATQLAHAQDHYHALNPTAAAAMARQVLEATRTLAEQPG

************* ****:******

XCC4179|AAM43395.1|conserved RGRAGRVAGTREWTVKHTPYVLVYRVRDDALQLLHVRLETQDWLPRTEPV

XAC4313|AAM39143.1|conserved RGRVGRVPGTREWVVKQTPYVLVYRVRDGALQVLHVHLDASDWLPRTEPL

XC_4266|AAY51304.1|conserved RGRAGRVAGTREWTVKHTPYVLVYRVRDDALQLLHVRLETQDWLPRTEPV

XCV4415|CAJ26146.1|putative RGRVGRAPGTREWVVKQTPYVLVYRVREGALQVLHVHLDASDWLPRTEPL

PXO_03524|ACD61624.1|plasmid RGRAGRVPGTREWVVKQTPYVLVYRVRDGALQVLHVHLDASDWLPRTEPL

XCR_4519|AEL09369.1| RGRAGRVAGTREWTVKHTPYVLVYRVRDDALQLLHVRLETQDWLPRTEPV

XOC_0202|AEQ94449.1|plasmid RGRAGRVPGTREWVVKQTPYVLVYRVRDGALQVLHVHLDASDWLPRTEPL

XAC29_21730|AGH79727.1|hypothetical RGRVGRVPGTREWVVKQTPYVLVYRVRDGALQVLHVHLDASDWLPRTEPL

XCAW_04682|AGI10440.1|Hypothetical RGRVGRVPGTREWVVKQTPYVLVYRVRDGALQVLHVHLDASDWLPRTEPL

***.**..*****.**:**********:.***:***:*::.********:

XCC4179|AAM43395.1|conserved IERIEPWIAALISALLHVLMLLILLSASTPSMTPPQGSASGGRTKVDFVG

XAC4313|AAM39143.1|conserved IERIEPWIAALISALLHVLMLLILLSASTPTMTPPQGSASGGRIKVDFVG

XC_4266|AAY51304.1|conserved IERIEPWIAALISALLHVLMLLILLSASTPSMTPPQGSASGGRTKVDFVG

XCV4415|CAJ26146.1|putative IERIEPWIAALISALLHVLMLLILLSASTPTMTPPQGSASGGRIKVDFVG

PXO_03524|ACD61624.1|plasmid IERIEPWIAALISALLHVLMLLILLSASTPTMTPPQGSASGGRIKVDFVG

XCR_4519|AEL09369.1| IERIEPWIAALISALLHVLMLLILLSASTPSMTPPQGSASGGRTKVDFVG

XOC_0202|AEQ94449.1|plasmid IERIEPWIAALISALLHVLMLLILLSASTPTMTPPQGSASGGRIKVDFVG

XAC29_21730|AGH79727.1|hypothetical IERIEPWIAALISALLHVLMLLILLSASTPTMTPPQGSASGGRIKVDFVG

XCAW_04682|AGI10440.1|Hypothetical IERIEPWIAALISALLHVLMLLILLSASTPTMTPPQGSASGGRIKVDFVG

******************************:************ ******

XCC4179|AAM43395.1|conserved DTSQPDQPVPSPTPVPPSPTPTPVQPPPAASPVQSTLVQQAKNPVPPQGD

XAC4313|AAM39143.1|conserved DTSQPDEPVPSPTPVPPTP--TPVQPPPAASPVQSTLVQQAKHPVPPQGD

XC_4266|AAY51304.1|conserved DTSQPDQPVPSPTPVPPSPTPTPVQPPPAASPVQSTLVQQAKNPVPPQGD

XCV4415|CAJ26146.1|putative DTSQPDQPVPSPTPVPPTS-----------TPVQSTLVQQAKNPVPRQGD

PXO_03524|ACD61624.1|plasmid DTSQPDQPVPSPTPVPPTPTPTPVQPPPAASPVQSTLVQQAKNPVPPQGN

XCR_4519|AEL09369.1| DTSQPDQPVPSPTPVPPSPTPTPVQPPPAASPVQSTLVQQAKNPVPPQGD

XOC_0202|AEQ94449.1|plasmid DTSQPDQPVPSPTPVPPTPIPTPVQPPPAALPVQSTLVQQAKNPVPPQGN

XAC29_21730|AGH79727.1|hypothetical DTSQPDEPVPSPTPVPPTP--TPVQPPPAASPVQSTLVQQAKHPVPPQGD

XCAW_04682|AGI10440.1|Hypothetical DTSQPDEPVPSPTPVPPTP--TPVQPPPAASPVQSTLVQQAKHPVPPQGD

******:**********:. ***********:*** **:

XCC4179|AAM43395.1|conserved TAPGSLAERRRQTRRQTRPTPPQPPAPPAASTQRRPETWTGRPPGMLEEE

XAC4313|AAM39143.1|conserved TAQGSLAER-RQPRRQQRPTPPQPPAPPAASAQRRPDTWTGRPPGMLDEP

XC_4266|AAY51304.1|conserved TAPGSLAERRRQTRRQTRPTPPQPPAPPAASTQRRPETWTGRPPGMLEEE

XCV4415|CAJ26146.1|putative TAQGSLAER-RQPRRQQRPTPPQPPAPPAASAQRRPDTWTGRPPGMLDEP

PXO_03524|ACD61624.1|plasmid TAPGSLAER-RQPRRQQRPTPPQPPGPPAASAQRRPDTWTGRPPGMLDEP

XCR_4519|AEL09369.1| TAPGSLAERRRQTRRQTRPTPPLPPAPPVASTQRRPETWTGRPPGMLEEE

XOC_0202|AEQ94449.1|plasmid TAPGSLAER-RQPRRQQRPTPPQPPGPPAASAQRRPDTWTGRPPGMLDEP

XAC29_21730|AGH79727.1|hypothetical TAQGSLAER-RQPRRQQRPTPPQPPAPPAASAQRRPDTWTGRPPGMLDEP

XCAW_04682|AGI10440.1|Hypothetical TAQGSLAER-RQPRRQQRPTPPQPPAPPAASAQRRPDTWTGRPPGMLDEP

** ****** **.*** ***** **.**.**:****:**********:*

XCC4179|AAM43395.1|conserved ADGAEDGTANTSTISQGRRNDRNNAQPSMDVGGFQVYYEVRSETQLRAWK

XAC4313|AAM39143.1|conserved ADGAEDGMANTPTISQGRRNDRNNAQPSLDIGGFQVYYDVRSETQLRAWK

XC_4266|AAY51304.1|conserved ADGAEDGTANTSTISQGRRNDRNNAQPSMDVGGFQVYYEVRSETQLRAWK

XCV4415|CAJ26146.1|putative ADGAEDGMANTPTISQGRRNDRNNAQPSLDIGGFQVYYDVRSETQLRAWK

PXO_03524|ACD61624.1|plasmid ADGAEDGMANTPTISQGRRNDRNNAQPSLDIGGFQVYYDVRSETQLRAWK

XCR_4519|AEL09369.1| ADGAEDGTANTSTISQGRRNDRNNAQPSMDVGGFQVYYEVRSETQLRAWK

XOC_0202|AEQ94449.1|plasmid ADGAEDGMANTPTISQGRRNDRNNAQPSLDIGGFQVYYDVRSETQLRAWK

XAC29_21730|AGH79727.1|hypothetical ADGAEDGMANTPTISQGRRNDRNNAQPSLDIGGFQVYYDVRSETQLRAWK

XCAW_04682|AGI10440.1|Hypothetical ADGAEDGMANTPTISQGRRNDRNNAQPSLDIGGFQVYYDVRSETQLRAWK

******* ***.****************:*:*******:***********

XCC4179|AAM43395.1|conserved EQGMQEIAIILPGTQQRMVCPLDVALKRGSSKCRLLPPDSPELKSIGDAR

XAC4313|AAM39143.1|conserved EQGMQEIAIILPGTQQRMICPLDVALKRGSSKCRLLPPDSPELKTIGDAR

XC_4266|AAY51304.1|conserved EQGMQEIAIILPGTQQRMVCPLDVALKRGSSKCRLLPPDSPELKSIGDAR

XCV4415|CAJ26146.1|putative EQGMQEIAIILPGTQQRMICPLDVALKRGSSKCRLLPPDSPELKSIGDAR

PXO_03524|ACD61624.1|plasmid EQGMQEIAIILPGTQQRMICPLDVALKRGSSKCRLLPPDSPELKSIGDAR

XCR_4519|AEL09369.1| EQGMQEIAIILPGTQQRMVCPLDVALKRGSSKCRLLPPDSPELKSIGDAR

XOC_0202|AEQ94449.1|plasmid EQGMQEIAIILPGTQQRMICPLDVALKRGSSKCRLLPPDSPELKSIGDAR

XAC29_21730|AGH79727.1|hypothetical EQGMQEIAIILPGTQQRMICPLDVALKRGSSKCRLLPPDSPELKTIGDAR

XCAW_04682|AGI10440.1|Hypothetical EQGMQEIAIILPGTQQRMICPLDVALKRGSSKCRLLPPDSPELKTIGDAR

******************:*************************:*****

XCC4179|AAM43395.1|conserved EVINMMEVYRQGEPVWRGPGPYR

XAC4313|AAM39143.1|conserved EVINMMEVYRQGEPVWRGPGPYR

XC_4266|AAY51304.1|conserved EVINMMEVYRQGEPVWRGPGPYR

XCV4415|CAJ26146.1|putative EVINMMEVYRQGEPVWRGPGPYR

PXO_03524|ACD61624.1|plasmid EVINMMEVYRQGEPVWRGPGPYR

XCR_4519|AEL09369.1| EVINMMEVYRQGEPVWRGPGPYR

XOC_0202|AEQ94449.1|plasmid EVINMMEVYRQGEPVWRGPGPYR

XAC29_21730|AGH79727.1|hypothetical EVINMMEVYRQGEPVWRGPGPYR

XCAW_04682|AGI10440.1|Hypothetical EVINMMEVYRQGEPVWRGPGPYR

***********************

**pfam05016 - plasm. stab. prot. RelE/ParE (3)**

NUCLEOTIDE ALIGNMENTS:

XCC1066|21229478_1227116-1227424 GTGGGA----TATTCCGTC-----CGCTTCACTCAGTACGCGCGCAACGA

XC_3185|66766352_c3818651-3818343 GTGGGA----TTTGCCGTC-----CGCTTCACTCAGCAAGCGCGCGACGA

XCV1189|78045556_1328426-1328734 GTGGGA----TTTTCTGTC-----CGCTTCACTCAGGAAGCGCGCGACGA

PXO_02069|188574270_c3836067-3835759 GTGGCA----TTTTTCGTT-----CGCTTCACGCAGCAAGCGCGCGAGGA

XCR_1272|384425691_1292642-1292950 GTGGGA----TTCTCTGTC-----CGCTTCACTCAGGAAGCGCGCGACGA

XOC_1220|384417079_1189950-1190153 GTGGGA----TTGTCCGTT-----CGCTTCACGCAGCAAGCGCGCGAGGA

XALq_3234|386081561_30773-31072 ATGGCCAAGAAGGCCCATGCGGTCGAGTGGACGCAATTCGCGCTCGATGA

*** * * ** ** **** * * **

XCC1066|21229478_1227116-1227424 TCTCGCGCGTCTTT-ACGACTGGCTGCTACAGCGTGCCGAAAGCGGCTTC

XC_3185|66766352_c3818651-3818343 TCTCGCGCGGCTTT-ACGACTGGCTGCTACAGCGTGCCGAAGGCGACTTC

XCV1189|78045556_1328426-1328734 TTTCGCGCGCCTTT-ACGACTGGCCGCTACAGCGTGCCGAAGGCGACTTC

PXO_02069|188574270_c3836067-3835759 TCTCGTGCGTCTTT-ACGACTGGCTGCTCCAGCGTGTCGATGGCGACTTC

XCR_1272|384425691_1292642-1292950 TCTCGCGCGTCTTT-ACGACTGGCTGCTACAGCGTGCCGAAGGCGACTTC

XOC_1220|384417079_1189950-1190153 TCTCGTGCGTCTTT-ACGACTGGCTGCTCCAGCGTGCCGAAGGCGACTTC

XALq_3234|386081561_30773-31072 TCTTGAA-GCCATCATCGAC-------TACATCGC--TCATGACAACGCC

* * * * * * **** * ** ** * * * *

XCC1066|21229478_1227116-1227424 ACCGTGGTCGAGCGCG-CGCTGCAGGCCATCCGCGACGGCGTCACCGTGC

XC_3185|66766352_c3818651-3818343 ACCGTGGCCGAGCGCG-CGCTGCAGGCCATCCGCGATGGCGTCACCGTGC

XCV1189|78045556_1328426-1328734 ACCCTGGCCAAGCGCG-CGCTGCAGGCCGTCGGCGACGGCGTCACCGTGC

PXO_02069|188574270_c3836067-3835759 ACCGTGGCCGAGCGCG-CACTGCAGGCCATCGGCGACGGCGTCACCGTGC

XCR_1272|384425691_1292642-1292950 ACCGTGGCCGAGCGCG-CGCTGCAGGCGATCCGCGACGGCGTCACCGTGC

XOC_1220|384417079_1189950-1190153 ACCGTGGCCGAGCGCG-CGCTGCAGGCCATCGGCGACGGCGTCACCGTAC

XALq_3234|386081561_30773-31072 GAGCGGGCCTACGGCTTCGCGGCCGA-AATTTACGAGAAGACGACCGCGC

** * * ** * * ** * * *** **** *

XCC1066|21229478_1227116-1227424 TGGCACTTGCCCCGTTGAGCTGCCGCAAGGCGGTGCCGGCA-GACCCGTT

XC_3185|66766352_c3818651-3818343 TTGAACTGGCCCCGTTGAGTTGCCGCAAGGTGGGGTTGGCG-GACCCGTT

XCV1189|78045556_1328426-1328734 TTGAACTGGCCCCTTTGAGCTGCCGCAAGGCGGGGTTGGCG-GACCCGTT

PXO_02069|188574270_c3836067-3835759 TTGAGCTGGCCGCGTTGAGCTGTCGCAAGGCGGGGGTGGCA-GATCCGTT

XCR_1272|384425691_1292642-1292950 TCGAACTGGCCCCGTTGAGCTGCCGCAAGGCGGGGTTGGCG-GACCCGTT

XOC_1220|384417079_1189950-1190153 TTGAACTGGCCGCGTTGAGCTGTCGCAAGGCGGGG-GGGCA-GATCCGTT

XALq_3234|386081561_30773-31072 TCGAGCGGCATCC--AGAGATA-GGC-AGGCCGGGTCGACCTGGTT--TG

* * * * *** * ** *** * * * * * *

XCC1066|21229478_1227116-1227424 CTTGCGGGAGTTGGTGATCGGCTTCGGTGCCAGCGGCTATGTGCTGCTGT

XC_3185|66766352_c3818651-3818343 CTTGCGGGAGCTGGTGATCGGCTTGGGTGCCAGCGGCTACGTGCTGCTGT

XCV1189|78045556_1328426-1328734 CTTGCGGGAGCTGGTGATCGGCTTCGGTGCAAGCGGTTATGTGCTGCTGT

PXO_02069|188574270_c3836067-3835759 CTTGCGGGAGCTGGTGATCGGCGTCGGTGCCAGCGGCTACGTGCTGCTGT

XCR_1272|384425691_1292642-1292950 CTTGCGGGAGCTGGTGATCGGCTTCGGTGCCAGCGGCTACGTCCTGCTGT

XOC_1220|384417079_1189950-1190153 CTTGCGGGAGCTG-------------------------------------

XALq_3234|386081561_30773-31072 CCTGCAGGCGTGCGGGAGTTGGTCGTGCACC-GGAATTACATCGTTCTGT

* *** ** *

XCC1066|21229478_1227116-1227424 TTGAGGTTGAAAGCAATCAGGTCGTCACCGTTTTGGCAGTCAGGCATCAG

XC_3185|66766352_c3818651-3818343 TTGAGGTGGAAAGCAATCAGGTCGTCACCGTTTTGGCAGTCAGGCATCAG

XCV1189|78045556_1328426-1328734 TTGAGGTTGAAAGCGATCAGGTCGTCACCGTTTTGGCAGTCAGGCATCGG

PXO_02069|188574270_c3836067-3835759 TCGAGGTGGAAAGCGACCAGGTCGTCACCGTTTTGGCAGTCAGGCATCAG

XCR_1272|384425691_1292642-1292950 TTGAGGTGGAAAGCGATCAGGTCGTCACCGTTTTAGCAATCAGGCATCAG

XOC_1220|384417079_1189950-1190153 --------------------------------------------------

XALq_3234|386081561_30773-31072 ACCAGTTGCTACCTGGCGACGACGTGGAGATCCTGCGCGTGAAGCA-CAC

XCC1066|21229478_1227116-1227424 CGTGAAGACGACTATCATTGA

XC_3185|66766352_c3818651-3818343 CGTGAAGACGACTATCATTAA

XCV1189|78045556_1328426-1328734 CGTGAAGACGACTATCATTAA

PXO_02069|188574270_c3836067-3835759 CGTGAAGACGACTATCATTAG

XCR_1272|384425691_1292642-1292950 TGTGAAGACGACTATCATTAA

XOC_1220|384417079_1189950-1190153 -----------------GTGA

XALq_3234|386081561_30773-31072 CGCGCAG-CGAATACCG-TGA

*

PROTEIN ALIGNMENTS:

XCC1066|AAM40365.1|conserve MG---YSVRFTQYARNDLARLYDWLLQ-RAESGFTVVERALQAIRDGVTV

XC_3185|AAY50229.1|conserved MG---FAVRFTQQARDDLARLYDWLLQ-RAEGDFTVAERALQAIRDGVTV

XCV1189|CAJ22820.1|conserved MG---FSVRFTQEARDDFARLYDWPLQ-RAEGDFTLAKRALQAVGDGVTV

PXO_02069|ACD60308.1|plasmid MA---FFVRFTQQAREDLVRLYDWLLQ-RVDGDFTVAERALQAIGDGVTV

XCR_1272|AEL06172.1|plasmid MG---FSVRFTQEARDDLARLYDWLLQ-RAEGDFTVAERALQAIRDGVTV

XOC_1220|AEQ95408.1|plasmid MG---LSVRFTQQAREDLVRLYDWLLQ-RAEGDFTVAERALQAIGDGVTV

XALq_3234|WP_014607104.1| MAKKAHAVEWTQFALDDLEAIIDYIAHDNAERAYGFA----AEIYEKTTA

*. *.:** * :*: : *: : ..: : .. : : .*.

XCC1066|AAM40365.1|conserved LALAPLSCRKAVPADP-FLRELVIGFGASGYVLLFEVESNQVVTVLAVRH

XC_3185|AAY50229.1|conserved LELAPLSCRKVGLADP-FLRELVIGLGASGYVLLFEVESNQVVTVLAVRH

XCV1189|CAJ22820.1|conserved LELAPLSCRKAGLADP-FLRELVIGFGASGYVLLFEVESDQVVTVLAVRH

PXO_02069|ACD60308.1|plasmid LELAALSCRKAGVADP-FLRELVIGVGASGYVLLFEVESDQVVTVLAVRH

XCR_1272|AEL06172.1|plasmid LELAPLSCRKAGLADP-FLRELVIGFGASGYVLLFEVESDQVVTVLAIRH

XOC_1220|AEQ95408.1|plasmid LELAALSCRKAGGQIR-SCGSW----------------------------

XALq_3234|WP_014607104.1| LERHPEIGRPGRPGLPAGVRELVVH---RNYIVLYQLLPGDDVEILRVKH

* . * .

XCC1066|AAM40365.1|conserved QREDDYH

XC_3185|AAY50229.1|conserved QREDDYH

XCV1189|CAJ22820.1|conserved RREDDYH

PXO_02069|ACD60308.1|plasmid QREDDYH

XCR_1272|AEL06172.1|plasmid QCEDDYH

XOC_1220|AEQ95408.1|plasmid -------

XALq_3234|WP_014607104.1| TAQRIP-

**pfam05016 - plasm. stab. prot. RelE/ParE (4)**

NUCLEOTIDE ALIGNMENTS:

XCC3092|21229478_c3665126-3664824 GTGACCAGGATCGCGCTTGCCGCGCGCGTCGCCGACGATGCGCGCCGTTT

XC_1066|66766352_1283252-1283554 GTGACCAGGATCGCGCTTGCCGCGCGCGTCGCCGACGATGCGCGCCGTTT

XCV3346|78045556_c3832236-3831946 GTG------------CTTGCCGCGCGCGTTGCCGACGATGCACGCCGTCT

*** ************** *********** ****** *

XCC3092|21229478_c3665126-3664824 AGTCGCTTATCTGCTGGAACACGATGCAGCCAGTGTGCAGCGAAAGCTCG

XC_1066|66766352_1283252-1283554 AGTCGCTTATCTGCTGGAACACGATGCAGCCAGTGTGCAGCGAAAGCTCG

XCV3346|78045556_c3832236-3831946 GGTCGCCTATCTGCTGGAACACGATGCAGCCAATGTGCAGCGAAAACTCG

***** ************************* ************ ****

XCC3092|21229478_c3665126-3664824 CTGGCATCTTCCAGGCGATCGACGCATTGGCGGACAATCCACTGATGGGG

XC_1066|66766352_1283252-1283554 CTGGCATCTTCCAGGCGATCGACGCATTGGCGGACAATCCACTGATGGGG

XCV3346|78045556_c3832236-3831946 CAGGTGTCTTCCAGGCCATCGATGCATTGGCGGACAATCCACTGATAGGG

* ** ********** ***** *********************** ***

XCC3092|21229478_c3665126-3664824 CGCGCAGTGCATGGCGGATTTCGCGAACTGGTCATAGGCCGCGATGCCAG

XC_1066|66766352_1283252-1283554 CGCGCAGTGCATGGCGGATTTCGCGAACTGGTCATAGGCCGCGATGCCAG

XCV3346|78045556_c3832236-3831946 CGCGCGGTGCAGGGCGGATTCCGCGAACTGGTCATCGGTCGCGATGCCAG

***** ***** ******** ************** ** ***********

XCC3092|21229478_c3665126-3664824 CGGCTATCTGGCGCTCTATCGTTACGCACCGTTGGACGACACCGTGTATG

XC_1066|66766352_1283252-1283554 CGGCTATCTGGCGCTCTATCGTTACGCACCGTTGGACGACACCGTGTATG

XCV3346|78045556_c3832236-3831946 CGGCTATCTTGCGCTCTATCGTTACGCACCGTTGGACGACACCGTGTACG

********* ************************************** *

XCC3092|21229478_c3665126-3664824 TGCTGGCGATCCGTTCGCAGCGCGAAGCGGGTTACCTTGAAAGCCTGTTT

XC_1066|66766352_1283252-1283554 TGCTGGCGATCCGTTCGCAGCGCGAAGCGGGTTACCTTGAAAGCCTGTTT

XCV3346|78045556_c3832236-3831946 TGCTGGCGATCCGTTCGCAGCGCGAAGCGGGTTACCTTGAAAGCCTGTTT

**************************************************

XCC3092|21229478_c3665126-3664824 TGA

XC_1066|66766352_1283252-1283554 TGA

XCV3346|78045556_c3832236-3831946 TGA

***

PROTEIN ALIGNMENTS:

XCC3092|AAM42363.1|conserved MTRIALAARVADDARRLVAYLLEHDAASVQRKLAGIFQAIDALADNPLMG

XC_1066|AAY48136.1|conserved MTRIALAARVADDARRLVAYLLEHDAASVQRKLAGIFQAIDALADNPLMG

XCV3346|CAJ25077.1|conserved ----MLAARVADDARRLVAYLLEHDAANVQRKLAGVFQAIDALADNPLIG

**********************.*******:************:*

XCC3092|AAM42363.1|conserved RAVHGGFRELVIGRDASGYLALYRYAPLDDTVYVLAIRSQREAGYLESLF

XC_1066|AAY48136.1|conserved RAVHGGFRELVIGRDASGYLALYRYAPLDDTVYVLAIRSQREAGYLESLF

XCV3346|CAJ25077.1|conserved RAVQGGFRELVIGRDASGYLALYRYAPLDDTVYVLAIRSQREAGYLESLF

***:**********************************************

**4 - DOMAIN TIGR01552/pfam02604 - phd/COG2161 - StbD**

NUCLEOTIDE ALIGNMENTS:

XAC0080|21240774_99461-99718 ATGC----G-----------CACCGAACTTGTCACCA----CGCTCAAGC

XCAW_00469|471265562_560954-561211 ATGC----G-----------CACCGAACTTGTCACCA----CGCTCAAGC

XAC29_00405|470469929_99494-99751 ATGC----G-----------CACCGAACTTGTCACCA----CGCTCAAGC

XCV0057|78045556_69816-70073 ATGC----G-----------CACCGAACTTGTCACCA----CGCTCAAGC

PXO_03336|188574270_c148941-148570 TTGGAAGCGACTGCCGACGACATCGCAATTGACAACGTGCCCGCTGCAGC

XOC_0151|384417079_156081-156311 ATGC----G-----------CACCGAACGTGTCACCA----CGCTCAAGC

** * ** ** * ** ** * **** ***

XAC0080|21240774_99461-99718 GT--------------------------------CAGGCGACCGAACTCC

XCAW_00469|471265562_560954-561211 GT--------------------------------CAGGCGACCGAACTCC

XAC29_00405|470469929_99494-99751 GT--------------------------------CAGGCGACCGAACTCC

XCV0057|78045556_69816-70073 GT--------------------------------CAGGCGACCGAACCCC

PXO_03336|188574270_c148941-148570 GGCGCTGGTGAAACGGGTGTTCGCGCACGTTGAGCAGTTGATCGAACCCC

XOC_0151|384417079_156081-156311 GT--------------------------------CAGGCGACCGAACTTC

* *** ** ***** *

XAC0080|21240774_99461-99718 TTG-CAGCTGCAGAGCGCGACAAGGAGCCAATCC----T----------G

XCAW_00469|471265562_560954-561211 TTG-CAGCTGCAGAGCGCGACAAGGAGCCAATCC----T----------G

XAC29_00405|470469929_99494-99751 TTG-CAGCTGCAGAGCGCGACAAGGAGCCAATCC----T----------G

XCV0057|78045556_69816-70073 TTG-CAGCTGCAGAGCGCGACAAGGAGCCAATCC----T----------G

PXO_03336|188574270_c148941-148570 CGGACAGTGGAAGCCGGCTACAGGAACTCAAGCGCTCGCGCTATCGCCAG

XOC_0151|384417079_156081-156311 TTG-CAGCTACAGAGCGCGACAAGGAGCCAATCC----T----------G

* *** ** ** *** * * *** * *

XAC0080|21240774_99461-99718 ATC--------AC---ACAGCACGGTTTACCCAG---------TGCCTAT

XCAW_00469|471265562_560954-561211 ATC--------AC---ACAGCACGGTTTACCCAG---------TGCCTAT

XAC29_00405|470469929_99494-99751 ATC--------AC---ACAGCACGGTTTACCCAG---------TGCCTAT

XCV0057|78045556_69816-70073 ATC--------AC---ACAGCACGGTTTACCCAG---------AGCCTAT

PXO_03336|188574270_c148941-148570 ATTGTCGAACCACCTTGCCGCGTGATCTACCGCTTGGATGGGCAGCGCAT

XOC_0151|384417079_156081-156311 ATC--------AC---ACAGCACGGTTTACCCAG---------CGCCTAT

** ** * ** * * **** ** **

XAC0080|21240774_99461-99718 C----TGGTGGATGTTGCCAGCTATGAGCGCATGCA--GCAGCGCATTGC

XCAW_00469|471265562_560954-561211 C----TGGTGGATGTTGCCAGCTATGAGCGCATGCA--GCAGCGCATTGC

XAC29_00405|470469929_99494-99751 C----TGGTGGATGTTGCCAGCTATGAGCGCATGCA--GCAGCGCATTGC

XCV0057|78045556_69816-70073 G----TGGTGGATGTTGCCAGCGATGAGCGCATGCA--GCAGCGCATTGC

PXO_03336|188574270_c148941-148570 CGTGGTGGTGCACGTGTTGCGCTCGGAGCGCGCGCTTCGCA-AGCGCCGC

XOC_0151|384417079_156081-156311 C----TGGGGGATGTTGCCAGCTATGAGCGCATGCA--GCAGCGCATGGT

*** * * ** ** ****** ** *** ** *

XAC0080|21240774_99461-99718 GCTCCTCGAAGGAATCGCCCGTGGAGA-AATG--GCAGTCGCG--GAAG-

XCAW_00469|471265562_560954-561211 GCTCCTCGAAGGAATCGCCCGTGGAGA-AATG--GCAGTCGCG--GAAG-

XAC29_00405|470469929_99494-99751 GCTCCTCGAAGGAATCGCCCGTGGAGA-AATG--GCAGTCGCG--GAAG-

XCV0057|78045556_69816-70073 GCTCCTGGAAGGAATCGCCCGTGGAGA-AATG--GCAGTCGCG--CAAG-

PXO_03336|188574270_c148941-148570 ATTTCACGCTGGAAGCGGCTAACAAAACGACGGCGCAGCCGCCCGGTGGG

XOC_0151|384417079_156081-156311 GATCCTCGAAGGAATCGCCCGTGGAGA-AATG--GCAGTAGCG--CAAG-

* * * **** ** * * * * * **** ** *

XAC0080|21240774_99461-99718 ----G-CCGTACGCTGAGC--CATGAACAG-GC-ACGGCACCGCATG---

XCAW_00469|471265562_560954-561211 ----G-CCGTACGCTGAGC--CATGAACAG-GC-ACGGCACCGCATG---

XAC29_00405|470469929_99494-99751 ----G-CCGTACGCTGAGC--CATGAACAG-GC-ACGGCACCGCATG---

XCV0057|78045556_69816-70073 ----G-CCGTACGCTGCGC--CATGAACAG-GC-ACAGCAACACATG---

PXO_03336|188574270_c148941-148570 CGCGGCCTGTGCCCCGTGCTGCATGGACAACGCTTCCACTCCGGTTGTGA

XOC_0151|384417079_156081-156311 ----G-CCGCACGCTGACC--CATGAACAG-GC-----------------

* * * * * * * **** *** **

XAC0080|21240774_99461-99718 GCGCGATGGCT-----GAAATAA

XCAW_00469|471265562_560954-561211 GCGCGATGGCT-----GAAATAA

XAC29_00405|470469929_99494-99751 GCGCGATGGCT-----GAAATAA

XCV0057|78045556_69816-70073 GCGCGATGGCT-----GAAATAA

PXO_03336|188574270_c148941-148570 ATGCACTGTCCACACCCGGCTGA

XOC_0151|384417079_156081-156311 -------------------ATAG

*

PROTEIN ALIGNMENTS:

XAC0080|AAM34972.1| MRTE--------------------------------------LVTTLKRQ

XCAW_00469|AGI06288.1| MRTE--------------------------------------LVTTLKRQ

XAC29_00405|AGH75617.1| MRTE--------------------------------------LVTTLKRQ

XCV0057|CAJ21688.1| MRTE--------------------------------------LVTTLKRQ

PXO_03336|ACD56820.1| MEATADDIAIDNVPAAAALVKRVFAHVEQLIEPPDSGSRLQELKRSRYRQ

XOC_0151|ACD56820.1| MEATADDIAIDNVPAAAALVKRVFAHVEQLIEPPDSGSRLQELKRSRYRQ

*.: * : **

XAC0080|AAM34972.1| ATELLAAAERDKEPILITQHGLPSAYLVDVASYERMQQRIALLEGIARGE

XCAW_00469|AGI06288.1| ATELLAAAERDKEPILITQHGLPSAYLVDVASYERMQQRIALLEGIARGE

XAC29_00405|AGH75617.1| ATELLAAAERDKEPILITQHGLPSAYLVDVASYERMQQRIALLEGIARGE

XCV0057|CAJ21688.1| ATEPLAAAERDKEPILITQHGLPRAYVVDVASDERMQQRIALLEGIARGE

PXO_03336|ACD56820.1| IVEPP-------------------CR----VIYRLDGQRIVVVH-VLRSE

XOC_0151|ACD56820.1| IVEPP-------------------CR----VIYRLDGQRIVVVH-VLRSE

.* . . . ***.::. : *.*

XAC0080|AAM34972.1| MAVAEGRTLSHEQARHRMARWLK---------------------------

XCAW_00469|AGI06288.1| MAVAEGRTLSHEQARHRMARWLK---------------------------

XAC29_00405|AGH75617.1| MAVAEGRTLSHEQARHRMARWLK---------------------------

XCV0057|CAJ21688.1| MAVAQGRTLRHEQAQQHMARWLK---------------------------

PXO_03336|ACD56820.1| RALR----------KRRISRWKRLTKRRRSRPVGAACAPCCMDNASTPVV

XOC_0151|ACD56820.1| RALR----------KRRISRWKRLTKRRRSRPVGAACAPCCMDNASTPVV

*: :::::** :

XAC0080|AAM34972.1| -------

XCAW_00469|AGI06288.1| -------

XAC29_00405|AGH75617.1| -------

XCV0057|CAJ21688.1| -------

PXO_03336|ACD56820.1| NALSTPG

XOC_0151|ACD56820.1| NALSTPG

**5 - DOMAIN cd09881 - PIN_VapC-FitB**

**cd09881 - PIN_VapC-FitB (1)**

NUCLEOTIDE ALIGNMENTS:

XAC4314|21240774_5099881-5100135 ATGCCTTCTTTCACCGTCCGCAATATCCCAGACGACGTGCATCGCGCGAT

XAC29_21735|470469929_5079562-5079816 ATGCCTTCTTTCACCGTCCGCAATATCCCAGACGACGTGCATCGCGCGAT

XCAW_04683|471265562_5245825-5246079 ATGCCTTCTTTCACCGTCCGCAATATCCCAGACGACGTGCATCGCGCGAT

**************************************************

XAC4314|21240774_5099881-5100135 CCGCGCGCGGGCTGCACTTCATGGCCGCAGCACCGAAGCGGAGATCCGCG

XAC29_21735|470469929_5079562-5079816 CCGCGCGCGGGCTGCACTTCATGGCCGCAGCACCGAAGCGGAGATCCGCG

XCAW_04683|471265562_5245825-5246079 CCGCGCGCGGGCTGCACTTCATGGCCGCAGCACCGAAGCGGAGATCCGCG

**************************************************

XAC4314|21240774_5099881-5100135 CAATCCTTGAATTGGCGGCCAAACCCGCAGACCGGGTCAAACTCGGCTCA

XAC29_21735|470469929_5079562-5079816 CAATCCTTGAATTGGCGGCCAAACCCGCAGACCGGGTCAAACTCGGCTCA

XCAW_04683|471265562_5245825-5246079 CAATCCTTGAATTGGCGGCCAAACCCGCAGACCGGGTCAAACTCGGCTCA

**************************************************

XAC4314|21240774_5099881-5100135 CTGCTGGTGAGCATCGGCCGCGACGCCGGCCTGACCGCAAAAGAGGCCAA

XAC29_21735|470469929_5079562-5079816 CTGCTGGTGAGCATCGGCCGCGACGCCGGCCTGACCGCAAAAGAGGCCAA

XCAW_04683|471265562_5245825-5246079 CTGCTGGTGAGCATCGGCCGCGACGCCGGCCTGACCGCAAAAGAGGCCAA

**************************************************

XAC4314|21240774_5099881-5100135 TGCCTTCGACAAGCTGCGCGGCAAGGCGGCGATCAAGCCGATCGGTCTGA

XAC29_21735|470469929_5079562-5079816 TGCCTTCGACAAGCTGCGCGGCAAGGCGGCGATCAAGCCGATCGGTCTGA

XCAW_04683|471265562_5245825-5246079 TGCCTTCGACAAGCTGCGCGGCAAGGCGGCGATCAAGCCGATCGGTCTGA

**************************************************

XAC4314|21240774_5099881-5100135 AGTGA

XAC29_21735|470469929_5079562-5079816 AGTGA

XCAW_04683|471265562_5245825-5246079 AGTGA

*****

PROTEIN ALIGNMENTS:

XAC4314|AAM39144.1|plasmid MPSFTVRNIPDDVHRAIRARAALHGRSTEAEIRAILELAAKPADRVKLGS

XAC29_21735|AGH79728.1|plasmid MPSFTVRNIPDDVHRAIRARAALHGRSTEAEIRAILELAAKPADRVKLGS

XCAW_04683|AGI10441.1|plasmid MPSFTVRNIPDDVHRAIRARAALHGRSTEAEIRAILELAAKPADRVKLGS

**************************************************

XAC4314|AAM39144.1|plasmid LLVSIGRDAGLTAKEANAFDKLRGKAAIKPIGLK

XAC29_21735|AGH79728.1|plasmid LLVSIGRDAGLTAKEANAFDKLRGKAAIKPIGLK

XCAW_04683|AGI10441.1|plasmid LLVSIGRDAGLTAKEANAFDKLRGKAAIKPIGLK

**********************************

**cd09881 - PIN_VapC-FitB (2)**

NUCLEOTIDE ALIGNMENTS:

XAC4315|21240774_5100132-5100548 GTGATCCTGCTCGATACCAATGTCATCTCCGAACTTTGGCGGCCTCAGCC

XAC29_21740 GTGATCCTGCTCGATACCAATGTCATCTCCGAACTTTGGCGGCCTCAGCC

XCAW_04684|471265562_5246076-5246492 GTGATCCTGCTCGATACCAATGTCATCTCCGAACTTTGGCGGCCTCAGCC

**************************************************

XAC4315|21240774_5100132-5100548 TAATCCGCAGGTCGTCGCCTGGATCGATGCACAAGCTGTCGAGACACTGT

XAC29_21740 TAATCCGCAGGTCGTCGCCTGGATCGATGCACAAGCTGTCGAGACACTGT

XCAW_04684|471265562_5246076-5246492 TAATCCGCAGGTCGTCGCCTGGATCGATGCACAAGCTGTCGAGACACTGT

**************************************************

XAC4315|21240774_5100132-5100548 TCCTGTCGGTCGTGACGGTGGCTGAACTCCGTTTCGGTATTGCCGTGATG

XAC29_21740 TCCTGTCGGTCGTGACGGTGGCTGAACTCCGTTTCGGTATTGCCGTGATG

XCAW_04684|471265562_5246076-5246492 TCCTGTCGGTCGTGACGGTGGCTGAACTCCGTTTCGGTATTGCCGTGATG

**************************************************

XAC4315|21240774_5100132-5100548 CCCAAGGGCCGAAAGCGATCCACCTTGCATGCTCGGTTGGAGGGCGAGGT

XAC29_21740 CCCAAGGGCCGAAAGCGATCCACCTTGCATGCTCGGTTGGAGGGCGAGGT

XCAW_04684|471265562_5246076-5246492 CCCAAGGGCCGAAAGCGATCCACCTTGCATGCTCGGTTGGAGGGCGAGGT

**************************************************

XAC4315|21240774_5100132-5100548 ATTGCCACTGTTCGACGGGCGCCTGCTCGCATTCGATCTGGATGCATCAC

XAC29_21740 ATTGCCACTGTTCGACGGGCGCCTGCTCGCATTCGATCTGGATGCATCAC

XCAW_04684|471265562_5246076-5246492 ATTGCCACTGTTCGACGGGCGCCTGCTCGCATTCGATCTGGATGCATCAC

**************************************************

XAC4315|21240774_5100132-5100548 ACGCATTTGCGGCGCTTGCATCGAAAGCCAGAACCGCAGGACTCACACTC

XAC29_21740 ACGCATTTGCGGCGCTTGCATCGAAAGCCAGAACCGCAGGACTCACACTC

XCAW_04684|471265562_5246076-5246492 ACGCATTTGCGGCGCTTGCATCGAAAGCCAGAACCGCAGGACTCACACTC

**************************************************

XAC4315|21240774_5100132-5100548 GGGCGCGCCGATGCCTACATCGCTGCAACAGCAGCCGCGCAAGGATTGAC

XAC29_21740 GGGCGCGCCGATGCCTACATCGCTGCAACAGCAGCCGCGCAAGGATTGAC

XCAW_04684|471265562_5246076-5246492 GGGCGCGCCGATGCCTACATCGCTGCAACAGCAGCCGCGCAAGGATTGAC

**************************************************

XAC4315|21240774_5100132-5100548 CGTTGCCACTCGCGACACGGCACCATTCGAGGCGATGGCGTTAGACGTCA

XAC29_21740 CGTTGCCACTCGCGACACGGCACCATTCGAGGCGATGGCGTTAGACGTCA

XCAW_04684|471265562_5246076-5246492 CGTTGCCACTCGCGACACGGCACCATTCGAGGCGATGGCGTTAGACGTCA

**************************************************

XAC4315|21240774_5100132-5100548 TTAACCCTTGGAGTTGA

XAC29_21740 TTAACCCTTGGAGTTGA

XCAW_04684|471265562_5246076-5246492 TTAACCCTTGGAGTTGA

*****************

PROTEIN ALIGNMENTS:

XAC4315|AAM39145.1|plasmid MILLDTNVISELWRPQPNPQVVAWIDAQAVETLFLSVVTVAELRFGIAVM

XAC29_21740 MILLDTNVISELWRPQPNPQVVAWIDAQAVETLFLSVVTVAELRFGIAVM

XCAW_04684|AGI10442.1|Nucleic MILLDTNVISELWRPQPNPQVVAWIDAQAVETLFLSVVTVAELRFGIAVM

**************************************************

XAC4315|AAM39145.1|plasmid PKGRKRSTLHARLEGEVLPLFDGRLLAFDLDASHAFAALASKARTAGLTL

XAC29_21740 PKGRKRSTLHARLEGEVLPLFDGRLLAFDLDASHAFAALASKARTAGLTL

XCAW_04684|AGI10442.1|Nucleic PKGRKRSTLHARLEGEVLPLFDGRLLAFDLDASHAFAALASKARTAGLTL

**************************************************

XAC4315|AAM39145.1|plasmid GRADAYIAATAAAQGLTVATRDTAPFEAMALDVINPWS

XAC29_21740 GRADAYIAATAAAQGLTVATRDTAPFEAMALDVINPWS

XCAW_04684|AGI10442.1|Nucleic GRADAYIAATAAAQGLTVATRDTAPFEAMALDVINPWS

**************************************

**cd09881 - PIN_VapC-FitB (3)**

NUCLEOTIDE ALIGNMENTS:

XACb0059|21264228_c52028-51627 ATGAAGCGGTACATGCTTGATACCAACACCGTGAGCCATCTCGTCAAGAG

XCVd0102|78045309_c112087-111827 GTGAGCC-----AA----GTTGCCAAG-CTG--------TTCATCAATGG

XAC29_22429|470464772_c51053-50652 ATGAAGCGGTACATGCTTGATACCAACACCGTGAGCCATCTCGTCAAGAG

XCAW_b00031|471265493_29193-29594 ATGAAGCGGTACATGCTTGATACCAACACCGTGAGCCATCTCGTCAAGAG

*** * * * * **** * * ** **** *

XACb0059|21264228_c52028-51627 CCATCCGGCTGTCTCTCGACGGGTCATTGAAGTGCCTATGACTGCGCTAT

XCVd0102|78045309_c112087-111827 CCG-----CAGC---CAG----------GCAGTCCGCTTGCCTGCC--GC

XAC29_22429|470464772_c51053-50652 CCATCCGGCTGTCTCTCGACGGGTCATTGAAGTGCCTATGACTGCGCTAT

XCAW_b00031|471265493_29193-29594 CCATCCGGCTGTCTCTCGACGGGTCATTGAAGTGCCTATGACTGCGCTAT

** * * * * *** * ** ****

XACb0059|21264228_c52028-51627 GTATGTCCGCAATTACCGGTGGGGAGCTGATGTTCGGCTTGGCTAAGGTG

XCVd0102|78045309_c112087-111827 CTAT---CGC-TTTACCGAGAAAGAG---GTTTTC--ATTCGCCAAGAC-

XAC29_22429|470464772_c51053-50652 GTATGTCCGCAATTACCGGTGGGGAGCTGATGTTCGGCTTGGCTAAGGTG

XCAW_b00031|471265493_29193-29594 GTATGTCCGCAATTACCGGTGGGGAGCTGATGTTCGGCTTGGCTAAGGTG

*** *** ****** *** * *** ** ** ***

XACb0059|21264228_c52028-51627 CCAGACGCCAAACGCCTTCAGCAAGCCGTCATGGAACTCCTGCGGCGCGT

XCVd0102|78045309_c112087-111827 CCGGAGACCGG-TGACGTCA---TCCTGTCTCGGAAG-CCTGCAACG--T

XAC29_22429|470464772_c51053-50652 CCAGACGCCAAACGCCTTCAGCAAGCCGTCATGGAACTCCTGCGGCGCGT

XCAW_b00031|471265493_29193-29594 CCAGACGCCAAACGCCTTCAGCAAGCCGTCATGGAACTCCTGCGGCGCGT

** ** ** * * *** * *** **** ***** ** *

XACb0059|21264228_c52028-51627 GGATGTGCTGCCTTGGGATGGCGCGGTAATGGAGCGTTACGGAAGCGTAA

XCVd0102|78045309_c112087-111827 GGGAGGACTTCTTT---ATCGCA----------------------CTCAA

XAC29_22429|470464772_c51053-50652 GGATGTGCTGCCTTGGGATGGCGCGGTAATGGAGCGTTACGGAAGCGTAA

XCAW_b00031|471265493_29193-29594 GGATGTGCTGCCTTGGGATGGCGCGGTAATGGAGCGTTACGGAAGCGTAA

** * ** * ** ** ** * **

XACb0059|21264228_c52028-51627 GAGCGGACCTAGAAAAGCAAGGTAAAGCGCTTGGGTCGCTCGACATGCTT

XCVd0102|78045309_c112087-111827 GAGCGC----AGATGTGCCTGCTAATTT-----------TCTAGATGCCG

XAC29_22429|470464772_c51053-50652 GAGCGGACCTAGAAAAGCAAGGTAAAGCGCTTGGGTCGCTCGACATGCTT

XCAW_b00031|471265493_29193-29594 GAGCGGACCTAGAAAAGCAAGGTAAAGCGCTTGGGTCGCTCGACATGCTT

***** *** ** * *** ** * ****

XACb0059|21264228_c52028-51627 ATCGCAGCACATGCACTGGAAACGGATTCTGTCCTCGTTACCAATGACGC

XCVd0102|78045309_c112087-111827 -CC-GAG---------------CGCAATCAGGGCACG-----CAGGA---

XAC29_22429|470464772_c51053-50652 ATCGCAGCACATGCACTGGAAACGGATTCTGTCCTCGTTACCAATGACGC

XCAW_b00031|471265493_29193-29594 ATCGCAGCACATGCACTGGAAACGGATTCTGTCCTCGTTACCAATGACGC

* ** ** * ** * * ** * **

XACb0059|21264228_c52028-51627 CGCATTTAGTCGTGTTGTTGGTTTGACCGTGGAAGATTGGACACGCAGCT

XCVd0102|78045309_c112087-111827 ---------CCGTGATCC--TTTTG-C---GG--GAT-G-G--CGCGAAT

XAC29_22429|470464772_c51053-50652 CGCATTTAGTCGTGTTGTTGGTTTGACCGTGGAAGATTGGACACGCAGCT

XCAW_b00031|471265493_29193-29594 CGCATTTAGTCGTGTTGTTGGTTTGACCGTGGAAGATTGGACACGCAGCT

**** * **** * ** *** * *** *

XACb0059|21264228_c52028-51627 AA

XCVd0102|78045309_c112087-111827 GA

XAC29_22429|470464772_c51053-50652 AA

XCAW_b00031|471265493_29193-29594 AA

*

PROTEIN ALIGNMENTS:

XACb0059|AAM39305.1|virulence MKRY-MLDTNTVSHLVKSHPAVSRRVIEVPMTALCMSAITGGELMFGLAK

XCVd0102|CAJ19914.1|conserved MSQVAKLFINGRSQAVR---------------------------------

XAC29_22429|WP_011053002.1|MULTIS MKRY-MLDTNTVSHLVKSHPAVSRRVIEVPMTALCMSAITGGELMFGLAK

XCAW_b00031|AGI10551.1|virulence MKRY-MLDTNTVSHLVKSHPAVSRRVIEVPMTALCMSAITGGELMFGLAK

*.: * * *: *:

XACb0059|AAM39305.1|virulence VPDAKRLQQAVMELLRRVDVLPWDGAVMERYGSVRADLEKQGKALGSLDM

XCVd0102|CAJ19914.1|conserved LPAAYRFTEK--EVFIRQDPETGDV-ILSRKP------------ATWEDF

XAC29_22429|WP_011053002.1|MULTI VPDAKRLQQAVMELLRRVDVLPWDGAVMERYGSVRADLEKQGKALGSLDM

XCAW_b00031|AGI10551.1|virulence VPDAKRLQQAVMELLRRVDVLPWDGAVMERYGSVRADLEKQGKALGSLDM

:* * *: : *:: * * . * ::.* *:

XACb0059|AAM39305.1|virulence LIAAHALETDSVLVTNDAAFSRVVGLTVED----WTRS

XCVd0102|CAJ19914.1|conserved FIALKSADVPAN---FLDAAERNQGTQDRDPFAGWR-E

XAC29_22429|WP_011053002.1|MULT LIAAHALETDSVLVTNDAAFSRVVGLTVED----WTRS

XCAW_b00031|AGI10551.1|virulence LIAAHALETDSVLVTNDAAFSRVVGLTVED----WTRS

:** :: :. : * .* * .* * .

**6 - DOMAIN TIGR02606 - antidote_CC2985**

NUCLEOTIDE ALIGNMENTS:

XCC1034|21229478_1183923-1184216 ATGC---------------------------------CGCTGCCGGGAGG

XAC1140|21240774_1291803-1292063 TTGC---------------------------------CAT---CCGGAGG

XC_3212|66766352_c3856626-3856333 ATGC---------------------------------CGCTGCCGGGAGG

PXO_02722|188574270_c4511553-4511386 AT------------------------------------------------

XCR_1233|384425691_1248283-1248609 ATGCGCCAGTACGTCTCGCGCAGCGGCTTGCCGATGCCGCTGCCGGGAGG

XAC29_05725|470469929_1295567-1295809 AT------------------------------------------------

XCAW_01240|471265562_1379022-1379264 AT------------------------------------------------

*

XCC1034|21229478_1183923-1184216 CACACTTTGGCAATCTTTGCCATCGGGTCGCTGCATGGCCACCATGAACA

XAC1140|21240774_1291803-1292063 ------------------------------CGGTATGGCCACCATGAACA

+XC_3212|66766352_c3856626-3856333 CACACTTTGGCAATCTTTGCCATCGGGTCGCTGCATGGCCACCATGAACA

PXO_02722|188574270_c4511553-4511386 ------------------------------------GGCCACCATGAACA

XCR_1233|384425691_1248283-1248609 CACACTTTGGCAATCTTTGCCATCGGGTCGCTGCATGGCCACCATGAACA

XAC29_05725|470469929_1295567-1295809 ------------------------------------GGCCACCATGAACA

XCAW_01240|471265562_1379022-1379264 ------------------------------------GGCCACCATGAACA

**************

XCC1034|21229478_1183923-1184216 TCTCGCTCCCGGACGAGCTCAAGGAATTTGTCGACCAGCAGGTCCTGGAA

XAC1140|21240774_1291803-1292063 TTTCTTTGCCTGACGAGCTCAAGCAGTTCGTCGATCAGCAGGTGGCGGAG

XC_3212|66766352_c3856626-3856333 TCTCGCTCCCGGACGAGCTCAAGGAATTTGTCGACCAGCAGGTCCTGGAA

PXO_02722|188574270_c4511553-4511386 TTGCTTTGCCTGACGAGCTCCAGCAGTGCGTTGATCCGCAAGTGGCGGAG

XCR_1233|384425691_1248283-1248609 TCTCGCTCCCGGACGAGCTCAAGGAATTTGTCGACCAGCAGGTCCTGGAA

XAC29_05725|470469929_1295567-1295809 TTTCTTTGCCTGACGAGCTCAAGCAGTTCGTCGATCAGCAGGTGGCGGAG

XCAW_01240|471265562_1379022-1379264 TTTCTTTGCCTGACGAGCTCAAGCAGTTCGTCGATCAGCAGGTGGCGGAG

* * * ** ********* ** * * ** ** * *** ** ***

XCC1034|21229478_1183923-1184216 CATGCTTACGGCTCGAGCAGCGAATATCTGCGCGAGCTGATCCGTATGCA

XAC1140|21240774_1291803-1292063 CATGCTTACGGTTCCAGCAGCGAGTACTTACGTGAGTTGATTCGCAAGCA

XC_3212|66766352_c3856626-3856333 CATGCTTACGGCTCGAGCAGCGAATATCTGCGCGAGCTGATCCGTATGCA

PXO_02722|188574270_c4511553-4511386 CATGCTTACGGGCCCGGCAGCGGGTACTTGCGTGCGCTGATGCGCAAGCA

XCR_1233|384425691_1248283-1248609 CATGCTTACGGCTCGAGCAGCGAATATCTGCGCGAGCTGATCCGTATGCA

XAC29_05725|470469929_1295567-1295809 CATGCTTACGGTTCCAGCAGCGAGTACTTACGTGAGTTGATTCGCAAGCA

XCAW_01240|471265562_1379022-1379264 CATGCTTACGGTTCCAGCAGCGAGTACTTACGTGAGTTGATTCGCAAGCA

*********** * ****** ** * ** * * **** ** * ***

XCC1034|21229478_1183923-1184216 GCGTGATGCGCAAAGCCTGCGCGCGCTGTTGCTGGACGGCGCAGAGTCCG

XAC1140|21240774_1291803-1292063 ACGCGATATCGAACAGCTGCGTGGTGTGTTGCTTGGTGGAGCGAACTCCG

XC_3212|66766352_c3856626-3856333 GCGTGATGCGCAAAGCCTGCGCGCGCTGTTGCTGGACGGCGCAGAGTCCG

PXO_02722|188574270_c4511553-4511386 ACGCGATATTGAACAGCTGCGTGGCGTGTTGCTTGATGGAGCGAACTC--

XCR_1233|384425691_1248283-1248609 GCGTGATGCGCAAAGCCTGCGCGCGCTGTTGCTGGACGGCGCAGAGTCCG

XAC29_05725|470469929_1295567-1295809 ACGCGATATCGAACAGCTGCGTGGTGTGTTGCTTGGTGGAGCGAACTCCG

XCAW_01240|471265562_1379022-1379264 ACGCGATATCGAACAGCTGCGTGGTGTGTTGCTTGGTGGAGCGAACTCCG

** *** ** ***** * ******* * ** ** * **

XCC1034|21229478_1183923-1184216 GGCCTGCTGTTGCGATGGAGGCCGACTTCTTTGACAGCATGCGCGCGCGT

XAC1140|21240774_1291803-1292063 GACCGGCAGTGGCGGCCGAGGCTGGCTTCTTCAACGCGATGCAAGCGCGT

XC_3212|66766352_c3856626-3856333 GGCCTGCTGTTGCGATGGAGGCCGACTTCTTTGACAGCATGCGCGCGCGT

PXO_02722|188574270_c4511553-4511386 --------------------------------------------------

XCR_1233|384425691_1248283-1248609 GGCCTGCTGTTGCGATGGAGGCCGACTTCTTTGACAGCATGCGCGCGCGC

XAC29_05725|470469929_1295567-1295809 GACCGGCAGTGGCGGCCGAGGCTGGCTTCTTCAACGCGATGCAAGCGCGT

XCAW_01240|471265562_1379022-1379264 GACCGGCAGTGGCGGCCGAGGCTGGCTTCTTCAACGCGATGCAAGCGCGT

XCC1034|21229478_1183923-1184216 GCGCGTCAGCGCGCTGCCGGAAAATGA

XAC1140|21240774_1291803-1292063 GCCAACGCGCGTGCCGGCGAGCAGTGA

XC_3212|66766352_c3856626-3856333 GCGCGTCAGCGCGCTGCCGGAAAATGA

PXO_02722|188574270_c4511553-4511386 -----------------------CTGA

XCR_1233|384425691_1248283-1248609 GCGCATCAGCGCGCTGCCGGAAAATGA

XAC29_05725|470469929_1295567-1295809 GCCAACGCGCGTGCCGGCGAGCAGTGA

XCAW_01240|471265562_1379022-1379264 GCCAACGCGCGTGCCGGCGAGCAGTGA

***

PROTEIN ALIGNMENTS:

XCC1034|21112058|gb|AAM40333.1| -----------MPLPGGTLWQSLPSGRCMATMNISLPDELKEFVDQQVLE

XAC1140|21107279|gb|AAM36012.1| ----------------------MPSGGGMATMNISLPDELKQFVDQQVAE

XC_3212|66574846|gb|AAY50256.1| -----------MPLPGGTLWQSLPSGRCMATMNISLPDELKEFVDQQVLE

PXO_02722|188522999|gb|ACD60944.1| ----------------------------MATMNIALPDELQQCVDPQVAE

XCR_1233|341935995|gb|AEL06134.1| MRQYVSRSGLPMPLPGGTLWQSLPSGRCMATMNISLPDELKEFVDQQVLE

XAC29_05725|469766241|gb|AGH76658.1| ----------------------------MATMNISLPDELKQFVDQQVAE

XCAW_01240|471210292|gb|AGI07042.1| ----------------------------MATMNISLPDELKQFVDQQVAE

******:*****:: ** ** *

XCC1034|21112058|gb|AAM40333.1| HAYGSSSEYLRELIRMQRDAQSLRALLLDGAESGPAVAMEADFFDSMRAR

XAC1140|21107279|gb|AAM36012.1| HAYGSSSEYLRELIRKQRDIEQLRGVLLGGANSGPAVAAEAGFFNAMQAR

XC_3212|66574846|gb|AAY50256.1| HAYGSSSEYLRELIRMQRDAQSLRALLLDGAESGPAVAMEADFFDSMRAR

PXO_02722|188522999|gb|ACD60944.1| HAYGPGSGYLRALMRKQRDIEQLRGVLLDGANS-----------------

XCR_1233|341935995|gb|AEL06134.1| HAYGSSSEYLRELIRMQRDAQSLRALLLDGAESGPAVAMEADFFDSMRAR

XAC29_05725|469766241|gb|AGH76658.1| HAYGSSSEYLRELIRKQRDIEQLRGVLLGGANSGPAVAAEAGFFNAMQAR

XCAW_01240|471210292|gb|AGI07042.1| HAYGSSSEYLRELIRKQRDIEQLRGVLLGGANSGPAVAAEAGFFNAMQAR

****..* *** *:* *** :.**.:**.**:*

XCC1034|21112058|gb|AAM40333.1| ARQRAAGK

XAC1140|21107279|gb|AAM36012.1| ANARAGEQ

XC_3212|66574846|gb|AAY50256.1| ARQRAAGK

PXO_02722|188522999|gb|ACD60944.1| --------

XCR_1233|341935995|gb|AEL06134.1| AHQRAAGK

XAC29_05725|469766241|gb|AGH76658.1| ANARAGEQ

XCAW_01240|471210292|gb|AGI07042.1| ANARAGEQ

**7 - DOMAIN COG2336 - MazE**

NUCLEOTIDE ALIGNMENTS:

XAC2188|21240774_c2561791-2561552 GTGGCCATGCAAGTCGCGAAGTGGGGTAACAGCCTGGCGGTGCGCCTGCC

XAC29_11085|470469929_c2577562-2577329 AT------GCAAGTCGCGAAGTGGGGTAACAGCCTGGCGGTGCGCCTGCC

XCAW_02430|471265562_c2702497-2702264 AT------GCAAGTCGCGAAGTGGGGTAACAGCCTGGCGGTGCGCCTGCC

* ******************************************

XAC2188|21240774_c2561791-2561552 GTCCAGTCTGGTGGAAGCGCTGGAACTGCGCGAGGGCGACGACATCGAGA

XAC29_11085|470469929_c2577562-2577329 GTCCAGTCTGGTGGAAGCGCTGGAACTGCGCGAGGGCGACGACATCGAGA

XCAW_02430|471265562_c2702497-2702264 GTCCAGTCTGGTGGAAGCGCTGGAACTGCGCGAGGGCGACGACATCGAGA

**************************************************

XAC2188|21240774_c2561791-2561552 TCGTCGTCGATGATCCGCGATTGTTCGCGGTGCGCAAGAAGCCGGGTCCG

XAC29_11085|470469929_c2577562-2577329 TCGTCGTCGATGATCCGCGATTGTTCGCGGTGCGCAAGAAGCCGGGTCCG

XCAW_02430|471265562_c2702497-2702264 TCGTCGTCGATGATCCGCGATTGTTCGCGGTGCGCAAGAAGCCGGGTCCG

**************************************************

XAC2188|21240774_c2561791-2561552 GAGGCCATGTTGGAGCGGCTGCGTGCCTTCCGGGGCAAGTTGCCCGCAGA

XAC29_11085|470469929_c2577562-2577329 GAGGCCATGTTGGAGCGGCTGCGTGCCTTCCGGGGCAAGTTGCCCGCAGA

XCAW_02430|471265562_c2702497-2702264 GAGGCCATGTTGGAGCGGCTGCGTGCCTTCCGGGGCAAGTTGCCCGCAGA

**************************************************

XAC2188|21240774_c2561791-2561552 TTTCAAGTTCAACCGGGACGAGGCCAATGGCCGCGGGTAA

XAC29_11085|470469929_c2577562-2577329 TTTCAAGTTCAACCGGGACGAGGCCAATGGCCGCGGGTAA

XCAW_02430|471265562_c2702497-2702264 TTTCAAGTTCAACCGGGACGAGGCCAATGGCCGCGGGTAA

****************************************

PROTEIN ALIGNMENTS:

XAC2188|AAM37041.1|cell MAMQVAKWGNSLAVRLPSSLVEALELREGDDIEIVVDDPRLFAVRKKPGP

XAC29_11085|AGH77676.1|cell --MQVAKWGNSLAVRLPSSLVEALELREGDDIEIVVDDPRLFAVRKKPGP

XCAW_02430|AGI08214.1|Cell --MQVAKWGNSLAVRLPSSLVEALELREGDDIEIVVDDPRLFAVRKKPGP

************************************************

XAC2188|AAM37041.1|cell EAMLERLRAFRGKLPADFKFNRDEANGRG

XAC29_11085|AGH77676.1|cell EAMLERLRAFRGKLPADFKFNRDEANGRG

XCAW_02430|AGI08214.1|Cell EAMLERLRAFRGKLPADFKFNRDEANGRG

*****************************

**8 - DOMAIN cd09855 - PIN_VapC-Smg6-like**

NUCLEOTIDE ALIGNMENTS:

XAC2187|21240774_c2561544-2561152 GTG---------------------TTTCTCGACAGCAACGTGGTGCTCTA

XCAW_02429|471265562_c2702277-2701864 ATGGCCGCGGGTAAGGGCAAAGTGTTTCTCGACAGCAACGTGGTGCTCTA

XAC29_11080|470469929_c2577342-2576929 ATGGCCGCGGGTAAGGGCAAAGTGTTTCTCGACAGCAACGTGGTGCTCTA

** **************************

XAC2187|21240774_c2561544-2561152 CCTGCTGTCGGAGGATGCGGTGAAGGCGGACGGCGCGGAGGCATTGCTGC

XCAW_02429|471265562_c2702277-2701864 CCTGCTGTCGGAGGATGCGGTGAAGGCGGACGGCGCGGAGGCATTGCTGC

XAC29_11080|470469929_c2577342-2576929 CCTGCTGTCGGAGGATGCGGTGAAGGCGGACGGCGCGGAGGCATTGCTGC

**************************************************

XAC2187|21240774_c2561544-2561152 AGCGCAGGCCCGTCATCAGTGTGCAGGTGCTCAATGAAGTGACCCACGTC

XCAW_02429|471265562_c2702277-2701864 AGCGCAGGCCCGTCATCAGTGTGCAGGTGCTCAATGAAGTGACCCACGTC

XAC29_11080|470469929_c2577342-2576929 AGCGCAGGCCCGTCATCAGTGTGCAGGTGCTCAATGAAGTGACCCACGTC

**************************************************

XAC2187|21240774_c2561544-2561152 TGCGTTCGCAAGCTCAAGATGGGCTGGGACGAAGTCGGGCAGTTCCTTGC

XCAW_02429|471265562_c2702277-2701864 TGCGTTCGCAAGCTCAAGATGGGCTGGGACGAAGTCGGGCAGTTCCTTGC

XAC29_11080|470469929_c2577342-2576929 TGCGTTCGCAAGCTCAAGATGGGCTGGGACGAAGTCGGGCAGTTCCTTGC

**************************************************

XAC2187|21240774_c2561544-2561152 GTTGGTGCGATCGTTCTGCAAGATAGTGCCTCTGACGGTCGATGTTCACG

XCAW_02429|471265562_c2702277-2701864 GTTGGTGCGATCGTTCTGCAAGATAGTGCCTCTGACGGTCGATGTTCACG

XAC29_11080|470469929_c2577342-2576929 GTTGGTGCGATCGTTCTGCAAGATAGTGCCTCTGACGGTCGATGTTCACG

**************************************************

XAC2187|21240774_c2561544-2561152 ACCGTGCGCGCCAGCTGGCGGAGCGGCACCAATTGTCGTTCTACGACGCG

XCAW_02429|471265562_c2702277-2701864 ACCGTGCGCGCCAGCTGGCGGAGCGGCACCAATTGTCGTTCTACGACGCG

XAC29_11080|470469929_c2577342-2576929 ACCGTGCGCGCCAGCTGGCGGAGCGGCACCAATTGTCGTTCTACGACGCG

**************************************************

XAC2187|21240774_c2561544-2561152 TGCATCGTGGCGGCGGCCGCCATCGAAGGGTGCCAGACCCTCTACTCCGA

XCAW_02429|471265562_c2702277-2701864 TGCATCGTGGCGGCGGCCGCCATCGAAGGGTGCCAGACCCTCTACTCCGA

XAC29_11080|470469929_c2577342-2576929 TGCATCGTGGCGGCGGCCGCCATCGAAGGGTGCCAGACCCTCTACTCCGA

**************************************************

XAC2187|21240774_c2561544-2561152 AGACATGCACCACGGCCTCATCATCGAAGAGAGCCTGTCGATTCGGAACC

XCAW_02429|471265562_c2702277-2701864 AGACATGCACCACGGCCTCATCATCGAAGAGAGCCTGTCGATTCGGAACC

XAC29_11080|470469929_c2577342-2576929 AGACATGCACCACGGCCTCATCATCGAAGAGAGCCTGTCGATTCGGAACC

**************************************************

XAC2187|21240774_c2561544-2561152 CGTTCAACGTCTGA

XCAW_02429|471265562_c2702277-2701864 CGTTCAACGTCTGA

XAC29_11080|470469929_c2577342-2576929 CGTTCAACGTCTGA

**************

PROTEIN ALIGNMENTS:

XAC2187|AAM37040.1|conserved -------MFLDSNVVLYLLSEDAVKADGAEALLQRRPVISVQVLNEVTHV

XCAW_02429|AGI08213.1|Nucleic MAAGKGKVFLDSNVVLYLLSEDAVKADGAEALLQRRPVISVQVLNEVTHV

XAC29_11080|AGH77675.1|hypothetical MAAGKGKVFLDSNVVLYLLSEDAVKADGAEALLQRRPVISVQVLNEVTHV

:******************************************

XAC2187|AAM37040.1|conserved CVRKLKMGWDEVGQFLALVRSFCKIVPLTVDVHDRARQLAERHQLSFYDA

XCAW_02429|AGI08213.1|Nucleic CVRKLKMGWDEVGQFLALVRSFCKIVPLTVDVHDRARQLAERHQLSFYDA

XAC29_11080|AGH77675.1|hypothetical CVRKLKMGWDEVGQFLALVRSFCKIVPLTVDVHDRARQLAERHQLSFYDA

**************************************************

XAC2187|AAM37040.1|conserved CIVAAAAIEGCQTLYSEDMHHGLIIEESLSIRNPFNV

XCAW_02429|AGI08213.1|Nucleic CIVAAAAIEGCQTLYSEDMHHGLIIEESLSIRNPFNV

XAC29_11080|AGH77675.1|hypothetical CIVAAAAIEGCQTLYSEDMHHGLIIEESLSIRNPFNV

*************************************

**9 - DOMAIN cd09875 - PIN_VapC-FitB-like**

NUCLEOTIDE ALIGNMENTS:

XCC1862|21229478_2168934-2169341 ATGATCGCCATTGATTCATCGGTGCTGGTCGACCTGCTGGCC---GACAG

XAC1884|21240774_2188362-2188772 ATGATCGCCCTGGATTCCTCGGTCCTGCTGGACATCCTGATCGGCGACCC

XC_2327|66766352_c2813977-2813570 ATGATCGCCATTGATTCATCGGTGCTGGTCGACCTGCTGGCC---GACAG

XCV1926|78045556_2177809-2178219 ATGATCGCCCTGGATTCCTCGGTCCTGCTGGACATCCTGATCGGCGACCC

PXO_00062|188574270_1800052-1800462 ATGATCGCTCTGGATTCCTCGGTGCTGCTGGACATCCTGATCGGCGACCC

XALc_1351|285016821_1565459-1565866 GTGATCGCGATCGACTCCTCGGTGCTGGTGGATCTGCTGGCC---GACAG

XCR_2115|384425691_2179992-2180399 ATGATTGCCATTGATTCATCGGTGCTGGTCGACCTGCTGGCC---GACAG

XOC_2272|384417079_2266749-2267159 ATGATCGCTCTGGATTCCTCGGTGCTGCTGGACATCCTGATCGGCGACCC

XAC29_09510|470469929_2190524-2190934 ATGATCGCCCTGGATTCCTCGGTCCTGCTGGACATCCTGATCGGCGACCC

XCAW_02515|471265562_c2819853-2819443 ATGATCGCCCTGGATTCCTCGGTCCTGCTGGACATCCTGATCGGCGACCC

**** ** * ** ** ***** *** * ** * *** * ***

XCC1862|21229478_2168934-2169341 TGCCCAAGCAGACGCTGCCGAAGCCTGCCTGCGCCAATGCCTGAGCAC-C

XAC1884|21240774_2188362-2188772 GGTCTATGGCGAAGTGTCGGAGATCTGCAT-CGGCGACGCCCTCGCGCGC

XC_2327|66766352_c2813977-2813570 TGCCCAAGCAGACGCTGCCGAAGCCTGCCTGCGCCAATGCCTGAGCAC-C

XCV1926|78045556_2177809-2178219 GGTCTATGGCGAAGTGTCGGAGGTCTGCAT-CGGCGACGCGCTGGCGCGC

PXO_00062|188574270_1800052-1800462 GGTCTACGGCGAAGTGTCGGAAATCTGCAT-CGGCGACGCCCTGGCGCGC

XALc_1351|285016821_1565459-1565866 CCCGCAGGCCGATGCGGCCGAAGCCTGTCTGCGCCAGTGCCTGAGCAC-C

XCR_2115|384425691_2179992-2180399 CGCACAAGCCGACGCTGCCGAAGCCTGCCTGCGCCAATGCCTGAGCAC-C

XOC_2272|384417079_2266749-2267159 GGTCTACGGCGAAGTGTCGGAGATCTGCAT-CGGCGACGCCCTGGCGCGC

XAC29_09510|470469929_2190524-2190934 GGTCTATGGCGAAGTGTCGGAGATCTGCAT-CGGCGACGCCCTCGCGCGC

XCAW_02515|471265562_c2819853-2819443 GGTCTATGGCGAAGTGTCGGAGATCTGCAT-CGGCGACGCCCTCGCGCGC

* * ** * * ** *** * ** * ** ** * *

XCC1862|21229478_2168934-2169341 GGCCCGGTGGTGGTCTGCGACGTGGTGCTGGCCGAGGTCTGCAGCGCGCT

XAC1884|21240774_2188362-2188772 GACGAAGTGGTGGTCTGCGACGCGGTGGTGGCCGAGGTGCTG---GCCAT

XC_2327|66766352_c2813977-2813570 GGCCCGGTGGTGGTCTGCGACGTGGTGCTGGCCGAGGTCTGCAGCGCGCT

XCV1926|78045556_2177809-2178219 GACGAAGTGGTGGTCTGCGACGCGGTGGTGGCCGAGGTGCTG---GCCAT

PXO_00062|188574270_1800052-1800462 GACGAGGTCGTGGTTTGCGACGCCGTGGTGGCCGAAGTGCTG---GCCAT

XALc_1351|285016821_1565459-1565866 GGCCCGGTGGTGGTTTGCGGTATCGTCCTGGCCGAGGTCTGCGCGGCGCT

XCR_2115|384425691_2179992-2180399 GGCCCGGTGGTGGTCTGCGACGTGGTGCTGGCCGAGGTTTGCAGCGCGCT

XOC_2272|384417079_2266749-2267159 GACGAGGTCGTGGTTTGCGACGCCGTGGTGGCCGAAGTGCTG---GCCAT

XAC29_09510|470469929_2190524-2190934 GACGAAGTGGTGGTCTGCGACGCGGTGGTGGCCGAGGTGCTG---GCCAT

XCAW_02515|471265562_c2819853-2819443 GACGAAGTGGTGGTCTGCGACGCGGTGGTGGCCGAGGTGCTG---GCCAT

* * ** ***** **** ** ******* ** ** *

XCC1862|21229478_2168934-2169341 GCGCGATGG---CGCCGAAGCGCTGTCGGTGCTGGAAGACATGAGCATCC

XAC1884|21240774_2188362-2188772 GCTCGACACCCAGGTCGACCTGATGGAAACCCTGGCCTCGATCGGCGTGC

XC_2327|66766352_c2813977-2813570 GCGCGATGG---CGCCGAAGCGCTGTCGGTGCTGGAAGACATGAGCATCC

XCV1926|78045556_2177809-2178219 GCTCGACACCCAGGTCGACCTGATGGAAACCCTGGCCTCGATCGGCGTGC

PXO_00062|188574270_1800052-1800462 GCTCGACACCCAGGTGGACCTGATGGAAACCCTGGCCTCGATCGGCGTGC

XALc_1351|285016821_1565459-1565866 GCGCGATGG---CGCCGAGGCGCTGTCGGTGCTGGAAGAGATGAGCATCC

XCR_2115|384425691_2179992-2180399 GCGCGATGG---CGCCGAAGCGCTGTCGGTGCTGGAAGACATGAGCATCC

XOC_2272|384417079_2266749-2267159 GCTCGACACCCAGGTCGACCTGATGGAAACCCTGGCCTCGATCGGCGTGC

XAC29_09510|470469929_2190524-2190934 GCTCGACACCCAGGTCGACCTGATGGAAACCCTGGCCTCGATCGGCGTGC

XCAW_02515|471265562_c2819853-2819443 GCTCGACACCCAGGTCGACCTGATGGAAACCCTGGCCTCGATCGGCGTGC

** *** * ** * ** **** ** ** * *

XCC1862|21229478_2168934-2169341 GCTTCAACGCGCTGGAGGCCAAGTCGGCCTTGCGCGCCGGCGAGATGCAG

XAC1884|21240774_2188362-2188772 GCTACGAAGCCACCCAGGAAGCGGCGGCAGTGCGCGCCGGCCATATGAAC

XC_2327|66766352_c2813977-2813570 GCTTCAACGCGCTGGAGGCCAAGTCGGCCTTGCGCGCCGGCGAGATGCAG

XCV1926|78045556_2177809-2178219 GCTACGAGGCCACCCAGGAAGCGGCGGCAGTGCGCGCCGGCCATATGAAC

PXO_00062|188574270_1800052-1800462 GCTACGAGGCCACCCAGGAAGCGGCGGCGGTGCGCGCCGGTCATATGAAC

XALc_1351|285016821_1565459-1565866 GTTTCAATGCCCTGGAATCGAAGTCGGCGTTGCGCGCCGGCGAGATGCAG

XCR_2115|384425691_2179992-2180399 GCTTCAACGCGCTGGAGGCCAAGTCGGCCTTGCGCGCCGGCGAGATGCAG

XOC_2272|384417079_2266749-2267159 GCTACGAGGCCACCCAGGAAGCGGCGGCGGTGCGCGCCGGTCATATGAAC

XAC29_09510|470469929_2190524-2190934 GCTACGAAGCCACCCAGGAAGCGGCGGCAGTGCGCGCCGGCCATATGAAC

XCAW_02515|471265562_c2819853-2819443 GCTACGAAGCCACCCAGGAAGCGGCGGCAGTGCGCGCCGGCCATATGAAC

* * * * ** * * **** ********** * *** *

XCC1862|21229478_2168934-2169341 CGCCGCTTCCGTGCGCGCGGCGGCAAGCGCGAACGGGTGGTGGCGGACTT

XAC1884|21240774_2188362-2188772 AAGCGCTTTCGCGCGCGCGGCGGCAAGCGCGAGCGGGTGGTGGCCGACTT

XC_2327|66766352_c2813977-2813570 CGCCGCTTCCGTGCGCGCGGCGGCAAGCGCGAACGGGTGGTGGCGGACTT

XCV1926|78045556_2177809-2178219 AAGCGCTTTCGCGCGCGCGGCGGCAAGCGTGAGCGGGTGGTGGCCGACTT

PXO_00062|188574270_1800052-1800462 AAGCGCTTCCGCGCACGCGGCGGCAAGCGCGAGCGGGTGGTGGCGGATTT

XALc_1351|285016821_1565459-1565866 CGCCGGTTTCGTGCCCGCGGTGGTCAGCGCGAACGGGTGGTGGCCGACTT

XCR_2115|384425691_2179992-2180399 CGCCGCTTCCGTGCGCGCGGCGGCAAGCGCGAGCGGGTGGTGGCGGACTT

XOC_2272|384417079_2266749-2267159 AAGCGCTTCCGCGCACGCGGCGGCAAGCGCGAGCGGGTGGTCGCGGATTT

XAC29_09510|470469929_2190524-2190934 AAGCGCTTTCGCGCGCGCGGCGGCAAGCGCGAGCGGGTGGTGGCCGACTT

XCAW_02515|471265562_c2819853-2819443 AAGCGCTTTCGCGCGCGCGGCGGCAAGCGCGAGCGGGTGGTGGCCGACTT

** ** ** ** ***** ** **** ** ******** ** ** **

XCC1862|21229478_2168934-2169341 CCTGATCGGTGCGCACGCGATGCTGCAATGCGACGGCCTGATCACCCGCG

XAC1884|21240774_2188362-2188772 TCTGATCGGCGCCCACGCGATGCTCCAGTGCGACGGGTTGATCACCCGCG

XC_2327|66766352_c2813977-2813570 CCTGATCGGTGCGCACGCGATGCTGCAATGCGACGGCCTGATCACCCGCG

XCV1926|78045556_2177809-2178219 CCTGATCGGCGCCCACGCCATGCTCCAGTGCGACGGGTTGATCACCCGCG

PXO_00062|188574270_1800052-1800462 TTTGATCGGCGCCCACGCAATGCTCCAGTGCGATGGGTTGATCACCCGCG

XALc_1351|285016821_1565459-1565866 CCTGATCGGCGCCCATGCCATGCTCCAGTGCGATGCCTTGATTACCCGCG

XCR_2115|384425691_2179992-2180399 CCTGATCGGTGCGCACGCGATGCTGCAATGCGACGGCCTGATCACCCGCG

XOC_2272|384417079_2266749-2267159 TTTGATCGGCGCCCACGCAATGCTCCAGTGCGATGGGTTGATCACCCGCG

XAC29_09510|470469929_2190524-2190934 TCTGATCGGCGCCCACGCGATGCTCCAGTGCGACGGGTTGATCACCCGCG

XCAW_02515|471265562_c2819853-2819443 TCTGATCGGCGCCCACGCGATGCTCCAGTGCGACGGGTTGATCACCCGCG

******* ** ** ** ***** ** ***** * **** *******

XCC1862|21229478_2168934-2169341 ACGACAGTTTTTTCCGCGACTACTTCAAGGGCTTGAAGATCATCGTGCCC

XAC1884|21240774_2188362-2188772 ACGAGGGCTTCTTCCGCGATTACTTCAAGGGTTTGAAGATCGTCGTTCCC

XC_2327|66766352_c2813977-2813570 ACGACAGTTTTTTCCGCGACTACTTCAAGGGCTTGAAGATCATCGTGCCC

XCV1926|78045556_2177809-2178219 ACGAGGGCTTCTTCCGCGATTACTTCAAGGGTTTGAAGATCATCGTTCCC

PXO_00062|188574270_1800052-1800462 ACGAGGGCTTCTTCCGCGATTACTTCAAGGGCTTGAAGATTACCGTTCCC

XALc_1351|285016821_1565459-1565866 ACGATGGCTTTTTCCGCGACTACTTCAAAGGTCTGAAGATCATCGTCCCC

XCR_2115|384425691_2179992-2180399 ACGACAGTTTTTTCCGCGACTACTTCAAGGGCTTGAAGATCATCGTGCCC

XOC_2272|384417079_2266749-2267159 ACGAGGGCTTCTTCCGCGATTACTTCAAGGGCTTGAAGATCACCGTCCCC

XAC29_09510|470469929_2190524-2190934 ACGAGGGCTTCTTCCGCGATTACTTCAAGGGTTTGAAGATCGTCGTTCCC

XCAW_02515|471265562_c2819853-2819443 ACGAGGGCTTCTTCCGCGATTACTTCAAGGGTTTGAAGATCGTCGTTCCC

**** * ** ******** ******** ** ******* *** ***

XCC1862|21229478_2168934-2169341 AAGCCCGCTGCCTGA

XAC1884|21240774_2188362-2188772 AAACCCGCCCCCTGA

XC_2327|66766352_c2813977-2813570 AAGCCCGCTGCCTGA

XCV1926|78045556_2177809-2178219 AAACCCGCACCCTGA

PXO_00062|188574270_1800052-1800462 AAGCCCGTTGCCTGA

XALc_1351|285016821_1565459-1565866 AGACCTGCGGCCTGA

XCR_2115|384425691_2179992-2180399 AAGCCCGCTGCCTGA

XOC_2272|384417079_2266749-2267159 AAGCCCGTTGCCTGA

XAC29_09510|470469929_2190524-2190934 AAACCCGCCCCCTGA

XCAW_02515|471265562_c2819853-2819443 AAACCCGCCCCCTGA

* ** * *****

PROTEIN ALIGNMENTS:

XCC1862|AAM41151.1|conserved MIAIDSSVLVDLL-ADSAQADAAEACLRQCLSTGPVVVCDVVLAEVCSAL

XAC1884|AAM36746.1|conserved MIALDSSVLLDILIGDPVYGEVSEICIGDALARDEVVVCDAVVAEVLAML

XC_2327|AAY49380.1|conserved MIAIDSSVLVDLL-ADSAQADAAEACLRQCLSTGPVVVCDVVLAEVCSAL

XCV1926|CAJ23603.1|conserved MIALDSSVLLDILIGDPVYGEVSEVCIGDALARDEVVVCDAVVAEVLAML

PXO_00062|ACD58400.1|PIN MIALDSSVLLDILIGDPVYGEVSEICIGDALARDEVVVCDAVVAEVLAML

XALc_1351|CBA15858.1|hypothetical MIAIDSSVLVDLL-ADSPQADAAEACLRQCLSTGPVVVCGIVLAEVCAAL

XCR_2115|AEL07002.1|PIN MIAIDSSVLVDLL-ADSAQADAAEACLRQCLSTGPVVVCDVVLAEVCSAL

XOC_2272|AEQ96409.1|PIN MIALDSSVLLDILIGDPVYGEVSEICIGDALARDEVVVCDAVVAEVLAML

XAC29_09510|AGH77373.1|hypothetical MIALDSSVLLDILIGDPVYGEVSEICIGDALARDEVVVCDAVVAEVLAML

XCAW_02515|AGI08298.1|Nucleic MIALDSSVLLDILIGDPVYGEVSEICIGDALARDEVVVCDAVVAEVLAML

***:*****:*:* .*. .:.:* *: :.*: . ****. *:*** : *

XCC1862|AAM41151.1|conserved RDGAEALSVLEDMSIRFNALEAKSALRAGEMQRRFRARGGKRERVVADFL

XAC1884|AAM36746.1|conserved DTQVDLMETLASIGVRYEATQEAAAVRAGHMNKRFRARGGKRERVVADFL

XC_2327|AAY49380.1|conserved RDGAEALSVLEDMSIRFNALEAKSALRAGEMQRRFRARGGKRERVVADFL

XCV1926|CAJ23603.1|conserved DTQVDLMETLASIGVRYEATQEAAAVRAGHMNKRFRARGGKRERVVADFL

PXO_00062|ACD58400.1|PIN DTQVDLMETLASIGVRYEATQEAAAVRAGHMNKRFRARGGKRERVVADFL

XALc_1351|CBA15858.1|hypothetical RDGAEALSVLEEMSIRFNALESKSALRAGEMQRRFRARGGQRERVVADFL

XCR_2115|AEL07002.1|PIN RDGAEALSVLEDMSIRFNALEAKSALRAGEMQRRFRARGGKRERVVADFL

XOC_2272|AEQ96409.1|PIN DTQVDLMETLASIGVRYEATQEAAAVRAGHMNKRFRARGGKRERVVADFL

XAC29_09510|AGH77373.1|hypothetical DTQVDLMETLASIGVRYEATQEAAAVRAGHMNKRFRARGGKRERVVADFL

XCAW_02515|AGI08298.1|Nucleic DTQVDLMETLASIGVRYEATQEAAAVRAGHMNKRFRARGGKRERVVADFL

.: :..* .:.:*::* : :*:***.*::*******:*********

XCC1862|AAM41151.1|conserved IGAHAMLQCDGLITRDDSFFRDYFKGLKIIVPKPAA

XAC1884|AAM36746.1|conserved IGAHAMLQCDGLITRDEGFFRDYFKGLKIVVPKPAP

XC_2327|AAY49380.1|conserved IGAHAMLQCDGLITRDDSFFRDYFKGLKIIVPKPAA

XCV1926|CAJ23603.1|conserved IGAHAMLQCDGLITRDEGFFRDYFKGLKIIVPKPAP

PXO_00062|ACD58400.1|PIN IGAHAMLQCDGLITRDEGFFRDYFKGLKITVPKPVA

XALc_1351|CBA15858.1|hypothetical IGAHAMLQCDALITRDDGFFRDYFKGLKIIVPRPAA

XCR_2115|AEL07002.1|PIN IGAHAMLQCDGLITRDDSFFRDYFKGLKIIVPKPAA

XOC_2272|AEQ96409.1|PIN IGAHAMLQCDGLITRDEGFFRDYFKGLKITVPKPVA

XAC29_09510|AGH77373.1|hypothetical IGAHAMLQCDGLITRDEGFFRDYFKGLKIVVPKPAP

XCAW_02515|AGI08298.1|Nucleic IGAHAMLQCDGLITRDEGFFRDYFKGLKIVVPKPAP

**********.*****:.*********** **:*..

**10 - DOMAIN smart00966 - SpoVT/AbrB**

NUCLEOTIDE ALIGNMENTS:

XCC1861|21229478_2168680-2168937 ATGGAAGCCACTGTTGCCGAACGCGGTCAGATCACGCTGCCCAAGGCGGT

XAC1883|21240774_2188117-2188365 ATGGAAGCCACCGTTGCCGAACGCGGACAGATCACGCTGCCAAAGGCCGT

XC_2328|66766352_c2814231-2813974 ATGGAAGCCACTGTTGCCGAACGCGGTCAGATCACGCTGCCCAAGGCGGT

XCV1925|78045556_2177564-2177812 ATGGAAGCCACCGTTGCCGAACGCGGACAGATCACGCTGCCAAAGGCCGT

PXO_00063|188574270_1799807-1800055 ATGGAAGCCACCGTTGCCGAACGCGGACAGATCACGCTGCCAAAAGCCGT

XALc_1350|285016821_1565205-1565462 ATGGAAGCCATCGTCGCCGAGCGTGGACAGATCACCTTGCCCAAAGCCGT

XCR_2114|384425691_2179738-2179995 ATGGAAGCCACTGTTGCCGAACGCGGCCAGATCACGCTGCCCAAGGCGGT

XOC_2271|384417079_2266504-2266752 ATGGAAGCCACCGTTGCCGAACGCGGACAGATCACGCTGCCAAAGGCCGT

XAC29_09505|470469929_2190279-2190527 ATGGAAGCCACCGTTGCCGAACGCGGACAGATCACGCTGCCAAAGGCCGT

XCAW_02516|471265562_c2820098-2819850 ATGGAAGCCACCGTTGCCGAACGCGGACAGATCACGCTGCCAAAGGCCGT

********** ** ***** ** ** ******** **** ** ** **

XCC1861|21229478_2168680-2168937 GCGCGATGCGCTGGGGCTGACCAAGGGCACCACGCTCAAGGTCGAGCTGG

XAC1883|21240774_2188117-2188365 GCGCGATGCCCTGGGCCTGACCAAGGGCACCACGTTGAAGATCGAGCTCG

XC_2328|66766352_c2814231-2813974 GCGCGATGCGCTGGGGCTGACCAAGGGCACCACGCTCAAGGTCGAGCTGG

XCV1925|78045556_2177564-2177812 GCGCGATGCCCTGGGCCTGACCAAGGGCACCACGTTGAAGATCGAGCTCG

PXO_00063|188574270_1799807-1800055 GCGCGATGCTCTGGGTCTGACCAAGGGCACCACTCTCAAGATCGAACTCG

XALc_1350|285016821_1565205-1565462 GCGCGATGCCTTGGGCTTGAGCAAGGGCACGGTGCTGAAAGTGGAGTTGG

XCR_2114|384425691_2179738-2179995 GCGCGACGCGCTGGGCCTGACCAAGGGCACCACGCTCAAGGTCGAGCTGG

XOC_2271|384417079_2266504-2266752 GCGCGATGCCCTGGGCCTGACCAAGGGCACCACCCTCAAGATCGAACTCG

XAC29_09505|470469929_2190279-2190527 GCGCGATGCCCTGGGCCTGACCAAGGGCACCACGTTGAAGATCGAGCTCG

XCAW_02516|471265562_c2820098-2819850 GCGCGATGCCCTGGGCCTGACCAAGGGCACCACGTTGAAGATCGAGCTCG

****** ** **** *** ********* * ** * ** * *

XCC1861|21229478_2168680-2168937 AAGGCGGCCGCATCATTCTGCGCAAGAGTGTTGACGATGCCATTTCACG-

XAC1883|21240774_2188117-2188365 ACGGCGGGCGCATCATCCTGCGCAAGGACGTCAGTGAGGCGCT-GCGCAA

XC_2328|66766352_c2814231-2813974 AAGGCGGCCGCATCATTCTGCGCAAGAGTGTTGACGATGCCATTTCACG-

XCV1925|78045556_2177564-2177812 ACGGCGGGCGCATCATCCTGCGCAAGGACGTCAGCGAGGCGCT-GCGCAA

PXO_00063|188574270_1799807-1800055 ATGGTGGCCGCATCATCTTGCGTAAAGATGTCAGCGAGGCGCT-ACGCAA

XALc_1350|285016821_1565205-1565462 ACGGTGGCCGCATCATCCTGCGCAAGAGCGTGGACGATGCGATCTCGCG-

XCR_2114|384425691_2179738-2179995 AAGGCGGCCGCATCATTCTGCGCAAGAGTGTTGACGATGCCATTTCACG-

XOC_2271|384417079_2266504-2266752 ATGGTGGCCGCATCATCTTGCGTAAAGATGTCAGCGAGGCGTT-ACGCAA

XAC29_09505|470469929_2190279-2190527 ACGGCGGGCGCATCATCCTGCGCAAGGACGTCAGTGAGGCGCT-GCGCAA

XCAW_02516|471265562_c2820098-2819850 ACGGCGGGCGCATCATCCTGCGCAAGGACGTCAGTGAGGCGCT-GCGCAA

* ** ** ******** **** ** ** ** ** * * *

XCC1861|21229478_2168680-2168937 TGCACGCGGCCGCTTCAAGCTG---GACGGCTTTGCCAGCACCGACGATG

XAC1883|21240774_2188117-2188365 GGTGCGCGGCAAGTTCAAGCTGGTCGACGGCCTGACCAGCACAGATGCGG

XC_2328|66766352_c2814231-2813974 TGCACGCGGCCGCTTCAAGCTG---GACGGCTTTGCCAGCACCGACGATG

XCV1925|78045556_2177564-2177812 GGTGCGCGGCAAGTTCAAGCTGGTCGACGGCCTGACCAGCACCGATGCGG

PXO_00063|188574270_1799807-1800055 GGTGCGCGGCAAGTTCAAGCTCGTCGATGGCCTGACCAGCACCGATGCCG

XALc_1350|285016821_1565205-1565462 TGCGCGTGGCCGCTTCAAGCTC---GATGGCTTCGCCAGCACCGATGAGG

XCR_2114|384425691_2179738-2179995 CGCACGCGGCCGCTTCAAGCTG---GACGGCTTTGCCAGCACCGACGATG

XOC_2271|384417079_2266504-2266752 GGTGCGCGGCAAGTTCAAGCTGGTCGATGGCCTGACCAGCACCGATGCCG

XAC29_09505|470469929_2190279-2190527 GGTGCGCGGCAAGTTCAAGCTGGTCGACGGCCTGACCAGCACAGATGCGG

XCAW_02516|471265562_c2820098-2819850 GGTGCGCGGCAAGTTCAAGCTGGTCGACGGCCTGACCAGCACAGATGCGG

* ** *** ******** ** *** * ******* ** * *

XCC1861|21229478_2168680-2168937 CGATGCGTGCCATCCGTGGGCGTGCGCCGGGCGATCCGCTCGATCCCGAA

XAC1883|21240774_2188117-2188365 CGATGCGCGTGATCCGTGGCCGCGCCCCCGGAGACCCGTTCGAC------

XC_2328|66766352_c2814231-2813974 CGATGCGTGCCATCCGTGGGCGTGCGCCGGGCGATCCGCTCGATCCCGAA

XCV1925|78045556_2177564-2177812 CCATGCGCGTGATCCGTGGCCGCGCGCCCGGGGATCCGTTCGAC------

PXO_00063|188574270_1799807-1800055 CCATGCGCGTCATCCGTGGCCGCGCCCCCGGAGACCCGTTCGAC------

XALc_1350|285016821_1565205-1565462 CGATGCGGGCGATCCGTGGCCGTGCGCCTGGCGATCCGTTCGAGTCGGAC

XCR_2114|384425691_2179738-2179995 CGATGCGTGCCATCCGTGGGCGTGCGCCGGGCGATCCGCTCGATCCCGAA

XOC_2271|384417079_2266504-2266752 CCATGCGCGTCATCCGTGGCCGCGCCCCCGGAGACCCGTTCGAT------

XAC29_09505|470469929_2190279-2190527 CGATGCGCGTGATCCGTGGCCGCGCCCCCGGAGACCCGTTCGAC------

XCAW_02516|471265562_c2820098-2819850 CGATGCGCGTGATCCGTGGCCGCGCCCCCGGAGACCCGTTCGAC------

* ***** * ******** ** ** ** ** ** *** ****

XCC1861|21229478_2168680-2168937 GCCGGCGCATGA

XAC1883|21240774_2188117-2188365 ------CCATGA

XC_2328|66766352_c2814231-2813974 GCCGGCGCATGA

XCV1925|78045556_2177564-2177812 ------CCATGA

PXO_00063|188574270_1799807-1800055 ------CCATGA

XALc_1350|285016821_1565205-1565462 GCCACGGCGTGA

XCR_2114|384425691_2179738-2179995 GCCGGCGCATGA

XOC_2271|384417079_2266504-2266752 ------CCATGA

XAC29_09505|470469929_2190279-2190527 ------CCATGA

XCAW_02516|471265562_c2820098-2819850 ------CCATGA

* ***

PROTEIN ALIGNMENTS:

XCC1861|AAM41150.1|conserved MEATVAERGQITLPKAVRDALGLTKGTTLKVELEGGRIILRKSVDDAISR

XAC1883|AAM36745.1|conserved MEATVAERGQITLPKAVRDALGLTKGTTLKIELDGGRIILRKDVSEALRK

XC_2328|AAY49381.1|conserved MEATVAERGQITLPKAVRDALGLTKGTTLKVELEGGRIILRKSVDDAISR

XCV1925|CAJ23602.1|conserved MEATVAERGQITLPKAVRDALGLTKGTTLKIELDGGRIILRKDVSEALRK

PXO_00063|ACD58399.1|SpoVT MEATVAERGQITLPKAVRDALGLTKGTTLKIELDGGRIILRKDVSEALRK

XALc_1350|CBA15857.1|conserved MEAIVAERGQITLPKAVRDALGLSKGTVLKVELDGGRIILRKSVDDAISR

XCR_2114|AEL07001.1|transcriptional MEATVAERGQITLPKAVRDALGLTKGTTLKVELEGGRIILRKSVDDAISR

XOC_2271|AEQ96408.1|SpoVT MEATVAERGQITLPKAVRDALGLTKGTTLKIELDGGRIILRKDVSEALRK

XAC29_09505|AGH77372.1|hypothetical MEATVAERGQITLPKAVRDALGLTKGTTLKIELDGGRIILRKDVSEALRK

XCAW_02516|AGI08299.1|Hypothetical MEATVAERGQITLPKAVRDALGLTKGTTLKIELDGGRIILRKDVSEALRK

*** *******************:***.**:**:********.*.:*: :

XCC1861|AAM41150.1|conserved ARGRFKL-DGFASTDDAMRAIRGRAPGDPLDPEAGA

XAC1883|AAM36745.1|conserved VRGKFKLVDGLTSTDAAMRVIRGRAPGDPFDP----

XC_2328|AAY49381.1|conserved ARGRFKL-DGFASTDDAMRAIRGRAPGDPLDPEAGA

XCV1925|CAJ23602.1|conserved VRGKFKLVDGLTSTDAAMRVIRGRAPGDPFDP----

PXO_00063|ACD58399.1|SpoVT VRGKFKLVDGLTSTDAAMRVIRGRAPGDPFDP----

XALc_1350|CBA15857.1|conserved ARGRFKL-DGFASTDEAMRAIRGRAPGDPFESDATA

XCR_2114|AEL07001.1|transcriptional ARGRFKL-DGFASTDDAMRAIRGRAPGDPLDPEAGA

XOC_2271|AEQ96408.1|SpoVT VRGKFKLVDGLTSTDAAMRVIRGRAPGDPFDP----

XAC29_09505||AGH77372.1|hypothetical VRGKFKLVDGLTSTDAAMRVIRGRAPGDPFDP----

XCAW_02516|AGI08299.1|Hypothetical VRGKFKLVDGLTSTDAAMRVIRGRAPGDPFDP----

.**:*** **::*** ***.*********::.

**11 - DOMAIN COG3654 - doc**

NUCLEOTIDE ALIGNMENTS:

XAC1194|21240774_c1361960-1361523 GTGAACACGCGCATGCTGGTCTGGGTTACGCATGCGCTTGCATTGGCCAT

XCV1221|78045556_c1368018-1367593 ATGCT------------GGTCTGGATCACGCACGCACTTGCATTGGCGAT

PXO_02086|188574270_3818774-3819199 ATGAT------------CATCTGGATCGAGCGGCCACTTGTCTTGGCTAT

XOC_1245|384417079_c1214853-1214428 ATGAT------------CATCTGGATCGAGCGGCCACTTGTCTTGGCTAT

XAC29_05995|384417079_c1214853-1214428 ATGAT------------CATCTGGATCGAGCGGCCACTTGTCTTGGCTAT

XCAW_01295|471265562_c1449145-1448720 ATGCT------------GGTCTGGGTTACGCATGCGCTTGCATTGGCCAT

** ***** * ** * **** ***** **

XAC1194|21240774_c1361960-1361523 CCACGAACGCCAATTGAGCGAGCACGGGGGCGCAAGCGGAGTGCGCGACG

XCV1221|78045556_c1368018-1367593 CCACGAACGTCAATTGAGCGAGCACGGCGGCGCAAGCGGAGTGCGCGACG

PXO_02086|188574270_3818774-3819199 TCACGACCGGCAGCTGGCCGAACATGGTGGCGGGAGCGGTGTGCGCGACG

XOC_1245|384417079_c1214853-1214428 TCACGACCGGCAGCTGGCCGAACATGGTGGCGGGAGCGGTGTGCGCGACG

XAC29_05995|384417079_c1214853-1214428 TCACGACCGGCAGCTGGCCGAACATGGTGGCGGGAGCGGTGTGCGCGACG

XCAW_01295|471265562_c1449145-1448720 CCACGAACGCCAATTGAGCGAGCACGGGGGCGCAAGCGGAGTGCGCGACG

***** ** ** ** *** ** ** **** ***** **********

XAC1194|21240774_c1361960-1361523 AAGCGCTACTGGATTCGGCACTTGCACGCCCGCAACAGCTGTTTTCGTAT

XCV1221|78045556_c1368018-1367593 AGGCACTCTTGGCCTCGGCACTGGCACGCCCGCAGCAACTGTTTTCCTAC

PXO_02086|188574270_3818774-3819199 ATGCGCTGCTCGACTCAGCGCTTGCCAGACCTAAACAACTGTCGGCATAT

XOC_1245|384417079_c1214853-1214428 ATGCGCTGCTCGACTCAGCGCTTGCCAGACCTAAACAACTGTCGGCATAT

XAC29_05995|384417079_c1214853-1214428 ATGCGCTGCTCGACTCAGCGCTTGCCAGACCTAAACAACTGTCGGCATAT

XCAW_01295|471265562_c1449145-1448720 AAGCGCTACTGGATTCGGCACTTGCACGCCCGCAACAGCTGTTTTCGTAT

* ** ** * * ** ** ** ** * ** * ** **** * **

XAC1194|21240774_c1361960-1361523 GGCGACCCTCCACCCGATCTGGTTGGGCTGACGGCCAGCCTGGCTTATGG

XCV1221|78045556_c1368018-1367593 GGCGATCCTCCACCCGATCTTGTAGAGCTGACGGCCAGCCTGGCCTACGG

PXO_02086|188574270_3818774-3819199 GGCGACCCATCTCCGGACTTGGCGGCGCTAGCAGCAAGCCTGGCGTATGG

XOC_1245|384417079_c1214853-1214428 GGCGACCCATCTGCGGACTTGGCGGCGCTAGCAGCAAGCCTGGCGTATGG

XAC29_05995|384417079_c1214853-1214428 GGCGACCCATCTGCGGACTTGGCGGCGCTAGCAGCAAGCCTGGCGTATGG

XCAW_01295|471265562_c1449145-1448720 GGCGACCCTCCACCCGATCTGGTTGGGCTGACGGCCAGCCTGGCTTATGG

***** ** * * ** * * * *** * ** ******** ** **

XAC1194|21240774_c1361960-1361523 GCTCGCGCGCAATCACCCATTCGTGGATGGCAACAAACGAACTGCCCACG

XCV1221|78045556_c1368018-1367593 GTTCGCGCGCAATCATCCGTTCGTAGATGGCAACAAACGGACTGCCCATG

PXO_02086|188574270_3818774-3819199 CCTGGCCCGTCATCATCCATTTGTCGACGGCAACAAGCGCACCGCAGCGG

XOC_1245|384417079_c1214853-1214428 CCTGGCCCGTCATCATCCATTTGTCGACGGCAACAAGCGCACCGCAGCGG

XAC29_05995|384417079_c1214853-1214428 CCTGGCCCGTCATCATCCATTTGTCGACGGCAACAAGCGCACCGCAGCGG

XCAW_01295|471265562_c1449145-1448720 GCTCGCGCGCAATCACCCATTCGTGGATGGCAACAAACGAACTGCCCACG

* ** ** **** ** ** ** ** ******** ** ** ** *

XAC1194|21240774_c1361960-1361523 TGTGCTACCGAGTCTTCTTGCTGCTCAACGGTGCCGAGCTCATCGCCTCG

XCV1221|78045556_c1368018-1367593 TGTGCTATCGGGTTTTTTTGCTACTCAACGGCGCCGAACTCATCGCTTCG

PXO_02086|188574270_3818774-3819199 TGGCCTGCGAAACGTTCATCGTGCTCAATGGTGCCA-CCTTGCTGGCAGG

XOC_1245|384417079_c1214853-1214428 TGGCCTGCGAAACGTTCATCGTGCTCAATGGTGCCA-CCTTGCTGGCAGG

XAC29_05995|384417079_c1214853-1214428 TGGCCTGCGAAACGTTCATCGTGCTCAATGGTGCCA-CCTTGCTGGCAGG

XCAW_01295|471265562_c1449145-1448720 TGTGCTACCGAGTCTTCTTGCTGCTCAACGGTGCCGAGCTCATCGCCTCG

** ** ** * * ***** ** *** ** * *

XAC1194|21240774_c1361960-1361523 C-AGGAAGAAAAATACGTTGCCATGATGAGGCTGGCCGATGGCGCGTGGA

XCV1221|78045556_c1368018-1367593 C-AGGAAGAGAAATACGTTGCCATGATGGGCCTGGCCGATGGCGCGTGGA

PXO_02086|188574270_3818774-3819199 CGACCTGGAGCTCTACGGTTTCTATATCGGCCTGGCAGATGGTTCGCTCG

XOC_1245|384417079_c1214853-1214428 CGACCTGGGGCTCTACGGTTTCTATATCGGCCTGGCAGATGGTTCGCTCG

XAC29_05995|384417079_c1214853-1214428 CGACCTGGGGCTCTACGGTTTCTATATCGGCCTGGCAGATGGTTCGCTCG

XCAW_01295|471265562_c1449145-1448720 C-AGGAAGAAAAATACGTTGCCATGATGAGGCTGGCCGATGGCGCGTGGA

* * * **** * * ** * ***** ***** **

XAC1194|21240774_c1361960-1361523 GCGAAGCGACATTCGCCCAGTGGCTACGGCCGCGCGTGCGATTGCGT-GC

XCV1221|78045556_c1368018-1367593 GCGAAGCGACGTTTGCGCAATGGCTGCGACCTCGCGTGCGTTTGCGC-GC

PXO_02086|188574270_3818774-3819199 ATGAGGCAGCGTGTGCAGGATGGCTACGTCCACGGTTGCAATCCCGGCAC

XOC_1245|384417079_c1214853-1214428 ATGAGGCAGCGTGTGCAGGATGGCTACGTCCACGGTTGCAATCCCGGCAC

XAC29_05995|384417079_c1214853-1214428 ATGAGGCAGCGTGTGCAGGATGGCTACGTCCACGGTTGCAATCCCGGCAC

XCAW_01295|471265562_c1449145-1448720 GCGAAGCGACATTCGCCCAGTGGCTACGGCCGCGCGTGCGATTGCGT-GC

** ** * * ** ***** ** ** ** *** * ** *

XAC1194|21240774_c1361960-1361523 AGACACGCATGTGCACGAACCGCAAGGTCATTACGGCTGA

XCV1221|78045556_c1368018-1367593 GGACACGCATGTACACGAACCGCAGGGCCACTACGGCTGA

PXO_02086|188574270_3818774-3819199 CAGC-AGCGCGTGCATGAGTCAGCACCGGACTACGGTTGA

XOC_1245|384417079_c1214853-1214428 CAGC-AGCGCGTGCATGAGTCAGCACCGGACTACGGTTGA

XAC29_05995|384417079_c1214853-1214428 CAGC-AGCGCGTGCATGAGTCAGCACCGGACTACGGTTGA

XCAW_01295|471265562_c1449145-1448720 AGACACGCATGTGCACGAACCGCAAGGTCATTACGGCTGA

* ** ** ** ** * * ***** ***

PROTEIN ALIGNMENTS:

XAC1194|AAM36066.1|death-on-curing MNTRMLVWVTHALALAIHERQLSEHGGASGVRDEALLDSALARPQQLFSY

XCV1221|CAJ22852.1|putative ----MLVWITHALALAIHERQLSEHGGASGVRDEALLASALARPQQLFSY

PXO_02086|ACD60291.1|death-on-curing ----MIIWIERPLVLAIHDRQLAEHGGGSGVRDDALLDSALARPKQLSAY

XOC_1245|AEQ95433.1|death-on-curing ----MIIWIERPLVLAIHDRQLAEHGGGSGVRDDALLDSALARPKQLSAY

XAC29_05995|WP_015471825.1|phage ----MLVWVTHALALAIHERQLSEHGGASGVRDEALLDSALARPQQLFSY

XCAW_01295|AGI07097.1|Prophage ----MLVWVTHALALAIHERQLSEHGGASGVRDEALLDSALARPQQLFSY

*::*: :.*.****:***:****.*****:*** ******:** :*

XAC1194|AAM36066.1|death-on-curing GDPPPDLVGLTASLAYGLARNHPFVDGNKRTAHVCYRVFLLLNGAELIAS

XCV1221|CAJ22852.1|putative GDPPPDLVELTASLAYGFARNHPFVDGNKRTAHVCYRVFLLLNGAELIAS

PXO_02086|ACD60291.1|death-on-curing GDPSPDLAALAASLAYGLARHHPFVDGNKRTAAVACETFIVLNGATLLAG

XOC_1245|AEQ95433.1|death-on-curing GDPSADLAALAASLAYGLARHHPFVDGNKRTAAVACETFIVLNGATLLAG

XAC29_05995|WP_015471825.1|phage GDPPPDLVGLTASLAYGLARNHPFVDGNKRTAHVCYRVFLLLNGAELIAS

XCAW_01295|AGI07097.1|Prophage GDPPPDLVGLTASLAYGLARNHPFVDGNKRTAHVCYRVFLLLNGAELIAS

***..**. *:******:**:*********** *. ..*::**** *:*.

XAC1194|AAM36066.1|death-on-curing QEEKYVAMMRLADGAWSEATFAQWLRPRVRLRADTHVHEPQGHYG

XCV1221|CAJ22852.1|putative QEEKYVAMMGLADGAWSEATFAQWLRPRVRLRADTHVHEPQGHYG

PXO_02086|ACD60291.1|death-on-curing DLELYGFYIGLADGSLDEAACAGWLRPRLQSRHQQRVHESAPDYG

XOC_1245|AEQ95433.1|death-on-curing DLGLYGFYIGLADGSLDEAACAGWLRPRLQSRHQQRVHESAPDYG

XAC29_05995|WP_015471825.1|phage QEEKYVAMMRLADGAWSEATFAQWLRPRVRLRADTHVHEPQGHYG

XCAW_01295|AGI07097.1|Prophage QEEKYVAMMRLADGAWSEATFAQWLRPRVRLRADTHVHEPQGHYG

: * : ****: .**: * *****:: * : :***. .**

**12 - DOMAIN TIGR02609 - hypothetical_protein_XAC1195 (doc_partner)**

NUCLEOTIDE ALIGNMENTS:

XAC1195|21240774_c1362178-1361957 ATGAAACTCAAGATCACTGCCATCGGCAATTCCGCTGGCGTCATCCTGCC

XCV1222|78045556_c1368248-1368027 ATGAAGCTCAAGATCACCGCCATCGGCAATTCCGCCGGCATCATCCTGCC

PXO_02087|188574270_3818543-3818764 ATGAAACTCAAGATCACTGCCATCGGCAATTCCGCCGGCGTCATCCTGCC

XOC_1246|384417079_c1215084-1214863 ATGAAACTCAAGATCACTGCCATCGGCAATTCCGCCGGCGTCATCCTGCC

XAC29_06000|470469929_c1365260-1365039 ATGAAACTCAAGATCACTGCCATCGGCAATTCCGCTGGCGTCATCCTGCC

XCAW_01296|471265562_c1449375-1449154 ATGAAACTCAAGATCACTGCCATCGGCAATTCCGCTGGCGTCATCCTGCC

***** *********** ***************** *** **********

XAC1195|21240774_c1362178-1361957 CAAGGAACTGCTGGCCCGCCTGCGGCTTGGAAAGGGCGACGAACTCTATG

XCV1222|78045556_c1368248-1368027 TAAGGAACTGCTGGCCCGCCTGCGGCTTGGAAAGGGCGACGAACTCTATG

PXO_02087|188574270_3818543-3818764 CAAGGAACTGCTGGCCCGCCTGCGGCTCGGAAAGGGCGACGTGCTTTATG

XOC_1246|384417079_c1215084-1214863 CAAGGAACTGCTGGCCCGCCTGCGGCTCGGAAAGGGCGACGAGCTTTATG

XAC29_06000|470469929_c1365260-1365039 CAAGGAACTGCTGGCCCGCCTGCGGCTTGGAAAGGGCGACGAACTCTATG

XCAW_01296|471265562_c1449375-1449154 CAAGGAACTGCTGGCCCGCCTGCGGCTTGGAAAGGGCGACGAACTCTATG

************************** ************* ** ****

XAC1195|21240774_c1362178-1361957 CACTGGAAACACCCGACGGCATCAAGCTGACCGCATTCGACCCGACGTTG

XCV1222|78045556_c1368248-1368027 CACTGGAAACGCCCGACGGCATCAGGCTGACCGCATTCGACCCGACGTTG

PXO_02087|188574270_3818543-3818764 CACTGGAAACGCCCGACGGCATCAAGCTGACCGCCTTCGACCCGACGTTG

XOC_1246|384417079_c1215084-1214863 CACTGGAAACGCCCGACGGCATCAAGCTGACCGCCTTCGACCCGACGTTG

XAC29_06000|470469929_c1365260-1365039 CACTGGAAACACCCGACGGCATCAAGCTGACCGCATTCGACCCGACGTTG

XCAW_01296|471265562_c1449375-1449154 CACTGGAAACACCCGACGGCATCAAGCTGACCGCATTCGACCCGACGTTG

********** ************* ********* ***************

XAC1195|21240774_c1362178-1361957 GCGGCGCAGATGGATGTTGCCGAACAGGTGATGCGTGAGGATCGCCAGGT

XCV1222|78045556_c1368248-1368027 GCCGCGCAGATGGATGTGGCCGAGCAGGTGATGCGCGAGGATCGCCAAGT

PXO_02087|188574270_3818543-3818764 GCCGCACAGATGGATGTGGCCGAGCAGGTGATGCGTGAGCGCCGCACCTT

XOC_1246|384417079_c1215084-1214863 GCCGCGCAGATGGATGTGGCCGAGCAGGTGATGCGTGAGCGCCGCACCTT

XAC29_06000|470469929_c1365260-1365039 GCGGCGCAGATGGATGTTGCCGAACAGGTGATGCGTGAGGATCGCCAGGT

XCAW_01296|471265562_c1449375-1449154 GCGGCGCAGATGGATGTTGCCGAACAGGTGATGCGTGAGGATCGCCAGGT

** ** *********** ***** *********** *** *** *

XAC1195|21240774_c1362178-1361957 GCTGAACAAGCTGGCCAAGTGA

XCV1222|78045556_c1368248-1368027 GCTGAACAAGCTGGCCAAGTGA

PXO_02087|188574270_3818543-3818764 GCTCAACAAACTGGCGCAGTAA

XOC_1246|384417079_c1215084-1214863 GCTCAACAAACTGGCGCAATAA

XAC29_06000|470469929_c1365260-1365039 GCTGAACAAGCTGGCCAAGTGA

XCAW_01296|471265562_c1449375-1449154 GCTGAACAAGCTGGCCAAGTGA

*** ***** ***** * * *

PROTEIN ALIGNMENTS:

XAC1195|21107341|gb|AAM36067.1| MKLKITAIGNSAGVILPKELLARLRLGKGDELYALETPDGIKLTAFDPTL

XCV1222|78035208|emb|CAJ22853.1| MKLKITAIGNSAGIILPKELLARLRLGKGDELYALETPDGIRLTAFDPTL

PXO_02087|188522345|gb|ACD60290.1| MKLKITAIGNSAGVILPKELLARLRLGKGDVLYALETPDGIKLTAFDPTL

XOC_1246|353461155|gb|AEQ95434.1| MKLKITAIGNSAGVILPKELLARLRLGKGDELYALETPDGIKLTAFDPTL

XAC29_06000|469766290|gb|AGH76707.1| MKLKITAIGNSAGVILPKELLARLRLGKGDELYALETPDGIKLTAFDPTL

XCAW_01296|471210348|gb|AGI07098.1| MKLKITAIGNSAGVILPKELLARLRLGKGDELYALETPDGIKLTAFDPTL

*************:**************** **********:********

XAC1195|21107341|gb|AAM36067.1| AAQMDVAEQVMREDRQVLNKLAK

XCV1222|78035208|emb|CAJ22853.1| AAQMDVAEQVMREDRQVLNKLAK

PXO_02087|188522345|gb|ACD60290.1| AAQMDVAEQVMRERRTLLNKLAQ

XOC_1246|353461155|gb|AEQ95434.1| AAQMDVAEQVMRERRTLLNKLAQ

XAC29_06000|469766290|gb|AGH76707.1| AAQMDVAEQVMREDRQVLNKLAK

XCAW_01296|471210348|gb|AGI07098.1| AAQMDVAEQVMREDRQVLNKLAK

************* * :*****:

**13 - DOMAIN COG1396 - HipB / cd00093 - HTH_XRE**

NUCLEOTIDE ALIGNMENTS:

XCC0532|21229478_c649919-648561 GTGG-----AGC-------------GCCTGATCACCACGCTGCGCGTCAA

XAC0188|21240774_c231312-230731 ATGG-----AT-------------------------ACTGTCCGCA----

XC_0544|66766352_c651982-650624 GTGG-----AGC-------------GCCTGATCACCACGCTGCGCGTCAA

PXO_03585|188574270_c286624-286004 ATGG-----AT-------------------------ACGGTCCGCA----

XCR_3973|384425691_4262526-4263902 ATGCCCCGTATAGCTGCATTAGAACGCCTGATCACCACGCTGCGCGTCAA

XOC_0275|384417079_c270664-270044 ATGG-----AT-------------------------ACGGTCCGCA----

XAC29_00965|470469929_c232467-231886 ATGG-----AT-------------------------ACTGTCCGCA----

XCAW_00584|471265562_c692822-692241 ATGG-----AT-------------------------ACTGTCCGCA----

** * ** * ***

XCC0532|21229478_c649919-648561 CGGCCCCATGCGTGGGGCCGAGTTGGCGGTGCGCGTGGGCGTGAGCCGGC

XAC0188|21240774_c231312-230731 CG---CC-----T---TCCGA--TATCGGTA-------ATATCATCC---

XC_0544|66766352_c651982-650624 CGGCCCCATGCGTGGGGCCGAGTTGGCGGTGCGCGTGGGCGTGAGCCGGC

PXO_03585|188574270_c286624-286004 CT---CC-----T---TCCGA--TGTCGGCA-------ACATCATCC---

XCR_3973|384425691_4262526-4263902 CGGCCCCATGCGTGCGGCCGAGTTGGCGGTGCGCTTGGGCGTGAGCCGGC

XOC_0275|384417079_c270664-270044 CT---CC-----T---TCCGA--TGTCGGCA-------ACATCATCC---

XAC29_00965|470469929_c232467-231886 CG---CC-----T---TCCGA--TATCGGTA-------ATATCATCC---

XCAW_00584|471265562_c692822-692241 CG---CC-----T---TCCGA--TATCGGTA-------ATATCATCC---

* ** * **** * *** * * **

XCC0532|21229478_c649919-648561 CGACTCTGTCTCGCCTAGTCGCAGATGCAGGCCCTGCGGTGCTGCGTTAT

XAC0188|21240774_c231312-230731 ----------GTGC-CAGGCGCAAGCGC-----CTG---GGCTGGGATC-

XC_0544|66766352_c651982-650624 CGACTCTGTCTCGCCTAGTCGCAGATGCAGGCCCTGCGGTGCTGCGTTAT

PXO_03585|188574270_c286624-286004 ----------GCGC-CAGGCGCAAACGC-----CTG---GGCTGGGATC-

XCR_3973|384425691_4262526-4263902 CGACCCTGTCTCGCCTAGTCGCAGATGCAGGCCCGGCGGTGCTGCGTTAT

XOC_0275|384417079_c270664-270044 ----------GCGC-CAGGCGCAAACGC-----CTG---GGCTGGGATC-

XAC29_00965|470469929_c232467-231886 ----------GTGC-CAGGCGCAAGCGC-----CTG---GGCTGGGATC-

XCAW_00584|471265562_c692822-692241 ----------GTGC-CAGGCGCAAGCGC-----CTG---GGCTGGGATC-

** ** **** ** * * **** * *

XCC0532|21229478_c649919-648561 GGCAGGGCGCGCAGCACCGGCTACGCGGCCACCGGTGAACTGCGGGGCGA

XAC0188|21240774_c231312-230731 ---AG-GCTCGC--------C-----------------------------

XC_0544|66766352_c651982-650624 GGCAGGGCGCGCAGCACCGGCTACGCGGCCACCGGTGAACTGCGGGGCGA

PXO_03585|188574270_c286624-286004 ---AG-GCTCGC--------C-----------------------------

XCR_3973|384425691_4262526-4263902 GGCAGGGCGCGCAGCACCGGCTACGCGGCCACCGGTGAACTGCGGGGCGA

XOC_0275|384417079_c270664-270044 ---AG-GCTCGC--------C-----------------------------

XAC29_00965|470469929_c232467-231886 ---AG-GCTCGC--------C-----------------------------

XCAW_00584|471265562_c692822-692241 ---AG-GCTCGC--------C-----------------------------

** ** *** *

XCC0532|21229478_c649919-648561 GCGTATGTGGCCGCTCTATCGGATTGATCACGATGCCCGCGTCATACCGC

XAC0188|21240774_c231312-230731 -------TGGC----------------CCACGAAATCGGCGTG-------

XC_0544|66766352_c651982-650624 GCGTATGTGGCCGCTCTATCGGATTGATCACGATGCCCGCGTCATACCGC

PXO_03585|188574270_c286624-286004 -------TGGC----------------CCACGAAATCGGCGTG-------

XCR_3973|384425691_4262526-4263902 GCGTATGTGGCCGCTCTATCGGATTGATCACGATGCCCGCGTCATACCGC

XOC_0275|384417079_c270664-270044 -------TGGC----------------CCACGAAATCGGCGTG-------

XAC29_00965|470469929_c232467-231886 -------TGGC----------------CCACGAAATCGGCGTG-------

XCAW_00584|471265562_c692822-692241 -------TGGC----------------CCACGAAATCGGCGTG-------

**** ***** * ****

XCC0532|21229478_c649919-648561 TGGGCCAACTATTGGCACTCCACGACGACCACTTCTTTGTCGAACTTTTG

XAC0188|21240774_c231312-230731 --AGCCGACAATGGA---TC---GTCGA---------TATCGA------G

XC_0544|66766352_c651982-650624 TGGGCCAACTATTGGCACTCCACGACGACCACTTCTTTGTCGAACTTTTG

PXO_03585|188574270_c286624-286004 --AGCCGACAGTGGA---TC---GTCGA---------TATCGA------G

XCR_3973|384425691_4262526-4263902 TGGGCCAACTATTGGCACTCCACAACGACCACTTCTTTGTCGAACTTTTG

XOC_0275|384417079_c270664-270044 --AGCCGACAATGGA---TC---GTCGA---------TATCGA------G

XAC29_00965|470469929_c232467-231886 --AGCCGACAATGGA---TC---GTCGA---------TATCGA------G

XCAW_00584|471265562_c692822-692241 --AGCCGACAATGGA---TC---GTCGA---------TATCGA------G

*** ** * * ** *** * **** *

XCC0532|21229478_c649919-648561 CAGGACAAC-CCCGTTCTATTGCATTCGCCATTGGACACCGGCGTGTTCG

XAC0188|21240774_c231312-230731 AAAGGCAAGCCGCGCGC----GGAGTTGCAAT------------------

XC_0544|66766352_c651982-650624 CAGGACAAC-CCCGTTCTATTGCATTCGCCATTGGACACCGGCGTGTTCG

PXO_03585|188574270_c286624-286004 AAAGGAAAGCCGCGTGC----GGAACTGCAGC------------------

XCR_3973|384425691_4262526-4263902 CAGGACAAC-CCCGTTCTATTGCATTCGCCATTGGACACCGGCGTGTTCG

XOC_0275|384417079_c270664-270044 AAAGGAAAGCCGCGTGC----GGAACTGCAGC------------------

XAC29_00965|470469929_c232467-231886 AAAGGCAAGCCGCGCGC----GGAGTTGCAAT------------------

XCAW_00584|471265562_c692822-692241 AAAGGCAAGCCGCGCGC----GGAGTTGCAAT------------------

* * ** * ** * * * **

XCC0532|21229478_c649919-648561 CATCGCTGCCCTGGTTTATAGACGACCAAAGGCCGCAGGGATTTCTTGGT

XAC0188|21240774_c231312-230731 ----------------------------------------------TGAT

XC_0544|66766352_c651982-650624 CATCGCTGCCCTGGTTTATAGACGACCAAAGGCCGCAGGGATTTCTTGGT

PXO_03585|188574270_c286624-286004 ----------------------------------------------TGAT

XCR_3973|384425691_4262526-4263902 CATCGCTGCCCTGGTTTATCGACGACCAAAGGCCACAGGGATTTCTTGGT

XOC_0275|384417079_c270664-270044 ----------------------------------------------TGAT

XAC29_00965|470469929_c232467-231886 ----------------------------------------------TGAT

XCAW_00584|471265562_c692822-692241 ----------------------------------------------TGAT

** *

XCC0532|21229478_c649919-648561 CGCAACCTGGCACACCGTATTTCTGCGCCGTTGCGCCTTCCGGACAACTT

XAC0188|21240774_c231312-230731 CCTG--CGGGCA--CTGC-------------------------------A

XC_0544|66766352_c651982-650624 CGCAACCTGGCACACCGTATTTCTGCGCCGTTGCGCCTTCCGGACAACTT

PXO_03585|188574270_c286624-286004 CCTA--AGAGCG--CTGC-------------------------------A

XCR_3973|384425691_4262526-4263902 CGCAACCTGGCACACCGTATTTCCGCGCCGTTGCGCCTTCCGGACAACTT

XOC_0275|384417079_c270664-270044 CCTA--AGAGCG--CTGC-------------------------------A

XAC29_00965|470469929_c232467-231886 CCTG--CGGGCA--CTGC-------------------------------A

XCAW_00584|471265562_c692822-692241 CCTG--CGGGCA--CTGC-------------------------------A

* ** * *

XCC0532|21229478_c649919-648561 GGCGCTGTGGTCGCCCAGGGACAACCTTGTGGCCATGCTGTCGGTTGGTC

XAC0188|21240774_c231312-230731 GGCGCTGGGGCT------------------GGAGTTGATGTT----GG--

XC_0544|66766352_c651982-650624 GGCGCTGTGGTCGCCCAGGGACAACCTTGTGGCCATGCTGTCGGTTGGTC

PXO_03585|188574270_c286624-286004 GGCGCTGGGGCT------------------GGAGTTGATTCT----GA--

XCR_3973|384425691_4262526-4263902 GGCGCTGTGGTCGCCCAGGGACAACCTTGTGGCCATGCTGTCGGTTGGCC

XOC_0275|384417079_c270664-270044 GGCGCTGGGGCT------------------GGAGTTGATTCT----GA--

XAC29_00965|470469929_c232467-231886 GGCGCTGGGGCT------------------GGAGTTGATGTT----GG--

XCAW_00584|471265562_c692822-692241 GGCGCTGGGGCT------------------GGAGTTGATGTT----GG--

******* ** ** ** * *

XCC0532|21229478_c649919-648561 ACGACCAGCCTGGCGATCTGCTGATCGGCGAGCGCGCACTGGCTCTGGCT

XAC0188|21240774_c231312-230731 --GCCCA----GGC-----------------------GT-TGCCCAGGTT

XC_0544|66766352_c651982-650624 ACGACCAGCCTGGCGATCTGCTGATCGGCGAGCGCGCACTGGCTCTGGCT

PXO_03585|188574270_c286624-286004 --GCTCG----GAC-----------------------GT-TGCTCAGATT

XCR_3973|384425691_4262526-4263902 ACGACCAGCCTGGCGACCTGCTGATCGGCGAACGCGCATTGGCTCTGGCT

XOC_0275|384417079_c270664-270044 --GCTCG----GAC-----------------------GT-TGCTCAGATT

XAC29_00965|470469929_c232467-231886 --GCCCA----GGC-----------------------GT-TGCCCAGGTT

XCAW_00584|471265562_c692822-692241 --GCCCA----GGC-----------------------GT-TGCCCAGGTT

* * * * ** * * *

XCC0532|21229478_c649919-648561 TTGGCGGCGATCGAACACCCTGAAGTTATCGCCACGCAGGCACGCACCGA

XAC0188|21240774_c231312-230731 TCGGAGGTGGGCGT---GCCTGA-----TCGCGACACCGTAACG--CCGA

XC_0544|66766352_c651982-650624 TTGGCGGCGATCGAACACCCTGAAGTTATCGCCACGCAGGCACGCACCGA

PXO_03585|188574270_c286624-286004 CCGCCGGCGAACGC---ACCTGA-----GCGCGGCACCGTGACG--CCGA

XCR_3973|384425691_4262526-4263902 TTGGCGGCGATCGAACAGCCTGAAGTCATCGCCACGCAGGCACGCTCCGA

XOC_0275|384417079_c270664-270044 CCGCCGGCGAACGC---GCCTGA-----GCGCGGTACCGTGACG--CCGA

XAC29_00965|470469929_c232467-231886 TCGGAGGTGGGCGT---GCCTGA-----TCGCGACACCGTAACG--CCGA

XCAW_00584|471265562_c692822-692241 TCGGAGGTGGGCGT---GCCTGA-----TCGCGACACCGTAACG--CCGA

* ** * ** ***** *** * * *** ****

XCC0532|21229478_c649919-648561 GGAATATCTCAGTCGCGCCGAAGCTGCATTGCGCGGCGAGGCCGTTGGCT

XAC0188|21240774_c231312-230731 TCAAT--CTCG-ACGCG---A----TCATTGAAC----------------

XC_0544|66766352_c651982-650624 GGAATATCTCAGTCGCGCCGAAGCTGCATTGCGCGGCGAGGCCGTTGGCT

PXO_03585|188574270_c286624-286004 TCGAT--CTCG-ACGCG---A----TCATTGAAC----------------

XCR_3973|384425691_4262526-4263902 GGAATATCTCAGTCGCGCCGAAGCTGCATTGCGCGGCGAGGCCGTTGGCT

XOC_0275|384417079_c270664-270044 TCGAT--CTCG-ACGCG---A----TCATTGAAC----------------

XAC29_00965|470469929_c232467-231886 TCAAT--CTCG-ACGCG---A----TCATTGAAC----------------

XCAW_00584|471265562_c692822-692241 TCAAT--CTCG-ACGCG---A----TCATTGAAC----------------

** *** **** * ***** *

XCC0532|21229478_c649919-648561 CGTCTGCCGCCGGCAAACAGCCGAAGTTCACCGCAGTGCTGAAGGAACGC

XAC0188|21240774_c231312-230731 -ATA-AC---CGG------GCC--AATCCACCA--TTG-----GGAACG-

XC_0544|66766352_c651982-650624 CGTCTGCCGCCGGCAAACAGCCGAAGTTCACCGCAGTGCTGAAGGAACGC

PXO_03585|188574270_c286624-286004 -ACA-AC---CGG------TCC--AATTCACGA--TTG-----GGAACG-

XCR_3973|384425691_4262526-4263902 CGTCTGCCGCCGGCGAGCAGCCGAAGTTCACCGCAGTGCTGAAGGAACAC

XOC_0275|384417079_c270664-270044 -ACA-AC---CGG------TCC--AATTCACGA--TTG-----GGAACG-

XAC29_00965|470469929_c232467-231886 -ATA-AC---CGG------GCC--AATCCACCA--TTG-----GGAACG-

XCAW_00584|471265562_c692822-692241 -ATA-AC---CGG------GCC--AATCCACCA--TTG-----GGAACG-

* *** ** * * *** ** *****

XCC0532|21229478_c649919-648561 GAAGCATACCGACCCGTGATCGTCAAGTTCACCGATCAGATCGATCAACC

XAC0188|21240774_c231312-230731 -----------AC-CGTTT---TCAA-------------TGCGTTCAAC-

XC_0544|66766352_c651982-650624 GAAGCATACCGACCCGTGATCGTCAAGTTCACCGATCAGATCGATCAACC

PXO_03585|188574270_c286624-286004 -----------AC-CGTTC---TCAA-------------TGCGTTTAAC-

XCR_3973|384425691_4262526-4263902 GGCGCGTATCGCCCCGTGATCGTCAAGTTCACCGATCAGATCGATCAACC

XOC_0275|384417079_c270664-270044 -----------AC-CGTTC---TCAA-------------TGCGTTTAAC-

XAC29_00965|470469929_c232467-231886 -----------AC-CGTTT---TCAA-------------TGCGTTCAAC-

XCAW_00584|471265562_c692822-692241 -----------AC-CGTTT---TCAA-------------TGCGTTCAAC-

* *** **** ** * ***

XCC0532|21229478_c649919-648561 CACCGGCCGGCGCTGGGCGGATCTTTTACAGATGGAATGGTTGGCCAGCC

XAC0188|21240774_c231312-230731 -AACAA-------------CATCTTCAA------GAAT-----------C

XC_0544|66766352_c651982-650624 CACCGGCCGGCGCTGGGCGGATCTTTTACAGATGGAATGGTTGGCCAGCC

PXO_03585|188574270_c286624-286004 -AACAA-------------CATCTTCAA------GAAT-----------C

XCR_3973|384425691_4262526-4263902 CACCGGCCGGCGCTGGGCGGATCTTTTACAGATGGAATGGTTGGCCAGCC

XOC_0275|384417079_c270664-270044 -AACAA-------------CATCTTCAA------GAAT-----------C

XAC29_00965|470469929_c232467-231886 -AACAA-------------CATCTTCAA------GAAT-----------C

XCAW_00584|471265562_c692822-692241 -AACAA-------------CATCTTCAA------GAAT-----------C

* * ***** * **** *

XCC0532|21229478_c649919-648561 AGATTCTCGTTGCCGCCGGCATCCCTGCTGCGGCAACACAGGTACTGCAG

XAC0188|21240774_c231312-230731 A-ATTCTCGC---CGTT-----CGA---TGCGC-----------------

XC_0544|66766352_c651982-650624 AGATTCTCGTTGCCGCCGGCATCCCTGCTGCGGCAACACAGGTACTGCAG

PXO_03585|188574270_c286624-286004 A-ATTCTCCT---CGCT-----CGA---TAGGC-----------------

XCR_3973|384425691_4262526-4263902 AGATTCTCGTTGCCGCCGGCATCCCTGCTGCGGCAACGCAGGTACTGCAG

XOC_0275|384417079_c270664-270044 A-ATTCTCCT---CGCT-----CGA---TAGGC-----------------

XAC29_00965|470469929_c232467-231886 A-ATTCTCGC---CGTT-----CGA---TGCGC-----------------

XCAW_00584|471265562_c692822-692241 A-ATTCTCGC---CGTT-----CGA---TGCGC-----------------

* ****** ** * * *

XCC0532|21229478_c649919-648561 GCAGGCGGGCGCGTCTTTCTCGAATCGACACGCTTCGATCGGACTGCTGC

XAC0188|21240774_c231312-230731 -------------------TGAAAAACACTGGCTTAGATAA---------

XC_0544|66766352_c651982-650624 GCAGGCGGGCGCGTCTTTCTCGAATCGACACGCTTCGATCGGACTGCTGC

PXO_03585|188574270_c286624-286004 -------------------TGAAAAACGCCGACTTGCACAG---------

XCR_3973|384425691_4262526-4263902 GCAGGCGGGCGCGTTTTTCTCGAATCGACACGCTTCGATCGGACTGCTGC

XOC_0275|384417079_c270664-270044 -------------------TGAAAAACGCCGACTTGCACAG---------

XAC29_00965|470469929_c232467-231886 -------------------TGAAAAACACTGGCTTAGATAA---------

XCAW_00584|471265562_c692822-692241 -------------------TGAAAAACACTGGCTTAGATAA---------

* ** * *** *

XCC0532|21229478_c649919-648561 GTTAGGCCGACGCGGATTCGTCTCGCTGATGTCGCTGTTTGCGTCCTTTT

XAC0188|21240774_c231312-230731 -------------------ATCTCC------------------CCCGATT

XC_0544|66766352_c651982-650624 GTTAGGCCGACGCGGATTCGTCTCGCTGATGTCGCTGTTTGCGTCCTTTT

PXO_03585|188574270_c286624-286004 -------------------ACCATT------------------CTCGG--

XCR_3973|384425691_4262526-4263902 GTTAGGCCGACGCGGATTCGTCTCGCTGATGTCGCTGTTTGCGTCCTTTT

XOC_0275|384417079_c270664-270044 -------------------ACCATT------------------CTCGG--

XAC29_00965|470469929_c232467-231886 -------------------ATCTCC------------------CCCGATT

XCAW_00584|471265562_c692822-692241 -------------------ATCTCC------------------CCCGATT

* *

XCC0532|21229478_c649919-648561 ATGGCGACGCAAACGTCAGCACCTGGCGAACACTTGCTCCACTACTCAAA

XAC0188|21240774_c231312-230731 AT--CGGTGCAAGCGTTAAA------------------------------

XC_0544|66766352_c651982-650624 ATGGCGACGCAAACGTCAGCACCTGGCGAACACTTGCTCCACTACTCAAA

PXO_03585|188574270_c286624-286004 ----CAGTGGATGCTCTAGA------------------------------

XCR_3973|384425691_4262526-4263902 ATGGCGACGCAAACATCAGCACCTGGCGAACACTTGCTCCACTACTCAAA

XOC_0275|384417079_c270664-270044 ----CAGTGGATGCTCTAGA------------------------------

XAC29_00965|470469929_c232467-231886 AT--CGGTGCAAGCGTTAAA------------------------------

XCAW_00584|471265562_c692822-692241 AT--CGGTGCAAGCGTTAAA------------------------------

* * * * *

XCC0532|21229478_c649919-648561 CGCGATGGCTGGCTGAGCCCGGAAGACGCCGAGCG-----------CCTG

XAC0188|21240774_c231312-230731 --AA----------GAATCCAGC---CAATGAGCA-----------CTTG

XC_0544|66766352_c651982-650624 CGCGATGGCTGGCTGAGCCCGGAAGACGCCGAGCG-----------CCTG

PXO_03585|188574270_c286624-286004 -GCA----------GTGTCCGGCA--CAGGCAGCATCAGCAGTAGCTCTG

XCR_3973|384425691_4262526-4263902 CGCGATGGCTGGCTGAGCCCGGAAGACGCCGAGCG-----------CCTG

XOC_0275|384417079_c270664-270044 -GCA----------GTCTCCGGTA--CAGGGAGCATCAGCAGTAGACCTG

XAC29_00965|470469929_c232467-231886 --AA----------GAATCCAGC---CAATGAGCA-----------CTTG

XCAW_00584|471265562_c692822-692241 --AA----------GAATCCAGC---CAATGAGCA-----------CTTG

* ** * * *** **

XCC0532|21229478_c649919-648561 GAAGTGATCAGCTGGTTCGGGACCCTCATCGGC-AATACCGACATGCACC

XAC0188|21240774_c231312-230731 CA--T---AAGCAG-TTC-------TCA---GC-AA---CGAA-------

XC_0544|66766352_c651982-650624 GAAGTGATCAGCTGGTTCGGGACCCTCATCGGC-AATACCGACATGCACC

PXO_03585|188574270_c286624-286004 AA--T---AGATAT-TCC-------TCG---ATGAT---CGAT-------

XCR_3973|384425691_4262526-4263902 GAAGTGATCAGCTGGTTCGGGACCCTCATCGGC-AATACCGACATGCACC

XOC_0275|384417079_c270664-270044 AA--T---AGATAT-TCC-------TCG---ATGAT---CGAT-------

XAC29_00965|470469929_c232467-231886 CA--T---AAGCAG-TTC-------TCA---GC-AA---CGAA-------

XCAW_00584|471265562_c692822-692241 CA--T---AAGCAG-TTC-------TCA---GC-AA---CGAA-------

* * * * ** * ***

XCC0532|21229478_c649919-648561 TGGGCAATGCCGCACTGGTACTCGCCGATACCCTGCCCCTGCAGCTTGCC

XAC0188|21240774_c231312-230731 ----------------------------------TCGCT-----------

XC_0544|66766352_c651982-650624 TGGGCAATGCCGCACTGGTACTCGCCGATACCCTGCCCCTGCAGCTTGCC

PXO_03585|188574270_c286624-286004 ----------------------------------GCGCC-----------

XCR_3973|384425691_4262526-4263902 TGGGCAATGCCGCACTGGTACTCGCCGATACCCTGCCCCTGCAGCTTGCC

XOC_0275|384417079_c270664-270044 ----------------------------------GCGCC-----------

XAC29_00965|470469929_c232467-231886 ----------------------------------TCGCT-----------

XCAW_00584|471265562_c692822-692241 ----------------------------------TCGCT-----------

* *

XCC0532|21229478_c649919-648561 CCGGTGTACGACATGTTGCCGATGGCGCTGCGCCCGGCATCCAGTGGCGA

XAC0188|21240774_c231312-230731 ----TGA----AGA-TTGGCGA-----G----------------------

XC_0544|66766352_c651982-650624 CCGGTGTACGACATGTTGCCGATGGCGCTGCGCCCGGCATCCAGTGGCGA

PXO_03585|188574270_c286624-286004 -AAATGAAAGAAAT-TTGGTGA-----GTGAGCC----------------

XCR_3973|384425691_4262526-4263902 CCGGTGTACGACATGTTGCCGATGGCGCTGCGCCCGGCATCCAGTGGCGA

XOC_0275|384417079_c270664-270044 -AAATGAAAGAAAT-TTGGTGA-----GTGAGCC----------------

XAC29_00965|470469929_c232467-231886 ----TGA----AGA-TTGGCGA-----G----------------------

XCAW_00584|471265562_c692822-692241 ----TGA----AGA-TTGGCGA-----G----------------------

** *** **

XCC0532|21229478_c649919-648561 AGTCGTGCACAGAGACATCGAGATTCCGCCGCCCACCGTGGATCAATTCG

XAC0188|21240774_c231312-230731 --------------ACA-----GTAG------------CTGATAG-TTTG

XC_0544|66766352_c651982-650624 AGTCGTGCACAGAGACATCGAGATTCCGCCGCCCACCGTGGATCAATTCG

PXO_03585|188574270_c286624-286004 ---CTTGCAA--AAACG-----GTTC-CCCGCGC----TTGATCG-TCCG

XCR_3973|384425691_4262526-4263902 AGTCGTGCACAGAGACATCGAGATTCCGCCGCCCACCGTGGATCAATTCG

XOC_0275|384417079_c270664-270044 ---CTTGCAA--AAACA-----GTTC-CCCGCGC----TTGATCG-TCCG

XAC29_00965|470469929_c232467-231886 --------------ACA-----GTAG------------CTGATAG-TTTG

XCAW_00584|471265562_c692822-692241 --------------ACA-----GTAG------------CTGATAG-TTTG

** * *** * *

XCC0532|21229478_c649919-648561 CCCAATGGCGGCGCGCTGCCGGAATGGCCGAGCAGT--TCTGGCAACATG

XAC0188|21240774_c231312-230731 AAGATCGGCGAGGC--AGAGAAAATATCCAAG--GTCATCTC-------G

XC_0544|66766352_c651982-650624 CCCAATGGCGGCGCGCTGCCGGAATGGCCGAGCAGT--TCTGGCAACATG

PXO_03585|188574270_c286624-286004 ACGATTGGCGAGAC--AACGAAAATATCCAAG--GTCATATC-------G

XCR_3973|384425691_4262526-4263902 CCCAATGGCGGCGCGCTGCCGGAATGGCCGAGCAGT--TCTGGCAACATG

XOC_0275|384417079_c270664-270044 ACGATTGGCGAGAC--AACGAAAATATCCAAG--GTCATATC-------G

XAC29_00965|470469929_c232467-231886 AAGATCGGCGAGGC--AGAGAAAATATCCAAG--GTCATCTC-------G

XCAW_00584|471265562_c692822-692241 AAGATCGGCGAGGC--AGAGAAAATATCCAAG--GTCATCTC-------G

* **** * *** ** ** ** * * *

XCC0532|21229478_c649919-648561 CGATCGACGACGACACTCTGAGCGAACAGATGCGCGGCATTGCCACGCGC

XAC0188|21240774_c231312-230731 CCAT----------CTTCC----AAAAAGAAGGG----------------

XC_0544|66766352_c651982-650624 CGATCGACGACGACACTCTGAGCGAACAGATGCGCGGCATTGCCACGCGC

PXO_03585|188574270_c286624-286004 AAAC----------CTTCT----AAAAAGAAGGC----------------

XCR_3973|384425691_4262526-4263902 CGATCGACGACGACACTCTGAGCGAACAGATGCGCGGCATTGCCACGCGC

XOC_0275|384417079_c270664-270044 AAAC----------CTTCT----AAAAAGAAGGC----------------

XAC29_00965|470469929_c232467-231886 CCAT----------CTTCC----AAAAAGAAGGG----------------

XCAW_00584|471265562_c692822-692241 CCAT----------CTTCC----AAAAAGAAGGG----------------

* ** ** *** *

XCC0532|21229478_c649919-648561 GCGTTGGCAAGCTTGCGTCGAACCGCATCACGCCTTGCCTGA

XAC0188|21240774_c231312-230731 --------------------------------CGTTCCGTGA

XC_0544|66766352_c651982-650624 GCGTTGGCAAGCTTGCGTCGAACCGCATCACGCCTTGCCTGA

PXO_03585|188574270_c286624-286004 --------------------------------CGATCCGTGA

XCR_3973|384425691_4262526-4263902 GCGTTGGCAAGCTTGCGTCGAACCGCATCACGCCTTGCCTGA

XOC_0275|384417079_c270664-270044 --------------------------------CGATCCGTGA

XAC29_00965|470469929_c232467-231886 --------------------------------CGTTCCGTGA

XCAW_00584|471265562_c692822-692241 --------------------------------CGTTCCGTGA

* * * ***

PROTEIN ALIGNMENTS:

XCC0532|AAM39848.1|conserved MERLIT------TLRVNGPMRG---------AELAVRVGVSRPTLSRLVA

XAC0188|AAM35080.1|transcriptional MDTVRT------PSDIGNIIRARRKRLGWDQARLAHEIGVSRQ-------

XC_0544|AAY47625.1|conserved MERLIT------TLRVNGPMRG---------AELAVRVGVSRPTLSRLVA

XC_0544|ACD56938.1|transcriptional MDTVRT------PSDVGNIIRARRKRLGWDQARLAHEIGVSRQ-------

XCR_3973|AEL08832.1|conserved MPRIAALERLITTLRVNGPMRA---------AELAVRLGVSRPTLSRLVA

XOC_0275|AEQ94514.1|transcriptional MDTVRT------PSDVGNIIRARRKRLGWDQARLAHEIGVSRQ-------

XAC29_00965|WP_011050156.1|MULT MDTVRT------PSDIGNIIRARRKRLGWDQARLAHEIGVSRQ-------

XCAW_00584|AGI06403.1|Hypothetical MDTVRT------PSDIGNIIRARRKRLGWDQARLAHEIGVSRQ-------

* : : . :.. :*. *.** .:****

XCC0532|AAM39848.1|conserved DAGPAVLRYGRARSTGYAATGELRGERMWPLYRIDHDARVIPLGQLLALH

XAC0188|AAM35080.1|transcriptional --------------------------------------------------

XC_0544|AAY47625.1|conserved DAGPAVLRYGRARSTGYAATGELRGERMWPLYRIDHDARVIPLGQLLALH

XC_0544|ACD56938.1|transcriptional --------------------------------------------------

XCR_3973|AEL08832.1|conserved DAGPAVLRYGRARSTGYAATGELRGERMWPLYRIDHDARVIPLGQLLALH

XOC_0275|AEQ94514.1|transcriptional --------------------------------------------------

XAC29_00965|WP_011050156.1|MULT --------------------------------------------------

XCAW_00584|AGI06403.1|Hypothetical --------------------------------------------------

XCC0532|AAM39848.1|conserved DDHFFVELLQDNPVLLHSPLDTGVFASLPWFIDDQRPQGFLGRNLAHRIS

XAC0188|AAM35080.1|transcriptional -----------------------------WIVD-----------------

XC_0544|AAY47625.1|conserved DDHFFVELLQDNPVLLHSPLDTGVFASLPWFIDDQRPQGFLGRNLAHRIS

XC_0544|ACD56938.1|transcriptional -----------------------------WIVD-----------------

XCR_3973|AEL08832.1|conserved NDHFFVELLQDNPVLLHSPLDTGVFASLPWFIDDQRPQGFLGRNLAHRIS

XOC_0275|AEQ94514.1|transcriptional -----------------------------WIVD-----------------

XAC29_00965|WP_011050156.1|MULT -----------------------------WIVD-----------------

XCAW_00584|AGI06403.1|Hypothetical -----------------------------WIVD-----------------

*::*

XCC0532|AAM39848.1|conserved APLRLPDNLALWSPRDNLVAMLSVGHDQPGDLLIGERALALALAAIEHPE

XAC0188|AAM35080.1|transcriptional --------IEKGKPRAELQLIL----------------------------

XC_0544|AAY47625.1|conserved APLRLPDNLALWSPRDNLVAMLSVGHDQPGDLLIGERALALALAAIEHPE

XC_0544|ACD56938.1|transcriptional --------IEKGKPRAELQLIL----------------------------

XCR_3973|AEL08832.1|conserved APLRLPDNLALWSPRDNLVAMLSVGHDQPGDLLIGERALALALAAIEQPE

XOC_0275|AEQ94514.1|transcriptional --------IEKGKPRAELQLIL----------------------------

XAC29_00965|WP_011050156.1|MULT --------IEKGKPRAELQLIL----------------------------

XCAW_00584|AGI06403.1|Hypothetical --------IEKGKPRAELQLIL----------------------------

: .** :* :*

XCC0532|AAM39848.1|conserved VIATQARTEEYLSRAEAALRGEAVGSSAAGKQPKFTAVLKEREAYRPVIV

XAC0188|AAM35080.1|transcriptional -------------RALQALGLELMLGPGVAQVSEVG--VPDRDTVTPINL

XC_0544|AAY47625.1|conserved VIATQARTEEYLSRAEAALRGEAVGSSAAGKQPKFTAVLKEREAYRPVIV

XC_0544|ACD56938.1|transcriptional -------------RALQALGLELILSSDVAQIPPAN--APERGTVTPIDL

XCR_3973|AEL08832.1|conserved VIATQARSEEYLSRAEAALRGEAVGSSAAGEQPKFTAVLKEHGAYRPVIV

XOC_0275|AEQ94514.1|transcriptional -------------RALQALGLELILSSDVAQIPPAN--APERGTVTPIDL

XAC29_00965|WP_011050156.1|MULT -------------RALQALGLELMLGPGVAQVSEVG--VPDRDTVTPINL

XCAW_00584|AGI06403.1|Hypothetical -------------RALQALGLELMLGPGVAQVSEVG--VPDRDTVTPINL

** ** * : .. ..: . :: : *: :

XCC0532|AAM39848.1|conserved KFTDQIDQPTGRRWADLLQMEWLASQILVAAGIPAAATQVLQAGGRVFLE

XAC0188|AAM35080.1|transcriptional DAIIEHNRA-----------------------NPPLGTTVFNAFNNNIFK

XC_0544|AAY47625.1|conserved KFTDQIDQPTGRRWADLLQMEWLASQILVAAGIPAAATQVLQAGGRVFLE

XC_0544|ACD56938.1|transcriptional DAIIEHNRS-----------------------NSRLGTTVLNAFNNNIFK

XCR_3973|AEL08832.1|conserved KFTDQIDQPTGRRWADLLQMEWLASQILVAAGIPAAATQVLQAGGRVFLE

XOC_0275|AEQ94514.1|transcriptional DAIIEHNRS-----------------------NSRLGTTVLNAFNNNIFK

XAC29_00965|WP_011050156.1|MULT DAIIEHNRA-----------------------NPPLGTTVFNAFNNNIFK

XCAW_00584|AGI06403.1|Hypothetical DAIIEHNRA-----------------------NPPLGTTVFNAFNNNIFK

. : ::. . .* *::* .. :::

XCC0532|AAM39848.1|conserved STRFDRTAALGRRGFVSLMSLFASFYGDANVSTWRTLAPLLKRDGWLSPE

XAC0188|AAM35080.1|transcriptional N-Q-------------------------------------------FSPF

XC_0544|AAY47625.1|conserved STRFDRTAALGRRGFVSLMSLFASFYGDANVSTWRTLAPLLKRDGWLSPE

XC_0544|ACD56938.1|transcriptional N-Q-------------------------------------------FSSL

XCR_3973|AEL08832.1|conserved STRFDRTAALGRRGFVSLMSLFASFYGDANISTWRTLAPLLKRDGWLSPE

XOC_0275|AEQ94514.1|transcriptional N-Q-------------------------------------------FSSL

XAC29_00965|WP_011050156.1|MULT N-Q-------------------------------------------FSPF

XCAW_00584|AGI06403.1|Hypothetical N-Q-------------------------------------------FSPF

. : :*.

XCC0532|AAM39848.1|conserved DAERLEVISWFGTLIGNTDMHLGNAALVLADTLPLQLAPVYDMLPMALRP

XAC0188|AAM35080.1|transcriptional DA------------LKNTGLDKSPP-------------------------

XC_0544|AAY47625.1|conserved DAERLEVISWFGTLIGNTDMHLGNAALVLADTLPLQLAPVYDMLPMALRP

XC_0544|ACD56938.1|transcriptional DR------------LKNADLHRPFSAVDALEQCPAQAASAVALNRYS---

XCR_3973|AEL08832.1|conserved DAERLEVISWFGTLIGNTDMHLGNAALVLADTLPLQLAPVYDMLPMALRP

XOC_0275|AEQ94514.1|transcriptional DR------------LKNADLHRPFSAVDALEQSPVQGASAVDLNRYS---

XAC29_00965|WP_011050156.1|MULT DA------------LKNTGLDKSPP-------------------------

XCAW_00584|AGI06403.1|Hypothetical DA------------LKNTGLDKSPP-------------------------

* : *:.:. .

XCC0532|AAM39848.1|conserved ASSGEVVHRDIEIPPPTVDQFAQWRRAAGMAEQFWQHAIDDDTLSEQ---

XAC0188|AAM35080.1|transcriptional --IIGASVKKNPANEHLHKQFS---------------ATNRLKIGETVAD

XC_0544|AAY47625.1|conserved ASSGEVVHRDIEIPPPTVDQFAQWRRAAGMAEQFWQHAIDDDTLSEQ---

XC_0544|ACD56938.1|transcriptional -SMIDAPNERNLVSEPLQKRFP---------------ALDRPTIGET---

XCR_3973|AEL08832.1|conserved ASSGEVVHRDIEIPPPTVDQFAQWRRAAGMAEQFWQHAIDDDTLSEQ---

XOC_0275|AEQ94514.1|transcriptional -SMIDAPNERNLVSEPLQKQFP---------------ALDRPTIGET---

XAC29_00965|WP_011050156.1|MULT --IIGASVKKNPANEHLHKQFS---------------ATNRLKIGETVAD

XCAW_00584|AGI06403.1|Hypothetical --IIGASVKKNPANEHLHKQFS---------------ATNRLKIGETVAD

. . .:*. * : .:.*

XCC0532|AAM39848.1|conserved -------MRGIATRALASLRRTASRLA

XAC0188|AAM35080.1|transcriptional SLKIGEAEKISKVISPSSKKKGVP---

XC_0544|AAY47625.1|conserved -------MRGIATRALASLRRTASRLA

XC_0544|ACD56938.1|transcriptional -------TKISKVISKPSKKKADP---

XCR_3973|AEL08832.1|conserved -------MRGIATRALASLRRTASRLA

XOC_0275|AEQ94514.1|transcriptional -------TKISKVISKPSKKKADP---

XAC29_00965|WP_011050156.1|MULT SLKIGEAEKISKVISPSSKKKGVP---

XCAW_00584|AGI06403.1|Hypothetical SLKIGEAEKISKVISPSSKKKGVP---

: . : .* :: .

**14 - DOMAIN TIGR03071 - Couple_hipA / pfam07805 - HipA_N**

Nucleotide alignments:

XAC0187|21240774_c230734-230156 GTGAGACTGGTCGCGCTGATCGAAAACCAAACGATCGGGCACCTCGCGTC

PXO_03586|188574270_c286007-284730 GTGAGACTGGTCGCGCTGATCGAAAACCAGGTGATCGGCCATCTGGCGCC

XOC_0274|384417079_c270047-268770 GTGAGACTGGTCGCGCTGATCGAAAACCAGGTGATCGGCCATCTGACGCC

XAC29_00960|470469929_c231889-231329 GTGAGACTGGTCGCGCTGATCGAAAACCAAACGATCGGGCACCTCGCGTC

XCAW_00583|471265562_c692244-691666 GTGAGACTGGTCGCGCTGATCGAAAACCAAACGATCGGGCACCTCGCGTC

***************************** ****** ** ** ** *

XAC0187|21240774_c230734-230156 CGACCCGCACGGGCATCCACGATTCACCTACGCGCCGGACTGGCAGCATT

PXO_03586|188574270_c286007-284730 CGATCAGCATGGGCATCCACGATTCACCTACGCGCCGGACTGGCAGCATT

XOC_0274|384417079_c270047-268770 CGATCAGCATGGGCATCCACGATTCACCTACGCGCCGGACTGGCAGCATT

XAC29_00960|470469929_c231889-231329 CGACCCGCACGGGCATCCACGATTCACCTACGCGCCGGACTGGCAGCATT

XCAW_00583|471265562_c692244-691666 CGACCCGCACGGGCATCCACGATTCACCTACGCGCCGGACTGGCAGCATT

*** * *** ****************************************

XAC0187|21240774_c230734-230156 CAAAGAGTGCGATACCGCTGTCTTTGAACCTTCCCCTCAGCAGGGAACAT

PXO_03586|188574270_c286007-284730 CGAAAAATGCGATACCGCTGTCGTTGAACCTTCCCCTCAGCAAGGAGCAT

XOC_0274|384417079_c270047-268770 CGAAAAATGCGATACCGCTGTCGTTGAACCTTCCCCTCAGCAAGGAGCAT

XAC29_00960|470469929_c231889-231329 CAAAGAGTGCGATACCGCTGTCTTTGAACCTTCCCCTCAGCAGGGAACAT

XCAW_00583|471265562_c692244-691666 CAAAGAGTGCGATACCGCTGTCTTTGAACCTTCCCCTCAGCAGGGAACAT

* ** * *************** ******************* *** ***

XAC0187|21240774_c230734-230156 CACCCGACCGAGGCGGTCAGTGCAGTGCTGTGGGGCCTGCTGCCAGACAA

PXO_03586|188574270_c286007-284730 CATCCGACTGAGGCGGTCAGTGCAGTGCTATGGGGCCTGCTGCCAGACAA

XOC_0274|384417079_c270047-268770 CATCCGACTGAAGCGGTCAGTGCAGTGCTATGGGGCCTGCTGCCAGACAA

XAC29_00960|470469929_c231889-231329 CACCCGACCGAGGCGGTCAGTGCAGTGCTGTGGGGCCTGCTGCCAGACAA

XCAW_00583|471265562_c692244-691666 CACCCGACCGAGGCGGTCAGTGCAGTGCTGTGGGGCCTGCTGCCAGACAA

** ***** ** ***************** ********************

XAC0187|21240774_c230734-230156 CGAAGCCACCTTGCAACGCTGGGCCTCCAGGTTCCAGGTCTCGCCGCGCA

PXO_03586|188574270_c286007-284730 CGAAGCCACCTTGCAACGCTGGGCATCCAGGTTCCAGGTTTCGCCGCGCA

XOC_0274|384417079_c270047-268770 TGAAGCCACCTTGCAACGCTGGGCATCCAGGTTCCAGGTTTCGCCGCGCA

XAC29_00960|470469929_c231889-231329 CGAAGCCACCTTGCAACGCTGGGCCTCCAGGTTCCAGGTCTCGCCGCGCA

XCAW_00583|471265562_c692244-691666 CGAAGCCACCTTGCAACGCTGGGCCTCCAGGTTCCAGGTCTCGCCGCGCA

*********************** ************** **********

XAC0187|21240774_c230734-230156 ATCCGCTCGCCCTGTTGTCGCATGTGGGCGAGGATTGCGCTGGTGCGGTG

PXO_03586|188574270_c286007-284730 ACCCGCTCGCCCTGTTGTCGCATGTCGGCGAGGATTGCGCTGGTGCGGTG

XOC_0274|384417079_c270047-268770 ACCCGCTCGCCCTGTTGTCGCATGTCGGCGAGGATTGCGCTGGTGCGGTG

XAC29_00960|470469929_c231889-231329 ATCCGCTCGCCCTGTTGTCGCATGTGGGCGAGGATTGCGCTGGTGCGGTG

XCAW_00583|471265562_c692244-691666 ATCCGCTCGCCCTGTTGTCGCATGTGGGCGAGGATTGCGCTGGTGCGGTG

* *********************** ************************

XAC0187|21240774_c230734-230156 CAGTTCGTCACCGAAGCGAGGCTCGAAGATGTGCTGTCCGGCGCCAGCGA

PXO_03586|188574270_c286007-284730 CAGTTCGTCACCGAAGCTAGGCTTGAGGATGTACTGTCCGGTGCCAGCGA

XOC_0274|384417079_c270047-268770 CAGTTCGTCACTGAAGCTAGGCTTGAAGATGTGCTGTCCGGTGCCAGCGA

XAC29_00960|470469929_c231889-231329 CAGTTCGTCACCGAAGCGAGGCTCGAAGATGTGCTGTCCGGCGCCAGCGA

XCAW_00583|471265562_c692244-691666 CAGTTCGTCACCGAAGCGAGGCTCGAAGATGTGCTGTCCGGCGCCAGCGA

*********** ***** ***** ** ***** ******** ********

XAC0187|21240774_c230734-230156 TGGCATCGAACCGCTCACCGATGGCGAACTGGAAGCACGCTTGCGCAGGC

PXO_03586|188574270_c286007-284730 TGGCATCGAACCGCTCACCGATGGCGAACTGGAAGCACGCTTGCGCAGGC

XOC_0274|384417079_c270047-268770 TGGCATCGAACCGCTCACCGATGGCGAACTGGAAGCACGCTTGCGCAGGC

XAC29_00960|470469929_c231889-231329 TGGCATCGAACCGCTCACCGATGGCGAACTGGAAGCACGCTTGCGCAGGC

XCAW_00583|471265562_c692244-691666 TGGCATCGAACCGCTCACCGATGGCGAACTGGAAGCACGCTTGCGCAGGC

**************************************************

XAC0187|21240774_c230734-230156 TGCGTCAGGACATCGGTGCGGCGCGCCAAGTCGAAGACGTGGGGCAGTTC

PXO_03586|188574270_c286007-284730 TGCGGCAGGACATCGGTGCGGCGCGCCAAGTCGATGACGTCGGGCAGTTC

XOC_0274|384417079_c270047-268770 TGCGGCAGGACATCGGTGCGGCGCGCCAAGTCGATGACGTCGGGCAGTTC

XAC29_00960|470469929_c231889-231329 TGCGTCAGGACATCGGTGCGGCGCGCCAAGTCGAAGACGTGGGGCAGTTC

XCAW_00583|471265562_c692244-691666 TGCGTCAGGACATCGGTGCGGCGCGCCAAGTCGATGACGTGGGGCAGTTC

**** ***************************** ***** *********

XAC0187|21240774_c230734-230156 AGCCTGGCCGGCGCGCAGGCCAAGATCGCATTGCTGCACGACGGCCTGCA

PXO_03586|188574270_c286007-284730 AGCCTGGCAGGCGCGCAGGCCAAGATCGCACTGCTGCACGACGGCCTGCA

XOC_0274|384417079_c270047-268770 AGCCTGGCAGGCGCGCAGGCCAAGATCGCACTGCTGCACGACGGCCTGCA

XAC29_00960|470469929_c231889-231329 AGCCTGGCCGGCGCGCAGGCCAAGATCGCATTGCTGCACGACGGCCTGCA

XCAW_00583|471265562_c692244-691666 AGCCTGGCCGGCGCGCAGGCCAAGATCGCATTGCTGCACGACGGCCTGCA

******** ********************* *******************

XAC0187|21240774_c230734-230156 GTGGGCCATCCCGGCCGGACGCATCCCGACCACGCATATCCTCAAACCAC

PXO_03586|188574270_c286007-284730 ATGGGCCATCCCGGCCGGACGCATCCCGACCACGCATATCCTCAAACCAC

XOC_0274|384417079_c270047-268770 ATGGGCCATCCCGGCCGGACGCATCCCGACCACGCATATCCTCAAACCAC

XAC29_00960|470469929_c231889-231329 GTGGGCCATCCCGGCCGGACGCATCCCGACCACGCATATCCTCAAACCAC

XCAW_00583|471265562_c692244-691666 GTGGGCCATCCCGGCCGGACGCATCCCGACCACGCATATCCTCAAACCAC

*************************************************

XAC0187|21240774_c230734-230156 CGAGCGGCGAAT--------------------------------------

PXO_03586|188574270_c286007-284730 CGAGCGGCGAATACGACGGGTTCGTCGAGAACGAGTTGTTCTGCCTGTGC

XOC_0274|384417079_c270047-268770 CGAGCGGCGAATACGACGGGTTCGTCGAGAACGAGTTGTTCTGCCTGTGC

XAC29_00960|470469929_c231889-231329 CGAGCGGC------------------------------------------

XCAW_00583|471265562_c692244-691666 CGAGCGGCGAAT--------------------------------------

********

XAC0187|21240774_c230734-230156 --------------------------------------------------

PXO_03586|188574270_c286007-284730 CTGGCGCACAGCATCGGTCTGCCAACCGCAACCGCACAGGCGCTGCGCGT

XOC_0274|384417079_c270047-268770 CTGGCGCACAGCATCGGTCTGCCAACCGCAACCGCACAGGCGCTGCGCGT

XAC29_00960|470469929_c231889-231329 --------------------------------------------------

XCAW_00583|471265562_c692244-691666 --------------------------------------------------

XAC0187|21240774_c230734-230156 --------------------------------------------------

PXO_03586|188574270_c286007-284730 CGGCGCTGAAACCGCCATTTGCGTCAAACGTTACGACCGCACCGGTGTTA

XOC_0274|384417079_c270047-268770 CGGCGCTGAAACCGCCATTTGCGTCAAACGTTACGACCGCACCGGTGTTA

XAC29_00960|470469929_c231889-231329 --------------------------------------------------

XCAW_00583|471265562_c692244-691666 --------------------------------------------------

XAC0187|21240774_c230734-230156 --------------------------------------------------

PXO_03586|188574270_c286007-284730 ACGGAGTGCTGCTGCGCATCCATCAGGAAGATACCTGTCAGGCGCTGGGG

XOC_0274|384417079_c270047-268770 ACGGAGTGCTGCTGCGCATCCATCAGGAAGATACCTGTCAGGCGCTGGGG

XAC29_00960|470469929_c231889-231329 --------------------------------------------------

XCAW_00583|471265562_c692244-691666 --------------------------------------------------

XAC0187|21240774_c230734-230156 --------------------------------------------------

PXO_03586|188574270_c286007-284730 GTGATGCCGCACATCAAGTATCAGAACCAGGGCGGTCCTGGGGTGGAGGC

XOC_0274|384417079_c270047-268770 GTGATGCCGCACATCAAGTATCAGAACCAGGGCGGTCCTGGGGTGGAGGC

XAC29_00960|470469929_c231889-231329 --------------------------------------------------

XCAW_00583|471265562_c692244-691666 --------------------------------------------------

XAC0187|21240774_c230734-230156 --------------------------------------------------

PXO_03586|188574270_c286007-284730 GATCTCACAACTCTTGTGGACGCACTCGTCGCAACCGCGGCAAGACGTAG

XOC_0274|384417079_c270047-268770 GATCTCACAACTCTTGTGGACGCACTCGTCGCAACCGCGGCAAGACGTAG

XAC29_00960|470469929_c231889-231329 --------------------------------------------------

XCAW_00583|471265562_c692244-691666 --------------------------------------------------

XAC0187|21240774_c230734-230156 -------------------------------------C------------

PXO_03586|188574270_c286007-284730 AAACCTTGTTCCAGGCCCTGGTCTTCAATTACCTGATCGGCGGCACCGAT

XOC_0274|384417079_c270047-268770 AAACCTTGTTCCAGGCCCTGGTCTTCAATTACCTGATCGGCGGCACCGAT

XAC29_00960|470469929_c231889-231329 --------------------------------------------------

XCAW_00583|471265562_c692244-691666 -------------------------------------C------------

XAC0187|21240774_c230734-230156 --------------------------------------------------

PXO_03586|188574270_c286007-284730 GCGCATGCGAAGAACTATTCGCTACTGCTCAGCCAACACCGGCAGGTACG

XOC_0274|384417079_c270047-268770 GCGCATGCGAAGAACTATTCGCTACTGCTCAGCCAACACCGGCAGGTACG

XAC29_00960|470469929_c231889-231329 --------------------------------------------------

XCAW_00583|471265562_c692244-691666 --------------------------------------------------

XAC0187|21240774_c230734-230156 -------CCGTT--AC----------------------------------

PXO_03586|188574270_c286007-284730 GCTGGCGCCGTTGTACGACATTTCCAGCGCCCTGCCGTATCCGCAGCTGC

XOC_0274|384417079_c270047-268770 GCTGGCGCCGTTGTACGACATTTCCAGCGCCCTGCCGTATCCGCACCTGC

XAC29_00960|470469929_c231889-231329 --------------------------------------------------

XCAW_00583|471265562_c692244-691666 -------CCGTT--AC----------------------------------

XAC0187|21240774_c230734-230156 ---A----------------------------------------------

PXO_03586|188574270_c286007-284730 AACAGCGCAAAATCAAGATGGCAATGAAGATCGGCAGCCACTATCGCTGG

XOC_0274|384417079_c270047-268770 AACAGCGCAAAATCAAGATGGCAATGAAGATCGGCAGCCACTATCGCTGG

XAC29_00960|470469929_c231889-231329 --------------------------------------------------

XCAW_00583|471265562_c692244-691666 ---A----------------------------------------------

XAC0187|21240774_c230734-230156 --------------------------------------------------

PXO_03586|188574270_c286007-284730 TGGGATATTCGGCCAGGCGACTGGCGGCAACTGGCCGAAACGCTGCATCT

XOC_0274|384417079_c270047-268770 TGGGATATTCGGCCAGGCGACTGGCGGCAACTGGCCGAAACGCTGCATCT

XAC29_00960|470469929_c231889-231329 --------------------------------------------------

XCAW_00583|471265562_c692244-691666 --------------------------------------------------

XAC0187|21240774_c230734-230156 --------------------------------------------------

PXO_03586|188574270_c286007-284730 GGACGCACCGGCCTGCCTGGCCTGGATGGCGATGGCTGCAAGCGCCTTGC

XOC_0274|384417079_c270047-268770 GGACGCACCGGCCTGCCTGGCCTGGATGGCGATGGCTGCAAGCGCCTTGC

XAC29_00960|470469929_c231889-231329 --------------------------------------------------

XCAW_00583|471265562_c692244-691666 --------------------------------------------------

XAC0187|21240774_c230734-230156 --------------------------------------------------

PXO_03586|188574270_c286007-284730 CCGATCAGGCGCGTGATCTTGCCGTCGCACTTCGAGAGGACGGCGTGGCG

XOC_0274|384417079_c270047-268770 CCGATCAGGCGCGTGATCTTGCCGTCGCACTTCGAGAGGACGGCGTGGCG

XAC29_00960|470469929_c231889-231329 --------------------------------------------------

XCAW_00583|471265562_c692244-691666 --------------------------------------------------

XAC0187|21240774_c230734-230156 CA-----------------TGG----------------------------

PXO_03586|188574270_c286007-284730 CATCCGGTCCTGGATGCACTGGTGGACGCCATCGCCGCTTCCTGTCAACG

XOC_0274|384417079_c270047-268770 CATCCGGTCCTGGATGCACTGGTGGACGCCATCGCCGCTTCGTGCCAACG

XAC29_00960|470469929_c231889-231329 --------------------------------------------------

XCAW_00583|471265562_c692244-691666 CA-----------------TGG----------------------------

XAC0187|21240774_c230734-230156 -------------------------TAG

PXO_03586|188574270_c286007-284730 CACGCGCACACTATTGCAATCGAACTGA

XOC_0274|384417079_c270047-268770 CACGCGCACACTATTGCAATCGAACTGA

XAC29_00960|470469929_c231889-231329 -------------------------GAA

XCAW_00583|471265562_c692244-691666 -------------------------TAG

PROTEIN ALIGNMENTS:

XAC0187|21106245|gb|AAM35079.1| MRLVALIENQTIGHLASDPHGHPRFTYAPDWQHSKSAIPLSLNLPLSREH

PXO_03586|188518992|gb|ACD56937.1| MRLVALIENQVIGHLAPDQHGHPRFTYAPDWQHSKNAIPLSLNLPLSKEH

XOC_0274|353460234|gb|AEQ94513.1| MRLVALIENQVIGHLTPDQHGHPRFTYAPDWQHSKNAIPLSLNLPLSKEH

XCAW_00583|471209652|gb|AGI06402.1| MRLVALIENQTIGHLASDPHGHPRFTYAPDWQHSKSAIPLSLNLPLSREH

**********.****:.* ****************.***********:**

XAC0187|21106245|gb|AAM35079.1| HPTEAVSAVLWGLLPDNEATLQRWASRFQVSPRNPLALLSHVGEDCAGAV

PXO_03586|188518992|gb|ACD56937.1| HPTEAVSAVLWGLLPDNEATLQRWASRFQVSPRNPLALLSHVGEDCAGAV

XOC_0274|353460234|gb|AEQ94513.1| HPTEAVSAVLWGLLPDNEATLQRWASRFQVSPRNPLALLSHVGEDCAGAV

XCAW_00583|471209652|gb|AGI06402.1| HPTEAVSAVLWGLLPDNEATLQRWASRFQVSPRNPLALLSHVGEDCAGAV

**************************************************

XAC0187|21106245|gb|AAM35079.1| QFVTEARLEDVLSGASDGIEPLTDGELEARLRRLRQDIGAARQVEDVGQF

PXO_03586|188518992|gb|ACD56937.1| QFVTEARLEDVLSGASDGIEPLTDGELEARLRRLRQDIGAARQVDDVGQF

XOC_0274|353460234|gb|AEQ94513.1| QFVTEARLEDVLSGASDGIEPLTDGELEARLRRLRQDIGAARQVDDVGQF

XCAW_00583|471209652|gb|AGI06402.1| QFVTEARLEDVLSGASDGIEPLTDGELEARLRRLRQDIGAARQVDDVGQF

********************************************:*****

XAC0187|21106245|gb|AAM35079.1| SLAGAQAKIALLHDGLQWAIPAGRIPTTHILKPPSGE-------------

PXO_03586|188518992|gb|ACD56937.1| SLAGAQAKIALLHDGLQWAIPAGRIPTTHILKPPSGEYDGFVENELFCLC

XOC_0274|353460234|gb|AEQ94513.1| SLAGAQAKIALLHDGLQWAIPAGRIPTTHILKPPSGEYDGFVENELFCLC

XCAW_00583|471209652|gb|AGI06402.1| SLAGAQAKIALLHDGLQWAIPAGRIPTTHILKPPSGE-------------

*************************************

XAC0187|21106245|gb|AAM35079.1| --------------------------------------------------

PXO_03586|188518992|gb|ACD56937.1| LAHSIGLPTATAQALRVGAETAICVKRYDRTGVNGVLLRIHQEDTCQALG

XOC_0274|353460234|gb|AEQ94513.1| LAHSIGLPTATAQALRVGAETAICVKRYDRTGVNGVLLRIHQEDTCQALG

XCAW_00583|471209652|gb|AGI06402.1| --------------------------------------------------

XAC0187|21106245|gb|AAM35079.1| --------------------------------------------------

PXO_03586|188518992|gb|ACD56937.1| VMPHIKYQNQGGPGVEAISQLLWTHSSQPRQDVETLFQALVFNYLIGGTD

XOC_0274|353460234|gb|AEQ94513.1| VMPHIKYQNQGGPGVEAISQLLWTHSSQPRQDVETLFQALVFNYLIGGTD

XCAW_00583|471209652|gb|AGI06402.1| --------------------------------------------------

XAC0187|21106245|gb|AAM35079.1| ---------------------------------------------SRYTW

PXO_03586|188518992|gb|ACD56937.1| AHAKNYSLLLSQHRQVRLAPLYDISSALPYPQLQQRKIKMAMKIGSHYRW

XOC_0274|353460234|gb|AEQ94513.1| AHAKNYSLLLSQHRQVRLAPLYDISSALPYPHLQQRKIKMAMKIGSHYRW

XCAW_00583|471209652|gb|AGI06402.1| ---------------------------------------------SRYTW

*:* *

XAC0187|21106245|gb|AAM35079.1| --------------------------------------------------

PXO_03586|188518992|gb|ACD56937.1| WDIRPGDWRQLAETLHLDAPACLAWMAMAASALPDQARDLAVALREDGVA

XOC_0274|353460234|gb|AEQ94513.1| WDIRPGDWRQLAETLHLDAPACLAWMAMAASALPDQARDLAVALREDGVA

XCAW_00583|471209652|gb|AGI06402.1| --------------------------------------------------

XAC0187|21106245|gb|AAM35079.1| -------------------------

PXO_03586|188518992|gb|ACD56937.1| HPVLDALVDAIAASCQRTRTLLQSN

XOC_0274|353460234|gb|AEQ94513.1| HPVLDALVDAIAASCQRTRTLLQSN

XCAW_00583|471209652|gb|AGI06402.1| -------------------------

**15 - DOMAIN cd00093 - HTH_XRE**

**cd00093 - HTH_XRE (1)**

NUCLEOTIDE ALIGNMENTS:

XAC1499|21240774_1733523-1733819 ATGAAGAAGCGGAGGAAGACACTTCGGCTCAGCCAAAAGGCCTTAGGAGT

XAC29_07580|470469929_1737365-1737661 ATGAAGAAGCGGAGGAAGACACTTCGGCTCAGCCAAAAGGCCTTAGGAGT

XCAW_02829|471265562_c3164354-3164058 ATGAAGAAGCGGAGGAAGACACTTCGGCTCAGCCAAAAGGCCTTAGGAGT

**************************************************

XAC1499|21240774_1733523-1733819 GCTCATGGGACTACCAGAAGATGTCGCAGGCGTTCGCATCAATCGATACG

XAC29_07580|470469929_1737365-1737661 GCTCATGGGACTACCAGAAGATGTCGCAGGCGTTCGCATCAATCGATACG

XCAW_02829|471265562_c3164354-3164058 GCTCATGGGACTACCAGAAGATGTCGCAGGCGTTCGCATCAATCGATACG

**************************************************

XAC1499|21240774_1733523-1733819 AGCGGGCAGTGCATGAGTGCGATAGTGAGACGGCTCAGAAAATGGCAGAC

XAC29_07580|470469929_1737365-1737661 AGCGGGCAGTGCATGAGTGCGATAGTGAGACGGCTCAGAAAATGGCAGAC

XCAW_02829|471265562_c3164354-3164058 AGCGGGCAGTGCATGAGTGCGATAGTGAGACGGCTCAGAAAATGGCAGAC

**************************************************

XAC1499|21240774_1733523-1733819 GCTCTTGGCGTCTCGCTTGCCTACCTTTATGCAGAGACAGACGAACTGGC

XAC29_07580|470469929_1737365-1737661 GCTCTTGGCGTCTCGCTTGCCTACCTTTATGCAGAGACAGACGAACTGGC

XCAW_02829|471265562_c3164354-3164058 GCTCTTGGCGTCTCGCTTGCCTACCTTTATGCAGAGACAGACGAACTGGC

**************************************************

XAC1499|21240774_1733523-1733819 AGAACTCATCCAGGAGTTCATGCAGCTTCCCGCCAAAGACCAGCACGCGC

XAC29_07580|470469929_1737365-1737661 AGAACTCATCCAGGAGTTCATGCAGCTTCCCGCCAAAGACCAGCACGCGC

XCAW_02829|471265562_c3164354-3164058 AGAACTCATCCAGGAGTTCATGCAGCTTCCCGCCAAAGACCAGCACGCGC

**************************************************

XAC1499|21240774_1733523-1733819 TCTTGGCTGATATCAAGTCTCGCTCCAAGACAAATCGTGTGCGCTAA

XAC29_07580|470469929_1737365-1737661 TCTTGGCTGATATCAAGTCTCGCTCCAAGACAAATCGTGTGCGCTAA

XCAW_02829|471265562_c3164354-3164058 TCTTGGCTGATATCAAGTCTCGCTCCAAGACAAATCGTGTGCGCTAA

***********************************************

PROTEIN ALIGNMENTS:

XAC1499|AAM36370.1|transcriptional... MKKRRKTLRLSQKALGVLMGLPEDVAGVRINRYERAVHECDSETAQKMAD

XAC29_07580|WP_011050959.1|transcr... MKKRRKTLRLSQKALGVLMGLPEDVAGVRINRYERAVHECDSETAQKMAD

XCAW_02829|AGI08606.1|Hypothetical... MKKRRKTLRLSQKALGVLMGLPEDVAGVRINRYERAVHECDSETAQKMAD

**************************************************

XAC1499|AAM36370.1|transcriptional... ALGVSLAYLYAETDELAELIQEFMQLPAKDQHALLADIKSRSKTNRVR

XAC29_07580|WP_011050959.1|transcr... ALGVSLAYLYAETDELAELIQEFMQLPAKDQHALLADIKSRSKTNRVR

XCAW_02829|AGI08606.1|Hypothetical... ALGVSLAYLYAETDELAELIQEFMQLPAKDQHALLADIKSRSKTNRVR

************************************************

**cd00093 - HTH_XRE (2)**

NUCLEOTIDE ALIGNMENTS:

XCC1604|21229478_1874502-1874843 ATGCCC-----AAACC-------------T---AAGACACCGGCCACGGT

XAC3288|21240774_c3868212-3867874 ATGCCC-----AAACC-------------C---AAGACACCGGCCACGGT

XC_2631|66766352_c3167763-3167422 ATGCCC-----AAACC-------------T---AAGACACCGGCCACGGT

XCV1100|78045556_1234982-1235338 GTGTTTTCGGTACACCCCCCAATGCCTCCCATCAAGCCGCCTGCATCACT

XAC29_16760|470469929_c3875749-3875411 ATGCCC-----AAACC-------------C---AAGACACCGGCCACGGT

XCAW_01037|471265562_1197624-1197965 ATGCCC-----GCATC-------------T---CTGCCCACAAGTTCCGT

** * * * * * * *

XCC1604|21229478_1874502-1874843 CTATGGCACGCGTCTGCGCCATGCGCGGATGGCCATGGGCTGGACACAAG

XAC3288|21240774_c3868212-3867874 CTATGGCACGCGTCTGCGCAATGCGCGGATGGCCATGGGCTGGACACAAG

XC_2631|66766352_c3167763-3167422 CTATGGCACGCGTCTGCGCCATGCGCGGATGGCCATGGGCTGGACACAAG

XCV1100|78045556_1234982-1235338 GTTCGGACGACGATTGCGCACGGCCCGGCTGTCCTTGGGCCTGCGTCAAG

XAC29_16760|470469929_c3875749-3875411 CTATGGCACGCGTCTGCGCAATGCGCGGATGGCCATGGGCTGGACACAAG

XCAW_01037|471265562_1197624-1197965 CTTTGGTCGTCGCCTGCGTGCGGCCAGGCGCGTGGCGGGCCTGTCGCAAG

* ** ** **** ** ** **** * ****

XCC1604|21229478_1874502-1874843 CCGAGCTGGCGGAGCGCATTGGG-ATGGTGGACTCGGTGTCGGGAGCCAC

XAC3288|21240774_c3868212-3867874 CCGAGCTGGCGGAGCGCATTGGG-ATGGTGGACTCGGTGTCGGGAGCCAC

XC_2631|66766352_c3167763-3167422 CCGAGCTGGCGGAGCGCATTGGG-ATGGTGGACTCGGTGTCGGGAGCCAC

XCV1100|78045556_1234982-1235338 CAGACTTGG-GAAGGATGTTGGGAATGGAAGAGCAAAACACCGGTGCACC

XAC29_16760|470469929_c3875749-3875411 CCGAGCTGGCGGAGCGCATTGGG-ATGGTGGACTCGGTGTCGGGAGCCAC

XCAW_01037|471265562_1197624-1197965 TCGAACTGGGAGCGCTGCTGGGG-ATGAACGAGAAA---GCTGCCTCGTC

** *** * * *** *** ** * * * *

XCC1604|21229478_1874502-1874843 GCGCGTGAGCCGCTACGAGACGGGGCAACATGACCCGGACCCTGCGACGG

XAC3288|21240774_c3868212-3867874 TCGGGTGAGCCGCTACGAGACGGGGCAACATGACCCGGACCCTGCGACCG

XC_2631|66766352_c3167763-3167422 GCGCGTGAGCCGCTACGAGACGGGGCAACATGACCCGGACCCTGCGACGG

XCV1100|78045556_1234982-1235338 TCGCATCTCCCGCTACGAGACGGGGCAACACGATCCGGATCCTGAAACCG

XAC29_16760|470469929_c3875749-3875411 TCGGGTGAGCCGCTACGAGACGGGGCAACATGACCCGGACCCTGCGACCG

XCAW_01037|471265562_1197624-1197965 ACGACTCTCCCGCTACGAGCGGGGGGAGCGAGAGCCCGGCCATGAGACGT

** * ********** **** * * ** ** * * ** **

XCC1604|21229478_1874502-1874843 CTGAGGCACTAGCGAAGGCGTTGAAGCTGCCTGTGGCCTATTTCCACGCA

XAC3288|21240774_c3868212-3867874 CTGAGGCCTTAGCAAAGGCGCTGGACTTGCCGGTGGCCTACTTTCATGCG

XC_2631|66766352_c3167763-3167422 CTGAGGCACTAGCGAAGGCGTTGAAGCTGCCTGTGGCCTATTTCCACGCA

XCV1100|78045556_1234982-1235338 CAGCAGAACTGGCTCAGGCGTTGGGTTTGCCGTTGGCGTACTTTTACGCA

XAC29_16760|470469929_c3875749-3875411 CTGAGGCCTTAGCAAAGGCGCTGGACTTGCCGGTGGCCTACTTTCATGCG

XCAW_01037|471265562_1197624-1197965 TGGCTGCTTTGAGCAACGCGCTGGGTGTTCCGCCGGCCTACTTTCACGCC

* * * * *** ** * ** *** ** ** * **

XCC1604|21229478_1874502-1874843 ACGTCAGACGTGCTGGCCGACATCATCTTGATTGTGTCGCGGTTGCCCGT

XAC3288|21240774_c3868212-3867874 ACGTCGGACCTTCTGGCCGAGGTGATTCTTGTGGTGTCCCAACTGCCGGC

XC_2631|66766352_c3167763-3167422 ACGTCAGACGTGCTGGCCGACATCATCTTAATTGTGTCGCGGTTGCCCGT

XCV1100|78045556_1234982-1235338 ACACCCGACATGCTCGCCGAGGCCATTTTGTTGATCGCGAAGTTGCCCGA

XAC29_16760|470469929_c3875749-3875411 ACGTCGGACCTTCTGGCCGAGGTGATTCTTGTGGTGTCCCAACTGCCGGC

XCAW_01037|471265562_1197624-1197965 AGCTCTGACGTGTTGGCCGAGGTCATACTGCTTGTGGCTCGTCTCCCCGC

* * *** * * ***** ** * * * * * ** *

XCC1604|21229478_1874502-1874843 CGCGAAGCAGAAGGAGGCCTTGGAGCGACTCAAGGATC--TTGCAGGC-A

XAC3288|21240774_c3868212-3867874 GGCAAAGCAGCGCGAGGCTCTGAACCTTGTGAAGGA-C--TTGCTCCCCA

XC_2631|66766352_c3167763-3167422 CGCGAAGCAGAAGGAGGCCTTGGAGCGACTCAAGGATC--TTGCAGGC-A

XCV1100|78045556_1234982-1235338 GGATCGTCAGCAGGAGGCGATAGC-CGCCTTGAGAGCC-ATTGCCGAG-A

XAC29_16760|470469929_c3875749-3875411 GGCAAAGCAGCGCGAGGCTCTGAACCTTGTGAAGGA-C--TTGCTCCCCA

XCAW_01037|471265562_1197624-1197965 GGAGCGTCAACAGACAGTGCTTGACCTAATCAAGGAGCATTTGACCTCGA

* ** * * * * ** * *** *

XCC1604|21229478_1874502-1874843 -AGCACTCCGGCGAATAA

XAC3288|21240774_c3868212-3867874 -CGCAG--CATCG-CTGA

XC_2631|66766352_c3167763-3167422 -AGCACTCCGGCGAATAA

XCV1100|78045556_1234982-1235338 ----AG--AAGGG-TTGA

XAC29_16760|470469929_c3875749-3875411 -CGCAG--CATCG-CTGA

XCAW_01037|471265562_1197624-1197965 CAGCGCCTTCGCG-CTAG

* *

PROTEIN ALIGNMENTS:

XCC1604|21229478_1874502-1874843 -------VAYFHATSDVLADIILIVSRLPVAKQKEALERLKDLAGKHSG-

XAC3288|21240774_c3868212-3867874 -------VAYFHATSDLLAEVILVVSQLPAAKQREALNLVKDLLPTQH--

XC_2631|66766352_c3167763-3167422 -------VAYFHATSDVLADIILIVSRLPVAKQKEALERLKDLAGKHSG-

XCV1100|CAJ22731.1|putative AQALGLPLAYFYATPDMLAEAILLIAKLPEDRQQEAIAALRAIAEKK---

XAC29_16760|AGH78771.1|hypothetical -------VAYFHATSDLLAEVILVVSQLPAAKQREALNLVKDLLPTQH--

XCAW_01037|AGI06849.1|Transcriptional --------AYFHASSDVLAEVILLVARLPAERQQTVLDLIKEHLTSTAPS

***:*:.*:**: **::::** :*: .: :: .

XCC1604|21229478_1874502-1874843 E

XAC3288|21240774_c3868212-3867874 R

XC_2631|66766352_c3167763-3167422 E

XCV1100|CAJ22731.1|putative G

XAC29_16760|AGH78771.1|hypothetical R

XCAW_01037|AGI06849.1|Transcriptional R

**cd00093 - HTH_XRE (3**)

NUCLEOTIDE ALIGNMENTS:

XCV2163|78045556_c2485161-2484823 ATGGGCAAAGAGGAAACCGAAAAAGTGGATATGGAACATTGGCTGCATGA

XOC_2640|384417079_c2715911-2715606 ATGACCA-AGAC--AACCACGACACCG--T-TCGACC--CAGCC----GA

*** ** *** **** * * * * * ** * ** **

XCV2163|78045556_c2485161-2484823 ACGAATCGGCCCAGCCTACGATGCGTTGAAGGCCGACCCAGGCCGCGCCG

XOC_2640|384417079_c2715911-2715606 ATACCTCGAC---GATGACGAGGCAATGACAACCTACCTC-TCCGAAGCA

* *** * * **** ** *** ** *** *** *

XCV2163|78045556_c2485161-2484823 TTACCGCTGACCAGGTGCGCGCCCGTCTGGCTGACATCGCCCAAGCTATC

XOC_2640|384417079_c2715911-2715606 CT-CCATTCTGGAGATAC-CGACCATTTCCAAGACACTCTGCAGGTCGTG

* ** * ** * * ** ** * * **** ** * *

XCV2163|78045556_c2485161-2484823 GACCTGGCCCGCCGCGTCGATGCGGACCTAGAATCCCTTACGCCCTTTGA

XOC_2640|384417079_c2715911-2715606 GCGCGTGCACGCGGCATG-GCGC------AGA-TTGCTGAGGCTGCAGGG

* * ** *** ** * ** *** * ** * ** *

XCV2163|78045556_c2485161-2484823 CCCGGCCG-AAGA-CCTG-ACCA-GCGCCGAAGCCGTGGCCGCATTCTTG

XOC_2640|384417079_c2715911-2715606 CTTGGCCGGGAGAGCCTATACAAGGCACTCAAGCCTGGCGCGAATCC---

* ***** *** *** ** * ** * ***** * ** ** *

XCV2163|78045556_c2485161-2484823 GCGGACGCCGAGGCCACGGCCGACCCGGCCTATATCGAGCACGCGCACCA

XOC_2640|384417079_c2715911-2715606 GC--GCTTTGACACCA--TCCAGCGCGTCCTG-ACTGCGCTTG-GCGTCC

** * ** *** ** * ** *** * * ** * ** *

XCV2163|78045556_c2485161-2484823 GGTCGCGGCACGTGCGCGAACCATGCACGGCATCGCGTCGTGA

XOC_2640|384417079_c2715911-2715606 GGCTG-AGCATCGACCCGGTCGATA-ACGTCAAAGCGGCCTGA

** * *** * ** * ** *** ** *** * ***

Protein alignments:

XCV2163|CAJ23840.1|conserved MGKEETEKVDMEHWLHERIGPAYDALKADPGRAVTADQVRARLADIAQAI

XOC_2640|AEQ96750.1|putative MTKT----------------------------------------------

* *

XCV2163|CAJ23840.1|conserved DLARRVDADLESLTPFDPAEDLTSAEAVAAFLADAEATADPAYIEHAHQV

XOC_2640|AEQ96750.1|putative -----------TTTPFDPAEYLDDDEAMTTYLSEALHSGDTDHFQDTLQV

: ******* * . **::::*::* :.*. :::.: **

XCV2163|CAJ23840.1|conserved AARARTMHGIAS--------------------------------------

XOC_2640|AEQ96750.1|putative VARARGMAQIAEAAGLGRESLYKALKPGANPRFDTIQRVLTALGVRLSID

.**** * **.

XCV2163|CAJ23840.1|conserved --------

XOC_2640|AEQ96750.1|putative PVDNVKAA

cd00093 - HTH_XRE (4)

Nucleotide alignments:

XCC4197|21229478_c5006644-5006354 ATGAC-AG--TCCTACCCAACATCCACCCCGGCGAAATCCTGCTTGAGGA

XC_4286|66766352_c5079329-5079039 ATGAC-AG--TCCTACCCAACATCCACCCCGGCGAAATCCTGCTTGAGGA

XCV4439|78045556_c5115748-5115458 ATGAC-GG--TCCTACCCAATATCCACCCTGGCGAAATTCTGCTTGAGGA

PXO_03904|188574270_334586-334867 GTGATCAGGAGCTTTATCGACAAGGACGCCGAAAAGATCTGGCTGGGTGA

XCR_4538|384425691_c4870890-4870600 ATGAC-AG--TCCTACCCAACATCCACGCCGGCGAAATCCTGCTTGAGGA

XOC_0101|384417079_c99858-99568 ATGAC-GG--TCCTACCCAATATCCATCCTGGCGACATTCTGCTTGAGGA

*** * * * * * * * * * * ** *** * **

XCC4197|21229478_c5006644-5006354 GTTCCTCGAGCCGATGGGCATCAGCCAGAACGCAC-TGG-CGCGGGCCAC

XC_4286|66766352_c5079329-5079039 GTTCCTCGAGCCGATGGGCATCAGCCAGAACGCAC-TGG-CGCGGGCCAC

XCV4439|78045556_c5115748-5115458 GTTACTCGAGCCTCTGGGCATCAGCCAGAACGCAC-TGG-CACGCGCCAC

PXO_03904|188574270_334586-334867 GCG-TTCCCGCCGGTTGC---CTGCCGACATCCAATTGGTCGCGCGCCGC

XCR_4538|384425691_c4870890-4870600 GTTCCTCGAGCCGATGGGCATCAGCCAGAACGCAC-TGG-CGCGGGCCAC

XOC_0101|384417079_c99858-99568 GTTTCTCGAGCCTCTGGGCATCAGCCAGAACGCAC-TGG-CACGCGCCAC

* ** *** * * * *** * ** *** * ** *** *

XCC4197|21229478_c5006644-5006354 CGATGTACCGCCGCGCCGCATCAACGAGATCGTGCTGGGCAAGCGTGGCA

XC_4286|66766352_c5079329-5079039 CGATGTACCGCCGCGCCGCATCAACGAGATCGTGCTGGGCAAGCGTGGCA

XCV4439|78045556_c5115748-5115458 CGGCGTCCCGCCGCGCGGTATCAACGAAATCGTGCTAGGCAAGCGCGGCA

PXO_03904|188574270_334586-334867 --AAGT-----TGCGCATGCTCAATGCC-----GCCGCGCAT-CTCGACG

XCR_4538|384425691_c4870890-4870600 CGATGTACCGCCGCGCCGCATCAACGAGATCGTGCTGGGCAAGCGTGGCA

XOC_0101|384417079_c99858-99568 CGGCGTCCCGCCGCGCCGTATCAACGAAATCGTGCTGGGCAAGCGCGGCA

** **** **** * ** *** * * *

XCC4197|21229478_c5006644-5006354 TCACCGCCGACACCGCCGTGCGTCTGGCTG-CAGCGTTCGGC-ACGACCG

XC_4286|66766352_c5079329-5079039 TCACCGCCGACACCGCCGTGCGTCTGGCTG-CAGCGTTCGGC-ACGACCG

XCV4439|78045556_c5115748-5115458 TCACTGCCGACACTGCCATGCGTCTGGCCG-CCGCGTTGGGA-ACCTCCG

PXO_03904|188574270_334586-334867 A-TCTGCGGATCCCGCCCGCCAACCGGCTGGAAGCGTTGAAGGGCCAGCG

XCR_4538|384425691_c4870890-4870600 TCACCGCCGACACCGCCGTGCGCCTGGCCG-CGGCGCTGGGC-ACGACCG

XOC_0101|384417079_c99858-99568 TCACTGCCGACACTGCCGTGCGTCTGGCCG-CCGCGTTGGGC-ACCTCCG

* ** ** * *** * * *** * *** * * **

XCC4197|21229478_c5006644-5006354 AGCGCTGCTGGCTGGGCCTGCAAGCCGATTACGAGCTCGAAGAGGCGCAC

XC_4286|66766352_c5079329-5079039 AGCGCTGCTGGCTGGGCCTGCAAGCCGATTACGAGCTCGAAGAGGCGCAC

XCV4439|78045556_c5115748-5115458 AGCGCTTCTGGCTTGGCCTGCAGGCCGACGACGAACTGGAGCAGGCGCAT

PXO_03904|188574270_334586-334867 -GCGCGGCCAGTACAGCATCCGGATCAACGATCAA-TGGCGCATCTGCTT

XCR_4538|384425691_c4870890-4870600 AGCGCTTCTGGCTGGGCCTGCAGGCCGATTACGAGCTGGAACAGGCGTAC

XOC_0101|384417079_c99858-99568 AGCGCTTCTGGCTGGGCCTGCAGGCCGACTACGAGCTGGAACAGGCGCAT

**** * * ** * * * * * * * * * *

XCC4197|21229478_c5006644-5006354 C-GCGCGCTG---GGCGATCTGCCGTCACGGATCAAGCGGCTGGCGGC--

XC_4286|66766352_c5079329-5079039 C-GCGCGCTG---GGCGATCTGCCGTCACGGATCAAGCGGCTGGCGGC--

XCV4439|78045556_c5115748-5115458 C-GCGCGCTG---GGCGACTTGCCGGCGCGGATCAAGCGGCTGGCGGC--

PXO_03904|188574270_334586-334867 CCGATGGATGGAAGGCGATGTG--GTCCAGGTCGAAATCGTCGATTACCA

XCR_4538|384425691_c4870890-4870600 C-GCGCGCTG---GGCGATCTGCCGTCACGGATCAAGCGGCTGGCGGC--

XOC_0101|384417079_c99858-99568 C-GCGCGCTG---GGCGATTTGCCGGCACGGATCAAGCGGCTGGCGGC--

* * * ** ***** ** * * ** ** * * *

XCC4197|21229478_c5006644-5006354 TTGA

XC_4286|66766352_c5079329-5079039 TTGA

XCV4439|78045556_c5115748-5115458 ATGA

PXO_03904|188574270_334586-334867 CTGA

XCR_4538|384425691_c4870890-4870600 TTGA

XOC_0101|384417079_c99858-99568 GTGA

***

PROTEIN ALIGNMENTS:

XCC4197|AAM43413.1|virulence MTV-LPNIHPGEILLEEFLEP-------------MGISQNALARATDVPP

XC_4286|AAY51321.1|virulence MTV-LPNIHPGEILLEEFLEP-------------MGISQNALARATDVPP

XCV4439|CAJ26170.1|putative MTV-LPNIHPGEILLEELLEP-------------LGISQNALARATGVPP

PXO_03904|ACD56975.1|plasmid MIRSFIDKDAEKIWLGERSRRLPADIQLVARRKLRMLNAAAHLDDLRIPP

XCR_4538|AEL09389.1|addiction MTV-LPNIHAGEILLEEFLEP-------------MGISQNALARATDVPP

XOC_0101|AEQ94359.1|addiction MTV-LPNIHPGDILLEEFLEP-------------LGISQNALARATGVPP

* : : .. .* * * . :. * :**

XCC4197|AAM43413.1|virulence RRINEIVLGKRGITADTAVRLAAAFGTTERCWLGLQADYELEEAHRALGD

XC_4286|AAY51321.1|virulence RRINEIVLGKRGITADTAVRLAAAFGTTERCWLGLQADYELEEAHRALGD

XCV4439|CAJ26170.1|putative RGINEIVLGKRGITADTAMRLAAALGTSERFWLGLQADDELEQAHRALGD

PXO_03904|ACD56975.1|plasmid ANRLEALKGQRR--GQYSIRINDQWRICFRWMEGDVVQVEIVDYH-----

XCR_4538|AEL09389.1|addiction RRINEIVLGKRGITADTAVRLAAALGTTERFWLGLQADYELEQAYRALGD

XOC_0101|AEQ94359.1|addiction RRINEIVLGKRGITADTAVRLAAALGTSERFWLGLQADYELEQAHRALGD

* : *:* .: ::*: * * .: *: : :

XCC4197|AAM43413.1|virulence LPSRIKRLAA

XC_4286|AAY51321.1|virulence LPSRIKRLAA

XCV4439|CAJ26170.1|putative LPARIKRLAA

PXO_03904|ACD56975.1|plasmid ----------

XCR_4538|AEL09389.1|addiction LPSRIKRLAA

XOC_0101|AEQ94359.1|addiction LPARIKRLAA

**16 - DOMAIN pfam02661 - Fic/DOC**

NUCLEOTIDE ALIGNMENTS:

XAC1501|21240774_1733943-1735031 TTGTCAGGGCATGTTGCCCGGGCTTCGTCAGTGCGTCGAAATTTTATGGA

XCAW_02828|471265562_c3163934-3162846 TTGTCAGGGCATGTTGCCCGGGCTTCGTCAGTGCGTCGAAATTTTATGGA

**************************************************

XAC1501|21240774_1733943-1735031 TGGAAATTTTGAGTTAGCGCCGCTTGATTTGGCCTTTGCGCCACTTCCAG

XCAW_02828|471265562_c3163934-3162846 TGGAAATTTTGAGTTAGCGCCGCTTGATTTGGCCTTTGCGCCACTTCCAG

**************************************************

XAC1501|21240774_1733943-1735031 GACGACGCGATGCGCCTGACGCTTCTGTTTGGCTCGATGCGTCCTTTTCG

XCAW_02828|471265562_c3163934-3162846 GACGACGCGATGCGCCTGACGCTTCTGTTTGGCTCGATGCGTCCTTTTCG

**************************************************

XAC1501|21240774_1733943-1735031 GTCTATACGCCAGCGCTGTTGGAGCCACCACTTCGAACCCATGAAAAAAT

XCAW_02828|471265562_c3163934-3162846 GTCTATACGCCAGCGCTGTTGGAGCCACCACTTCGAACCCATGAAAAAAT

**************************************************

XAC1501|21240774_1733943-1735031 AGGGGATTACCAACGCTTTGAGTCAGATGCACAATGCGTCGGTTACCTTT

XCAW_02828|471265562_c3163934-3162846 AGGGGATTACCAACGCTTTGAGTCAGATGCACAATGCGTCGGTTACCTTT

**************************************************

XAC1501|21240774_1733943-1735031 CAGCCATATCTTCCAGGCTAATTCACGTATGCAGAGGGAGTTGGAAGTTC

XCAW_02828|471265562_c3163934-3162846 CAGCCATGTCTTCCAGGCTAATTCACGTATGCAGAGGGAGTTGGAAGTTC

******* ******************************************

XAC1501|21240774_1733943-1735031 GCCTCGATTCCTGATTTGAGGGCAAGTCGCCAAGCGGCGTCAGCCATCAA

XCAW_02828|471265562_c3163934-3162846 GCCTCGATTCCTGATTTGAGGGCAAGTCGCCAAACGGCGTCAGCCATCAA

********************************* ****************

XAC1501|21240774_1733943-1735031 TGCGTCCCTCAGAGCAGGACTTCCAGATACGCATGGTATTTTAAATTGGT

XCAW_02828|471265562_c3163934-3162846 TGCGTCCCTCAGAGCAGGACTTCCAGATACGCATGGTATTTTAAATTGGT

**************************************************

XAC1501|21240774_1733943-1735031 CATTAAATTTGGCCAAAAATTTAGGCAATAGCCAAGTCAGAAGCCGTACA

XCAW_02828|471265562_c3163934-3162846 CATTAAATTTGGCCAAAAATTTAGGCAATAGCCAAGTCAGAAGCCGTACA

**************************************************

XAC1501|21240774_1733943-1735031 GATAATGCATGGGTTGGTGGTAAGAAACCGTCAGAAGCGTGGCTTGTTTT

XCAW_02828|471265562_c3163934-3162846 GATAATGCATGGGTTGGTGGTAAGAAACCGTCAGAAGCGTGGCTTGTTTT

**************************************************

XAC1501|21240774_1733943-1735031 ACCGCAGGCAGATCAACTGCCTTCTCTTTTGAAAAACCTTGAAGATTTGC

XCAW_02828|471265562_c3163934-3162846 ACCGCAGGCAGATCAACTGCCTTCTCTTTTGAAAAACCTTGAAGATTTGC

**************************************************

XAC1501|21240774_1733943-1735031 TACAAGGCAAGAGCGCCCAGCCGGTCAACTGCGCATTTTTCGAAGCCATT

XCAW_02828|471265562_c3163934-3162846 TACAAGGCAAGAGCGCCCAGCCGGTCAACTGCGCATTTTTCGAAGCCATT

**************************************************

XAC1501|21240774_1733943-1735031 GCGTATCAGATAATCTGTATCCATCCGCTCAGTGACACCAATGGACGGGT

XCAW_02828|471265562_c3163934-3162846 GCGTATCAGATAATCTGTATCCATCCGCTCAGTGACACCAATGGACGGGT

**************************************************

XAC1501|21240774_1733943-1735031 GACTCGCACTCTCTTGATTAATCTTGCGGGACGCTATCGAAATTTTTATC

XCAW_02828|471265562_c3163934-3162846 GACTCGCACTCTCTTGATTAATCTTGCGGGACGCTATCGAAATTTTTATC

**************************************************

XAC1501|21240774_1733943-1735031 CACTATACATTGCTCACTGCCTGGTCTTTGCCAAACAGCGAACAGGTCAA

XCAW_02828|471265562_c3163934-3162846 CACTATACATTGCTCACTGCCTGGTCTTTGCCAAACAGCGAACAGGTCAA

**************************************************

XAC1501|21240774_1733943-1735031 ACCTGGATGCATGCATCCTTGACGGCAACAAGTCGAAATCTCAGTAATTC

XCAW_02828|471265562_c3163934-3162846 ACCTGGATGCATGCATCCTTGACGGCAACAAGTCGAAATCTCAGTAATTC

**************************************************

XAC1501|21240774_1733943-1735031 AGAAGCGTGGTTGGATCAAATCAAATTGATCTTGCAAAAAATTTCTGACT

XCAW_02828|471265562_c3163934-3162846 AGAAGCGTGGTTGGATCAAATCAAATTGATCTTGCAAAAAATTTCTGACT

**************************************************

XAC1501|21240774_1733943-1735031 TAGCAACAGCTGGTTTAGATCCGAGAGCGCTGTGGTCGCTTCTCTCATAC

XCAW_02828|471265562_c3163934-3162846 TAGCAACAGCTGGTTTAGATCCGAGAGCGCTGTGGTCGCTTCTCTCATAC

**************************************************

XAC1501|21240774_1733943-1735031 GGATATGTATCCGTAGAAACGCTTAGTGCTGGCAAAAATGCATGCAGTCC

XCAW_02828|471265562_c3163934-3162846 GGATATGTATCCGTAGAAACGCTTAGTGCTGGCAAAAATGCATGCAGTCC

**************************************************

XAC1501|21240774_1733943-1735031 AGCGCTTGCTGCAAAAATTATGGGGCGTCTACCGGCGCTTTTGGAACAGG

XCAW_02828|471265562_c3163934-3162846 AGCGCTTGCTGCAAAAATTATGGGGCGTCTACCGGCGCTTTTGGAACAGG

**************************************************

XAC1501|21240774_1733943-1735031 AACAGGGGATATTTTACTCAGCCGAGCTTGAAATCCTGATCATGGGAATT

XCAW_02828|471265562_c3163934-3162846 AACAGGGGATATTTTACTCAGCCGAGCTTGAAATCCTGATCATGGGAATT

**************************************************

XAC1501|21240774_1733943-1735031 GATCGCTCTATTAATAAGGATAGTTATGAAATTGAATGA

XCAW_02828|471265562_c3163934-3162846 GATCGCTCTATTAATAAGGATAGTTATGAAATTGAATGA

***************************************

PROTEIN ALIGNMENTS:

XAC1501|AAM36371.1|Hypothetical MSGHVARASSVRRNFMDGNFELAPLDLAFAPLPGRRDAPDASVWLDASFS

XCAW_02828|AGI08605.1|Hypothetical MSGHVARASSVRRNFMDGNFELAPLDLAFAPLPGRRDAPDASVWLDASFS

**************************************************

XAC1501|AAM36371.1|Hypothetical VYTPALLEPPLRTHEKIGDYQRFESDAQCVGYLSAISSRLIHVCRGSWKF

XCAW_02828|AGI08605.1|Hypothetical VYTPALLEPPLRTHEKIGDYQRFESDAQCVGYLSAMSSRLIHVCRGSWKF

***********************************:**************

XAC1501|AAM36371.1|Hypothetical ASIPDLRASRQAASAINASLRAGLPDTHGILNWSLNLAKNLGNSQVRSRT

XCAW_02828|AGI08605.1|Hypothetical ASIPDLRASRQTASAINASLRAGLPDTHGILNWSLNLAKNLGNSQVRSRT

***********:**************************************

XAC1501|AAM36371.1|Hypothetical DNAWVGGKKPSEAWLVLPQADQLPSLLKNLEDLLQGKSAQPVNCAFFEAI

XCAW_02828|AGI08605.1|Hypothetical DNAWVGGKKPSEAWLVLPQADQLPSLLKNLEDLLQGKSAQPVNCAFFEAI

**************************************************

XAC1501|AAM36371.1|Hypothetical AYQIICIHPLSDTNGRVTRTLLINLAGRYRNFYPLYIAHCLVFAKQRTGQ

XCAW_02828|AGI08605.1|Hypothetical AYQIICIHPLSDTNGRVTRTLLINLAGRYRNFYPLYIAHCLVFAKQRTGQ

**************************************************

XAC1501|AAM36371.1|Hypothetical TWMHASLTATSRNLSNSEAWLDQIKLILQKISDLATAGLDPRALWSLLSY

XCAW_02828|AGI08605.1|Hypothetical TWMHASLTATSRNLSNSEAWLDQIKLILQKISDLATAGLDPRALWSLLSY

**************************************************

XAC1501|AAM36371.1|Hypothetical GYVSVETLSAGKNACSPALAAKIMGRLPALLEQEQGIFYSAELEILIMGI

XCAW_02828|AGI08605.1|Hypothetical GYVSVETLSAGKNACSPALAAKIMGRLPALLEQEQGIFYSAELEILIMGI

**************************************************

XAC1501|AAM36371.1|Hypothetical DRSINKDSYEIE

XCAW_02828|AGI08605.1|Hypothetical DRSINKDSYEIE

************

**17 - NO KNOWN DOMAIN**

**no known domain (1)**

NUCLEOTIDE ALIGNMENTS:

XCC4177|21229478_4985010-4985237 GTGTTCCCCCACCGCAGTGCCATGTCCCCCATCAAGCAACACGCGCACCA

XAC4312|21240774_5098436-5098642 ATG---------------------TCCGCCATCAAGCAAGACGCCCATAC

XC_4265|66766352_5059208-5059435 GTGTTCCCCCACCGCAGTGCCATGTCCCCCATCAAGCAACACGCGCACCA

XCV4414|78045556_5090246-5090452 ATG---------------------TCCGCCATCAAGCAAGACGCCCACAT

PXO_03525|188574270_5205253-5205459 ATG---------------------TCCGCCATCAAGCAAGACGCCCATAC

XCR_4517|384425691_4850416-4850622 ATGT---------------------CCCCCATCAAGCAACACGCGCACCA

XOC_0203|384417079_c192991-192785 ATG---------------------TCCGCCATCAAGCAAGACGCCCATAC

XAC29_21725|470469929_5078117-5078323 ATG---------------------TCCGCCATCAAGCAAGACGCCCATAC

XCAW_04681|471265562_5244359-5244586 GTGCTCCCCCTCCGCCGTGCCATGTCCGCCATCAAGCAAGACGCCCATAC

** ** *********** **** **

XCC4177|21229478_4985010-4985237 GCTGATCGACGCGCTGCCCGAGGCCGCCGGGTGGGACGACCTTGCGCGCG

XAC4312|21240774_5098436-5098642 GCTGATCGACACGCTGCCCGAGACGGCTGGATGGGGCGAGGTGGTCCGCG

XC_4265|66766352_5059208-5059435 GCTGATCGACGCGCTGCCCGAGGCCGCCGGGTGGGACGACCTTGCGCGCG

XCV4414|78045556_5090246-5090452 GCTGATCGACACGCTGCCCGAGACGGCTGGATGGAGTGACGTGGTCCGCG

PXO_03525|188574270_5205253-5205459 GCTGATCGACACGCTGCCCGAGACCACCGGATGGGGCGAGGTGGTGCGCG

XCR_4517|384425691_4850416-4850622 GCTGATCGACGCGCTGCCCGAGGCCGCCGGGTGGGACGACCTTGCGCGCG

XOC_0203|384417079_c192991-192785 GCTGATCGACACGCTGCCCGAGACGGCCGGATGGGGCGAGGTGGTGCGCG

XAC29_21725|470469929_5078117-5078323 GCTGATCGACACGCTGCCCGAGACGGCTGGATGGGGCGAGGTGGTCCGCG

XCAW_04681|471265562_5244359-5244586 GCTGATCGACACGCTGCCCGAGACGGCTGGATGGGGCGAGGTGGTCCGCG

********** *********** * * ** *** ** * * ****

XCC4177|21229478_4985010-4985237 CGGTGGACGGCGCCCGCTTCGAGGCCGCGGTGCATGCAGGCATGGCCGCT

XAC4312|21240774_5098436-5098642 TCGTGGCCGACGCCAGCTTCCTGGCCGCGGTGCAGGAAGGGATTGCCGCT

XC_4265|66766352_5059208-5059435 CGGTGGACGGCGCCCGCTTCGAGGCCGCGGTGCATGCAGGCATGGCCGCT

XCV4414|78045556_5090246-5090452 TCGTGGCCGACGCCAGCTTCCAGGCCGCGGTGCAGGACGGCATTGCCGCT

PXO_03525|188574270_5205253-5205459 TCGTGGCCGATGCCAGCTTTCAGGCCGCCGTGCAGGACGGCATTGCCGCT

XCR_4517|384425691_4850416-4850622 CGGTGGACGGCGCCCGCTTCGAGGCCGCGGTGCATGCAGGCATGGCCGCT

XOC_0203|384417079_c192991-192785 TCGTGGCCGATGCCAGCTTTCAGGCCGCCGTGCAGGACGGCATTGCCGCT

XAC29_21725|470469929_5078117-5078323 TCGTGGCCGACGCCAGCTTCCTGGCCGCGGTGCAGGAAGGGATTGCCGCT

XCAW_04681|471265562_5244359-5244586 TCGTGGCCGACGCCAGCTTCCTGGCCGCGGTGCAGGAAGGGATTGCCGCT

**** ** *** **** ****** ***** * ** ** ******

XCC4177|21229478_4985010-4985237 GCCGACCAGGGCCAGATCGCCAGCCCGGCACAGGTGACGGCGATGTTCGC

XAC4312|21240774_5098436-5098642 GCAGATCAGGGCGCGTTGACGGCTCCGGCACAGGTGAGCGCACTGTTCGC

XC_4265|66766352_5059208-5059435 GCCGACCAGGGCCAGATCGCCAGCCCGGCACAGGTGACGGCGATGTTCGC

XCV4414|78045556_5090246-5090452 GCAGATCAGGGCGCGTTGACGACTCCGGCACAGGTGAGCGCACTGTTCGC

PXO_03525|188574270_5205253-5205459 GCAGATCAGGGCGCGTTGACGGCGCCGGCACAGGTGAGCGCACTGTTCGC

XCR_4517|384425691_4850416-4850622 GCCGACCAGGGCCAGATCGCCAGCCCGGCACAGGTGACGGCGATGTTCGC

XOC_0203|384417079_c192991-192785 GCAGATCAGGGCGCGTTGACGGCACCGGCACAGGTGAGCGCACTGTTCGC

XAC29_21725|470469929_5078117-5078323 GCAGATCAGGGCGCGTTGACGGCTCCGGCACAGGTGAGCGCACTGTTCGC

XCAW_04681|471265562_5244359-5244586 GCAGATCAGGGCGCGTTGACGGCTCCGGCACAGGTGAGCGCACTGTTCGC

** ** ****** * * * ************* ** *******

XCC4177|21229478_4985010-4985237 CAGATGGGGTGTTGATGTTGCGGCGTGA

XAC4312|21240774_5098436-5098642 CGGATGGGGCGTTGATGTTACGGCGTGA

XC_4265|66766352_5059208-5059435 CAGATGGGGTGTTGATGTTGCGGCGTGA

XCV4414|78045556_5090246-5090452 CAGATGGGGCGTTGATGTTACGGCGTGA

PXO_03525|188574270_5205253-5205459 CAGATGGGGCGTTGATGTTACGGCGTGA

XCR_4517|384425691_4850416-4850622 CAGATGGGGTGTTGATGTTGCGGCGTGA

XOC_0203|384417079_c192991-192785 CAGATGGGGCGTTGATGTTACGGCGTGA

XAC29_21725|470469929_5078117-5078323 CGGATGGGGCGTTGATGTTACGGCGTGA

XCAW_04681|471265562_5244359-5244586 CGGATGGGGCGTTGATGTTACGGCGTGA

* ******* ********* ********

PROTEIN ALIGNMENTS:

XCC4177|AAM43394.1|conserved MFPHRSAMSPIKQHAHQLIDALPEAAGWDDLARAVDGARFEAAVHAGMAA

XAC4312|AAM39142.1|conserved -------MSAIKQDAHTLIDTLPETAGWGEVVRVVADASFLAAVQEGIAA

XC_4265|AAY51303.1|conserved MFPHRSAMSPIKQHAHQLIDALPEAAGWDDLARAVDGARFEAAVHAGMAA

XCV4414|CAJ26145.1|conserved -------MSAIKQDAHMLIDTLPETAGWSDVVRVVADASFQAAVQDGIAA

PXO_03525|ACD61623.1|hypothetical -------MSAIKQDAHTLIDTLPETTGWGEVVRVVADASFQAAVQDGIAA

XCR_4517|AEL09368.1|conserved -------MSPIKQHAHQLIDALPEAAGWDDLARAVDGARFEAAVHAGMAA

XOC_0203|AEQ94450.1|hypothetical -------MSAIKQDAHTLIDTLPETAGWGEVVRVVADASFQAAVQDGIAA

XAC29_21725|AGH79726.1|hypothetical -------MSAIKQDAHTLIDTLPETAGWGEVVRVVADASFLAAVQEGIAA

XCAW_04681|AGI10439.1|hypothetical MLPLRRAMSAIKQDAHTLIDTLPETAGWGEVVRVVADASFLAAVQEGIAA

**.***.** ***:***::**.::.*.* .* * ***: *:**

XCC4177|AAM43394.1|conserved ADQGQIASPAQVTAMFARWGVDVAA

XAC4312|AAM39142.1|conserved ADQGALTAPAQVSALFAGWGVDVTA

XC_4265|AAY51303.1|conserved ADQGQIASPAQVTAMFARWGVDVAA

XCV4414|CAJ26145.1|conserved ADQGALTTPAQVSALFARWGVDVTA

PXO_03525|ACD61623.1|hypothetical ADQGALTAPAQVSALFARWGVDVTA

XCR_4517|AEL09368.1|conserved ADQGQIASPAQVTAMFARWGVDVAA

XOC_0203|AEQ94450.1|hypothetical ADQGALTAPAQVSALFARWGVDVTA

XAC29_21725|AGH79726.1|hypothetical ADQGALTAPAQVSALFAGWGVDVTA

XCAW_04681|AGI10439.1|hypothetical ADQGALTAPAQVSALFAGWGVDVTA

**** :::****:*:** *****:*

**no known domain (2)**

NUCLEOTIDE ALIGNMENTS:

XCC1065|21229478_1226846-1227109 ATGAA-----------A---------------------------------

XAC1168|21240774_1330756-1331019 ATGAAA--------------------------------------------

XC_3186|66766352_c3818920-3818657 ATGAAA--------------------------------------------

XCV1188|78045556_1328156-1328419 ATGAA-----------A---------------------------------

PXO_02068|188574270_c3836337-3836074 ATGAA-----------A---------------------------------

XCR_1271|384425691_1292372-1292635 ATGAA-----------A---------------------------------

XOC_1219|384417079_1189257-1189943 GTGGTGGCCTTGAGCGATCGCCTGTCCATTGGCTTGGTGACAGCGACAGC

XALq_3233|386081561_30308-30769 ATGCAG---------AG---------------------------------

XAC29_05870|470469929_1334339-1334602 ATGAAA--------------------------------------------

XCAW_01269|471265562_1417957-1418220 ATGAAA--------------------------------------------

**

XCC1065|21229478_1226846-1227109 --------------------------------------------------

XAC1168|21240774_1330756-1331019 --------------------------------------------------

XC_3186|66766352_c3818920-3818657 --------------------------------------------------

XCV1188|78045556_1328156-1328419 --------------------------------------------------

PXO_02068|188574270_c3836337-3836074 --------------------------------------------------

XCR_1271|384425691_1292372-1292635 --------------------------------------------------

XOC_1219|384417079_1189257-1189943 ATTCGTGGCGACCAAGCGCGAAGCCTTCGTTACCCGAGGCAACAGCGACT

XALq_3233|386081561_30308-30769 --------------------------------------------------

XAC29_05870|470469929_1334339-1334602 --------------------------------------------------

XCAW_01269|471265562_1417957-1418220 --------------------------------------------------

XCC1065|21229478_1226846-1227109 ---------------------------TC------------------C--

XAC1168|21240774_1330756-1331019 ---------------------------TCC--------------------

XC_3186|66766352_c3818920-3818657 ---------------------------TC------------------C--

XCV1188|78045556_1328156-1328419 ---------------------------TC------------------C--

PXO_02068|188574270_c3836337-3836074 ---------------------------TC------------------T--

XCR_1271|384425691_1292372-1292635 ---------------------------TC------------------C--

XOC_1219|384417079_1189257-1189943 TTCTTTCCAGTCACGACCTGGAGGACGTCCTCAATGTCGTCGATGGCCGT

XALq_3233|386081561_30308-30769 -----TA--------------------TG------------------A--

XAC29_05870|470469929_1334339-1334602 ---------------------------TCC--------------------

XCAW_01269|471265562_1417957-1418220 ---------------------------TCC--------------------

*

XCC1065|21229478_1226846-1227109 -----------------------------G--------------------

XAC1168|21240774_1330756-1331019 --------------------------------------------------

XC_3186|66766352_c3818920-3818657 -----------------------------G--------------------

XCV1188|78045556_1328156-1328419 -----------------------------G--------------------

PXO_02068|188574270_c3836337-3836074 -----------------------------G--------------------

XCR_1271|384425691_1292372-1292635 -----------------------------G--------------------

XOC_1219|384417079_1189257-1189943 ACCGAGGTGGTGGAAGAGTTTGCCGTGGAGCATGCAGACCTTCGGCAGTG

XALq_3233|386081561_30308-30769 -----------------------------C--------------------

XAC29_05870|470469929_1334339-1334602 --------------------------------------------------

XCAW_01269|471265562_1417957-1418220 --------------------------------------------------

XCC1065|21229478_1226846-1227109 --------------------------------------------------

XAC1168|21240774_1330756-1331019 --------------------------------------------------

XC_3186|66766352_c3818920-3818657 --------------------------------------------------

XCV1188|78045556_1328156-1328419 --------------------------------------------------

PXO_02068|188574270_c3836337-3836074 --------------------------------------------------

XCR_1271|384425691_1292372-1292635 --------------------------------------------------

XOC_1219|384417079_1189257-1189943 GGTTGCCGATGTATTTGCGGAGCTAGTGGATACCCCGGCGTTTGTGAACG

XALq_3233|386081561_30308-30769 --------------------------------------------------

XAC29_05870|470469929_1334339-1334602 --------------------------------------------------

XCAW_01269|471265562_1417957-1418220 --------------------------------------------------

XCC1065|21229478_1226846-1227109 --------------------------------------------------

XAC1168|21240774_1330756-1331019 --------------------------------------------------

XC_3186|66766352_c3818920-3818657 --------------------------------------------------

XCV1188|78045556_1328156-1328419 --------------------------------------------------

PXO_02068|188574270_c3836337-3836074 --------------------------------------------------

XCR_1271|384425691_1292372-1292635 --------------------------------------------------

XOC_1219|384417079_1189257-1189943 CGTTGCCGGGAATGGTTGCCGAACCGGCGCGCGCAAGCATCGTGCTTCAG

XALq_3233|386081561_30308-30769 --------------------------------------------------

XAC29_05870|470469929_1334339-1334602 --------------------------------------------------

XCAW_01269|471265562_1417957-1418220 --------------------------------------------------

XCC1065|21229478_1226846-1227109 --------------------------------------------------

XAC1168|21240774_1330756-1331019 --------------------------------------------------

XC_3186|66766352_c3818920-3818657 --------------------------------------------------

XCV1188|78045556_1328156-1328419 --------------------------------------------------

PXO_02068|188574270_c3836337-3836074 --------------------------------------------------

XCR_1271|384425691_1292372-1292635 --------------------------------------------------

XOC_1219|384417079_1189257-1189943 CGTCTACGCGCCATCGCATCAGCATGGAAGCGAGGTTGTCTGCTTAGGTC

XALq_3233|386081561_30308-30769 --------------------------------------------------

XAC29_05870|470469929_1334339-1334602 --------------------------------------------------

XCAW_01269|471265562_1417957-1418220 --------------------------------------------------

XCC1065|21229478_1226846-1227109 --------------------------------------------------

XAC1168|21240774_1330756-1331019 --------------------------------------------------

XC_3186|66766352_c3818920-3818657 --------------------------------------------------

XCV1188|78045556_1328156-1328419 --------------------------------------------------

PXO_02068|188574270_c3836337-3836074 --------------------------------------------------

XCR_1271|384425691_1292372-1292635 --------------------------------------------------

XOC_1219|384417079_1189257-1189943 TGGCAGAGAAAACCTTGAAGCACCGGCGCTCGCTTGCGATGATGCATTGC

XALq_3233|386081561_30308-30769 --------------------------------------------------

XAC29_05870|470469929_1334339-1334602 --------------------------------------------------

XCAW_01269|471265562_1417957-1418220 --------------------------------------------------

XCC1065|21229478_1226846-1227109 ---------------------------------CATC-----ACTT-CCA

XAC1168|21240774_1330756-1331019 --------------------------------GCATC-----TCTT-CCA

XC_3186|66766352_c3818920-3818657 ---------------------------------CATC-----TCTT-CCA

XCV1188|78045556_1328156-1328419 ---------------------------------CATC-----TCTT-CCA

PXO_02068|188574270_c3836337-3836074 ---------------------------------CATC-----TCTT-CCA

XCR_1271|384425691_1292372-1292635 ---------------------------------CATC-----TCTT-CCA

XOC_1219|384417079_1189257-1189943 AATTGCAACGCACTGGGCCTGCCATGAAATCTGCATC-----TCTT-CCA

XALq_3233|386081561_30308-30769 ---------------------------------CATCGAACAGCTGCGCA

XAC29_05870|470469929_1334339-1334602 --------------------------------GCATC-----TCTT-CCA

XCAW_01269|471265562_1417957-1418220 --------------------------------GCATC-----TCTT-CCA

**** ** **

XCC1065|21229478_1226846-1227109 TCGC-------TCCGG---------------------G-TAGACCC-GGC

XAC1168|21240774_1330756-1331019 TCGC-------TCCGG---------------------G-TAGACCC-GGC

XC_3186|66766352_c3818920-3818657 TCGC-------TCCGT---------------------G-TAGACCC-GGC

XCV1188|78045556_1328156-1328419 TCGC-------TCCGT---------------------G-TGGACCC-GGC

PXO_02068|188574270_c3836337-3836074 TCGC-------TCCGG---------------------G-TAGACCC-GGC

XCR_1271|384425691_1292372-1292635 TCGC-------TCCGG---------------------G-TAGACCC-GGC

XOC_1219|384417079_1189257-1189943 TCGC-------TCCGG---------------------G-TAGACCC-GGC

XALq_3233|386081561_30308-30769 TCGCCAATAGAGCCGGAGGCGTCGCCGGCGTCACCTTGAAAGGCCAGGGC

XAC29_05870|470469929_1334339-1334602 TCGC-------TCCGG---------------------G-TAGACCC-GGC

XCAW_01269|471265562_1417957-1418220 TCGC-------TCCGG---------------------G-TAGACCC-GGC

**** *** * * ** ***

XCC1065|21229478_1226846-1227109 GCTG----------------CGCGAA--GCG--GCCGAAGCCGTGTTGC-

XAC1168|21240774_1330756-1331019 GCTG----------------CGCGAA--GCG--GCCGAAGCCGTGTTGC-

XC_3186|66766352_c3818920-3818657 GTTG----------------CGCGAA--GCG--GCCGAAGCCGTGTTGC-

XCV1188|78045556_1328156-1328419 GCTG----------------CGCGAA--GCG--GCCGAAGCCGTGTTGC-

PXO_02068|188574270_c3836337-3836074 GCTG----------------CGCGAA--GCG--GCCGAGGCCGTGTTGC-

XCR_1271|384425691_1292372-1292635 GCTG----------------CGCCAA--GCG--GCCGAAGCCGTGTTGC-

XOC_1219|384417079_1189257-1189943 GCTG----------------CGCGAA--GCG--GCCGAGGCCGTGTTGC-

XALq_3233|386081561_30308-30769 GGTGTTTTTCTCGTCCAGATCACGACACGCAACGGCGATGCGGTGCTGGC

XAC29_05870|470469929_1334339-1334602 GCTG----------------CGCGAA--GCG--GCCGAAGCCGTGTTGC-

XCAW_01269|471265562_1417957-1418220 GCTG----------------CGCGAA--GCG--GCCGAAGCCGTGTTGC-

* ** * * * ** * *** ** *** **

XCC1065|21229478_1226846-1227109 -------------AGGAGGGTGAGACGCTT------TCCAGCTTTGTCGA

XAC1168|21240774_1330756-1331019 -------------AGGAGGGCGAGACGCTG------TCCAGCTTTGTCGA

XC_3186|66766352_c3818920-3818657 -------------AGGAGGGTGAGACGCTG------TCCAGCTTTGTCGA

XCV1188|78045556_1328156-1328419 -------------AGGAGGGCGAGACGCTT------TCCAGCTTCGTCGA

PXO_02068|188574270_c3836337-3836074 -------------AGGAGGGTGAGACGCTG------TCCAGCTTTGTCGA

XCR_1271|384425691_1292372-1292635 -------------AGGAGGGTGAGACGCTC------TCCAGCTTTGTCGA

XOC_1219|384417079_1189257-1189943 -------------AGGAGGGTGAGACGCTG------TCCAGCTTTGTCGA

XALq_3233|386081561_30308-30769 CAAGGCCCGCGGCACGGAGCCGCGCCGCTTTGGCAATCCGGCGTCGGCCA

XAC29_05870|470469929_1334339-1334602 -------------AGGAGGGCGAGACGCTG------TCCAGCTTTGTCGA

XCAW_01269|471265562_1417957-1418220 -------------AGGAGGGCGAGACGCTG------TCCAGCTTTGTCGA

* * * * * **** *** ** * * * *

XCC1065|21229478_1226846-1227109 GCACT---CCGTGC-------G-----TGCCCAGGTGCAG----------

XAC1168|21240774_1330756-1331019 GCACT---CCGTCC-------G-----CGCCCAGGTGCAG----------

XC_3186|66766352_c3818920-3818657 GCACT---CCGTAC-------G-----CGCCCAGGTGCAG----------

XCV1188|78045556_1328156-1328419 GCACT---CCGTGC-------G-----CGCCCAGGTGCAG----------

PXO_02068|188574270_c3836337-3836074 GCACT---CCGTGC-------G-----CGCCCAGGTGCAG----------

XCR_1271|384425691_1292372-1292635 GCACT---CCGTGC-------G-----CGCCCAGGTGCAG----------

XOC_1219|384417079_1189257-1189943 GCACT---CCGTGC-------G-----CGCCCAGGTGCAG----------

XALq_3233|386081561_30308-30769 TCAATGTGCTGTGCGACGTGGGCATCACGATCGGGTCCTTCGATGCCAGC

XAC29_05870|470469929_1334339-1334602 GCACT---CCGTCC-------G-----CGCCCAGGTGCAG----------

XCAW_01269|471265562_1417957-1418220 GCACT---CCGTCC-------G-----CGCCCAGGTGCAG----------

** * * ** * * * * *** *

XCC1065|21229478_1226846-1227109 ------CAGCGCCAGCAGCAGGAAG---CC---TTC-ATTGCCCGTGGGC

XAC1168|21240774_1330756-1331019 ------CAGCGCCAGCAGCAGGAAG---CC---TTC-ATCGCACGCGGGC

XC_3186|66766352_c3818920-3818657 ------CAGCGTCAGCAGCAGGAAG---CC---TTC-ATTGCGCGCGGGC

XCV1188|78045556_1328156-1328419 ------CAGCGCCAGCAGCAGGAAG---CC---TTC-ATCGCACGCGGGC

PXO_02068|188574270_c3836337-3836074 ------CAGCGGCAGCAGCAGGAAG---CC---TTC-ATCGCGCGCGGGC

XCR_1271|384425691_1292372-1292635 ------CAGCGCCAGCGGCAGGACG---CC---TTC-ATCGCACGCGGGC

XOC_1219|384417079_1189257-1189943 ------CAGCGGCAGCAGCAGGAAG---CC---TTC-ATCGCGCGCGGGC

XALq_3233|386081561_30308-30769 GAATGGCATCCCGAGGAGAAGGAAGAAACCGCCGGCAATCGCGGTCGTGC

XAC29_05870|470469929_1334339-1334602 ------CAGCGCCAGCAGCAGGAAG---CC---TTC-ATCGCACGCGGGC

XCAW_01269|471265562_1417957-1418220 ------CAGCGCCAGCAGCAGGAAG---CC---TTC-ATCGCACGCGGGC

** * ** * **** * ** * ** ** * **

XCC1065|21229478_1226846-1227109 TGGCTT-------------CGCGTGA------------C----A-GC--G

XAC1168|21240774_1330756-1331019 TGGCCT-------------CGCGTGA------------C----A-GC--G

XC_3186|66766352_c3818920-3818657 TGGCCT-------------CGCGTGA------------C----A-GC--G

XCV1188|78045556_1328156-1328419 TGGCCT-------------CGCGTGA------------C----A-GC--G

PXO_02068|188574270_c3836337-3836074 TGGCCT-------------CGCGTGA------------C----A-GC--G

XCR_1271|384425691_1292372-1292635 TGGCCT-------------CGCGTGA------------C----A-GC--G

XOC_1219|384417079_1189257-1189943 TGGCCT-------------CGCGTGA------------C----A-GC--G

XALq_3233|386081561_30308-30769 CGAGCACATGCGCAAGGCCCACCAGGCCGCGGCATATACGGATTGGCTGG

XAC29_05870|470469929_1334339-1334602 TGGCCT-------------CGCGTGA------------C----A-GC--G

XCAW_01269|471265562_1417957-1418220 TGGCCT-------------CGCGTGA------------C----A-GC--G

* * * * * ** *

XCC1065|21229478_1226846-1227109 CCAAGGCATC-CAACCGGTACATCGAG-----------GC-------G-A

XAC1168|21240774_1330756-1331019 CCAAGGCGGC-AGGCCACTACATCGAC-----------GT-------G-A

XC_3186|66766352_c3818920-3818657 CCAAGGCGTC-CAACCGGTACACCGAC-----------GC-------G-A

XCV1188|78045556_1328156-1328419 CCAAGGCATC-CAACCAGTACATCGAC-----------GC-------G-A

PXO_02068|188574270_c3836337-3836074 CCAAGGCATC-CAACCGATACATCGAC-----------GC-------G-A

XCR_1271|384425691_1292372-1292635 CCAAGGCATC-CAACCAGTACATCGAC-----------GC-------G-A

XOC_1219|384417079_1189257-1189943 CCAAGGCGGC-GGGCCACTACATCGAC-----------GC-------G-A

XALq_3233|386081561_30308-30769 CCAAGGAGACACAAGCGGC-GATCGACGATCCGCGGCCGCGTGTGGCGCA

XAC29_05870|470469929_1334339-1334602 CCAAGGCGGC-AGGCCACTACATCGAC-----------GT-------G-A

XCAW_01269|471265562_1417957-1418220 CCAAGGCGGC-AGGCCACTACATCGAC-----------GT-------G-A

****** * * * *** * * *

XCC1065|21229478_1226846-1227109 A--GGATGTGCTGGC-----CGGGCTGCAG----TCCCAACTGGACAAG-

XAC1168|21240774_1330756-1331019 A--GGATGTGCTGGC-----CGGGCTGCAG----TCCCAGCTGGACGAG-

XC_3186|66766352_c3818920-3818657 A--GGATGTGCTTGC-----CGGGCTGCAG----TCCCAGCTGGGCCAG-

XCV1188|78045556_1328156-1328419 A--GGATGTGCTGGC-----CGGGCTGCAG----TCCAAACTGGACAAG-

PXO_02068|188574270_c3836337-3836074 C--CGATGTGCTGGC-----CGGGCTGCAG----TCCCAACTGCACAAG-

XCR_1271|384425691_1292372-1292635 A--GGATGTGCTGGC-----CGGGCTGCAG----TCCCAACTGGACCAG-

XOC_1219|384417079_1189257-1189943 C--CGATGTGCTGGC-----CGGACTGCAG----TCCCAACTGCACAAG-

XALq_3233|386081561_30308-30769 AGCGGGCGTGCTCGAGCGTCTGGATCGCAAGATGGCCACGCTCGGCAAGC

XAC29_05870|470469929_1334339-1334602 A--GGATGTGCTGGC-----CGGGCTGCAG----TCCCAGCTGGACGAG-

XCAW_01269|471265562_1417957-1418220 A--GGATGTGCTGGC-----CGGGCTGCAG----TCCCAGCTGGACGAG-

* ***** * ** *** ** ** * **

XCC1065|21229478_1226846-1227109 -----------GCGCGCAAGG---G---CTGA

XAC1168|21240774_1330756-1331019 -----------GCGCGCAAGA---G---CTGA

XC_3186|66766352_c3818920-3818657 -----------GCGCGCAAGG---G---CTAG

XCV1188|78045556_1328156-1328419 -----------GCGCGCAAGG---G---CTAA

PXO_02068|188574270_c3836337-3836074 -----------GCGCGCAAGG---G---CTGA

XCR_1271|384425691_1292372-1292635 -----------GCGCGCAAGG---G---CTAA

XOC_1219|384417079_1189257-1189943 -----------GCGCGCAAGG---G---CTGA

XALq_3233|386081561_30308-30769 CCAGCGTGGCTGCGCGCAAGAAGCGCGCGTGA

XAC29_05870|470469929_1334339-1334602 -----------GCGCGCAAGA---G---CTGA

XCAW_01269|471265562_1417957-1418220 -----------GCGCGCAAGA---G---CTGA

********* * *

PROTEIN ALIGNMENTS:

XCC1065|AAM40364.1|conserved --------------------------------------------------

XAC1168|AAM36040.1|conserved --------------------------------------------------

XC_3186|AAY50230.1|conserved --------------------------------------------------

XCV1188|CAJ22819.1|conserved --------------------------------------------------

PXO_02068|ACD60309.1|hypothetical --------------------------------------------------

XCR_1271|AEL06171.1|conserved --------------------------------------------------

XOC_1219|AEQ95407.1| MVALSDRLSIGLVTATAFVATKREAFVTRGNSDFLSSHDLEDVLNVVDGR

XALq_3233|WP_014607103.1| --------------------------------------------------

XAC29_05870|AGH76681.1|hypothetical --------------------------------------------------

XCAW_01269|AGI07071.1|hypothetical --------------------------------------------------

XCC1065|AAM40364.1|conserved --------------------------------------------------

XAC1168|AAM36040.1|conserved --------------------------------------------------

XC_3186|AAY50230.1|conserved --------------------------------------------------

XCV1188|CAJ22819.1|conserved --------------------------------------------------

PXO_02068|ACD60309.1|hypothetical --------------------------------------------------

XCR_1271|AEL06171.1|conserved --------------------------------------------------

XOC_1219|AEQ95407.1| TEVVEEFAVEHADLRQWVADVFAELVDTPAFVNALPGMVAEPARASIVLQ

XALq_3233|WP_014607103.1| --------------------------------------------------

XAC29_05870|AGH76681.1|hypothetical --------------------------------------------------

XCAW_01269|AGI07071.1|hypothetical --------------------------------------------------

XCC1065|AAM40364.1|conserved -----------------------------------------MKSASLPSL

XAC1168|AAM36040.1|conserved -----------------------------------------MKSASLPSL

XC_3186|AAY50230.1|conserved -----------------------------------------MKSASLPSL

XCV1188|CAJ22819.1|conserved -----------------------------------------MKSASLPSL

PXO_02068|ACD60309.1|hypothetical -----------------------------------------MKSASLPSL

XCR_1271|AEL06171.1|conserved -----------------------------------------MKSASLPSL

XOC_1219|AEQ95407.1| RLRAIASAWKRGCLLRSGRENLEAPALACDDALQLQRTGPAMKSASLPSL

XALq_3233|WP_014607103.1| -----------------------------------------MQSMTIEQL

XAC29_05870|AGH76681.1|hypothetical -----------------------------------------MKSASLPSL

XCAW_01269|AGI07071.1|hypothetical -----------------------------------------MKSASLPSL

*:* :: .*

XCC1065|AAM40364.1|conserved RVDPALREAAEA--------------------------------------

XAC1168|AAM36040.1|conserved RVDPALREAAEA--------------------------------------

XC_3186|AAY50230.1|conserved RVDPALREAAEA--------------------------------------

XCV1188|CAJ22819.1|conserved RVDPALREAAEA--------------------------------------

PXO_02068|ACD60309.1|hypothetical RVDPALREAAEA--------------------------------------

XCR_1271|AEL06171.1|conserved RVDPALRQAAEA--------------------------------------

XOC_1219|AEQ95407.1| RVDPALREAAEA--------------------------------------

XALq_3233|WP_014607103.1| RIANRAGGVAGVTLKGQGGVFLVQITTRNGDAVLAKARGTEPRRFGNPAS

XAC29_05870|AGH76681.1|hypothetical RVDPALREAAEA--------------------------------------

XCAW_01269|AGI07071.1|hypothetical RVDPALREAAEA--------------------------------------

*: .* .

XCC1065|AAM40364.1|conserved ----VLQEGETLSSFVEHS-------------VRAQVQQRQQQEAFIARG

XAC1168|AAM36040.1|conserved ----VLQEGETLSSFVEHS-------------VRAQVQQRQQQEAFIARG

XC_3186|AAY50230.1|conserved ----VLQEGETLSSFVEHS-------------VRAQVQQRQQQEAFIARG

XCV1188|CAJ22819.1|conserved ----VLQEGETLSSFVEHS-------------VRAQVQQRQQQEAFIARG

PXO_02068|ACD60309.1|hypothetical ----VLQEGETLSSFVEHS-------------VRAQVQQRQQQEAFIARG

XCR_1271|AEL06171.1|conserved ----VLQEGETLSSFVEHS-------------VRAQVQQRQRQDAFIARG

XOC_1219|AEQ95407.1| ----VLQEGETLSSFVEHS-------------VRAQVQQRQQQEAFIARG

XALq_3233|WP_014607103.1| AINVLCDVGITIGSFDASEWHPEEKEETAGNRGRAEHMRKAHQAAAYTDW

XAC29_05870|AGH76681.1|hypothetical ----VLQEGETLSSFVEHS-------------VRAQVQQRQQQEAFIARG

XCAW_01269|AGI07071.1|hypothetical ----VLQEGETLSSFVEHS-------------VRAQVQQRQQQEAFIARG

: : * *:.** . **: :: :* * :

XCC1065|AAM40364.1|conserved LASRDSAKASNRYIEAKDVLAGLQSQLDK-----------ARK--G

XAC1168|AAM36040.1|conserved LASRDSAKAAGHYIDVKDVLAGLQSQLDE-----------ARK--S

XC_3186|AAY50230.1|conserved LASRDSAKASNRYTDAKDVLAGLQSQLGQ-----------ARK--G

XCV1188|CAJ22819.1|conserved LASRDSAKASNQYIDAKDVLAGLQSKLDK-----------ARK--G

PXO_02068|ACD60309.1|hypothetical LASRDSAKASNRYIDATDVLAGLQSQLHK-----------ARK--G

XCR_1271|AEL06171.1|conserved LASRDSAKASNQYIDAKDVLAGLQSQLDQ-----------ARK--G

XOC_1219|AEQ95407.1| LASRDSAKAAGHYIDATDVLAGLQSQLHK-----------ARK--G

XALq_3233|WP_014607103.1| LAKETQAAIDD--PRPRVAQAGVLERLDRKMATLGKPSVAARKKRA

XAC29_05870|AGH76681.1|hypothetical LASRDSAKAAGHYIDVKDVLAGLQSQLDE-----------ARK--S

XCAW_01269|AGI07071.1|hypothetical LASRDSAKAAGHYIDVKDVLAGLQSQLDE-----------ARK--S

**.. .* . . **: .:* . *** .

**18 - DOMAIN cl00877 - superf. MazE / PRK11347 - ChpS**

**cl00877 - superf. MazE / PRK11347 - ChpS (1)**

NUCLEOTIDE ALIGNMENTS:

XACa0028|58033143_c18814-18557 ATGCATACGACCAATCTGCGTAAGGTCGGTGGCTCGATCATGTTGGCTGT

XAC29_22934|470474282_c30692-30435 ATGCATACGACCAATCTGCGTAAGGTCGGTGGCTCGATCATGTTGGCTGT

XCAW_b00013|471265493_10384-10641 ATGCATACGACCAATCTGCGTAAGGTCGGTGGCTCGATCATGTTGGCTGT

**************************************************

XACa0028|58033143_c18814-18557 CCCGCCCGCCATTCTGGATATGCTGCGTCTGCGTGCCGGCGCTACCGTTG

XAC29_22934|470474282_c30692-30435 CCCGCCCGCCATTCTGGATATGCTGCGTCTGCGTGCCGGCGCTACCGTTG

XCAW_b00013|471265493_10384-10641 CCCGCCCGCCATTCTGGATATGCTGCGTCTGCGTGCCGGCGCTACCGTTG

**************************************************

XACa0028|58033143_c18814-18557 GCTTGGCCGTCGATCATGGTCGCCTGGTGATCGAACCCACGCTGCGTCCG

XAC29_22934|470474282_c30692-30435 GCTTGGCCGTCGATCATGGTCGCCTGGTGATCGAACCCACGCTGCGTCCG

XCAW_b00013|471265493_10384-10641 GCTTGGCCGTCGATCATGGTCGCCTGGTGATCGAACCCACGCTGCGTCCG

**************************************************

XACa0028|58033143_c18814-18557 TACTACAGCCTGGATGAGCTGCTGGCCCAATGCGACGCATCCGCCGAACT

XAC29_22934|470474282_c30692-30435 TACTACAGCCTGGATGAGCTGCTGGCCCAATGCGACGCATCCGCCGAACT

XCAW_b00013|471265493_10384-10641 TACTACAGCCTGGATGAGCTGCTGGCCCAATGCGACGCATCCGCCGAACT

**************************************************

XACa0028|58033143_c18814-18557 CTCCACCGAAGATCGGGAATGGCTCGACGCCAAGCCTGTTGGCAGCGAGC

XAC29_22934|470474282_c30692-30435 CTCCACCGAAGATCGGGAATGGCTCGACGCCAAGCCTGTTGGCAGCGAGC

XCAW_b00013|471265493_10384-10641 CTCCACCGAAGATCGGGAATGGCTCGACGCCAAGCCTGTTGGCAGCGAGC

**************************************************

XACa0028|58033143_c18814-18557 TGCTGTGA

XAC29_22934|470474282_c30692-30435 TGCTGTGA

XCAW_b00013|471265493_10384-10641 TGCTGTGA

********

Protein alignments:

XACa0028|AAM39232.1|plasmid MHTTNLRKVGGSIMLAVPPAILDMLRLRAGATVGLAVDHGRLVIEPTLRP

XAC29_22934|AGH79940.1|plasmid MHTTNLRKVGGSIMLAVPPAILDMLRLRAGATVGLAVDHGRLVIEPTLRP

XCAW_b00013|AGI10533.1|plasmid MHTTNLRKVGGSIMLAVPPAILDMLRLRAGATVGLAVDHGRLVIEPTLRP

**************************************************

XACa0028|AAM39232.1|plasmid YYSLDELLAQCDASAELSTEDREWLDAKPVGSELL

XAC29_22934|AGH79940.1|plasmid YYSLDELLAQCDASAELSTEDREWLDAKPVGSELL

XCAW_b00013|AGI10533.1|plasmid YYSLDELLAQCDASAELSTEDREWLDAKPVGSELL

***********************************

**cl00877 - superf. MazE / COG4456 - VagC (2)**

NUCLEOTIDE ALIGNMENTS:

XACb0060|21264228_c52285-52025 GTGAG---------CC-----AAG--------TTG-----CCA-------

XCVd0101|78045309_c111830-111429 ATGAA---------GCGGTACATGCT---TG-ATA-----CCAAC-ACCG

XAC29_22434|470464772_c51310-51050 GTGAG---------CC-----AAG--------TTG-----CCA-------

XCAW_b00030|471265493_c28889-28173 ATGTTTGGTATATATC-----AAGGCGTATGCCTGTCCGACAAACGATCG

** * * * * * *

XACb0060|21264228_c52285-52025 ------------------AGCTGT-TC-AT------------CAATGG--

XCVd0101|78045309_c111830-111429 TG----------------AGCCATCTC-GT------------CAAGAG--

XAC29_22434|470464772_c51310-51050 ------------------AGCTGT-TC-AT------------CAATGG--

XCAW_b00030|471265493_c28889-28173 TTTGTCGGTCGTGCCGGACGCTGT-CCTGAAAACTGCTTGTGCAACGGGT

** * * *** *

XACb0060|21264228_c52285-52025 --CCGC-A------G---CCAGGCA-------------------------

XCVd0101|78045309_c111830-111429 --CCATCC-GGC-TGTCTCTCGACGG--------G---TCATTGAA----

XAC29_22434|470464772_c51310-51050 --CCGC-A------G---CCAGGCA-------------------------

XCAW_b00030|471265493_c28889-28173 TGTCGG-ACACCAAG---ATAGACGGACGGAATGGCGGACA----AGATG

* * * *

XACb0060|21264228_c52285-52025 -GTCCG-CTTGC----------CT--------------GC--------CG

XCVd0101|78045309_c111830-111429 -GTGCC-TATGA----------CT--------------GCG-C-----TA

XAC29_22434|470464772_c51310-51050 -GTCCG-CTTGC----------CT--------------GC--------CG

XCAW_b00030|471265493_c28889-28173 GGTGGCAAATGGCACTGATCGGCTATGCGCGGGTATCAACGGCGGAACAG

** ** ** *

XACb0060|21264228_c52285-52025 CCTAT---CGC-TTTACCGA-------------GAAA---------GAG-

XCVd0101|78045309_c111830-111429 TGTATGTCCGCAATTACCGG-------------TGGG---------GAGC

XAC29_22434|470464772_c51310-51050 CCTAT---CGC-TTTACCGA-------------GAAA---------GAG-

XCAW_b00030|471265493_c28889-28173 GACAC---CGC-TTTGCAGACGGATGCGCTGCGCAAAGCAGGCTGCGAGC

* *** ** * * ***

XACb0060|21264228_c52285-52025 --GTTTTC--A----------TT----CGCCAAGAC--------CCGGA-

XCVd0101|78045309_c111830-111429 TGATGTTCGGC----------TT----GGCTAAGGT-------GCCAGA-

XAC29_22434|470464772_c51310-51050 --GTTTTC--A----------TT----CGCCAAGAC--------CCGGA-

XCAW_b00030|471265493_c28889-28173 GCGTTTTC--GAGGACACGGCTTCCGGGGCCAAGGCTGACCGCCCCGGTT

* *** ** ** *** ** *

XACb0060|21264228_c52285-52025 -------------------------GACCG-GTGACGT-----CATC---

XCVd0101|78045309_c111830-111429 -------------------------CGCCAAACGCCTTCAG--CAAG---

XAC29_22434|470464772_c51310-51050 -------------------------GACCG-GTGACGT-----CATC---

XCAW_b00030|471265493_c28889-28173 TGGCTGATGCGCTGGCCTACCTGCGCAACG-GCGACGTGCTGGCAGTCTG

* * * * **

XACb0060|21264228_c52285-52025 ---------CTGTCTCGGAA--GC----------CTG----CAACG--T-

XCVd0101|78045309_c111830-111429 ---------CCGTCATGGAAC-TC----------CTG----CGGCGCGT-

XAC29_22434|470464772_c51310-51050 ---------CTGTCTCGGAA--GC----------CTG----CAACG--T-

XCAW_b00030|471265493_c28889-28173 GCGGCTGGACCGGCTTGGGCGCTCCATGCCGCACCTGATCGAAACG--AT

* * * ** * *** **

XACb0060|21264228_c52285-52025 ---------GGGAGG-----------AC-TTCTTT---AT----------

XCVd0101|78045309_c111830-111429 ---------GGATGT-----------GC-TGCCTTGGGATGGCGCGGTAA

XAC29_22434|470464772_c51310-51050 ---------GGGAGG-----------AC-TTCTTT---AT----------

XCAW_b00030|471265493_c28889-28173 AGGCGCGCTGGAAGCGCGAGGCGTCGGCTTTCGTT---------------

** * * * * **

XACb0060|21264228_c52285-52025 -----CG--T-AC------TCAA--------------------GAGCG--

XCVd0101|78045309_c111830-111429 TGGAGCG--TTACGGAAGCGTAA--------------------GAGCGGA

XAC29_22434|470464772_c51310-51050 -----CG--T-AC------TCAA--------------------GAGCG--

XCAW_b00030|471265493_c28889-28173 -----CGCTG-AC------GGAAGCCATCGACACCACCACGCCGGGCG--

** ** ** * ***

XACb0060|21264228_c52285-52025 --CA---GAT---------GT------------GCCTGCT----------

XCVd0101|78045309_c111830-111429 CCTA---GAA---------AA------------GCAAGGT----------

XAC29_22434|470464772_c51310-51050 --CA---GAT---------GT------------GCCTGCT----------

XCAW_b00030|471265493_c28889-28173 GGCGGCTCATCTTCCACGTGTTCGGCGCGCTGGGCCAGTTCGAGCGCGAT

* ** * *

XACb0060|21264228_c52285-52025 --AAT---------TT--TCT---------AG----------ATGCC-GC

XCVd0101|78045309_c111830-111429 --AAAGCGCTTGGGTCGCTCG---------AC----------ATGCTTAT

XAC29_22434|470464772_c51310-51050 --AAT---------TT--TCT---------AG----------ATGCC-GC

XCAW_b00030|471265493_c28889-28173 CTGAT---------TC--GCGAGCGCACCAAGGCCGGGTTGAGTGCC-GC

* * * * ***

XACb0060|21264228_c52285-52025 ---------CGAGCG-----------------CAA-TCAG----------

XCVd0101|78045309_c111830-111429 C--------GCAGCACATG--CACTGGAAACGGAT-TCTG----------

XAC29_22434|470464772_c51310-51050 ---------CGAGCG-----------------CAA-TCAG----------

XCAW_b00030|471265493_c28889-28173 CGCCGCTCGCGGGCGCAAGGGC----G-GACGCAAACCAGTCATCACCGC

** * * *

XACb0060|21264228_c52285-52025 ---------------GGCACG-----CAG---------------------

XCVd0101|78045309_c111830-111429 ---------------TCCTCGTTACCAAT---------------------

XAC29_22434|470464772_c51310-51050 ---------------GGCACG-----CAG---------------------

XCAW_b00030|471265493_c28889-28173 CGACAAGTTGCAGCAGGCACG-----TAAACACATCGCCAACGGGATAAA

* ** *

XACb0060|21264228_c52285-52025 -------GA--CCG---------------TGA--------TCCTTT--TG

XCVd0101|78045309_c111830-111429 -------GACGCCG----------CAT--TTAG-------TCGTGT--TG

XAC29_22434|470464772_c51310-51050 -------GA--CCG---------------TGA--------TCCTTT--TG

XCAW_b00030|471265493_c28889-28173 TGTCCGGGAGGCCGCTACACGGCTCAAGGTGAGTAAAACAGCCTTGTATG

** *** * * * * **

XACb0060|21264228_c52285-52025 C----------------------GGGAT-G-GC---GCGAATGA

XCVd0101|78045309_c111830-111429 TTGGTTTGAC-------C-GTGGAAGATTGGACA-CGCAGCTAA

XAC29_22434|470464772_c51310-51050 C----------------------GGGAT-G-GC---GCGAATGA

XCAW_b00030|471265493_c28889-28173 CAGCTCTG-CAATCCACCAGTGCAGGAG-A-TCTCCAAAAATGA

** * * *

PROTEIN ALIGNMENTS:

XACb0060|AAM39306.1|virulence MSQVA----------------------------KLFINGRSQA-------

XCVd0101|CAJ19913.1|conserved MKRYM-----------------------------LDTNTVS---------

XAC29_22434|AGH79841.1|virulence MSQVA----------------------------KLFINGRSQA-------

XCAW_b00030|AGI10550.1|invertase MFGIYQGVCLSDKRSFVGRAGRCPENCLCNGLSDTKIDGRNGGQDGWQMA

* : .

XACb0060|AAM39306.1|virulence ----VRLPAAYRFTEKEVFIRQD-----------PETGDVILSRKPATWE

XCVd0101|CAJ19913.1|conserved --------------------------------------------------

XAC29_22434|AGH79841.1|virulence ----VRLPAAYRFTEKEVFIRQD-----------PETGDVILSRKPATWE

XCAW_b00030|AGI10550.1|invertase LIGYARVSTA----EQDTALQTDALRKAGCERVFEDTASGAKADRPGL-A

XACb0060|AAM39306.1|virulence DFFIVLKSADVPAN------------------------------------

XCVd0101|CAJ19913.1|conserved -------------------------HLVKSHPAVSRRVIE-VPMTALCMS

XAC29_22434|AGH79841.1|virulence DFFIVLKSADVPAN------------------------------------

XCAW_b00030|AGI10550.1|invertase DALAYLRNGDVLAVWRLDRLGRSMPHLIETIGALEARGVGFRSLTEAIDT

XACb0060|AAM39306.1|virulence --------------------------------------------------

XCVd0101|CAJ19913.1|conserved AITGGELMFGLAK--------------------------------VPDAK

XAC29_22434|AGH79841.1|virulence --------------------------------------------------

XCAW_b00030|AGI10550.1|invertase TTPGGRLIFHVFGALGQFERDLIRERTKAGLSAAAARGRKGGRKPVITAD

XACb0060|AAM39306.1|virulence --------------FL-----------------DAAERNQ----------

XCVd0101|CAJ19913.1|conserved RLQQAVMELLRRVDVLPWDGAVMERYGSVRADLEKQGKALGSLDMLIAAH

XAC29_22434|AGH79841.1|virulence --------------FL-----------------DAAERNQ----------

XCAW_b00030|AGI10550.1|invertase KLQQARKHIANGINVR-----------------EAATRLKVSK-TAL---

. : :

XACb0060|AAM39306.1|virulence -------------------GTQDRDPFAGWR-E

XCVd0101|CAJ19913.1|conserved ALETDSVLVTNDAAFSRVVGLTVED----WTRS

XAC29_22434|AGH79841.1|virulence -------------------GTQDRDPFAGWR-E

XCAW_b00030|AGI10550.1|invertase --------------YAALQSTSAGD----LQK-

. *

**19 - DOMAIN cl00995 - superf. PemK / PRK09812 - ChpB**

NUCLEOTIDE ALIGNMENTS:

XACa0027|58033143_c18560-18225 GTGATGGATCGCGGCGATGTCTATTTGGTTTCGCTCGATCCCACGTCCGG

XAC29_22929|470474282_c30435-30103 ATG---GATCGCGGCGATGTCTATTTGGTTTCGCTCGATCCCACGTCCGG

** ********************************************

XACa0027|58033143_c18560-18225 CCATGAACAACAGGGCACGCGCCCGGTGCTGATTGTGTCGCCAAGCGCGT

XAC29_22929|470474282_c30435-30103 CCATGAACAACAGGGCACGCGCCCGGTGCTGATTGTGTCGCCAAGCGCGT

**************************************************

XACa0027|58033143_c18560-18225 TCAACCGCCTGACGAAAACACCGGTCGTGCTGCCGATCACCAGTGGGGGT

XAC29_22929|470474282_c30435-30103 TCAACCGCCTGACGAAAACACCGGTCGTGCTGCCGATCACCAGTGGGGGT

**************************************************

XACa0027|58033143_c18560-18225 AACTTCGCCCGCACGGCAGGCTTTACCGTGTCGCTCATGGGTGCGGGTAC

XAC29_22929|470474282_c30435-30103 AACTTCGCCCGCACGGCAGGCTTTACCGTGTCGCTCATGGGTGCGGGTAC

**************************************************

XACa0027|58033143_c18560-18225 CAACACAACTGGCGTAGTTCGCTGCGATCAGCCCCGCGCTCTCGATCTAG

XAC29_22929|470474282_c30435-30103 CAACACAACTGGCGTAGTTCGCTGCGATCAGCCCCGCGCTCTCGATCTAG

**************************************************

XACa0027|58033143_c18560-18225 CCTCGCGGCGCGCGCGCAAGCTGGAAACCGTGCCGTCCCCGATCATGGAT

XAC29_22929|470474282_c30435-30103 CCTCGCGGCGCGCGCGCAAGCTGGAAACCGTGCCGTCCCCGATCATGGAT

**************************************************

XACa0027|58033143_c18560-18225 GAGGTATTAGCGAAGCTGAGCGCGATCCTTGAGTAA

XAC29_22929|470474282_c30435-30103 GAGGTATTAGCGAAGCTGAGCGCGATCCTTGAGTAA

************************************

PROTEIN ALIGNMENTS:

XACa0027AAM39231.1|plasmid MMDRGDVYLVSLDPTSGHEQQGTRPVLIVSPSAFNRLTKTPVVLPITSGG

XAC29_22929|AGH79939.1|plasmid -MDRGDVYLVSLDPTSGHEQQGTRPVLIVSPSAFNRLTKTPVVLPITSGG

*************************************************

XACa0027|AAM39231.1|plasmid NFARTAGFTVSLMGAGTNTTGVVRCDQPRALDLASRRARKLETVPSPIMD

XAC29_22929|AGH79939.1|plasmid NFARTAGFTVSLMGAGTNTTGVVRCDQPRALDLASRRARKLETVPSPIMD

**************************************************

XACa0027|AAM39231.1|plasmid EVLAKLSAILE

XAC29_22929|AGH79939.1|plasmid EVLAKLSAILE

***********

**20 - DOMAIN RHH (recently described by Gallo, 2010)**

NUCLEOTIDE ALIGNMENTS:

XACa0037|58033143_c26043-25804 ATGAATACCGTGCGCTGGAACATCGCCGTATCGCCGGACGTGGATCAGTC

XCVb0017|78045242_c14988-14749 ATGAATACCGTGCGCTGGAACATCGCCGTATCGCCGGACGTGGATCAGTC

XAC29_22484|470464772_c62940-62701 ATGAATACCGTGCGCTGGAACATCGCCGTATCGCCGGACGTGGATCAGTC

XCAW_b00020|471265493_17583-17822 ATGAATACCGTGCGCTGGAACATCGCCGTATCGCCGGACGTGGATCAGTC

**************************************************

XACa0037|58033143_c26043-25804 CGTCCGCATGTTCATCGCCGCGCAAGGCGGCGGTCGCAAGGGCGACCTGT

XCVb0017|78045242_c14988-14749 CGTCCGCATGTTCATCGCCGCGCAAGGCGGCGGTCGCAAGGGCGACCTGT

XAC29_22484|470464772_c62940-62701 CGTCCGCATGTTCATCGCCGCGCAAGGCGGCGGTCGCAAGGGCGACCTGT

XCAW_b00020|471265493_17583-17822 CGTCCGCATGTTCATCGCCGCGCAAGGCGGCGGTCGCAAGGGCGACCTGT

**************************************************

XACa0037|58033143_c26043-25804 CACGCTTCATCGAGGATGCGGTGCGCGCCTACCTCTTCGAGCGGGCTGTG

XCVb0017|78045242_c14988-14749 CACGCTTCATCGAGGATGCGGTGCGCGCCTACCTCTTCGAGCGGGCTGTG

XAC29_22484|470464772_c62940-62701 CACGCTTCATCGAGGATGCGGTGCGCGCCTACCTCTTCGAGCGGGCTGTG

XCAW_b00020|471265493_17583-17822 CACGCTTCATCGAGGATGCGGTGCGCGCCTACCTCTTCGAGCGGGCTGTG

**************************************************

XACa0037|58033143_c26043-25804 GAACAAGCCAAAGCCGCTACGGTGGGTATGGGTGAGACAGAACTGAACGA

XCVb0017|78045242_c14988-14749 GAACAAGCCAAAGCCGCTACGGTGGGTATGGGTGAGACAGAACTGAACGA

XAC29_22484|470464772_c62940-62701 GAACAAGCCAAAGCCGCTACGGTGGGTATGGGTGAGACAGAACTGAACGA

XCAW_b00020|471265493_17583-17822 GAACAAGCCAAAGCCGCTACGGTGGGTATGGGTGAGACAGAACTGAACGA

**************************************************

XACa0037|58033143_c26043-25804 CCTCATTGATGAGGCGGTGCAATGGGCGCGTGAGCATTAA

XCVb0017|78045242_c14988-14749 CCTCATTGATGAGGCGGTGCAATGGGCGCGTGAGCATTAA

XAC29_22484|470464772_c62940-62701 CCTCATTGATGAGGCGGTGCAATGGGCGCGTGAGCATTAA

XCAW_b00020|471265493_17583-17822 CCTCATTGATGAGGCGGTGCAATGGGCGCGTGAGCATTAA

****************************************

PROTEIN ALIGNMENTS:

XACa0037|21110934|gb|AAM39316.1| MNTVRWNIAVSPDVDQSVRMFIAAQGGGRKGDLSRFIEDAVRAYLFERAV

XCVb0017|78033763|emb|CAJ19764.1| MNTVRWNIAVSPDVDQSVRMFIAAQGGGRKGDLSRFIEDAVRAYLFERAV

XAC29_22484|AGH79908.1| MNTVRWNIAVSPDVDQSVRMFIAAQGGGRKGDLSRFIEDAVRAYLFERAV

XCAW_b00020|AGI10540.1| MNTVRWNIAVSPDVDQSVRMFIAAQGGGRKGDLSRFIEDAVRAYLFERAV

**************************************************

XACa0037|21110934|gb|AAM39316.1| EQAKAATVGMGETELNDLIDEAVQWAREH

XCVb0017|78033763|emb|CAJ19764.1| EQAKAATVGMGETELNDLIDEAVQWAREH

XAC29_22484|AGH79908.1| EQAKAATVGMGETELNDLIDEAVQWAREH

XCAW_b00020|AGI10540.1| EQAKAATVGMGETELNDLIDEAVQWAREH

*****************************

**21 - DOMAIN COG1569 - with PIN domain**

NUCLEOTIDE ALIGNMENTS:

XACa0036|58033143_c25804-25385 ATGCGGGTCGTCCTCGACACCAACGTATTGCTGGCCGCGCTGATCTCGTC

XCVb0016|78045242_c14749-14330 ATGCGGGTCGTCCTCGACACCAACGTATTGCTGGCCGCGCTGATCTCGTC

XAC29_22479|470464772_c62701-62282 ATGCGGGTCGTCCTCGACACCAACGTATTGCTGGCCGCGCTGATCTCGTC

XCAW_b00021|471265493_17822-18241 ATGCGGGTCGTCCTCGACACCAACGTATTGCTGGCCGCGCTGATCTCGTC

**************************************************

XACa0036|58033143_c25804-25385 GCACAGCCCACCCGACATCATCTATCGCGGTTGGCTTGCAGCACGCTTTG

XCVb0016|78045242_c14749-14330 GCACAGCCCACCCGACATCATCTATCGCGGTTGGCTTGCAGCACGCTTTG

XAC29_22479|470464772_c62701-62282 GCACAGCCCACCCGACATCATCTATCGCGGTTGGCTTGCAGCACGCTTTG

XCAW_b00021|471265493_17822-18241 GCACAGCCCACCCGACATCATCTATCGCGGTTGGCTTGCAGCACGCTTTG

**************************************************

XACa0036|58033143_c25804-25385 AACTGGTGACAGGGACGGCGCAGCTTGATGAACTGCGCCGCGTGAGCCGT

XCVb0016|78045242_c14749-14330 AACTGGTGACAGGGACGGCGCAGCTTGATGAACTGCGCCGCGTGAGCCGT

XAC29_22479|470464772_c62701-62282 AACTGGTGACAGGGACGGCGCAGCTTGATGAACTGCGCCGCGTGAGCCGT

XCAW_b00021|471265493_17822-18241 AACTGGTGACAGGGACGGCGCAGCTTGATGAACTGCGCCGCGTGAGCCGT

**************************************************

XACa0036|58033143_c25804-25385 TACCCGAAGATCAAGGCAATCCTGCCCGCGCATCGCGTCGGCACGATGAT

XCVb0016|78045242_c14749-14330 TACCCGAAGATCAAGGCAATCCTGCCCGCGCATCGCGTCGGCACGATGAT

XAC29_22479|470464772_c62701-62282 TACCCGAAGATCAAGGCAATCCTGCCCGCGCATCGCGTCGGCACGATGAT

XCAW_b00021|471265493_17822-18241 TACCCGAAGATCAAGGCAATCCTGCCCGCGCATCGCGTCGGCACGATGAT

**************************************************

XACa0036|58033143_c25804-25385 CAACAACATGCAGCGCGCCGTTGTGCTGCATGTATTGCCGCCTCTGCCTG

XCVb0016|78045242_c14749-14330 CAACAACATGCAGCGCGCCGTTGTGCTGCATGTATTGCCGCCTCTGCCTG

XAC29_22479|470464772_c62701-62282 CAACAACATGCAGCGCGCCGTTGTGCTGCATGTATTGCCGCCTCTGCCTG

XCAW_b00021|471265493_17822-18241 CAACAACATGCAGCGCGCCGTTGTGCTGCATGTATTGCCGCCTCTGCCTG

**************************************************

XACa0036|58033143_c25804-25385 ATCGCATCGAGGTCAATGATCCGAACGATGCGTTCCTGCTGGCGATGGCA

XCVb0016|78045242_c14749-14330 ATCGCATCGAGGTCAATGATCCGAACGATGCGTTCCTGCTGGCGATGGCA

XAC29_22479|470464772_c62701-62282 ATCGCATCGAGGTCAATGATCCGAACGATGCGTTCCTGCTGGCGATGGCA

XCAW_b00021|471265493_17822-18241 ATCGCATCGAGGTCAATGATCCGAACGATGCGTTCCTGCTGGCGATGGCA

**************************************************

XACa0036|58033143_c25804-25385 CTGGCCAGCGAGGCCGATTACCTTGTGACTGGCGACCGCCGCGCTGGGCT

XCVb0016|78045242_c14749-14330 CTGGCCAGCGAGGCCGATTACCTTGTGACTGGCGACCGCCGCGCTGGGCT

XAC29_22479|470464772_c62701-62282 CTGGCCAGCGAGGCCGATTACCTTGTGACTGGCGACCGCCGCGCTGGGCT

XCAW_b00021|471265493_17822-18241 CTGGCCAGCGAGGCCGATTACCTTGTGACTGGCGACCGCCGCGCTGGGCT

**************************************************

XACa0036|58033143_c25804-25385 GCTGCAACGCGGTAGCATTGGCCGCACGCGCATCGTCACGCCAGTCACCT

XCVb0016|78045242_c14749-14330 GCTGCAACGCGGTAGCATTGGCCGCACGCGCATCGTCACGCCAGTCACCT

XAC29_22479|470464772_c62701-62282 GCTGCAACGCGGTAGCATTGGCCGCACGCGCATCGTCACGCCAGTCACCT

XCAW_b00021|471265493_17822-18241 GCTGCAACGCGGTAGCATTGGCCGCACGCGCATCGTCACGCCAGTCACCT

**************************************************

XACa0036|58033143_c25804-25385 TCTGCGCCGAGGCGCTTTGA

XCVb0016|78045242_c14749-14330 TCTGCGCCGAGGCGCTTTGA

XAC29_22479|470464772_c62701-62282 TCTGCGCCGAGGCGCTTTGA

XCAW_b00021|471265493_17822-18241 TCTGCGCCGAGGCGCTTTGA

********************

PROTEIN ALIGNMENTS:

XACa0036|AAM39315.1|conserved MRVVLDTNVLLAALISSHSPPDIIYRGWLAARFELVTGTAQLDELRRVSR

XCVb0016|CAJ19807.1|conserved MRVVLDTNVLLAALISSHSPPDIIYRGWLAARFELVTGTAQLDELRRVSR

XAC29_22479|AGH79907.1|PilT MRVVLDTNVLLAALISSHSPPDIIYRGWLAARFELVTGTAQLDELRRVSR

XCAW_b00021|AGI10588.1|hypothetical MRVVLDTNVLLAALISSHSPPDIIYRGWLAARFELVTGTAQLDELRRVSR

**************************************************

XACa0036|AAM39315.1|conserved YPKIKAILPAHRVGTMINNMQRAVVLHVLPPLPDRIEVNDPNDAFLLAMA

XCVb0016|CAJ19807.1|conserved YPKIKAILPAHRVGTMINNMQRAVVLHVLPPLPDRIEVNDPNDAFLLAMA

XAC29_22479|AGH79907.1|PilT YPKIKAILPAHRVGTMINNMQRAVVLHVLPPLPDRIEVNDPNDAFLLAMA

XCAW_b00021|AGI10588.1|hypothetical YPKIKAILPAHRVGTMINNMQRAVVLHVLPPLPDRIEVNDPNDAFLLAMA

**************************************************

XACa0036|AAM39315.1|conserved LASEADYLVTGDRRAGLLQRGSIGRTRIVTPVTFCAEAL

XCVb0016|CAJ19807.1|conserved LASEADYLVTGDRRAGLLQRGSIGRTRIVTPVTFCAEAL

XAC29_22479|AGH79907.1|PilT LASEADYLVTGDRRAGLLQRGSIGRTRIVTPVTFCAEAL

XCAW_b00021|AGI10588.1|hypothetical LASEADYLVTGDRRAGLLQRGSIGRTRIVTPVTFCAEAL

***************************************

**22 - DOMAIN pfam02452 - PemK -like proteins**

NUCLEOTIDE ALIGNMENTS:

XACb0032|21264228_c31716-31393 ATG---CGGGGTGACTTTGTGACCATCGCCATGCAAGGCGACTTCGGAAA

XCVc0023|78045265_c19486-19160 ATGATGCGAGGTGACTTTGTGACCATCGCCATGCAAGGCGACTTCGGAAA

XAC29_22294|470464772_c31455-31132 ATG---CGGGGTGACTTTGTGACCATCGCCATGCAAGGCGACTTCGGAAA

*** ** *****************************************

XACb0032|21264228_c31716-31393 ACCAAGGCCCGCTCTGGTGATCCAGGCCGACCAGTTTGACGCACACACCA

XCVc0023|78045265_c19486-19160 ACCACGGCCCGCTCTGGTCATCCAGGCCAACCAGTTTGACGAACACACCA

XAC29_22294|470464772_c31455-31132 ACCAAGGCCCGCTCTGGTGATCCAGGCCGACCAGTTTGACGCACACACCA

**** ************* ********* ************ ********

XACb0032|21264228_c31716-31393 CCGTGACGGTGCTGCCGGTGACAAGCACGCTCGTTGCTGCGCCGCTGCTG

XCVc0023|78045265_c19486-19160 CCGTGACGGTGCTGCCGGTGACAAGCACGCTTGTTGCTGCGCCGCTGCTG

XAC29_22294|470464772_c31455-31132 CCGTGACGGTGCTGCCGGTGACAAGCACGCTCGTTGCTGCGCCGCTGCTG

******************************* ******************

XACb0032|21264228_c31716-31393 CGTATTACCGTTCATCCAAGTACCGACAATGGTTTGCAGAAGCCGTCTCA

XCVc0023|78045265_c19486-19160 CGCATTACCGTTCATCCAAGTACCGACAATGGCCTGCTGAAGCCGTCTCA

XAC29_22294|470464772_c31455-31132 CGTATTACCGTTCATCCAAGTACCGACAATGGTTTGCAGAAGCCGTCTCA

** ***************************** *** ************

XACb0032|21264228_c31716-31393 GGTGATGGTGGACAAGGCCATGACCGTGAAGCGTGACAAGGTAGGTCGAG

XCVc0023|78045265_c19486-19160 GGTGATGGTGGACAAGGCCATGACCGTGAAGCGTGACAAGATAGGTCGGG

XAC29_22294|470464772_c31455-31132 GGTGATGGTGGACAAGGCCATGACCGTGAAGCGTGACAAGGTAGGTCGAG

**************************************** ******* *

XACb0032|21264228_c31716-31393 CCTTTGGGCGCGTAGACGCTGATGCGTTGGTGGAAATTGAGCGCTGCCTG

XCVc0023|78045265_c19486-19160 CCTTTGGGCGCGTAGACGCTGATGCGTTGGTGGAAATTGAGCGCTGCCTG

XAC29_22294|470464772_c31455-31132 CCTTTGGGCGCGTAGACGCTGATGCGTTGGTGGAAATTGAGCGCTGCCTG

**************************************************

XACb0032|21264228_c31716-31393 GCCGTGTTCTTGGGCATCGCTAAGTGA

XCVc0023|78045265_c19486-19160 GCCGTGTTCTTGGGCATCGCTAAGTGA

XAC29_22294|470464772_c31455-31132 GCCGTGTTCTTGGGCATCGCTAAGTGA

***************************

PROTEIN ALIGNMENTS:

XACb0032|AAM39278.1|conserved... M-RGDFVTIAMQGDFGKPRPALVIQADQFDAHTTVTVLPVTSTLVAAPLL

XCVc0023|CAJ19792.1|putative... MMRGDFVTIAMQGDFGKPRPALVIQANQFDEHTTVTVLPVTSTLVAAPLL

XAC29_22294|WP_011052979.1|... M-RGDFVTIAMQGDFGKPRPALVIQADQFDAHTTVTVLPVTSTLVAAPLL

* ************************:*** *******************

XACb0032|AAM39278.1|conserved... RITVHPSTDNGLQKPSQVMVDKAMTVKRDKVGRAFGRVDADALVEIERCL

XCVc0023|CAJ19792.1|putative... RITVHPSTDNGLLKPSQVMVDKAMTVKRDKIGRAFGRVDADALVEIERCL

XAC29_22294|WP_011052979.1|... RITVHPSTDNGLQKPSQVMVDKAMTVKRDKVGRAFGRVDADALVEIERCL

************ *****************:*******************

XACb0032|AAM39278.1|conserved... AVFLGIAK

XCVc0023|CAJ19792.1|putative... AVFLGIAK

XAC29_22294|WP_011052979.1|... AVFLGIAK

********

**23 - DOMAIN pfam11455 - DUF3018**

NUCLEOTIDE ALIGNMENTS:

XACb0033|21264228_c31934-31716 ATG-----------------------------------------------

XCVc0024|78045265_c19809-19483 GTGTCGGCGGCTTCTTGGGGCACCAAGCCGGAACCACAGAAAGTCCCGCT

**

XACb0033|21264228_c31934-31716 --------------------------------------------------

XCVc0024|78045265_c19809-19483 TTTGTTGCATGTAACAAAAATGAGGCGTAAACTTGTTGCATGTAACGGTG

XACb0033|21264228_c31934-31716 -----------GCACACGTCAATTCACGAGTTCAAAAGCACCGCGACGCC

XCVc0024|78045265_c19809-19483 AGGCCAACATGGCACACGTCAATTCACGAGTTCAAAAGCACCGTGACGCC

******************************** ******

XACb0033|21264228_c31934-31716 CTGCGCATGGCAGGGCTGCGTCCGGTGCAAATTTGGGTGCCGGACACACG

XCVc0024|78045265_c19809-19483 CTGCGCATGGCAGGGCTGCGTCCGGTGCAAATTTGGGTGCCGGACACACG

**************************************************

XACb0033|21264228_c31934-31716 GCGGCCTGACTTCGCCGAGGAATGCCGCCGTCAGTGTCGTCTTGCTGCAC

XCVc0024|78045265_c19809-19483 GCGGCCTGACTTCGCCGAGGAATGCCGCCGTCAGTGTCGTCTTGCTGCAC

**************************************************

XACb0033|21264228_c31934-31716 AAGCGGACATGGCGGATACCGACATGCAGCGCTTCATGGATGAGGCGCTA

XCVc0024|78045265_c19809-19483 AAGCGGACATGGAGGACACCGACATGCAGCGCTTCATGGATGAGGCGCTA

************ *** *********************************

XACb0033|21264228_c31934-31716 GCAGACATGGATGGCTGGACGGAATGA

XCVc0024|78045265_c19809-19483 GCAGACATGGATGGCTGGACGGAATGA

***************************

PROTEIN ALIGNMENTS:

XACb0033|AAM39279.1|conserved ------------------------------------MAHVNSRVQKHRDA

XCVc0024|CAJ19793.1|conserved MSAASWGTKPEPQKVPLLLHVTKMRRKLVACNGEANMAHVNSRVQKHRDA

**************

XACb0033|AAM39279.1|conserved LRMAGLRPVQIWVPDTRRPDFAEECRRQCRLAAQADMADTDMQRFMDEAL

XCVc0024|CAJ19793.1|conserved LRMAGLRPVQIWVPDTRRPDFAEECRRQCRLAAQADMEDTDMQRFMDEAL

************************************* ************

XACb0033|AAM39279.1|conserved ADMDGWTE

XCVc0024|CAJ19793.1|conserved ADMDGWTE

********

**24 - DOMAIN spoVT_AbrB/MazE**

Nucleotide alignments:

XCC1631|21229478_1901388-1901687 TTGC----------------------------------------------

XC_2600|66766352_c3138533-3138141 ATGTCCGAAAACCCGTTAAGCAAGTGCATTGCCGGACAATGTCCGGCATG

XALr_3245|386076359_c7997-7731 ATG-----------------------------------------------

XAC29_22829|470474282_15953-16204 ATG-----------------------------------------------

**

XCC1631|21229478_1901388-1901687 -----------------------------------------------ATT

XC_2600|66766352_c3138533-3138141 GCCGACTAATGATCGTTTGACGGACATTGAAAGTAAGGCAGTTTTGCATT

XALr_3245|386076359_c7997-7731 --------------------------------------------------

XAC29_22829|470474282_15953-16204 --------------------------------------------------

XCC1631|21229478_1901388-1901687 ACAATGCAAAGCATCAACCTTGGATTGCGAGGCAAACCATCATGACCACA

XC_2600|66766352_c3138533-3138141 ACAATGCAAAGCATCAACCTTGGATTGCGAGGCAAACCATCATGACCACA

XALr_3245|386076359_c7997-7731 --------------------------------------------AGCACA

XAC29_22829|470474282_15953-16204 --------------------------------------------ACCACA

* ****

XCC1631|21229478_1901388-1901687 TTGACCGTTACCGCACGGGGACAAGTGACGTTTCGGAAGGACGTACTGCA

XC_2600|66766352_c3138533-3138141 TTGACCGTTACCGCACGGGGACAAGTGACGTTTCGGAAGGACGTACTGCA

XALr_3245|386076359_c7997-7731 CTAACCGTCACCACGCGCGGCCAGGTGACATTTCGCGCCGAGGTATTGCA

XAC29_22829|470474282_15953-16204 TTGACCGTTACCGCACGTGGACAAGTGACGTTTCGGAAGGACGTACTGCA

* ***** *** * ** ** ** ***** ***** ** *** ****

XCC1631|21229478_1901388-1901687 ACACCTCGGCATCAGGCCAGGCGACAAGATCGAACTGAACTTGCTGCCAG

XC_2600|66766352_c3138533-3138141 ACACCTCGGCATCAGGCCAGGCGACAAGATCGAACTGAACTTGCTGCCAG

XALr_3245|386076359_c7997-7731 GCACCTCGGCATCAAGCCAGGCGAGAAGATCGAGGTGTATTTGATGCCCG

XAC29_22829|470474282_15953-16204 ACACCTCGGCATTAGGCCAGGCGACAAGATCGAGCTTGACTTGCTGCCAG

*********** * ********* ******** * * *** **** *

XCC1631|21229478_1901388-1901687 ATGGTCGGGGCGTGCTCAAGGCGGCCCGGCCCGCAGGGACGAT--AGCCA

XC_2600|66766352_c3138533-3138141 ATGGTCGGGGCGTGCTCAAGGCGGCCCGGCCCGCAGGGACGAT--AGCCA

XALr_3245|386076359_c7997-7731 ACGGTCGGGCCGAGCTTAAAGCGGCAAA-ACCGAAGGGTTCATTCAGGGA

XAC29_22829|470474282_15953-16204 ATGGTCGGGGAGTACTCAAGGCGGCACGGCCCGCAGGGACGAT--AGCCA

* ******* * ** ** ***** *** **** ** ** *

XCC1631|21229478_1901388-1901687 GCTTTGTCGGCCTGCTCGCGGGCAGGACGCAGAAG-GTTG--CAACCATC

XC_2600|66766352_c3138533-3138141 GCTTTGTCGGCCTGCTCGCGGGCAGGACGCAGAAG-GTTG--CAACCATC

XALr_3245|386076359_c7997-7731 GCTGCG-GGGCATCCTCAAGCACAAGACGAACGGCGCTCGGTTGAGCATC

XAC29_22829|470474282_15953-16204 GCTTTGTCGGCCTGCTCGCAGGTAAGACGCAGAAG-GTTG--CCACCATC

*** * *** * *** * **** * * * * ****

XCC1631|21229478_1901388-1901687 GAAGAAATCAACGAGGCGGCGGCGCAAG--------GCT----GGGCAGG

XC_2600|66766352_c3138533-3138141 GAAGAAATCAACGAGGCGGCGGCGCAAG--------GCT----GGGCAGG

XALr_3245|386076359_c7997-7731 GAAGAAATCAACGATGCAATAGCCGAAGCCGGTGATGCTGCCGGGACAGG

XAC29_22829|470474282_15953-16204 GAAGAAATCAACGAGGCGGCTGCACAAG--------GAT----GGGCAGG

************** ** ** *** * * ** ****

XCC1631|21229478_1901388-1901687 TAAGCAATGA

XC_2600|66766352_c3138533-3138141 TAAGCAATGA

XALr_3245|386076359_c7997-7731 CAACACATGA

XAC29_22829|470474282_15953-16204 CAAGCAATGA

** ****

PROTEIN ALIGNMENTS:

XCC1631|AAM40925.1|conserved... -------------------------------MHYNAKHQPWIARQTIMTT

XC_2600|AAY49649.1|conserved... MSENPLSKCIAGQCPAWPTNDRLTDIESKAVLHYNAKHQPWIARQTIMTT

XALr_3245|WP_014607045.1|trans... -----------------------------------------------MST

XAC29_22829|AGH79919.1|regulator -----------------------------------------------MTT

*:*

XCC1631|AAM40925.1|conserved... LTVTARGQVTFRKDVLQHLGIRPGDKIELNLLPDGRGVLKAARPAGTIAS

XC_2600|AAY49649.1|conserved... LTVTARGQVTFRKDVLQHLGIRPGDKIELNLLPDGRGVLKAARPAGTIAS

XALr_3245|WP_014607045.1|trans... LTVTTRGQVTFRAEVLQHLGIKPGEKIEVYLMPDGRAELKAAKPKGSFRE

XAC29_22829|AGH79919.1|regulator LTVTARGQVTFRKDVLQHLGIRPGDKIELDLLPDGRGVLKAARPAGTIAS

****:******* :*******:**:***: *:****. ****:* *:: .

XCC1631|AAM40925.1|conserved... FVGLLAGRTQKV-ATIEEINEAAAQGW--AGK--Q

XC_2600|AAY49649.1|conserved... FVGLLAGRTQKV-ATIEEINEAAAQGW--AGK--Q

XALr_3245|WP_014607045.1|trans... LRGILKHKTNGARLSIEEINDAIAEAGDAAGTGNT

XAC29_22829|AGH79919.1|regulator FVGLLAGKTQKV-ATIEEINEAAAQGW--AGK--Q

: *:* :*: . :*****:* *:. **.

**25 - DOMAIN cd09861 PIN-VapC-like**

NUCLEOTIDE ALIGNMENTS:

XCC1632|21229478_1901684-1902079 ATGAAGGTCGCCGCCGATACCAACGTCCTTGTGCGTGCGGTTGTGCGTGA

XC_2599|66766352_c3138144-3137749 ATGAAGGTCGCCGCCGATACCAACGTCCTTGTGCGTGCGGTTGTGCGTGA

XALr_3244|386076359_c7734-7339 ATGAAAGTGACCGCTGATACAAACGTTCTGCTTCGTACCATTGTTGAGGA

XAC29_22834|470474282_16201-16596 ATGAAGGTCGCAGTCGATACCAACGTCCTTGTGCGTGCTGTTGTGCGTGA

***** ** * * ***** ***** ** * *** * **** **

XCC1632|21229478_1901684-1902079 CGATCCCGCACAAGCGGACGTTGCCGCCGCAGTCTTGACCGACGCCGAGT

XC_2599|66766352_c3138144-3137749 CGATCCCGCACAAGCGGACGTTGCCGCCGCAGTCTTGACCGACGCCGAGT

XALr_3244|386076359_c7734-7339 TGATGAAATCCAAACAAAGCGCGCCGTCGAACTACTGGAAACTGCGGATA

XAC29_22834|470474282_16201-16596 CGATCCCGCACAAGCGGACGTTGCCGCCGCAGTCTTGACCGATGCCGAGT

*** *** * * **** ** * * ** ** **

XCC1632|21229478_1901684-1902079 TGATCGCGGTCGCGTTGCCGTGCCTATGTGAATTTGTTTGGGTGCTGCTG

XC_2599|66766352_c3138144-3137749 TGATCGCGGTCGCGTTGCCGTGCCTATGTGAATTTGTTTGGGTGCTGCTG

XALr_3244|386076359_c7734-7339 TGGTAGCTGTGAGCCTGCAAACACTCTGCGAGGTTGTATGGGTGCTGCGC

XAC29_22834|470474282_16201-16596 TGATCGCGGTCGCGCTGCCGTGCCTATGCGAATTTGTTTGGGTGCTGATG

** * ** ** *** ** ** ** **** *********

XCC1632|21229478_1901684-1902079 CGAGTCTACGGCTTCCAGCAAGCCGACGCGGCCAGCGCGATCCGGGCACT

XC_2599|66766352_c3138144-3137749 CGAGTCTACGGCTTCCAGCAAGCCGACGCGGCCAGCGCGATCCGGGCACT

XALr_3244|386076359_c7734-7339 GGCCGGTATGGTGTGGAGCGCACGGACGTTGCCGCCGCGATCCGGACACT

XAC29_22834|470474282_16201-16596 CGCGTCTATGGCTTCCAGCAATCTGACGCGGCCGATGCGATCCGGGCGCT

* ** ** * *** * **** *** ********* * **

XCC1632|21229478_1901684-1902079 ACTGGCCGCCGCGAATGTGGAAGTGAACCGGCCTGCCGTGGAGGCTGGCT

XC_2599|66766352_c3138144-3137749 ACTGGCCGCCGCGAATGTGGAAGTGAACCGGCCTGCCGTGGAGGCTGGCT

XALr_3244|386076359_c7734-7339 GTTGGACACACGCAATGTTGTCACGAATCGCCCAGCCGCCGAAGCTGGCC

XAC29_22834|470474282_16201-16596 ACTGGCCGCCGCCAACGTGGAAGTGAACCGGCCCGCCGTGGAGGCCGGTT

*** * * ** ** * *** ** ** **** ** ** **

XCC1632|21229478_1901684-1902079 TGCTGGTGCTCGACGCGGGCGGAGACTTTGCCGATGGCGTCATTGCCTAC

XC_2599|66766352_c3138144-3137749 TGCTGGTGCTCGACGCGGGCGGAGACTTTGCCGATGGCGTCATTGCCTAC

XALr_3244|386076359_c7734-7339 TAAAGTTGTTGGATGCAGGCGGCGATTTTGCCGATGGCGTCATTGCGTTC

XAC29_22834|470474282_16201-16596 TGCTGGTGCTCGACGCAGGCGGTGACTTTGCCGATGGCGTCATTGCCTAC

* * ** * ** ** ***** ** ******************** * *

XCC1632|21229478_1901684-1902079 GAAGGCAACTGGCTTGGCGGGGAAACCTTCGTTTCCTTCGATAAGAAGGC

XC_2599|66766352_c3138144-3137749 GAAGGCAACTGGCTTGGCGGGGAAACCTTCGTTTCCTTCGATAAGAAGGC

XALr_3244|386076359_c7734-7339 GACGGTACGTGGCTTGGCGCTGAAACGTTCGTTTCTTTCGATAAGAAAGC

XAC29_22834|470474282_16201-16596 GAAGGTAATTGGCTTGGTGGGGAAACCTTCGTTTCCTTCGATAAGAAGGC

** ** * ******** * ***** ******** *********** **

XCC1632|21229478_1901684-1902079 GGTGGTACTTCTCACAGCGCAAGGGCAATCAACGCGCCTTTTGTGA

XC_2599|66766352_c3138144-3137749 GGTGGTACTTCTCACAGCGCAAGGGCAATCAACGCGCCTTTTGTGA

XALr_3244|386076359_c7734-7339 AGTATCACTTTTGATAAAGCATGGACAATCCGCGCTGCTGCTTTGA

XAC29_22834|470474282_16201-16596 GGTGACACTTCTCTCTGTCCAAGGGCAATCAGCGCGTCTTTTGTGA

** **** * ** ** ***** *** ** * ***

PROTEIN ALIGNMENTS:

XCC1632|AAM40926.1|conserved MKVAADTNVLVRAVVRDDPAQADVAAAVLTDAELIAVALPCLCEFVWVLL

XC_2599|AAY49648.1|conserved MKVAADTNVLVRAVVRDDPAQADVAAAVLTDAELIAVALPCLCEFVWVLL

XALr_3244|WP_014607044.1|hypot.. MKVTADTNVLLRTIVEDDEIQTKRAVELLETADMVAVSLQTLCEVVWVLR

XAC29_22834|AGH79920.1|hypot... MKVAVDTNVLVRAVVRDDPAQADVAAAVLTDAELIAVALPCLCEFVWVLM

***:.*****:*::*.** *:. *. :* *:::**:* ***.****

XCC1632|AAM40926.1|conserved RVYGFQQADAASAIRALLAAANVEVNRPAVEAGLLVLDAGGDFADGVIAY

XC_2599|AAY49648.1|conserved RVYGFQQADAASAIRALLAAANVEVNRPAVEAGLLVLDAGGDFADGVIAY

XALr_3244|WP_014607044.1|hypot... GRYGVERTDVAAAIRTLLDTRNVVTNRPAAEAGLKLLDAGGDFADGVIAF

XAC29_22834|AGH79920.1|hypot... RVYGFQQSDAADAIRALLAAANVEVNRPAVEAGLLVLDAGGDFADGVIAY

**.:::*.* ***:** : ** .****.**** :*************:

XCC1632|AAM40926.1|conserved EGNWLGGETFVSFDKKAVVLLTAQGQSTRLL

XC_2599|AAY49648.1|conserved EGNWLGGETFVSFDKKAVVLLTAQGQSTRLL

XALr_3244|WP_014607044.1|hypot... DGTWLGAETFVSFDKKAVSLLIKHGQSALLL

XAC29_22834|AGH79920.1|hypot... EGNWLGGETFVSFDKKAVTLLSVQGQSARLL

:*.***.*********** ** :***: **

**26 - DOMAIN cl21503 - Plasmid_stabil super family / COG3549 - HigB**

NUCLEOTIDE ALIGNMENTS:

XCV4440|78045556_c5115984-5115766 TTGCC--------T------------------------------------

PXO_03903|188574270_334885-335175 ATGAC--G-GTCCTACCCAATATCCATCCTGGCGACATTCTGCTTGAGGA

XOC_0102|384417079_c100157-99876 ATGATCAGGAGCTTTATCGACAAGGACGCCGAAAAGATCTGGCTGGGTGA

** *

XCV4440|78045556_c5115984-5115766 -----------------------GCCGACATCCAATTGGTCGCGCG-CCG

PXO_03903|188574270_334885-335175 GTTTCTCGAGCCTCTGGGCATCAGCCAGAATGCACTGGCACGCGCCACCG

XOC_0102|384417079_c100157-99876 GCGTTC----CCGCCGGTTGCCTGCCGACATCCAATTGGTCGCGCG-CCG

*** ** ** * * ***** ***

XCV4440|78045556_c5115984-5115766 ----CAAGTTGCGCATGCTCAATG-----CCGCCGCGCAT-CTCGACAA-

PXO_03903|188574270_334885-335175 GCGTCCCGCCGCGCCGTATCAACGAAATCGTGCTGGGCAAGCGCGGCATC

XOC_0102|384417079_c100157-99876 ----CAAGTTGCGCATGCTCAATG-----CCGCCGCGCAT-CTCGACGA-

* * **** **** * ** * *** * ** *

XCV4440|78045556_c5115984-5115766 TCTGCCGATTCCGCCCGCCAACCGGCTGGAAGCGTCGAAGGGCGAGC-GG

PXO_03903|188574270_334885-335175 ACTGCCGACACTGCCGTGCGTCTGGCCG-CCGCGTTGGGC-ACCTCCGAG

XOC_0102|384417079_c100157-99876 TCTGCGGATCCCGCCCGCCAACCGGCTGGAAGCGTTGAAGGGCCAGC-GG

**** ** * *** * * *** * **** * * * *

XCV4440|78045556_c5115984-5115766 CCCGGCCAGTACCGCATCCGGATCAACGATCAA-TGGCGCATCTGCTTCC

PXO_03903|188574270_334885-335175 CGCTTCTGGCTGGGCCTGCAGGCCGACTACGAGCTGGAACAGGCGCATCG

XOC_0102|384417079_c100157-99876 CGCGGCCAGTACAGCATCCGGATCAACGATCAA-TGGCGCATCTGCTTCC

* * * * ** * * * * ** * * *** ** ** **

XCV4440|78045556_c5115984-5115766 GATGGATGGAAGGCGATGTGGTCCAGGTCGAAATCGTCGATTACC-A-CT

PXO_03903|188574270_334885-335175 CG----CGCTGGGCGATTTG--CCGGCACGGATCAAGCGGCTGGCGGCGT

XOC_0102|384417079_c100157-99876 GATGGATGGAAGGCGATGTGGTCCAGGTCGAAATCGTCGATTACC-A-CT

* ****** ** ** * ** * ** * * *

XCV4440|78045556_c5115984-5115766 GA

PXO_03903|188574270_334885-335175 GA

XOC_0102|384417079_c100157-99876 GA

**

PROTEIN ALIGNMENTS:

XCV4440|CAJ26171.1|conserved ---------------------MPADIQLVARRKLRMLNAAAHLDNLPIPP

PXO_03903|ACD56976.1|addiction MT--------------VLPNIHPGDILLEEFLEPLGISQNALARATGVPP

XOC_0102|AEQ94360.1| MIRSFIDKDAEKIWLGERSRRLPADIQLVARRKLRMLNAAAHLDDLRIPP

*.** * : :. * :**

XCV4440|CAJ26171.1|conserved ANRLEASKGER--PGQYRIRINDQWRICFRWMEGDVVQVEIVDYH-----

PXO_03903|ACD56976.1|addiction RRINEIVLGKRGITADTAVRLAAALGTSERFWLGLQADYELEQAHRALGD

XOC_0102|AEQ94360.1| ANRLEALKGQR--RGQYSIRINDQWRICFRWMEGDVVQVEIVDYH-----

. * *:* .: :*: . *: * .: *: : *

XCV4440|CAJ26171.1|conserved ----------

PXO_03903|ACD56976.1|addiction LPARIKRLAA

XOC_0102|AEQ94360.1| ----------

**27 - DOMAIN cl22461 - RHH_1 COG3905**

NUCLEOTIDE ALIGNMENTS:

XC_1065|66766352_1282754-1283248 GTGGAAAGCCGCCTTCGGGCGGCTTTCTGCGTTTTGGGCGATGTCTTACC

XCV3347|78045556_c3832596-3832252 ATGCAA--------------------------------------------

** **

XC_1065|66766352_1282754-1283248 TGGATGGCGGGAATTGCTGACTGCCCCGCGCGCGTTGGGCCGACTGCCCG

XCV3347|78045556_c3832596-3832252 --------------------------------------------------

XC_1065|66766352_1282754-1283248 GCTCCCTGCTTGCAGCGATGCTGAGCTCTGAATCGCACAAGGAGCGTGTC

XCV3347|78045556_c3832596-3832252 --------------------------------------------------

XC_1065|66766352_1282754-1283248 ATGCAAAGTGTGTCACAAGTGTGCTACTGTGAGCCTGTCATCCGGTCTTC

XCV3347|78045556_c3832596-3832252 ------AGTGTGTCACAAGTGTGCTACTTTGGGTCTGCCATTCGGTCCCC

********************** ** * *** *** ***** *

XC_1065|66766352_1282754-1283248 CGAGGTTGCCATGTCCACGACCACAATCCGTTTGCCTGACGAACTGAAAG

XCV3347|78045556_c3832596-3832252 CGAGGTTGCCATGTCCACGACCACCATCCGTCTGCCCGACGAACTGAAAG

************************ ****** **** *************

XC_1065|66766352_1282754-1283248 CGCGTGTCGCCAGGGCGGCGAAAGATGCCGGAACTACGGCGCACGGCTTT

XCV3347|78045556_c3832596-3832252 CGCGTGTCGCCAGGGCGGCGAAAGATGCCGGAACCACGGCGCACGGCTTT

********************************** ***************

XC_1065|66766352_1282754-1283248 ATCGTCGACGCCATTGCGGAAAAAATCGAGGCGCACGAGCGCCGCACTGC

XCV3347|78045556_c3832596-3832252 ATCGTCGACGCCATCGCGGAAAAAATCGATGCATACGAGCGCCGCACAGC

************** ************** ** ************* **

XC_1065|66766352_1282754-1283248 ATTCCATGCCGAAGCGCAGCAACGCTGGGCTGCTATCGAAGGCGGCGACA

XCV3347|78045556_c3832596-3832252 ATTTCATGCCGAGGCGCAGCAACGCTGGGCTGCCATCCAGGAGGGCGCCA

*** ******** ******************** *** * * **** **

XC_1065|66766352_1282754-1283248 ACACGCTGTCCTGGGCGGACGTACGCCAGTACATGCAGCAGCTTGCTGAC

XCV3347|78045556_c3832596-3832252 ACACGCTGTCCTGGGCGGACGTACGCAAGTACATGCAGCAACTTGCCGAC

************************** ************* ***** ***

XC_1065|66766352_1282754-1283248 GGAGAGAACCCCGCGCCACCGGTCGCCAGCGCCGTCGAGCGCTGA

XCV3347|78045556_c3832596-3832252 GGAGACGACCCCGCGCCACCGGTGGCCAGCGCCGTCGAGCGCTGA

***** **************** *********************

PROTEIN ALIGNMENTS:

XC_1065|AAY48135.1|hypothetical MESRLRAAFCVLGDVLPGWRELLTAPRALGRLPGSLLAAMLSSESHKERV

XCV3347|CAJ25078.1|hypothetical --------------------------------------------------

XC_1065|AAY48135.1|hypothetical MQSVSQVCYCEPVIRSSEVAMSTTTIRLPDELKARVARAAKDAGTTAHGF

XCV3347|CAJ25078.1|hypothetical MQSVSQVCYFGSAIRSPEVAMSTTTIRLPDELKARVARAAKDAGTTAHGF

********* ..***.*********************************

XC_1065|AAY48135.1|hypothetical IVDAIAEKIEAHERRTAFHAEAQQRWAAIEGGDNTLSWADVRQYMQQLAD

XCV3347|CAJ25078.1|hypothetical IVDAIAEKIDAYERRTAFHAEAQQRWAAIQEGANTLSWADVRKYMQQLAD

*********:*:*****************: * *********:*******

XC_1065|AAY48135.1|hypothetical GENPAPPVASAVER

XCV3347|CAJ25078.1|hypothetical GDDPAPPVASAVER

*::***********

**28 - DOMAIN cl02188/pfam07362 - CcdA**

NUCLEOTIDE ALIGNMENTS:

XCC0715|21229478_c859213-858908 TTGA----------------CAATGCGCATTCATGCGCATAGCGTGCGCA

XAC0769|21240774_c913584-913240 ATGT----------------TTCTGCGC----------AAAA-AAACCAT

XC_3520|66766352_4181328-4181633 TTGA----------------CAATGCGCATTCATGCGCATAGCGTGCGCA

XCV0820|78045556_c935339-935088 ATGAATCGCTATTACGACGTTTCAGCGC----------AGAAGAAGCCGC

XCR_0881|384425691_c921263-920964 ATG----------------------CGCATTCATGCGCATAGCGTGCGCA

XOC_0832|384417079_c836553-835987 ATGAACCGCTACTACGATGTTTCTGCGC----------AGAACAAGCCGC

XAC29_03925|470469929_c916998-916654 ATGT----------------TTCTGCGC----------AAAA-AAACCAT

** *** * * *

XCC0715|21229478_c859213-858908 TGAAT--GACG-GG-ATGCC-------------AATCATGAGCCGTTACT

XAC0769|21240774_c913584-913240 CGAATCTGACCGCC-ATGCCGACCTGGCGGCGCAAGCCCGCGCGATGACC

XC_3520|66766352_4181328-4181633 TGAAT--GACG-GG-ATGCC-------------AATCATGAGCCGTTACT

XCV0820|78045556_c935339-935088 TGAATCTGACCATCAATGCCGACCTGGCGGCGCAAGCCCGCGCGATGACT

XCR_0881|384425691_c921263-920964 TGAAT--GACG-GG-ATGCC-------------AATCATGAGCCGTTACT

XOC_0832|384417079_c836553-835987 TGAATCTGACCATCAACGCCGACCTGGCGGCGCAAGCCCGCACGCTGACC

XAC29_03925|470469929_c916998-916654 CGAATCTGACCGCC-ATGCCGACCTGGCGGCGCAAGCCCGCGCGATGACC

**** *** * *** ** * * * * **

XCC0715|21229478_c859213-858908 ACGACACTTCCGCGCAAA-----AAAAGCCGCTGAACCTGACCATCAACT

XAC0769|21240774_c913584-913240 GGCAAGCT-CTCGGCAAAGGTGGAAGAGTTGCTGGCCGAGTACATCGCCA

XC_3520|66766352_4181328-4181633 ACGACACTTCCGCGCAAA-----AAAAGCCGCTGAACCTGACCATCAACT

XCV0820|78045556_c935339-935088 GGCAACCT-TTCGGCAAAGGTGGAAGAGTTGCTGGCCGAGTACGTCACCA

XCR_0881|384425691_c921263-920964 ACGACACTTCCGCGCAAA-----AAAAGCCGCTGAACCTGACCATCAACT

XOC_0832|384417079_c836553-835987 GGCAATCT-CTCGGCAAAGGTCGAAGCGCTGCTGGCCGAGTACGTCACCA

XAC29_03925|470469929_c916998-916654 GGCAAGCT-CTCGGCAAAGGTGGAAGAGTTGCTGGCCGAGTACATCGCCA

* ** ***** ** * **** * * * ** *

XCC0715|21229478_c859213-858908 CGGAC-TTGGCCGC-GCAG-GCACGGGCGA-TGACCGGCAACCTGTCGGC

XAC0769|21240774_c913584-913240 AGGAACGCCACAGCCACAGCGCAGGAGCGCTTGAGCTGCAACGCG-CGAC

XC_3520|66766352_4181328-4181633 CGGAC-TTGGCCGC-GCAG-GCACGGGCGA-TGACCGGCAACCTGTCGGC

XCV0820|78045556_c935339-935088 AGGAACGCGACAACCACAGCGCCAGAGCGCTTGAGCTGCAACGCG-CCGC

XCR_0881|384425691_c921263-920964 CGGAC-TTGGCCGC-GCAG-GCACGGGCGA-TGACCGGCAACCTGTCGGC

XOC_0832|384417079_c836553-835987 AGGAACGCGACAGCCACAGCGCAAGAGCGCTTGCGCTGCAACGCG-CCGC

XAC29_03925|470469929_c916998-916654 AGGAACGCCACAGCCACAGCGCAGGAGCGCTTGAGCTGCAACGCG-CGAC

*** * * *** ** * *** ** * ***** * * *

XCC0715|21229478_c859213-858908 AAAGG--TCGAAGAACTGCT----GG---CCGACTACGTGACAAAAGAGC

XAC0769|21240774_c913584-913240 AAGCGAATGGAAAGCCTTCACCGAGGCGCACGGCTCCTTCGCCGACGAGT

XC_3520|66766352_4181328-4181633 AAAGG--TCGAAGAACTGCT----GG---CCGACTACGTGACAAAAGAGC

XCV0820|78045556_c935339-935088 GAGCGAATGGAAGGTCTTCACTGAAGCGCACGGCTCCTTCGCCGACGAGT

XCR_0881|384425691_c921263-920964 AAAGG--TCGAAGAACTGCT----GG---CCGACTACGTGACAAAAGAGC

XOC_0832|384417079_c836553-835987 GAGCGAATGGAAGGCCTTCACTGAGGCGCACGGCTCCTTCGCCGACGAGT

XAC29_03925|470469929_c916998-916654 AAGCGAATGGAAAGCCTTCACCGAGGCGCACGGCTCCTTCGCCGACGAGT

* * * *** ** * * ** ** * * * * ***

XCC0715|21229478_c859213-858908 G--CGACAGC-CACAGCG-CCAGAGCGATGGAGCT-GCAACGC--GCCGC

XAC0769|21240774_c913584-913240 T-TCCACGCT-GTGACCGACCAGCTCGATGTTCACGCCAATGTCGGCCAG

XC_3520|66766352_4181328-4181633 G--CGACAGC-CACAGCG-CCAGAGCGATGGAGCT-GCAACGC--GCCGC

XCV0820|78045556_c935339-935088 T-------------------------------------------------

XCR_0881|384425691_c921263-920964 G--CGACAGC-CACAGCG-CCAGAGCGATGGAGCT-GCAACGC--GCTGC

XOC_0832|384417079_c836553-835987 TCTCCACACTTATGACCGACCAGTTCGATGTCTACGCCAATGTCGGCCAG

XAC29_03925|470469929_c916998-916654 T-TCCACGCT-GTGACCGACCAGCTCGATGTTCACGCCAATGTCGGCCAG

XCC0715|21229478_c859213-858908 -AAGCGAATG-----------GAAGACCTTCAC-C-GACGCGCACGGC--

XAC0769|21240774_c913584-913240 AACACGAACAT-CCGTACGTGCTCGTCGTTCAGTCGAACATTTTCGAC--

XC_3520|66766352_4181328-4181633 -AAGCGAATG-----------GAAGACCTTCAC-C-GACGCGCACGGC--

XCV0820|78045556_c935339-935088 --------------------------------------------------

XCR_0881|384425691_c921263-920964 -AAGCGAATG-----------GAAGACCTTCAC-C-GACGCGTACGAC--

XOC_0832|384417079_c836553-835987 AACAAGAACATCCCGTACGTGGTCGTCGTTCAGTCGAAAATTTTCGACGC

XAC29_03925|470469929_c916998-916654 AACACGAACAT-CCGTACGTGCTCGTCGTTCAGTCGAACATTTTCGAC--

XCC0715|21229478_c859213-858908 --------------------------------------------------

XAC0769|21240774_c913584-913240 --------------------------------------------------

XC_3520|66766352_4181328-4181633 --------------------------------------------------

XCV0820|78045556_c935339-935088 --------------------------------------------------

XCR_0881|384425691_c921263-920964 --------------------------------------------------

XOC_0832|384417079_c836553-835987 CGCACCACGCCGCGTCGTCATCCCCCTGGTCCGAAAGTCCACGCAGTCCC

XAC29_03925|470469929_c916998-916654 --------------------------------------------------

XCC0715|21229478_c859213-858908 --------------------------------------------------

XAC0769|21240774_c913584-913240 --------------------------------------------------

XC_3520|66766352_4181328-4181633 --------------------------------------------------

XCV0820|78045556_c935339-935088 --------------------------------------------------

XCR_0881|384425691_c921263-920964 --------------------------------------------------

XOC_0832|384417079_c836553-835987 CGACCCCATCCCGCGTCACCCCGGAACTCACCGTGGCCGGCCACAGCGTC

XAC29_03925|470469929_c916998-916654 --------------------------------------------------

XCC0715|21229478_c859213-858908 --------------------------------------------------

XAC0769|21240774_c913584-913240 --------------------------------------------------

XC_3520|66766352_4181328-4181633 --------------------------------------------------

XCV0820|78045556_c935339-935088 --------------------------------------------------

XCR_0881|384425691_c921263-920964 --------------------------------------------------

XOC_0832|384417079_c836553-835987 ATCCTGCAACCGCTGGAAACGACTTCGGTGCCGCTGGCCGTGCTCAACAA

XAC29_03925|470469929_c916998-916654 --------------------------------------------------

XCC0715|21229478_c859213-858908 --------------------------------TCCTTCGC------C---

XAC0769|21240774_c913584-913240 --------------------------------ACCT-CGC----CAC---

XC_3520|66766352_4181328-4181633 --------------------------------TCCTTCGC------C---

XCV0820|78045556_c935339-935088 --------------------------------------------------

XCR_0881|384425691_c921263-920964 --------------------------------TCCTTCGC------C---

XOC_0832|384417079_c836553-835987 ACCTGCCGGCACATTGAAAGACCAGGGCCAACTCAT-CATCGACGCCCTG

XAC29_03925|470469929_c916998-916654 --------------------------------ACCT-CGC----CAC---

XCC0715|21229478_c859213-858908 GACGAGTTCTCCAC--GCT-------ATGA

XAC0769|21240774_c913584-913240 GCCGCGGCGGCGGC---CC-------CTGA

XC_3520|66766352_4181328-4181633 GACGAGTTCTCCAC--GCT-------ATGA

XCV0820|78045556_c935339-935088 --------CTCCAC--GCT-------ATGA

XCR_0881|384425691_c921263-920964 GACGAGTTCTCCAC--GCT-------ATGA

XOC_0832|384417079_c836553-835987 GAGGAGCTATTCACCCGCTCGTTCGGCTAG

XAC29_03925|470469929_c916998-916654 GCCGCGGCGGCGGC---CC-------CTGA

* * *

PROTEIN ALIGNMENTS:

XCC0715|AAM40030.1|conserved MTMRIHAHSVRMNDGMPIMSRYYDTSAQKKPLNLTINSDLAAQARAMTGN

XAC0769|AAM35657.1|conserved ------------------------MFLRKKTIESDRHADLAAQARAMTGK

XC_3520|AAY50563.1|conserved MTMRIHAHSVRMNDGMPIMSRYYDTSAQKKPLNLTINSDLAAQARAMTGN

XCV0820|CAJ22451.1|putative ------------------MNRYYDVSAQKKPLNLTINADLAAQARAMTGN

XCR_0881|AEL05798.1|conserved M--RIHAHSVRMNDGMPIMSRYYDTSAQKKPLNLTINSDLAAQARAMTGN

XOC_0832|AEQ95038.1|hypothetical ------------------MNRYYDVSAQNKPLNLTINADLAAQARTLTGN

XAC29_03925|AGH76311.1|hypothetical ------------------------MFLRKKTIESDRHADLAAQARAMTGK

::*.:: ::*******::**:

XCC0715|AAM40030.1|conserved LSAKVEELLADYVTKERDSHSARAMELQRAASEWKTFTDAHGSFADEFST

XAC0769|AAM35657.1|conserved LSAKVEELLAEYIAKERHSHSAGALELQRATSEWKAFTEAHGSFADEFPR

XC_3520|AAY50563.1|conserved LSAKVEELLADYVTKERDSHSARAMELQRAASEWKTFTDAHGSFADEFST

XCV0820|CAJ22451.1|putative LSAKVEELLAEYVTKERDNHSARALELQRAASEWKVFTEAHGSFADEFST

XCR_0881|AEL05798.1|conserved LSAKVEELLADYVTKERDSHSARAMELQRAASEWKTFTDAYDSFADEFST

XOC_0832|AEQ95038.1|hypothetical LSAKVEALLAEYVTKERDSHSARALALQRAASEWKAFTEAHGSFADEFST

XAC29_03925|AGH76311.1|hypothetical LSAKVEELLAEYIAKERHSHSAGALELQRATSEWKAFTEAHGSFADEFPR

****** ***:*::***..*** *: ****:****.**:*:.******.

XCC0715|AAM40030.1|conserved LXA-------------------------------C0--------------

XAC0769|AAM35657.1|conserved CDRPAR-CSRQCRPEHEHPYVLVVQSNIFDTSPRRGGG------------

XC_3520|AAY50563.1|conserved --------------------------------------------------

XCV0820|CAJ22451.1|putative --------------------------------------------------

XCR_0881|AEL05798.1|conserved LXO------------------------------C_0--------------

XOC_0832|AEQ95038.1|hypothetical LMTDQFDVYANVGQNKNIPYVVVVQSKIFDAAPRRVVIPLVRKSTQSPTP

XAC29_03925|AGH76311.1|hypothetical CDRPAR-CSRQCRPEHEHPYVLVVQSNIFDTSPRRGGG------------

XCC0715|AAM40030.1|conserved --------------------------------------------------

XAC0769|AAM35657.1|conserved --------------------------------------------------

XC_3520|AAY50563.1|conserved --------------------------------------------------

XCV0820|CAJ22451.1|putative --------------------------------------------------

XCR_0881|AEL05798.1|conserved --------------------------------------------------

XOC_0832|AEQ95038.1|hypothetical SRVTPELTVAGHSVILQPLETTSVPLAVLNKPAGTLKDQGQLIIDALEEL

XAC29_03925|AGH76311.1|hypothetical --------------------------------------------------

XCC0715|AAM40030.1|conserved ---769

XAC0769|AAM35657.1|conserved -----P

XC_3520|AAY50563.1|conserved -----L

XCV0820|CAJ22451.1|putative -----L

XCR_0881|AEL05798.1|conserved ---832

XOC_0832|AEQ95038.1|hypothetical FTRSFG

XAC29_03925|AGH76311.1|hypothetical -----P

**29 - DOMAIN cl03380/pfam01845 - CcdB**

NUCLEOTIDE ALIGNMENTS:

XCC0714|21229478_c858911-858594 ATGAC---------------------------------------------

XC_3521|66766352_4181630-4181947 ATGAC---------------------------------------------

XCV0819|78045556_c935091-934774 ATGA----------------------------------------------

XCR_0880|384425691_c920967-920650 ATGAC---------------------------------------------

XOC_0832|384417079_c836553-835987 ATGAACCGCTACTACGATGTTTCTGCGCAGAACAAGCCGCTGAATCTGAC

****

XCC0714|21229478_c858911-858594 ---------C----------------------------------------

XC_3521|66766352_4181630-4181947 ---------C----------------------------------------

XCV0819|78045556_c935091-934774 --------CC----------------------------------------

XCR_0880|384425691_c920967-920650 ---------C----------------------------------------

XOC_0832|384417079_c836553-835987 CATCAACGCCGACCTGGCGGCGCAAGCCCGCACGCTGACCGGCAATCTCT

*

XCC0714|21229478_c858911-858594 --------------------------------------------------

XC_3521|66766352_4181630-4181947 --------------------------------------------------

XCV0819|78045556_c935091-934774 --------------------------------------------------

XCR_0880|384425691_c920967-920650 --------------------------------------------------

XOC_0832|384417079_c836553-835987 CGGCAAAGGTCGAAGCGCTGCTGGCCGAGTACGTCACCAAGGAACGCGAC

XCC0714|21229478_c858911-858594 --------------------------------------------------

XC_3521|66766352_4181630-4181947 --------------------------------------------------

XCV0819|78045556_c935091-934774 --------------------------------------------------

XCR_0880|384425691_c920967-920650 --------------------------------------------------

XOC_0832|384417079_c836553-835987 AGCCACAGCGCAAGAGCGCTTGCGCTGCAACGCGCCGCGAGCGAATGGAA

XCC0714|21229478_c858911-858594 --------------------------------------------------

XC_3521|66766352_4181630-4181947 --------------------------------------------------

XCV0819|78045556_c935091-934774 --------------------------------------------------

XCR_0880|384425691_c920967-920650 --------------------------------------------------

XOC_0832|384417079_c836553-835987 GGCCTTCACTGAGGCGCACGGCTCCTTCGCCGACGAGTTCTCCACACTTA

XCC0714|21229478_c858911-858594 -----GACCAGTTCGATGTCTACGCGAACGTCGGCCAGAACAAGAACATC

XC_3521|66766352_4181630-4181947 -----GACCAGTTCGATGTCTACGCGAACGTCGGCCAGAACAAGAACATC

XCV0819|78045556_c935091-934774 -----GACCAGTTCGATGTCTATGCCAATGTCGGCCAGAACAAGAACATC

XCR_0880|384425691_c920967-920650 -----GACCAGTTCGATGTCTACGCGAACGTCGGCCAGAACAAGAACATC

XOC_0832|384417079_c836553-835987 TGACCGACCAGTTCGATGTCTACGCCAATGTCGGCCAGAACAAGAACATC

***************** ** ** *********************

XCC0714|21229478_c858911-858594 CCGTACGTGGTGGTCGTCCAGTCAAAGATCTTCGACGCCTCACCTCGCCG

XC_3521|66766352_4181630-4181947 CCGTACGTGGTGGTCGTCCAGTCAAAGATCTTCGACGCCTCACCTCGCCG

XCV0819|78045556_c935091-934774 CCGTACGTGGTGGTGGTCCAGTCGAAGATCTTCGACGCCTCTCCTCGCCG

XCR_0880|384425691_c920967-920650 CCGTACGTGGTGGTCGTCCAGTCAAAGATCTTCGACGCCTCACCTCGCCG

XOC_0832|384417079_c836553-835987 CCGTACGTGGTCGTCGTTCAGTCGAAAATTTTCGACGCCGCACCACGCCG

*********** ** ** ***** ** ** ********* * ** *****

XCC0714|21229478_c858911-858594 CGTCGTCGTGCCCCTGGTCCGAAAGTCCACCCACTCCCCGACCCCATCCC

XC_3521|66766352_4181630-4181947 CGTCGTCGTGCCCCTGGTCCGAAAGTCCACCCACTCCCCGACCCCATCCC

XCV0819|78045556_c935091-934774 CGTCGTCGTGCCCCTGGTCATCAAGTCCGGCCACTCCCCGACCCCATCCC

XCR_0880|384425691_c920967-920650 CGTCGTCGTGCCCCTGGTCCGAAAGTCCACCCACTCCCCGACCCCATCCC

XOC_0832|384417079_c836553-835987 CGTCGTCATCCCCCTGGTCCGAAAGTCCACGCAGTCCCCGACCCCATCCC

******* * ********* ****** ** ****************

XCC0714|21229478_c858911-858594 GCTTCACCCCGGAACTCACGGTGTCCGGCCACAGCGTCATCCTGCAGCCC

XC_3521|66766352_4181630-4181947 GCTTCACCCCGGAACTCACGGTGTCCGGCCACAGCGTCATCCTGCAGCCC

XCV0819|78045556_c935091-934774 GTTTCACCCCGGAACTCACCGTAGCCGGCCACAGCGTCATCCTGCAGCCT

XCR_0880|384425691_c920967-920650 GCTTCACCCCGGAACTCACGGTGTCCGGCCACAGCGTCATCCTGCAACCC

XOC_0832|384417079_c836553-835987 GCGTCACCCCGGAACTCACCGTGGCCGGCCACAGCGTCATCCTGCAACCG

* **************** ** ********************** **

XCC0714|21229478_c858911-858594 CTGGAAATGACCTCGGTGCCGCTGGCGGCGCTCAGCAAACCTGCCGGCTC

XC_3521|66766352_4181630-4181947 CTGGAAATGACCTCGGTGCCGCTGGCGGCGCTCAGCAAACCTGCCGGCTC

XCV0819|78045556_c935091-934774 CTGGAAATGACCTCGGTGCCGCTGGCCGTGCTCAACAAACCTGCCGGCAC

XCR_0880|384425691_c920967-920650 CTGGAAATGACCTCGGTGCCGCTGGCGGCGCTCAGCAAACCTGCCGGCTC

XOC_0832|384417079_c836553-835987 CTGGAAACGACTTCGGTGCCGCTGGCCGTGCTCAACAAACCTGCCGGCAC

******* *** ************** * ***** ************* *

XCC0714|21229478_c858911-858594 CCTGAAAGACCAAGGCCAAACCATCATCGACGCCCTGGACGAACTGTTCA

XC_3521|66766352_4181630-4181947 CCTGAAAGACCAAGGCCAAACCATCATCGACGCCCTGGACGAACTGTTCA

XCV0819|78045556_c935091-934774 ATTGAAGGACCAAGGCCAGATCATCATCGACGCCCTGGACGAGCTATTCA

XCR_0880|384425691_c920967-920650 CCTGAAAGACCAAGGCCAAACCATCATCGACGCCCTGGACGAACTGTTCA

XOC_0832|384417079_c836553-835987 ATTGAAAGACCAGGGCCAACTCATCATCGACGCCCTGGAGGAGCTATTCA

**** ***** ***** ****************** ** ** ****

XCC0714|21229478_c858911-858594 CCCGCTCATTCGGCTAA

XC_3521|66766352_4181630-4181947 CCCGCTCATTCGGCTAA

XCV0819|78045556_c935091-934774 CCCGCTCGTTCGGTTAA

XCR_0880|384425691_c920967-920650 CCCGCTCATTCGGCTAA

XOC_0832|384417079_c836553-835987 CCCGCTCGTTCGGCTAG

******* ***** **

PROTEIN ALIGNMENTS:

XCC0714|AAM40029.1|conserved --------------------------------------------------

XC_3521|AAY50564.1|conserved --------------------------------------------------

XCV0819|CAJ22450.1|putative --------------------------------------------------

XCR_0880|AEL05797.1|conserved --------------------------------------------------

XOC_0832|AEQ95038.1|hypothetical MNRYYDVSAQNKPLNLTINADLAAQARTLTGNLSAKVEALLAEYVTKERD

XCC0714|AAM40029.1|conserved ---------------------------------MTDQFDVYANVGQNKNI

XC_3521|AAY50564.1|conserved ---------------------------------MTDQFDVYANVGQNKNI

XCV0819|CAJ22450.1|putative ---------------------------------MTDQFDVYANVGQNKNI

XCR_0880|AEL05797.1|conserved ---------------------------------MTDQFDVYANVGQNKNI

XOC_0832|AEQ95038.1|hypothetical SHSARALALQRAASEWKAFTEAHGSFADEFSTLMTDQFDVYANVGQNKNI

*****************

XCC0714|AAM40029.1|conserved PYVVVVQSKIFDASPRRVVVPLVRKSTHSPTPSRFTPELTVSGHSVILQP

XC_3521|AAY50564.1|conserved PYVVVVQSKIFDASPRRVVVPLVRKSTHSPTPSRFTPELTVSGHSVILQP

XCV0819|CAJ22450.1|putative PYVVVVQSKIFDASPRRVVVPLVIKSGHSPTPSRFTPELTVAGHSVILQP

XCR_0880|AEL05797.1|conserved PYVVVVQSKIFDASPRRVVVPLVRKSTHSPTPSRFTPELTVSGHSVILQP

XOC_0832|AEQ95038.1|hypothetical PYVVVVQSKIFDAAPRRVVIPLVRKSTQSPTPSRVTPELTVAGHSVILQP

*************:*****:*** ** :******.******:********

XCC0714|AAM40029.1|conserved LEMTSVPLAALSKPAGSLKDQGQTIIDALDELFTRSFG

XC_3521|AAY50564.1|conserved LEMTSVPLAALSKPAGSLKDQGQTIIDALDELFTRSFG

XCV0819|CAJ22450.1|putative LEMTSVPLAVLNKPAGTLKDQGQIIIDALDELFTRSFG

XCR_0880|AEL05797.1|conserved LEMTSVPLAALSKPAGSLKDQGQTIIDALDELFTRSFG

XOC_0832|AEQ95038.1|hypothetical LETTSVPLAVLNKPAGTLKDQGQLIIDALEELFTRSFG

** ******.*.****:****** *****:********
