# Supplementary figures and images for: Type II Toxin-Antitoxin Distribution and Adaptive Aspects on Xanthomonas Genomes: Focus on Xanthomonas citri
Source: Front Microbiol. 2016 May 10;7:652. doi: 10.3389/fmicb.2016.00652 (PMC4861877; doi:10.3389/fmicb.2016.00652)

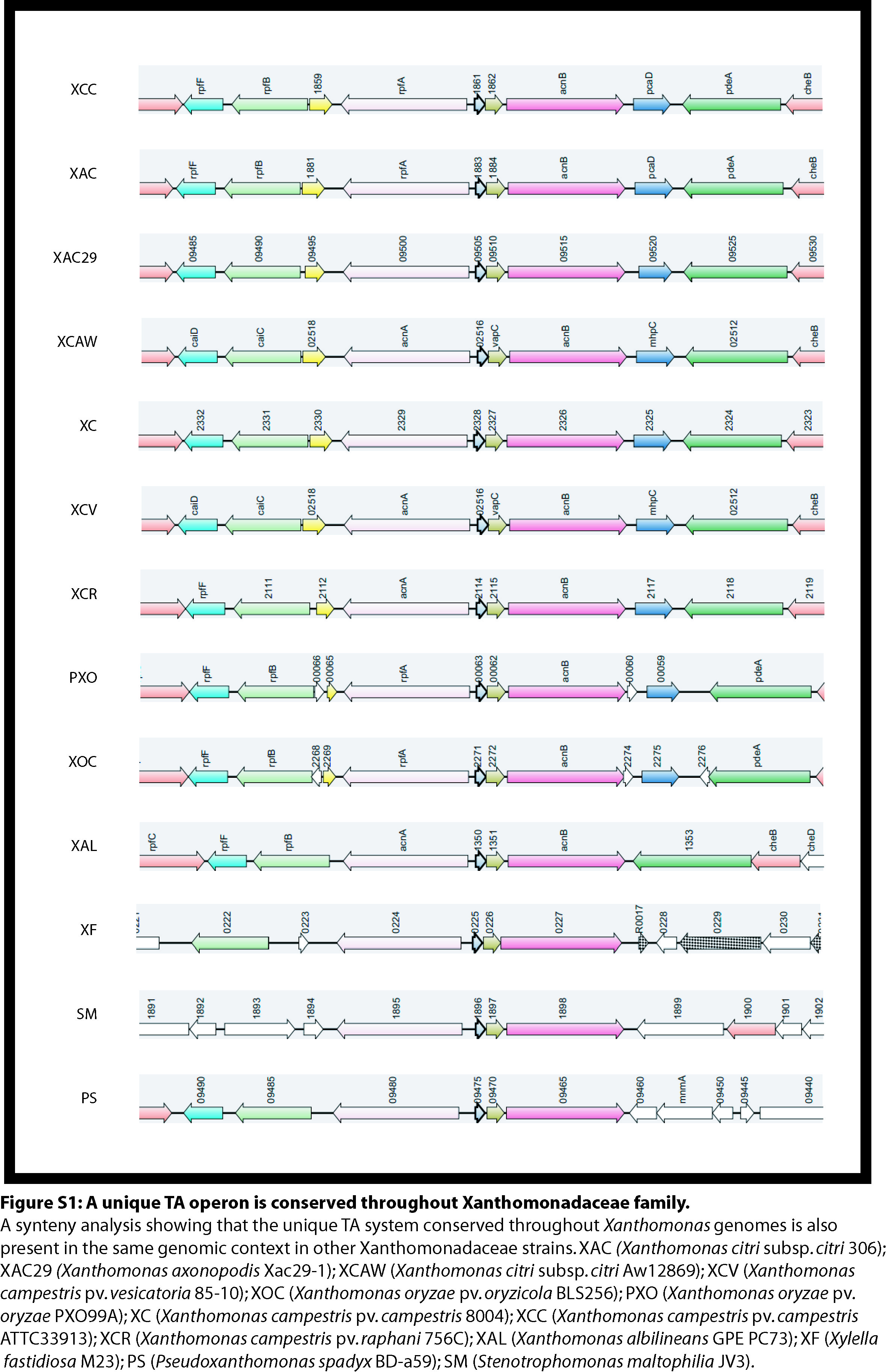

Supplement: Supplementary file 5 [file Image1.JPEG]
